# ESPLSM: An Efficient and Interpretable Mediation Analysis Framework Using Sparse Envelopes

**DOI:** 10.1002/sim.70464

**Published:** 2026-03-18

**Authors:** Yeonhee Park, Zhihua Su

**Affiliations:** ^1^ Department of Statistics Sungkyunkwan University Seoul South Korea; ^2^ nVerses Capital, LLC Wellington Florida USA

**Keywords:** cell line data analysis, dimension reduction, mediation, multivariate regression, partial least squares

## Abstract

Mediation analysis is a fundamental tool for understanding biological mechanisms through which an exposure exerts its effect on an outcome via intermediate variables, or mediators. However, modern biomedical studies often involve multiple exposures and mediators with complex correlation structures, and may also involve multiple outcomes, as in multi‐omics or imaging studies, where existing mediation analyses can suffer from instability and limited interpretability. In this work, we propose Envelope‐Based Sparse Partial Least Squares for Mediation Analysis (ESPLSM), which integrates dimension reduction and sparsity enforcement via the sparse envelope model to improve estimation and interpretation of causal effects. We embed the envelope model within the causal mediation framework based on potential outcomes, which allows us to formally define and identify direct and indirect effects and to establish theoretical guarantees, including asymptotic efficiency and selection consistency. Through simulation studies, we show that ESPLSM outperforms existing methods in terms of estimation accuracy, statistical power, and variable selection. Finally, we apply ESPLSM to a cancer cell line dataset to investigate the role of RNA expression in mediating the effect of EGFR mutations on drug responses. Our results provide new insights into the molecular mechanisms underlying targeted cancer therapies. Overall, ESPLSM provides a statistically principled yet practical solution for interpretable and efficient mediation analysis in modern high‐dimensional biomedical applications.

## 1 Introduction

Mediation analysis is a fundamental statistical tool for understanding causal mechanisms, allowing researchers to investigate how exposure variables influence outcomes through one or more mediators. By decomposing the total effect into direct and indirect effects, mediation analysis provides valuable insights into whether intermediate variables transmit the effect of exposures to outcomes and to what extent. Traditionally, mediation analysis has been guided by the framework of [1]. While foundational, this approach has known limitations, particularly in the presence of exposure‐mediator interactions or nonlinearities, and does not guarantee a causal interpretation of the estimated effects. To address these gaps, the modern causal mediation analysis framework was developed using counterfactuals, providing formal definitions for the natural direct effect and natural indirect effect. The identification of these causal pathways, however, relies on strong, untestable assumptions about confounding [2].

To motivate the proposed methodology for mediation analysis, we consider the cancer cell line datasets from 612 subjects in Reference [3] collected by Genomic of Drug Sensitivity in Cancer [4] and Cancer Cell Line Encyclopedia [5] study cohorts. These datasets provide an opportunity to investigate the relationship between genetic and transcriptomic data (e.g., DNA mutations and RNA expression levels) and responses to targeted treatments for a specific targeted pathway. The study by [3] demonstrates that incorporating RNA expression data alongside traditional DNA mutation information can significantly enhance the prediction of targeted therapy responses. Specifically, RNA expression signatures can complement traditional DNA‐based approaches by identifying tumors where the target pathway is activated through phenocopying mutations. Building on these findings, we consider mediation analysis as a framework to further understand the underlying mechanisms linking genetic alterations to drug response. By quantifying (1) the direct effect of an independent variable (e.g., a genetic mutation) on drug response (the dependent variable) and (2) the indirect effect, where the mutation influences RNA expression (the mediator), which in turn affects drug response, mediation analysis provides deeper insights into the mechanisms driving therapeutic sensitivity. While observational studies alone can only suggest the presence of mediation effects, they serve as a foundation for further validation through follow‐up experiments or longitudinal studies, enabling a more rigorous and comprehensive understanding of the biological pathways involved in targeted treatment response.

A significant frontier in this field is mediation analysis with high‐dimensional mediators, where a large vector of variables is considered simultaneously. In this context, standard methods are computationally and theoretically challenged. Classical approaches, such as ordinary least squares (OLS) estimation, are not well‐suited for high‐dimensional mediators due to inefficiency, overfitting, and an inability to perform variable selection. Models based on Partial Least Squares (PLS) fail to leverage structural dependencies among mediators and may include irrelevant variables, leading to suboptimal inference. While Sparse Partial Least Squares (SPLS) performs variable selection, it does not explain the estimates of indirect effects. The recent advancements in mediation analysis have addressed some of these challenges [6] For instance, [7, 8] propose high‐dimensional mediation frameworks that enhance the detection of outcome‐side active mediators by modeling multiple mediators jointly and adjusting for confounding effects. Reference [9] develops a penalized approach combined with principal components analysis, which focuses on the indirect effect for mediation analysis with both high‐dimensional exposures and high‐dimensional mediators. Reference [10] models latent factor structures to account for unobserved variables and identifies potential mediators based on the max‐squared test. Reference [11] proposes the partial sum statistics and sample splitting strategy framework to improve the test to detect the global indirect effect and prioritize outcome‐side active mediators. However, these approaches primarily focus on variable selection and estimation of indirect effects, often treating the direct effect as secondary or focusing on indirect pathways for inference. While such designs are well‐suited for identifying mediators that contribute to indirect effects, they can result in unstable finite‐sample behavior and slow empirical convergence of the outcome model estimators (e.g., direct effect and outcome‐relevant mediator effect).

We propose Envelope‐based Sparse Partial Least Squares for Mediation Analysis (ESPLSM), a novel approach that integrates the predictor envelope models [12, 13] with sparsity methods [14, 15], and is motivated by the causal mediation framework to enhance biomedical interpretability and practical utility in mediation studies. Unlike penalized regression approaches that rely solely on sparsity for mediator selection, ESPLSM provides an interpretable low‐dimensional subspace that captures material variation relevant to causal pathways. By removing immaterial variation and imposing sparsity, ESPLSM simultaneously improves both estimation efficiency and identification of scientifically meaningful mediators. Compared to existing methods, we make several key contributions. First, we propose a mediation model that jointly characterizes direct and indirect effects while accommodating dependence among mediators, and naturally extends to settings with multiple exposures and multiple outcomes. This generality enables mediation analysis for complex causal structures beyond conventional single‐exposure, single‐outcome settings. Second, the model incorporates sparsity constraints on the mediators, enabling the identification of a parsimonious set of mediators that are most relevant to the indirect pathway. Mediator selection is conducted primarily through the outcome model, prioritizing outcome‐side active mediators that contribute to variation in the indirect effect, which can enhance interpretability and stability in the presence of correlated mediators. Third, we establish the root n‐consistency and oracle properties of the causal estimators under the ESPLSM framework, which enable asymptotically valid Wald‐type hypothesis testing for both direct and indirect effects within a single estimation procedure, and demonstrate its superior performance in estimation accuracy, statistical power, and variable selection through extensive simulations. Lastly, applying ESPLSM to an EGFR pathway dataset from cancer cell lines uncovers new insights into how RNA expression mediates the effects of EGFR mutations on targeted drug responses, contributing to a better understanding of mechanisms relevant to precision medicine.

The rest of the paper is organized as follows. Section 2 provides a brief review of the envelope model, laying the foundation for our proposed method. Section 3 introduces the ESPLSM, detailing its formulation, estimation procedure, and theoretical properties. Section 4 presents simulation studies that evaluate the performance of ESPLSM in terms of estimation accuracy, variable selection, and statistical power, comparing it to existing methods. Section 5 applies ESPLSM to a cancer cell line dataset, investigating how RNA expression mediates the effect of EGFR mutations on drug response, providing biologically meaningful insights. Section 6 concludes the paper with a discussion on the implications of our findings and potential directions for future research.

## 2 Review of the Envelope Model

Consider a linear regression model $Y=\mu_{Y}+\beta^{T}(X-\mu_{X})+\epsilon$, where $Y\in {\mathbb{R}}^{r}$ is the univariate response (r=1) or multivariate response vector (r>1) with mean $\mu_{Y}$, $X\in {\mathbb{R}}^{p}$ is the predictor vector with mean $\mu_{X}$ and covariance matrix $\sum_{X}$, $\epsilon \in {\mathbb{R}}^{r}$ denotes the error vector with mean 0 and covariance matrix $\sum_{Y|X}$, and $\beta \in {\mathbb{R}}^{p\times r}$ denotes unknown regression coefficients. Reference [12] proposed the predictor envelope model, assuming that part of X is immaterial to the linear regression and does not affect the distribution of Y directly or indirectly under the linear regression model. Specifically, the predictor envelope model assumes that there is a subspace $𝒮\subseteq {\mathbb{R}}^{p}$ such that (a) $\text{cov}(Y,Q_{𝒮}X|P_{𝒮}X)=0$ and (b) $\text{cov}(P_{𝒮}X,Q_{𝒮}X)=0$, where $P_{\cdot }$ denotes the projection matrix, I denotes the identity matrix, and $Q_{\cdot }=I-P_{\cdot }$. The assumptions (a) and (b) are equivalent to imposing the following structure to the model parameters: (c) span(β)⊆𝒮 and (d) $\sum_{X}=P_{𝒮}\sum_{X}P_{𝒮}+Q_{𝒮}\sum_{X}Q_{𝒮}$. In (c), the structure of β asserts that the span of β is contained in 𝒮. When $\sum_{X}$ can be decomposed as in (d), then 𝒮 is called a reducing subspace of $\sum_{X}$ [16]. Then, the predictor envelope, i.e., the $\sum_{X}$‐envelope of β, is defined as the smallest reducing subspace that contains span(β). For convenience, the $\sum_{X}$‐envelope of β is denoted by ${ℰ}_{\sum_{X}}(\beta )$. We call $P_{{ℰ}_{\sum_{X}}(\beta )}X$ the material part of X and $Q_{{ℰ}_{\sum_{X}}(\beta )}X$ the immaterial part.

Reference [12] showed that partial least squares (PLS) can be viewed as an envelope estimation procedure based on a moment‐based method, whereas the envelope model provides a likelihood‐based formulation of the same subspace. Thus, the two frameworks share a common goal of extracting predictive information from a lower‐dimensional subspace.

Reference [13] extends this idea to the partial predictor envelope model, which imposes the envelope structure only on a subset of predictors that carry material information for the response. By partitioning $X=(X_{1},X_{2})$ and applying the envelope structure to $X_{1}$ while adjusting for $X_{2}$, the model enables dimension reduction while retaining modeling flexibility. This partial envelope framework serves as the foundation for the proposed method, linking PLS and envelope methods.

## 3 Envelope‐Based Sparse Partial Least Squares for Mediation Analysis

### 3.1 Formulation for Mediation Analysis

Mediation analysis is a statistical method used to investigate the underlying mechanisms or pathways through which exposure variables $X={\left(X_{1},\ldots ,X_{k}\right)}^{T}\in {\mathbb{R}}^{k}$ influence dependent variables $Y={\left(Y_{1},\ldots ,Y_{r}\right)}^{T}\in {\mathbb{R}}^{r}$ by examining the role of one or more intermediate variables, known as mediators $M={\left(M_{1},\ldots ,M_{p}\right)}^{T}\in {\mathbb{R}}^{p}$.

**FIGURE 1:**
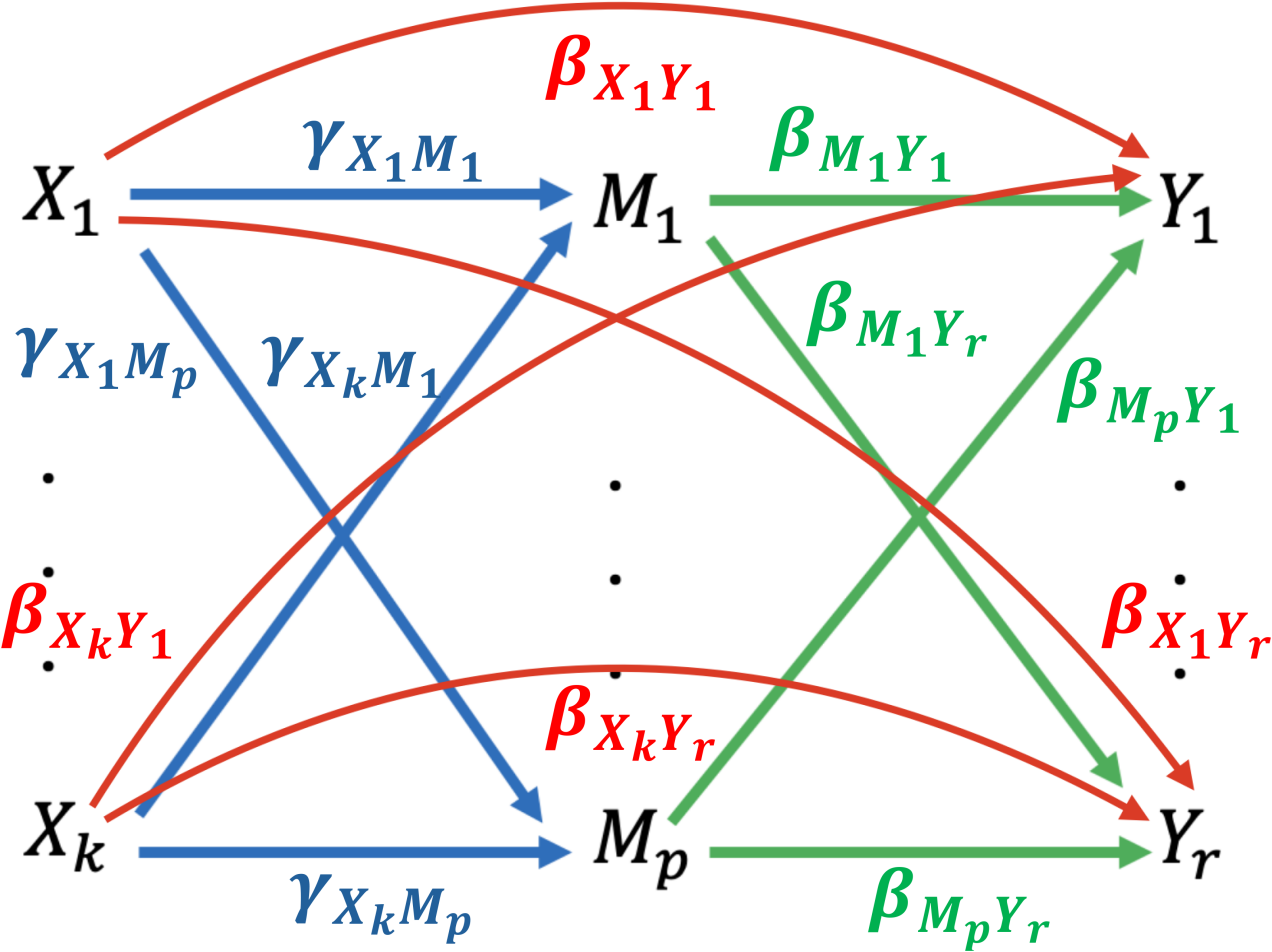
Schema of the model with k exposure variables $X_{1},\ldots ,X_{k}$, p mediators $M_{1},\ldots ,M_{p}$, and r outcome variables $Y_{1},\ldots ,Y_{r}$.

Figure 1 illustrates the underlying causal structure among the variables X, M, and Y. The total effect of X on Y can be decomposed into the direct effect and the indirect effect through the mediators M. The direct effect represents the pathway from X to Y that is not mediated by M, whereas the indirect effect captures the portion of the total effect transmitted through M. This decomposition allows for a mechanistic interpretation of how exposures influence the outcomes.

Baron and Kenny [1] illustrates a simple mediation model using the linear regression equations, assuming normality of the errors for univariate variables. To extend this formulation to the multivariate case and infer causality and achieve identifiable mediation effects [2, 17], we consider the following multivariate linear regression model with confounding variables $Z\in {\mathbb{R}}^{q}$:

(1) $$\begin{array}{ll} Y & =\mu_{Y}+\beta_{M}^{T}\left(M-\mu_{M}\right)+\beta_{X}^{T}\left(X-\mu_{X}\right)+\beta_{Z}^{T}\left(Z-\mu_{Z}\right)+\epsilon ,\\ M & =\mu_{M}+\gamma_{X}^{T}\left(X-\mu_{X}\right)+\gamma_{Z}^{T}\left(Z-\mu_{Z}\right)+e, \end{array}$$

where $\mu_{Y},\mu_{M},\mu_{X},\mu_{Z}$ denote the mean of Y, M, X, Z, respectively, $\beta_{M}\in {\mathbb{R}}^{p\times r},\beta_{X}\in {\mathbb{R}}^{k\times r},\beta_{Z}\in {\mathbb{R}}^{q\times r}$, $\gamma_{X}\in {\mathbb{R}}^{k\times p},\gamma_{Z}\in {\mathbb{R}}^{q\times p}$ denote unknown regression coefficients, ϵ denotes the error vector with mean **0** and covariance matrix $\sum_{Y|M,X,Z}$ and is assumed to be independent of M, X, and Z, and e denotes the error vector with mean **0** and covariance matrix $\sum_{M|X,Z}$ and is assumed to be independent of ϵ, X, and Z. Here, the coefficient $\beta_{X}^{T}$ denotes the direct effect of X on Y. The coefficients $\gamma_{X}^{T}$ and $\beta_{M}^{T}$ correspond to the causal pathways X→M and M→Y, respectively. Consequently, the indirect effect of X on Y through M is expressed as ${\left(\gamma_{X}\beta_{M}\right)}^{T}$.

To address the challenges arising from high‐dimensional mediators and to improve estimation efficiency, we adopt a dimension reduction strategy for mediator effects. We assume that there exists a subspace 𝒮 of ${\mathbb{R}}^{p}$ such that

(2) $$\begin{array}{ll} & (i)\;\text{cov}\left(Y,Q_{𝒮}M|P_{𝒮}M,X,Z\right)=0\;\text{and}\\ & (\mathrm{ii})\;\text{cov}\left(Q_{𝒮}M,P_{𝒮}M|X,Z\right)=0. \end{array}$$

The smallest subspace 𝒮 of ${\mathbb{R}}^{p}$ satisfying the conditions (2) is the envelope we are interested in for mediation analysis, denoted by ${ℰ}_{\sum_{M|X,Z}}(\beta_{M})$ or ℰ for short. Then, we have (iii) $\text{span}(\beta_{M})\subseteq ℰ$ and (iv) $\sum_{M|X,Z}=P_{ℰ}\sum_{M|X,Z}P_{ℰ}+Q_{ℰ}\sum_{M|X,Z}Q_{ℰ}$. Let d denote the dimension of ℰ with 0≤d≤p, $\Gamma \in {\mathbb{R}}^{p\times d}$ an orthonormal basis of ℰ and $\Gamma_{0}\in {\mathbb{R}}^{p\times (p-d)}$ an orthonormal basis of ${ℰ}^{⊥}$. Then, the coordinate form of the linear regression model (1) with (iii) and (iv) is

(3) $$\begin{array}{l} \begin{array}{cc} & Y=\mu_{Y}+\eta^{T}\Gamma^{T}\left(M-\mu_{M}\right)+\beta_{X}^{T}\left(X-\mu_{X}\right)+\beta_{Z}^{T}\left(Z-\mu_{Z}\right)+\epsilon ,\\ & M=\mu_{M}+\gamma_{X}^{T}\left(X-\mu_{X}\right)+\gamma_{Z}^{T}\left(Z-\mu_{Z}\right)+e\;\text{and}\\ & \sum_{M|X,Z}=\Gamma \Omega \Gamma^{T}+\Gamma_{0}\Omega_{0}\Gamma_{0}^{T}, \end{array} \end{array}$$

where $\beta_{M}=\Gamma \eta$ and $\eta \in {\mathbb{R}}^{d\times r}$ carries the coordinates of $\beta_{M}$ with respect to Γ. In the decomposition of the covariance matrix, the positive definite matrices $\Omega \in {\mathbb{R}}^{d\times d}$ and $\Omega_{0}\in {\mathbb{R}}^{\left(p-d)\times (p-d\right)}$ contain the coordinates of $\sum_{M|X,Z}$ with respect to Γ and $\Gamma_{0}$ and represent the variability associated with the material and immaterial parts of the mediators, respectively.

This envelope‐based parameterization (3) provides a structured representation of the causal mechanisms. The total effect of exposures X on Y is decomposed as $\beta_{X}^{T}+{\left(\gamma_{X}\Gamma \eta \right)}^{T}$, where $\beta_{X}^{T}$ represents the direct causal pathway X→Y, and ${\left(\gamma_{X}\beta_{M}\right)}^{T}={\left(\gamma_{X}\Gamma \eta \right)}^{T}$ corresponds to the indirect pathway X→M→Y captured within the material subspace. Variation in the immaterial subspace spanned by $\Gamma_{0}$ does not affect mediation and thus improves estimation efficiency by removing irrelevant variation. Following Park et al. [13], we refer to this model as the envelope‐based partial least squares for mediation analysis (EPLSM) to highlight its application in mediation analysis. The identification results of causal effects showing the interpretation is valid under EPLSM are provided in Section 3.3.1. Also, the regression coefficients in the EPLSM serve as consistent estimators of the direct and indirect causal effects, as formally established in Section 3.3.2. This formulation provides a framework that connects envelope models with causal mediation inference.

We define causally active mediators as those satisfying contributing to both the exposure‐mediator and mediator‐outcome pathways, i.e., satisfying both $\gamma_{X}\neq \text{0}$ and $\beta_{M}\neq \text{0}$, while mediators with either coefficient equal to zero are considered causally inactive. Motivated by high‐dimensional mediation analysis [7, 18], we assume that X and Z are fixed‐dimensional and the model (3) has sparse mediation effects, i.e., we impose sparsity on $\beta_{M}$, but not on $\beta_{X}$ and $\beta_{Z}$. A mediator is defined as sparsity‐selected if the corresponding row in Γ is nonzero, indicating that it lies in the material subspace that drives the outcome variation, and sparsity‐excluded otherwise. Thus, sparsity‐excluded mediators are outcome‐irrelevant in the sense that they do not have active outcome‐side effects under the EPLSM framework, although they may still be associated with the exposure. Under the EPLSM framework, sparsity is primarily imposed on $\beta_{M}$ through the envelope structure, ensuring that only mediators with active outcome‐side effects are retained, while still allowing flexibility to incorporate additional sparsity on $\gamma_{X}$ if desired. Without loss of generality, we can write $M={\left(M_{𝒜}^{T},M_{ℐ}^{T}\right)}^{T}$, where $M_{𝒜}\in {\mathbb{R}}^{p_{𝒜}}$ denotes the vector of sparsity‐selected mediators and $M_{ℐ}\in {\mathbb{R}}^{p_{ℐ}}$ denotes the vector of sparsity‐excluded mediators with $p_{𝒜}+p_{ℐ}=p$. Then, Γ has a sparse structure, i.e.,

(4) $$\Gamma =\left(\begin{array}{l} \Gamma_{𝒜}\\ \text{0} \end{array}\right),$$

and thus $\beta_{M}=\Gamma \eta$ has a sparse structure and $\beta_{M,𝒜}=\Gamma_{𝒜}\eta$ denotes the coefficients for sparsity‐selected mediators. We refer to the EPLSM in Equation (3) as the envelope‐based sparse partial least squares for mediation analysis (ESPLSM) if Γ has the sparse structure (4). In other words, ESPLSM is the EPLSM with the sparse vector of $\beta_{M}$.

### 3.2 Estimation

We use the normal likelihood as an objective function for estimation. Let $\left\{M_{i},X_{i},Z_{i},Y_{i}\right\}$, i=1,…,n be n independent observations from the EPLSM. Let ${𝕄}^{T}=(M_{1}^{T},M_{2}^{T},\ldots ,M_{n}^{T})\in {\mathbb{R}}^{p\times n}$, ${𝕏}^{T}=(X_{1}^{T},X_{2}^{T},\ldots ,X_{n}^{T})\in {\mathbb{R}}^{k\times n}$, ${\mathbb{Z}}^{T}=(Z_{1}^{T},Z_{2}^{T},\ldots ,Z_{n}^{T})\in {\mathbb{R}}^{q\times n}$, and ${𝕐}^{T}=(Y_{1}^{T},Y_{2}^{T},\ldots ,Y_{n}^{T})\in {\mathbb{R}}^{r\times n}$ be the data matrices. For a fixed dimension d of the EPLSM, the normal likelihood is given by

(5) $$\begin{array}{l} \begin{array}{cc} l & =\frac{n(r+p)}{2}\log(2\pi )-\frac{n}{2}\log|\Omega |-\frac{n}{2}\log|\Omega_{0}|\\ & \;-\frac{1}{2}\;\mathrm{tr}\left[\left\{𝕄-1_{n}\mu_{M}^{T}-\left(𝕏-1_{n}{\bar{X}}^{T}\right)\gamma_{X}-\left(\mathbb{Z}-1_{n}{\bar{Z}}^{T}\right)\gamma_{Z}\right\}\right.\\ & \;\times {\left(\Gamma \Omega \Gamma^{T}+\Gamma_{0}\Omega_{0}\Gamma_{0}^{T}\right)}^{-1}\left\{𝕄-1_{n}\mu_{M}^{T}-\left(𝕏-1_{n}{\bar{X}}^{T}\right)\gamma_{X}\right.\\ & \;\left.{\left.-\left(\mathbb{Z}-1_{n}{\bar{Z}}^{T}\right)\gamma_{Z}\right\}}^{T}\right]-\frac{n}{2}\log|\sum_{Y|M,X,Z}|\\ & \;-\frac{1}{2}\;\mathrm{tr}\left[\left\{𝕐-1_{n}\mu_{Y}^{T}-\left(𝕄-1_{n}\mu_{M}^{T}\right)\Gamma \eta -\left(𝕏-1_{n}{\bar{X}}^{T}\right)\beta_{X}\right.\right.\\ & \;\left.-\left(\mathbb{Z}-1_{n}{\bar{Z}}^{T}\right)\beta_{Z}\right\}\sum_{Y|M,X,Z}^{-1}\left\{𝕐-1_{n}\mu_{Y}^{T}\right.\\ & \left.{\left.-\left(𝕄-1_{n}\mu_{M}^{T}\right)\Gamma \eta -\left(𝕏-1_{n}{\bar{X}}^{T}\right)\beta_{X}-\left(\mathbb{Z}-1_{n}{\bar{Z}}^{T}\right)\beta_{Z}\right\}}^{T}\right], \end{array} \end{array}$$

where $\bar{X}=(1/n)\sum_{i=1}^{n}X_{i}$ and $\bar{Z}=(1/n)\sum_{i=1}^{n}Z_{i}$ denote the sample mean of X and Z, respectively, $1_{n}\in {\mathbb{R}}^{n}$ denotes an n‐dimensional vector of 1's, and tr(A) denotes the trace of a matrix A. Then, ${𝕄}_{c}=𝕄-1_{n}{\bar{M}}^{T}$, ${𝕎}_{c}=𝕎-1_{n}{\bar{W}}^{T}$, and ${𝕐}_{c}=𝕐-1_{n}{\bar{Y}}^{T}$ are the centered data matrices for 𝕄, 𝕎, and 𝕐, respectively, where 𝕎=(𝕏,ℤ). Let $S_{M|X,Z}=(1/n){𝕄}_{c}^{T}Q_{{𝕎}_{c}}{𝕄}_{c}$, $S_{Y|X,Z}=(1/n){𝕐}_{c}^{T}Q_{{𝕎}_{c}}{𝕐}_{c}$, and $S_{(Y,M)|X,Z}=(1/n){𝕐}_{c}^{T}Q_{{𝕎}_{c}}{𝕄}_{c}$ denote the sample conditional variance of M given X and Z, the sample conditional variance of Y given X and Z, and the sample conditional covariance between Y and M given X and Z, respectively. Then, $S_{M|Y,X,Z}=S_{M|X,Z}-S_{(Y,M)|X,Z}^{T}S_{Y|X,Z}^{-1}S_{(Y,M)|X,Z}$. To estimate the parameters, we first fix an orthonormal basis Γ and estimate other parameters by maximizing the objective function (5). The estimators of these parameters can be written as explicit functions of Γ (See equations (8) below). We then substitute them back to (5) and obtain the objective function

(6) $$\begin{array}{l} \underset{\text{span}(\Gamma )\in {ℒ}_{p\times d}}{\text{argmin}}\left\{\log|S_{Y|X,Z}|+\log|\Gamma^{T}S_{M|X,Z}^{-1}\Gamma |+\log|\Gamma^{T}S_{M|Y,X,Z}\Gamma |\right\}, \end{array}$$

where ${ℒ}_{p\times d}$ denotes a p×d Grassmann manifold.

To make the EPLSM estimator of $\beta_{M}$ a sparse estimator for the ESPLSM, we add an adaptive group lasso penalty to the objective function (6), which results in the sparsity in Γ. The estimator of the ESPLSM can be obtained by solving the optimization problem

(7) $$\begin{array}{ll} \hat{ℰ} & \equiv \underset{\text{span}(\Gamma )\in {ℒ}_{p\times d}}{\text{argmin}}\left\{\log|S_{Y|X,Z}|+\log|\Gamma^{T}S_{M|X,Z}^{-1}\Gamma |\right.\\ & \;\left.+\log|\Gamma^{T}S_{M|Y,X,Z}\Gamma |+\lambda \overset{p-d}{\underset{i=1}{\sum }}w_{i}\|\Gamma_{i}{\|}_{2}\right\}, \end{array}$$

where $\Gamma_{i}$ denotes the ith row of Γ, $\|\cdot {\|}_{2}$ denotes the norm of a vector, λ is the tuning parameters, and $w_{i}$'s are the adaptive weights.

Once we have the solution of (6) or (7) for the EPLSM or the ESPLSM, respectively, $\hat{\Gamma }$ can be taken to be any orthonormal basis of the solution of (6) or (7), and ${\hat{\Gamma }}_{0}$ can be taken to be any completion of $\hat{\Gamma }$. Then the estimators of the parameters for the EPLSM or the ESPLSM are

(8) $$\begin{array}{l} \begin{array}{cc} & {\hat{\mu }}_{Y}=\bar{Y},{\hat{\mu }}_{X}=\bar{X},\;{\hat{\mu }}_{Z}=\bar{Z},{\hat{\mu }}_{M}=\bar{M},\\ & {\hat{\gamma }}_{X}=S_{X|Z}^{-1}\left(S_{XM}-S_{XZ}S_{Z}^{-1}S_{ZM}\right)=S_{X|Z}^{-1}S_{(X,M)|Z},\\ & {\hat{\gamma }}_{Z}=S_{Z|X}^{-1}\left(S_{ZM}-S_{ZX}S_{X}^{-1}S_{XM}\right)=S_{Z|X}^{-1}S_{(Z,M)|X},\\ & \hat{\eta }={\left({\hat{\Gamma }}^{T}R_{M|X,Z}^{T}R_{M|X,Z}\hat{\Gamma }\right)}^{-1}{\hat{\Gamma }}^{T}R_{M|X,Z}^{T}R_{Y|X,Z},\\ & {\hat{\beta }}_{X}=S_{X|Z}^{-1}\left\{S_{XY}-S_{XM}{\hat{\beta }}_{M}-S_{XZ}S_{Z}^{-1}\left(S_{ZY}-S_{ZM}{\hat{\beta }}_{M}\right)\right\}\\ & \;=S_{X|Z}^{-1}\left(S_{(X,Y)|Z}-S_{(X,M)|Z}{\hat{\beta }}_{M}\right)\\ & {\hat{\beta }}_{Z}=S_{Z|X}^{-1}\left\{S_{ZY}-S_{ZM}{\hat{\beta }}_{M}-S_{ZX}S_{X}^{-1}\left(S_{XY}-S_{XM}{\hat{\beta }}_{M}\right)\right\}\\ & \;=S_{Z|X}^{-1}\left(S_{(Z,Y)|X}-S_{(Z,M)|X}{\hat{\beta }}_{M}\right)\\ & \hat{\Omega }=(1/n){\hat{\Gamma }}^{T}R_{M|X,Z}^{T}R_{M|X,Z}\hat{\Gamma }={\hat{\Gamma }}^{T}S_{M|X,Z}\hat{\Gamma },\\ & {\hat{\Omega }}_{0}=(1/n){\hat{\Gamma }}_{0}^{T}R_{M|X,Z}^{T}R_{M|X,Z}{\hat{\Gamma }}_{0}={\hat{\Gamma }}_{0}^{T}S_{M|X,Z}{\hat{\Gamma }}_{0},\\ & {\hat{\sum }}_{Y|M,X,Z}=(1/n)R_{Y|X,Z}^{T}Q_{R_{M|X,Z}\hat{\Gamma }}R_{Y|X,Z}, \end{array} \end{array}$$

where $R_{M|X,Z}=Q_{{𝕎}_{c}}{𝕄}_{c}$ denotes the sample residuals from the regression of M on X and Z, $R_{Y|X,Z}=Q_{{𝕎}_{c}}{𝕐}_{c}$ denotes the sample residuals from the regression of Y on X and Z, $S_{M|X,Z}=R_{M|X,Z}^{T}R_{M|X,Z}/n$, and $S_{XZ}$ denotes the sample covariance between X and Z, and thus ${\hat{\beta }}_{M}=\hat{\Gamma }\hat{\eta }=P_{\hat{\Gamma }(S_{M|X,Z})}S_{M|X,Z}^{-1}S_{(M,Y)|X,Z}=P_{\hat{\Gamma }(S_{M|X,Z})}{\hat{\beta }}_{M,\text{ols}}$ and ${\hat{\sum }}_{M|X,Z}=\hat{\Gamma }\hat{\Omega }{\hat{\Gamma }}^{T}+{\hat{\Gamma }}_{0}{\hat{\Omega }}_{0}{\hat{\Gamma }}_{0}^{T}=P_{\hat{\Gamma }}S_{M|X,Z}P_{\hat{\Gamma }}+Q_{\hat{\Gamma }}S_{M|X,Z}Q_{\hat{\Gamma }},$ where $S_{(Y,M)|X,Z}=R_{Y|X,Z}^{T}R_{M|X,Z}/n$, $P_{\hat{\Gamma }(S_{M|X,Z})}$ denotes the projection matrix onto $\text{span}(\hat{\Gamma })$ with $S_{M|X,Z}$ inner product, and ${\hat{\beta }}_{M,\text{ols}}=S_{M|X,Z}^{-1}S_{(Y,M)|X,Z}$ is the OLS estimator of $\beta_{M}$.

When the number of mediators is larger than the sample size, i.e., p>n for the high‐dimensional mediation analysis, the matrices $S_{M|X,Z}$ and $S_{M|Y,X,Z}$ are singular. Since the objective function in Equation (7) depends on the inverse of $S_{M|X,Z}$, and the inverse of $S_{(M,Y)|X,Z}$ is required in the algorithm to solve (7), we replace $S_{M|X,Z}^{-1}$ and $S_{M|Y,X,Z}^{-1}$ in the objective function and the estimation algorithm with alternative estimators of $\sum_{M|X,Z}^{-1}$ and $\sum_{M|Y,X,Z}^{-1}$. We consider sparse permutation invariant covariance estimators (SPICE), which [19] proposes to address the issue for the high‐dimensional situation. SPICE is a convex, permutation‐invariant, and tuning‐parameter‐free approach for estimating covariance matrices. It ensures positive definiteness and numerical stability in high‐dimensional settings, which is crucial when the number of mediators exceeds the sample size (p>n). Unlike $l_{1}$‐penalized approaches, SPICE does not impose sparsity via an explicit penalty term; instead, SPICE implicitly induces sparsity through its optimization with structured constraints. Furthermore, it possesses desirable theoretical properties, including consistency and asymptotic normality, making it a robust choice for downstream inference in our mediation analysis. Specifically, the SPICE estimators of $S_{M|X,Z}$ and $S_{M|Y,X,Z}$ denoted by $S_{M|X,Z,\text{sp}}$ and $S_{M|Y,X,Z,\text{sp}}$ are used in the objective function as follows:

$$\begin{array}{ll} & \underset{\text{span}(\Gamma )\in {ℒ}_{p\times d}}{\text{argmin}}\left\{\log|S_{Y|X,Z}|+\log|\Gamma^{T}S_{M|X,Z,\text{sp}}^{-1}\Gamma |\right.\\ & \;\left.+\log|\Gamma^{T}S_{M|Y,X,Z,\text{sp}}\Gamma |+\lambda \overset{p-d}{\underset{i=1}{\sum }}w_{i}\|\Gamma_{i}{\|}_{2}\right\}. \end{array}$$

### 3.3 Theoretical Properties

Section 3.3.1 establishes causal identification of the direct and indirect effects, whereas Section 3.3.2 establishes theoretical properties of the estimators of the proposed model. The proofs are provided in the Supporting Information.

#### 3.3.1 Causal Identification

For any exposure level x, we write M(x) for the potential mediator value that would be observed if the exposure were set to X=x, and Y(x,m) for the potential outcome that would be observed if the exposure were set to X=x and the mediator set to M=m. We then define two population‐level causal functionals of interest. The first is the average causal mediation effect (ACME), which captures how much of the effect of changing the exposure is transmitted through changes in the mediator, i.e., $\bar{\delta }(x,x^{\prime })=E[Y(x,M(x))-Y(x,M(x^{\prime }))]\in {\mathbb{R}}^{r}$ for any two exposure settings $x,x^{\prime }\in {\mathbb{R}}^{k}$. The second is the average natural direct effect (ANDE), which captures the portion of the exposure effect that operates through pathways other than the mediator, i.e., $\bar{\zeta }(x,x^{\prime })=E[Y(x,M(x^{\prime }))-Y(x^{\prime },M(x^{\prime }))]\in {\mathbb{R}}^{r}$ for any two exposure settings $x,x^{\prime }\in {\mathbb{R}}^{k}$.

To establish identification for causal interpretation in the multivariate setting, the following assumptions (A1–A4) are required. The assumptions extend the standard identification conditions commonly used in the mediation analysis literature to accommodate vector‐valued exposures, mediators, and outcomes. In addition, the resulting identification expressions are vector‐valued and hold component‐wise.

A1. Stable Unit Treatment Value Assumption (SUTVA): We assume no interference between individuals and no hidden variations of the treatment, ensuring that potential outcomes are well‐defined.
A2. Consistency: The observed outcome for each individual is equal to their potential outcome under the treatment level that they actually received.
A3. Sequential ignorability: Conditional on the observed covariates Z, (i) the exposure assignment is independent of the potential mediators and potential outcomes, and (ii) the potential mediators are independent of the potential outcomes given exposures and covariates, i.e., $\{Y(x^{\prime },m),M(x)\}╨X|Z=z$ and Y(x,m)╨M(x)|{X=x,Z=z}
A4. Positivity: Every subject has a positive probability of receiving each exposure and mediator level for all combinations of the covariates.

Proposition 1 *Under A1–A4, for any pair of exposures* x, $x^{\prime }\in {\mathbb{R}}^{k}$, *both the multivariate ACME* $\bar{\delta }(x,x^{\prime })$ *and the multivariate ANDE* $\bar{\zeta }(x,x^{\prime })$ *are nonparametrically identified*.

Proposition 1 shows that, under conditions that generalize the sequential ignorability framework of [20] to the multivariate setting, these effects are in fact nonparametrically identified.

Next, Proposition 2 specializes the nonparametrically identified effects in Proposition 1 to the EPLSM (and ESPLSM) framework, where the linear structure yields closed‐form expressions for the multivariate ACME and ANDE in terms of regression coefficients.

Proposition 2 *Consider the EPLSM defined in Equation (* 3 *). Under A1–A4, for any pair of exposures* x, $x^{\prime }\in {\mathbb{R}}^{k}$, *the multivariate ACME is identified by* $\bar{\delta }(x,x^{\prime })=\eta^{T}\Gamma^{T}\gamma_{X}^{T}(x-x^{\prime })=\beta_{M}^{T}\gamma_{X}^{T}(x-x^{\prime })$, *the multivariate ANDE* $\bar{\zeta }(x,x^{\prime })=\beta_{X}^{T}(x-x^{\prime })$, *and thus the average total causal effect of changing the exposure from* x *to* $x^{\prime }$ *is* $E(Y|X=x)-E(Y|X=x^{\prime })={\left(\beta_{X}+\gamma_{X}\Gamma \eta \right)}^{T}(x-x^{\prime })$.

Proposition 2 revisits the linear structural equation model framework from a causal perspective. We show that the ACME is identified as the product of the mediator coefficient linking exposure to mediator and the outcome coefficient linking mediator to outcome, and that the ANDE is identified as the direct exposure coefficient in the outcome model. Moreover, in the model without exposure‐mediator interaction, the ACME does not depend on the exposure level at which it is evaluated, and the total effect decomposes additively into the direct and mediated components. In this formulation, independence among exposure or mediator components is not required; instead, their potential dependence is naturally accommodated, as the envelope‐based formulation jointly models the multivariate relationships. Moreover, the cross‐world independence implied by the sequential ignorability assumption is not imposed explicitly but rather interpreted through conditional independence among multivariate potential outcomes and mediators within our modeling framework.

While Proposition 2 establishes identification of the multivariate ACME and ANDE under the EPLSM model, it is important to understand whether such identification is preserved under transformation of the mediator space. In particular, dimension reduction and reparameterization of mediators play a central role in high‐dimensional mediation analysis. We therefore investigate the invariance of causal identification under (i) general invertible linear reparameterizations of the mediators and (ii) envelope‐based projections that remove immaterial variation.

Proposition 3 *Let* $A\in {\mathbb{R}}^{p\times p}$ *be invertible and define the reparameterized mediator* $M^{†}=AM$. *Then, under A1–A4, the identified ACME and ANDE computed with* $M^{†}$ *are identical to those computed with* M. *Moreover, A1–A4 hold with* $M^{†}$ *in place of* M.

Proposition 4 *Let* $U\equiv \Gamma^{T}M$ *and* $V=\Gamma_{0}^{T}M$. *Assume* $Y(x,m)╨V|(U=\Gamma^{T}m,X=x,Z)$, *i.e., conditional on* X,Z, *and the material part of mediators* U, *the immaterial part carries no additional information for* Y. *Then, under A1–A4, the identified ACME and ANDE computed with* U *are identical to those computed with* M.

Proposition 4 establishes that envelope‐based dimension reduction preserves identification of the multivariate ACME and ANDE, even though the envelope projection is not invertible, provided that the immaterial component of the mediators carries no additional information for the outcome conditional on the exposure, covariates, and the material component. In ESPLSM, sparsity is imposed on the coefficients associated with the envelope (material) subspace, without altering the envelope decomposition itself. Consequently, the sparse representation continues to characterize the same material component identified under EPLSM. Under the conditions of Proposition 4, the identification results therefore extend directly to ESPLSM. Moreover, in Proposition 4, the conditional independence statement between the immaterial mediator component and the outcome, given the exposure, covariates, and the material component, is not a strong assumption. Under the joint normality of the mediators and the outcome, it is equivalent to the first assumption of the EPLSM model (i.e., condition (i) in Equation (2)) [12].

#### 3.3.2 Statistical Properties of the Estimators

We present the theory focusing on the low‐dimensional mediator setting in this section, and additional theoretical developments for the high‐dimensional mediator setting are included in the Supporting Information. These results provide a complete characterization of the asymptotic behavior of ESPLSM under a broad range of dimensionality and sparsity conditions. In the following, we use the notations: Let ⊗ denote the Kronecker product, † denote the Moore–Penrose generalized inverse, ∼ denote identical distribution, and $\overset{d}{\to }$ denote convergence in distribution. The vector operator vec stacks the columns of a matrix into a vector, and the vector half operator vech stacks elements from the lower triangle of a symmetric matrix into a vector column‐wise.

We first provide the theoretical result in the Supporting Information, saying that the EPLSM estimator is $\sqrt{n}$‐consistent and asymptotically normal under mild conditions. Note that although the EPLSM estimators are derived using the normal likelihood as an objective function, normality is not required to establish the consistency. The result is established under a fixed p setting, following the classical low‐dimensional asymptotic framework. If we further assume normality, then we can obtain the explicit form of the parameters, as the following proposition reveals.

Proposition 5 *Assume that the conditions in Web Proposition* S1 *hold. We further assume that* ${\left(\epsilon^{T},e^{T}\right)}^{T}$ *is normally distributed. Then, we have*

(9) $$\sqrt{n}\left(\;\mathrm{vec}\left({\hat{\beta }}_{X}\right)-\;\mathrm{vec}\left(\beta_{X}\right)\right)\overset{d}{\to }N\left(\text{0},V_{direct}\right),$$

*where* $V_{direct}=\sum_{Y|M,X,Z}⊗(\gamma_{X}\Gamma \Omega^{-1}\Gamma^{T}\gamma_{X}^{T}+\sum_{X|Z}^{-1})+(\eta^{T}⊗\gamma_{X}\Gamma_{0}){\left(\eta \sum_{Y|M,X,Z}^{-1}\eta^{T}⊗\Omega_{0}+\Omega ⊗\Omega_{0}^{-1}+\Omega^{-1}⊗\Omega_{0}-2I_{d}⊗I_{p-d}\right)}^{-1}(\eta ⊗\Gamma_{0}^{T}\gamma_{X}^{T})$ *and*

(10) $$\sqrt{n}\left(\;\mathrm{vec}\left({\hat{\gamma }}_{X}{\hat{\beta }}_{M}\right)-\;\mathrm{vec}\left(\gamma_{X}\beta_{M}\right)\right)\overset{d}{\to }N\left(\text{0},V_{indirect}\right),$$

*where* $V_{indirect}=\sum_{Y|M,X,Z}⊗\gamma_{X}\Gamma \Omega^{-1}\Gamma^{T}\gamma_{X}^{T}+(\eta^{T}⊗\gamma_{X}\Gamma_{0}){\left(\eta \sum_{Y|M,X,Z}^{-1}\eta^{T}⊗\Omega_{0}+\Omega ⊗\Omega_{0}^{-1}+\Omega^{-1}⊗\Omega_{0}-2I_{d}⊗I_{p-d}\right)}^{-1}(\eta ⊗\Gamma_{0}^{T}\gamma_{X}^{T})+\eta^{T}\Omega \eta ⊗\sum_{X|Z}^{-1}.$

The above theoretical results enable statistical inference on the mediation effects. Specifically, we estimate the indirect effect as ${\hat{\gamma }}_{X}{\hat{\beta }}_{M}$ and the direct effect as ${\hat{\beta }}_{X}$. Under standard regularity conditions, the asymptotic variances of these estimators are derived, allowing for Wald‐type tests and confidence interval construction. Alternatively, a nonparametric bootstrap procedure can be employed to obtain empirical confidence intervals for both effects, which has been shown to provide stable finite‐sample inference.

Now, we prove consistency in the sparsity case when p≤n. Let $\lambda_{𝒜}=\lambda \max\{w_{1},\ldots ,w_{p_{𝒜}-d}\}$ and $\lambda_{ℐ}=\lambda \min\{w_{p_{𝒜}-d+1},\ldots ,w_{p-d}\}$.

Theorem 6 *Assume that ESPLSM holds, and* ${\left(\epsilon^{T},e^{T}\right)}^{T}$ *has finite fourth moments. We further assume that* $\sqrt{n}\lambda_{𝒜}\to 0$. *Then, there exists a local minimizer* $\hat{ℰ}$ *of (* 7 *) such that* $\hat{ℰ}$ *is a* $\sqrt{n}$ *‐consistent estimator of* ℰ *and the estimators* ${\hat{\beta }}_{X}$ *and* ${\hat{\gamma }}_{X}{\hat{\beta }}_{M}$ *for direct and indirect effects are* $\sqrt{n}$ *‐consistent estimators of* $\beta_{X}$ *and* $\gamma_{X}\beta_{M}$, *respectively*.

Theorem 7 *Assume that the conditions in Theorem* 6 *holds, and further assume that* $\sqrt{n}\lambda_{ℐ}\to \infty$. *Then* $\mathrm{Pr}({\hat{\Gamma }}_{i}=\text{0})\to 1$.

Theorem 7 establishes the selection consistency of the ESPLSM estimator. Note that if $\Gamma_{i}=\text{0}$, the outcome‐side regression coefficients corresponding to the ith mediator are zero. Thus, Theorem 7 indicates the ESPLSM estimator selects the sparsity‐selected and excluded mediators correctly with probability tending to 1.

In the Supporting Information, we establish the oracle property of the ESPLSM estimator, showing that it is asymptotically equivalent to the estimator obtained under the true envelope subspace, implying that it correctly selects the sparsity‐excluded predictors with probability tending to 1, and estimates the coefficients of the sparsity‐selected predictors with the same efficiency as if the true model were known. We also establish the convergence rate and selection consistency when p→∞ as n→∞. The selection consistency results say that the ESPLSM estimator correctly identifies sparsity‐selected and excluded mediators with probability tending to 1 when p→∞ as n→∞.

## 4 Simulation Study

In this section, we evaluate the performance of the proposed model, referred to as ESPLSM, and compare it to existing models in terms of both estimation accuracy and variable selection effectiveness. We consider a high‐dimensional linear mediation model (HIMA) using the method of [7], HIMA2 [8], Ordinary least squares (OLS), and Sparse partial least squares (SPLS) as comparator methods for evaluation. OLS does not perform variable selection, and we do not include this for variable selection performance. SPLS does not provide full coefficient estimates and thus is not directly comparable in bias‐based metrics. HIMA extends the partial penalization approach to mediation models and is implemented through HIMA R package [7, 18]. This extension enables the simultaneous estimation and testing of both direct and indirect effects of an exposure. HIMA2 extends the HIMA method [21] to high‐dimensional mediation models and provides mediator‐level inference while controlling false discoveries. We implement HIMA2 using the publicly available R code from the author's repository. The repository output reports mediator‐specific estimates of the exposure‐mediator effects and the mediator‐outcome effects, along with the corresponding product form indirect effects. Because the implementation does not explicitly report the exposure coefficient in the outcome model (i.e., the direct‐effect parameter $\beta_{X}$), the simulation evaluation for HIMA2 focuses on the estimation accuracy of mediator‐outcome effects and indirect effects, as well as mediator selection performance.

The data are generated from the ESPLSM model, with p=11, k=1, q=2, d=1, r=1, and $\sum_{Y|M,X,Z}=3I_{r}$. The matrix $\left(\Gamma ,\Gamma_{0}\right)$ is obtained by normalizing a p×p matrix of independent normal (0,1) variates. We set the last seven rows in Γ to be zero and normalize the first four rows such that $\Gamma^{T}\Gamma =I_{d}$. Then the first four mediator variables are sparsity‐selected and the last seven are sparsity‐excluded Also, η is a d×r matrix with each element being independent normal $\left(3,0.25^{2}\right)$ variates, $\beta_{X}=1.51_{r}^{T}$ and $\beta_{Z}={\left(-51_{r},21_{r}\right)}^{T}$, where $1_{r}\in {\mathbb{R}}^{r}$ denotes a vector of 1. Let $A\in {\mathbb{R}}^{d\times d}$ be independent normal $\left(1,1^{2}\right)$ variates and $B\in {\mathbb{R}}^{\left(p-d)\times (p-d\right)}$ be a matrix of independent uniform (0.4,0.7) variates, $\Omega =AA^{T}$ and $\Omega_{0}=BB^{T}$. We have ‖Ω‖=1.18 and $\|\Omega_{0}\|=29.34$, where ‖·‖ denotes the spectral norm. To generate the continuous predictors M, we let $\gamma_{X}=1_{p}^{T}$, $\gamma_{Z}={\left(-0.11_{p},2.51_{p}\right)}^{T}$. We also let e follow a multivariate normal distribution with a zero mean vector and variance matrix $\sum_{M|X,Z}=\Gamma \Omega \Gamma^{T}+\Gamma_{0}\Omega_{0}\Gamma_{0}^{T}$. The error ϵ is generated from a multivariate normal distribution with mean **0** and covariance $\sum_{Y|M,X,Z}$. The exposure X is generated from Bernoulli variates with response probability 0.5, and the confounders $Z=(Z_{1},Z_{2})$ are independently generated from Bernoulli variates with response probability 0.1 and normal $\left(0,0.1^{2}\right)$ variates, respectively.

We vary the sample size (n=100,300,500,1000) and perform 1000 replications for each sample size to calculate the square root of the mean square error (MSE) of the estimators based on the Frobenius norm, denoted by $\|\cdot {\|}_{F}$. The results are summarized in Table 1. ESPLSM has the smallest MSE among all the methods across all parameters and sample sizes. For the mediator effect $\beta_{M}$, both HIMA and HIMA2 substantially outperform OLS. However, HIMA2 performs worse than HIMA in small samples, reflecting a finite‐sample efficiency loss due to its additional regularization. As the sample size increases, HIMA2 becomes increasingly competitive and eventually outperforms HIMA in large samples (e.g., n=1000). For $\beta_{X}$ and $\gamma_{X}\beta_{M}$, HIMA‐type methods exhibit slower convergence compared to ESPLSM.

**TABLE 1:** Results of average bias (standard deviation/${\left(\text{number of replications}\right)}^{1/2}$) of the estimate based on 1000 replications.

Methods	n=100	n=300	n=500	n=1000
	$\|{\hat{\beta }}_{X}-\beta_{X}{\|}_{F}$			
ESPLSM	0.745 (0.017)	0.610 (0.011)	0.560 (0.009)	0.518 (0.006)
HIMA	1.386 (0.004)	1.379 (0.003)	1.371 (0.002)	1.366 (0.001)
OLS	2.591 (0.065)	1.389 (0.033)	1.046 (0.025)	0.718 (0.017)
	$\|{\hat{\beta }}_{M}-\beta_{M}{\|}_{F}$			
ESPLSM	0.469 (0.005)	0.376 (0.003)	0.341 (0.002)	0.309 (0.001)
HIMA2	5.519 (0.065)	3.178 (0.040)	2.506 (0.032)	1.788 (0.022)
HIMA	3.903 (0.035)	2.883 (0.017)	2.526 (0.013)	2.201 (0.008)
OLS	13.99 (0.252)	7.401 (0.129)	5.660 (0.095)	3.914 (0.064)
	$\|{\hat{\gamma }}_{X}{\hat{\beta }}_{M}-\gamma_{X}\beta_{M}{\|}_{F}$			
ESPLSM	0.789 (0.017)	0.622 (0.011)	0.553 (0.009)	0.526 (0.006)
HIMA2	2.936 (0.082)	1.728 (0.046)	1.381 (0.035)	0.990 (0.025)
HIMA	1.460 (0.005)	1.393 (0.003)	1.294 (0.002)	1.229 (0.001)
OLS	2.589 (0.065)	1.381 (0.033)	1.041 (0.025)	0.712 (0.017)

As a sensitivity analysis in the mediation setting to evaluate the accuracy of the estimated effects, we consider two scenarios: (1) mediators associated with Y but independent of X (i.e., $\gamma_{X}=\text{0}$ but $\beta_{M}\neq \text{0}$) and (2) mediators associated with X but independent of Y (i.e., $\gamma_{X}\neq \text{0}$ but η=0, leading to $\beta_{M}=\text{0}$). Of note, when there are no mediators (i.e., $\gamma_{X}=\text{0}$ and $\beta_{M}=\text{0}$), the ESPLSM reduces to the standard linear model, yielding estimates identical to those from OLS. Because this case does not demonstrate the advantage of the proposed framework, it is excluded from the analysis. The results are summarized in Tables 2 and 3. When mediators are associated with Y but independent of X (i.e., in Table 2), ESPLSM and OLS yield nearly identical estimation accuracy for $\beta_{X}$, while ESPLSM substantially outperforms competing methods in estimating $\beta_{M}$. HIMA provides the most accurate estimation of the indirect effect, while HIMA2 exhibits substantially larger estimation errors. When mediators are associated with X but independent of Y (i.e., in Table 3), a similar pattern is observed: HIMA continues to perform well for indirect‐effect estimation, while HIMA2 performs poorly. Across all parameters, however, ESPLSM maintains stable and competitive performance, confirming its robustness for simultaneous estimation of direct, mediator, and indirect effects. Compared with Table 1, these sensitivity analyses reinforce that ESPLSM provides reliable overall estimation, whereas HIMA is preferable when the primary goal is indirect‐effect estimation under misspecification.

**TABLE 2:** Results of average bias (standard deviation/${\left(\text{number of replications}\right)}^{1/2}$) of the estimate based on 1000 replications when mediators associated with Y but independent of X.

Methods	n=100	n=300	n=500	n=1000
	$\|{\hat{\beta }}_{X}-\beta_{X}{\|}_{F}$			
ESPLSM	0.491 (0.012)	0.290 (0.007)	0.221 (0.005)	0.156 (0.004)
HIMA	1.349 (0.002)	1.345 (0.001)	1.342 (0.001)	1.335 (0.001)
OLS	0.488 (0.011)	0.282 (0.007)	0.218 (0.005)	0.152 (0.004)
	$\|{\hat{\beta }}_{M}-\beta_{M}{\|}_{F}$			
ESPLSM	0.469 (0.005)	0.376 (0.003)	0.341 (0.002)	0.309 (0.001)
HIMA2	7.046 (0.097)	3.890 (0.051)	3.036 (0.039)	2.080 (0.026)
HIMA	3.825 (0.032)	2.898 (0.016)	2.557 (0.013)	2.243 (0.008)
OLS	13.99 (0.252)	7.401 (0.129)	5.660 (0.096)	3.914 (0.064)
	$\|{\hat{\gamma }}_{X}{\hat{\beta }}_{M}-\gamma_{X}\beta_{M}{\|}_{F}$			
ESPLSM	0.593 (0.013)	0.292 (0.007)	0.227 (0.006)	0.154 (0.004)
HIMA2	0.944 (0.026)	0.450 (0.011)	0.346 (0.008)	0.250 (0.006)
HIMA	0.063 (0.001)	0.031 (0.001)	0.025 (0.001)	0.017 (0.0004)
OLS	0.625 (0.015)	0.305 (0.008)	0.239 (0.006)	0.160 (0.004)

**TABLE 3:** Results of average bias (standard deviation/${\left(\text{number of replications}\right)}^{1/2}$) of the estimate based on 1000 replications when mediators associated with X but independent of Y.

Methods	n=100	n=300	n=500	n=1000
	$\|{\hat{\beta }}_{X}-\beta_{X}{\|}_{F}$			
ESPLSM	0.485 (0.011)	0.291 (0.007)	0.225 (0.005)	0.159 (0.004)
HIMA	1.281 (0.004)	1.281 (0.002)	1.281 (0.002)	1.279 (0.001)
OLS	2.597 (0.065)	1.390 (0.033)	1.046 (0.025)	0.715 (0.017)
	$\|{\hat{\beta }}_{M}-\beta_{M}{\|}_{F}$			
ESPLSM	0.455 (0.025)	0.292 (0.136)	0.190 (0.010)	0.177 (0.008)
HIMA2	6.752 (0.089)	3.799 (0.048)	3.000 (0.039)	2.094 (0.027)
HIMA	3.524 (0.053)	1.908 (0.026)	1.396 (0.020)	1.004 (0.014)
OLS	13.98 (0.251)	7.403 (0.129)	5.661 (0.095)	3.914 (0.064)
	$\|{\hat{\gamma }}_{X}{\hat{\beta }}_{M}-\gamma_{X}\beta_{M}{\|}_{F}$			
ESPLSM	0.150 (0.005)	0.082 (0.003)	0.064 (0.002)	0.047 (0.001)
HIMA2	3.854 (0.097)	2.111 (0.053)	1.660 (0.042)	1.189 (0.030)
HIMA	0.059 (0.002)	0.030 (0.001)	0.022 (0.001)	0.015 (0.0004)
OLS	2.562 (0.063)	1.350 (0.032)	1.025 (0.024)	0.706 (0.017)

**FIGURE 2:**
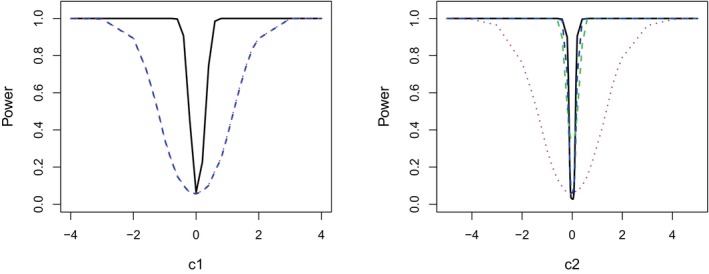
Power for the direct effect with $c_{2}=0.5$ (left panel) and power for the indirect effect with $c_{1}=0.5$ (right panel) when n=1000. The black solid line marks for ESPLSM, the green dashed line marks for HIMA2, the blue dashed line marks for HIMA, and the magenta dotted line marks for OLS.

We conduct hypothesis tests for both the direct and indirect effects at a significance level of 0.05, using the same simulation setting as in Table 1. For the direct effect, the hypotheses are $H_{0(d)}:\beta_{X}=0$ vs. $H_{a(d)}:\beta_{X}\neq 0$, and for the indirect effect, the hypotheses are $H_{0(i)}:\gamma_{X}\beta_{M}=0$ vs. $H_{a(i)}:\gamma_{X}\beta_{M}\neq 0$. To conduct the tests, we modify the generated regression coefficients $\beta_{X}$ and $\beta_{M}$ by introducing scaling factors. Specifically, we replace $\beta_{X}$ with $c_{1}\beta_{X}$ and $\beta_{M}$ with $c_{2}\beta_{M}$ where $c_{1}=0,\pm 0.2,\pm 0.4$, ±0.6,±0.8,±1, ±1.2,±1.4,±1.6, ±1.8,±2,±3, ±4 and $c_{2}=0,\pm 0.03,\pm 0.05,\pm 0.07$, ±0.1,±0.15,±0.2,±0.4, ±0.6,±0.8,±1,±1.2, ±1.4,±1.6,±1.8, ±2,±3,±4,±5 are chosen to reflect varying effect sizes and evaluate test power. We construct the Wald test statistic for ESPLSM and OLS using the estimated covariance matrix derived from the asymptotic normality of their estimators. HIMA2 tests for indirect effects using a three‐step procedure designed for high‐dimensional data. It first narrows down the pool of potential mediators via sure independence screening, selecting candidates with the largest joint indirect effect estimates. Next, it applies a de‐biased Lasso procedure to the screened mediators to obtain accurate *p*‐values for the mediator‐outcome paths, effectively handling the high dimensionality. Finally, the method performs a joint significance test using a mixture distribution approach, which identifies significant mediators by controlling the false discovery rate and with greater power than methods assuming a uniform distribution. For HIMA, as outlined in Reference [7], the modified F‐type lack‐of‐fit test proposed by Guo et al. [18] is applied to test the direct effect, while the Wald test statistic is used to assess the indirect effect. The results, based on 1000 replications, are displayed in Figure 2. We observe that power increases in the magnitude of $c_{1}$ or $c_{2}$, indicating stronger effects. Among the methods compared, ESPLSM demonstrates the highest power to detect signals of the direct and indirect effects, outperforming HIMA2, HIMA, and OLS. HIMA and HIMA2, in turn, are more powerful than OLS to detect the indirect effect. All methods except for HIMA2 maintain the overall Type I error rate at the nominal level when $c_{1}$ or $c_{2}$ is zero, indicating that the direct or indirect effect is absent.

Moreover, we investigate the performance of the method when there are multiple responses, i.e., r>1, or when there are multiple exposures, i.e., k>1. The results are summarized in Web Appendix C. Web Table S1 shows that ESPLSM demonstrates superior performance, maintaining low bias and variability across values of r=10,50, whereas OLS suffers from rapidly increasing bias as r grows. Since HIMA and HIMA2 work for univariate responses only and are not applicable for evaluating multiple responses, we do not include the results in Web Table S1. Web Table S2 shows that ESPLSM demonstrates the best overall performance, yielding the smallest estimation bias under a setting with two correlated exposures, varying the exposure correlation (ρ=0,0.3,0.5,0.75). HIMA2 shows clear sample‐size dependence: While its bias is relatively large at n=100, it becomes substantially more stable at n=1000, indicating improved reliability in large‐sample settings. In contrast, HIMA exhibits persistent bias across all scenarios, particularly for mediator and indirect effects, and shows limited improvement with increasing sample size. OLS performs poorly throughout.

To evaluate the robustness of the proposed method when the underlying assumptions are violated, we conduct additional simulations under various forms of model misspecification. First, to evaluate robustness to non‐normal errors, we consider error distributions including the t distribution with degrees of freedom 3, chi‐squared distribution with degrees of freedom 4, and uniform distribution defined on the unit interval. Second, we perform simulations introducing unobserved confounding that affects both mediators and outcomes. Specifically, we add a linear confounder‐mediator effect $\gamma_{U}$ and a confounder‐outcome effect $\beta_{U}$. The results are summarized in Web Tables S3 and S4. They show that ESPLSM maintains competitive performance in estimating direct and indirect effects, although estimation bias mildly increases under more severe misspecification.

We examine the variable selection performance of the ESPLSM with three measures: True positive rate (TPR or called recall or sensitivity), true negative rate (TNR or specificity), and Precision (or called positive predictive value). The averages of TPR, TNR, and Precision, computed over 1000 replications, are summarized in Web Appendix C. We first vary the sample size (n=100,300,500,1000) and variance ($\sum_{Y|M,X,Z}=3I_{r},I_{r},0.5I_{r},0.1I_{r}$), with results reported in Web Table S5. We further examine scenarios in which the proportion of sparsity‐selected mediators varies across 45.5%, 54.6%, 63.6%, 72.7%, and 81.8%, corresponding to 5, 6, 7, 8, and 9 sparsity‐selected mediators out of p=11 in total. The results are reported in Web Table S6. In addition, we investigate settings with weak indirect effects by setting $c_{2}=0.05,0.07,0.1,0.2$, corresponding to the cases shown in the right panel of Figure 2. The results are reported in Web Table S7. Taken together, Web Tables S5–S7 demonstrate that ESPLSM balances variable selection performance, achieving high TNRs and precision while maintaining competitive TPRs across all scenarios. In contrast, SPLS decreases TNR as the proportion of active mediators increases and decreases TPR as the indirect effects become weak. HIMA, while achieving high TPRs, consistently selects nearly all mediators under the considered settings, resulting in TNRs close to zero and limiting its utility for sparse mediator identification. HIMA2 improves false discovery control relative to HIMA, but still shows greater variability in selection performance and lower precision compared with ESPLSM across many scenarios.

We next investigate the performance of ESPLSM when n=100<p. We vary the number of mediators (p=150,200,300,500). In this setting, where n<p, it is not possible to compute the OLS estimators; however, we include the results from SIMPLS as a reference. We summarize the mean prediction error, calculated using five‐fold cross‐validation, and the average TPR, TNR, and precision from 100 replications in Web Appendix C. The results show that the ESPLSM estimator achieves the lowest mean prediction error, regardless of the value of p. The ESPLSM estimator demonstrates superior selection performance compared to SPLS.

We further align the simulation setup with the data application presented in Section 5 by matching the sample size and the dimensionality of the variables (i.e., n=612,p=296,k=1,q=0,d=2,r=14). Since 74 RNA expressions are identified as sparsity‐selected mediators by ESPLSM in the real data analysis, we set the first 74 rows of Γ to be nonzero in the simulation. Following the main simulation setting, the data are generated from the ESPLSM model without confounding variables Z. Under this configuration, we obtain the average estimation bias (with standard deviation in parentheses) based on 1000 replications: $\left\|{\hat{\beta }}_{X}-\beta_{X}{\|}_{F}=1.654(0.069\right)$, $\left\|{\hat{\beta }}_{M}-\beta_{M}{\|}_{F}=2.160(0.123\right)$, and $\left\|{\hat{\gamma }}_{X}{\hat{\beta }}_{M}-\gamma_{X}\beta_{M}{\|}_{F}=3.177(0.234\right)$. The corresponding variable selection performance yields a true positive rate of 0.927, a true negative rate of 1.000, and a precision of 1.000. In addition, the empirical power is 0.97 and 1.00 for testing the direct and indirect effects, respectively.

## 5 Data Application

We applied the envelope‐based sparse partial least squares for mediation analysis (ESPLSM) to the cancer cell line datasets as described in Section 1. We used the preprocessed data by [3] for the Epidermal Growth Factor Receptor (EGFR) pathway. The dataset includes DNA mutation status, RNA expression levels, and drug response data measured by the normalized IC‐50 Z‐scores for 612 cell lines. EGFR mutation status is represented as a binary variable. As suggested in Reference [3], using a knowledge discovery approach that integrates biological insights from literature reviews to identify genes associated with the mutation types of interest, we have continuous RNA expression levels for 296 genes. The study considered 14 targeted treatments for the EGFR pathway, such as Erlotinib, Lapatinib, Pelitinib, Tivozanib, CI.1033, PF.00299804, AST.1306, Gefitinib, Afatinib, Cetuximab, AZD8931, Sapitinib, AZD3759, and Osimertinib, and thus we have r=14 responses considered as continuous variables. Our problem of interest is to investigate whether a DNA mutation induces molecular changes in cancer cells that, through alterations in RNA expression, ultimately affect therapeutic response, and to determine whether RNA expression significantly mediates the effect of a DNA mutation on clinical outcomes.

To follow the causal structure assumed in the proposed model, the DNA mutation status serves as the exposure (X), the RNA expression levels act as mediators (M), and the drug response represents the outcome (Y). Under this framework, the total effect of DNA mutation on drug response can be decomposed into (i) a direct effect, capturing the mutation's remaining influence after adjusting for RNA expression, and (ii) an indirect effect, quantifying how the mutation affects response through downstream molecular changes in RNA expression. Although baseline covariates were not available in this dataset, the EGFR mutation status is predetermined, and both RNA expression and drug response were measured without interference. Therefore, the cause assumptions A1–A4 described in Section 3.1 are considered reasonable in this application.

To fit the proposed model of the Z‐scores on a DNA mutation and RNA expressions, we standardized all variables and used the cross‐validation method to choose the dimension of the ESPLSM. It suggests d=2. The analysis selects 74 RNA expressions whose list is provided in the Supporting Information. We also fitted a standard linear regression model using OLS as a benchmark for comparison. However, OLS does not perform variable selection—it includes all 296 RNA expressions in the analysis. Moreover, because our dataset comprises multiple responses and lacks confounders, the HIMA method is not directly applicable in this context.

Table 4 summarizes the estimated direct, indirect, and total effects of a DNA mutation on the Z‐scores for various targeted drugs for the EGFR pathway with bootstrap‐based *p*‐values.

**TABLE 4:** Results of estimating the effects of a DNA mutation on the Z‐scores.

Targeted drug	Direct effect		Indirect effect		Total effect	
	ESPLSM	OLS	ESPLSM	OLS	ESPLSM	OLS
Erlotinib	−0.053 (0.367)	−0.066 (0.182)	−**0.007 (0.008)**	0.007 (0.853)	−0.060 (0.302)	−0.060 (0.161)
Lapatinib	−0.051 (0.251)	−0.013 (0.707)	−0.0007 (0.843)	−0.038 (0.211)	−0.051 (0.249)	−0.051 (0.131)
Pelitinib	−**0.093 (0.073)**	−**0.080 (0.069)**	0.0001 (0.961)	−0.013 (0.672)	−**0.093 (0.076)**	−**0.093 (0.030)**
Tivozanib	−0.031 (0.531)	−0.044 (0.360)	**0.008 (0.002)**	0.021 (0.447)	−0.022 (0.649)	−0.022 (0.609)
CI.1033	−0.015 (0.779)	0.012 (0.799)	**0.005 (0.046)**	−0.022 (0.537)	−0.010 (0.857)	−0.010 (0.811)
PF.00299804	−0.056 (0.323)	−0.034 (0.496)	**0.008 (0.003)**	−0.014 (0.623)	−0.048 (0.392)	−0.048 (0.287)
AST.1306	−0.005 (0.926)	−0.008 (0.864)	**0.005 (0.077)**	0.007 (0.833)	−0.0005 (0.993)	−0.0005 (0.991)
Gefitinib	−0.083 (0.108)	−**0.076 (0.057)**	−**0.006 (0.057)**	−0.013 (0.619)	−**0.089 (0.081)**	−**0.089 (0.024)**
Afatinib	−0.067 (0.214)	−0.042 (0.406)	−**0.010 (0.0001)**	−0.035 (0.222)	−0.077 (0.149)	−**0.077 (0.088)**
Cetuximab	−0.032 (0.523)	−0.016 (0.680)	−**0.013 (0.00002)**	−0.029 (0.323)	−0.045 (0.377)	−0.045 (0.272)
AZD8931	−0.064 (0.208)	−0.066 (0.126)	−**0.004 (0.078)**	−0.002 (0.946)	−0.069 (0.182)	−**0.069 (0.097)**
Sapitinib	−**0.094 (0.056)**	−0.068 (0.180)	−**0.006 (0.025)**	−0.032 (0.327)	−**0.100 (0.039)**	−**0.100 (0.019)**
AZD3759	−**0.098 (0.071)**	−0.059 (0.171)	−**0.008 (0.00006)**	−0.047 (0.134)	−**0.106 (0.051)**	−**0.106 (0.010)**
Osimertinib	−**0.113 (0.028)**	−**0.096 (0.016)**	−0.00005 (0.986)	−0.018 (0.624)	−**0.113 (0.025)**	−**0.113 (0.003)**

*Note:* The value in parentheses represents the *p*‐value computed using the bootstrap method. Values that are bolded indicate statistical significance at the 10% level.

As we use IC‐50 Z‐scores as the dependent variable, lower values indicate a stronger drug response. In this context, a negative regression coefficient implies that as the independent variable increases, the IC‐50 (or viability) decreases—thus signifying an improved response. Under the ESPLSM, drugs such as Erlotinib, Gefitinib, Afatinib, and Cetuximab exhibit statistically significant indirect effects, indicating that RNA expression alterations mediate the impact of DNA mutations. However, their overall total effects differ: Gefitinib shows a significant total effect, suggesting that its mediated impact is sufficiently strong, whereas Erlotinib, Afatinib, and Cetuximab do not exhibit significant total effects despite their significant indirect effects. In contrast, the non‐sparse OLS model is inefficient in its estimation and fails to capture these subtle direct or indirect effects at the 10% significance level. Although Afatinib and AZD8931 display significant total effects under OLS, their individual direct and indirect effects are not statistically significant. This discrepancy underscores that more effective estimation methods, like ESPLSM, are better suited for detecting mediated effects. Under both ESPLSM and OLS, both Osimertinib and Pelitinib exhibit significant direct effects without an accompanying indirect effect, indicating that their efficacy may be more directly associated with the mutation itself. Additionally, drugs like Sapitinib and AZD3759 show both significant direct and indirect effects under ESPLSM, implying that their clinical effectiveness might result from a combination of direct molecular impact and RNA expression‐mediated mechanisms. For some drugs (e.g., Tivozanib, CI.1033, PF.00299804, and AST.1306), Table 4 reveals statistically significant positive indirect effects under ESPLSM, suggesting that the mutation could, via mediated pathways such as RNA expression changes, increase the Z‐scores. However, these positive indirect effects are relatively small and insufficient to counterbalance the dominant negative direct effects. As a result, the overall total effect remains negative and is not statistically significant, indicating that the mutation's negative impact on drug efficacy prevails. These findings are consistent with clinical observations where certain drugs demonstrate differential efficacy based on the underlying molecular pathways, highlighting the importance of considering both direct and mediated effects when evaluating treatment responses. For example, Gefitinib was approved by the FDA for the treatment of non‐small cell lung cancer (NSCLC) based on its efficacy in patients with EGFR mutations, with key references [22, 23]. The pivotal FLAURA trial [24] demonstrated that Osimertinib significantly outperformed first‐generation EGFR inhibitors, such as Gefitinib and Erlotinib, in terms of progression‐free survival in patients with untreated advanced EGFR‐mutated NSCLC. The results suggest that Osimertinib provides more direct and effective inhibition of EGFR mutations, potentially due to its selective targeting of both the EGFR sensitizing mutations and the T790M resistance mutation. This evidence aligns with the observation that taking into account both direct effects (via mutation‐specific inhibition) and mediated effects (through downstream molecular changes) is essential in evaluating the therapeutic responses to targeted treatments.

## 6 Discussion

We propose Envelope‐based Sparse Partial Least Squares for Mediation Analysis (ESPLSM), addressing the limitations of existing methods in handling large mediator sets. Rather than replacing existing penalized regression‐based mediation methods, ESPLSM serves as a complementary framework by incorporating envelope‐based dimension reduction to explicitly account for mediator dependence and filter out immaterial variation, thereby improving estimation efficiency, while retaining interpretability through sparsity. We establish the causal identification of the direct and indirect effects under the ESPLSM model. We also establish theoretical results for ESPLSM under both fixed‐ and diverging‐dimension asymptotic regimes, ensuring consistent estimation and valid inference for both direct and indirect causal effects. The availability of asymptotically valid Wald‐type tests allows practitioners to conduct formal hypothesis testing, which provides a theoretical bridge between classical envelope estimation and high‐dimensional causal mediation analysis. Our findings show that ESPLSM consistently outperforms existing sparse mediation approaches by providing more efficient and stable estimation of causal pathways, leading to more reliable and interpretable inference for direct and indirect effects, even in high‐dimensional mediator settings.

In the real data application, ESPLSM identifies biologically meaningful RNA mediators linking EGFR mutations to drug response, providing clear evidence of its ability to reveal mechanistic pathways consistent with the assumed causal structure. Given that previous analyses on the same dataset (e.g., [3]; [25]) adopted linear frameworks and reported no substantial evidence of nonlinear or interaction effects, we follow the same modeling assumption for consistency and interpretability. In our cancer cell line analysis, ESPLSM identifies 74 significant RNA mediators linking EGFR mutations to drug responses, offering biological insights. Gefitinib and Erlotinib act through RNA‐mediated pathways, while Osimertinib and Pelitinib primarily inhibit mutations directly. Sapitinib and AZD3759 exhibit both direct and indirect effects. ESPLSM also reveals the inefficiency of nonsparse models like OLS, which fail to capture subtle mediation effects. By integrating a sparse envelope structure, ESPLSM improves mediator selection, interpretability, and estimation efficiency.

Despite its efficiency and interpretability, the current formulation also has several limitations. First, the proposed ESPLSM framework primarily imposes sparsity on the outcome‐side coefficients through the envelope structure, while sparsity on the exposure‐mediator pathway is not explicitly modeled. As a result, mediators that are inactive on the exposure side may still be partially represented in the envelope subspace, although their contribution to the indirect effect remains negligible. Analogous to that of the mediators cannot be formally defined for X due to the absence of a stochastic covariance matrix, exposure selection may still be achieved by introducing sparsity in the exposure effect $\gamma_{X}$ [9], which would enable the identification of influential exposure variables without violating the design assumption. Extending the framework to jointly regularize both $\gamma_{X}$ and $\beta_{M}$ within the envelope formulation would further enhance the interpretability of the identified mediation pathways. In addition, the current model assumes a linear relationship among variables. While this assumption offers tractability and a transparent, parameter‐based representation of direct and indirect effects, real biological systems may involve nonlinear or interaction effects. In such settings, obtaining interpretable estimates of average causal mediation effects, while maintaining causal interpretability, may require additional assumptions or sensitivity analyses. Developing a generalized envelope‐based mediation framework that accommodates nonlinear or interaction structures while preserving interpretable causal pathways is an important direction for future research. Moreover, the current formulation assumes a homogeneous covariance structure for the predictors, where the covariance matrix $\sum_{M}$ does not depend on M. This assumption simplifies estimation and ensures that the immaterial component has no linear effect on the response on the mean scale. However, in practice, heteroscedasticity may arise when the variability of the predictor changes with its levels or covariates. Extending the proposed framework to accommodate heteroscedastic covariance structure—such as through the envelope model proposed by [26]—would allow more flexible modeling of real‐world data.

## Funding

This work was supported by National Research Foundation of Korea (NRF), South Korea grant funded by the Korea government (MSIT) (RS‐2025‐02216235).

## Conflicts of Interest

The authors declare no conflicts of interest.

## Supporting information




**Data S1:** sim70464‐sup‐0001‐Supinfo.

## Data Availability

The data that support the findings of this study are available from the corresponding author upon reasonable request.
