# Supplementary material for: ESPLSM: An Efficient and Interpretable Mediation Analysis Framework Using Sparse Envelopes
Source: Stat Med. 2026 Mar 18;45(6-7):e70464. doi: 10.1002/sim.70464 (PMC12999549; doi:10.1002/sim.70464)
Supplement: Supplementary file 1 — Data S1: sim70464‐sup‐0001‐Supinfo. [file SIM-45-0-s001.pdf]

Supplementary Material for "ESPLSM: An Efficient and Interpretable Mediation Analysis Framework Using Sparse Envelopes"

Yeonhee Park[1,*] and Zhihua Su[2]

[1]Department of Statistics, Sungkyunkwan University, Seoul, South Korea

[2]nVerses Capital, LLC, Wellington, FL, USA

[*]email: yeonheepark@skku.edu

Web Appendix A: Further theoretical results

In preparation, we introduce notations. Let $\otimes$ denote the Kronecker product, $\dagger$ denote the Moore-Penrose generalized inverse, $\sim$ denote identical distribution, and $\xrightarrow{d}$ denote convergence in distribution. The vector operator vec stacks the columns of a matrix to a vector, and the vector half operator vech stacks elements from the lower triangle of a symmetric matrix into a vector column-wise. For example, if $\mathbf{A} \in \mathbb{R}^{a\times b}$ is a matrix, then $\operatorname{vec}(\mathbf{A}) \in \mathbb{R}^{ab}$ is a column vector. If $\mathbf{A} \in \mathbb{R}^{a\times a}$ is a symmetric matrix, $\operatorname{vech}(\mathbf{A}) \in \mathbb{R}^{a(a+1)/2}$. For symmetric matrices, say $\mathbf{A} \in \mathbb{R}^{a\times a}$, $\mathbf{E}_a \in \mathbb{R}^{a^2\times a(a+1)/2}$ and $\mathbf{C}_a \in \mathbb{R}^{a(a+1)/2\times a^2}$ are expansion and contraction operators that connect vec and vech operator, i.e., $\operatorname{vec}(\mathbf{A}) = \mathbf{E}_a \operatorname{vech}(\mathbf{A})$

and $\mathrm{vech}(\mathbf{A}) = \mathbf{C}_a \,\mathrm{vec}(\mathbf{A})$. If $\mathbf{A}$ is a positive semi-definite matrix, then we write $\mathbf{A} \succeq 0$.

Under the EPLSM model (3), the parameter vector is $\mathbf{h} = (\boldsymbol{\mu}_{\mathbf{Y}}^T, \boldsymbol{\mu}_{\mathbf{M}}^T, \boldsymbol{\mu}_{\mathbf{X}}^T, \boldsymbol{\mu}_{\mathbf{Z}}^T,$ $\mathrm{vec}^T(\boldsymbol{\beta}_{\mathbf{X}}), \mathrm{vec}^T(\boldsymbol{\beta}_{\mathbf{Z}}), \mathrm{vec}^T(\boldsymbol{\beta}_{\mathbf{M}}), \mathrm{vec}^T(\boldsymbol{\gamma}_{\mathbf{X}}), \mathrm{vec}^T(\boldsymbol{\gamma}_{\mathbf{Z}}), \mathrm{vech}^T(\boldsymbol{\Sigma}_{\mathbf{Y}|\mathbf{M},\mathbf{X},\mathbf{Z}}), \mathrm{vech}^T(\boldsymbol{\Sigma}_{\mathbf{M}|\mathbf{X},\mathbf{Z}}))^T$. The constituent parameter vector is $\boldsymbol{\phi} = (\boldsymbol{\mu}_{\mathbf{Y}}^T, \boldsymbol{\mu}_{\mathbf{M}}^T, \boldsymbol{\mu}_{\mathbf{X}}^T, \boldsymbol{\mu}_{\mathbf{Z}}^T, \mathrm{vec}^T(\boldsymbol{\beta}_{\mathbf{X}}), \mathrm{vec}^T(\boldsymbol{\beta}_{\mathbf{Z}}),$ $\mathrm{vec}^T(\boldsymbol{\Gamma}), \mathrm{vec}^T(\boldsymbol{\eta}), \mathrm{vec}^T(\boldsymbol{\gamma}_{\mathbf{X}}), \mathrm{vec}^T(\boldsymbol{\gamma}_{\mathbf{Z}}), \mathrm{vech}^T(\boldsymbol{\Sigma}_{\mathbf{Y}|\mathbf{M},\mathbf{X},\mathbf{Z}}), \mathrm{vech}^T(\boldsymbol{\Omega}), \mathrm{vech}^T(\boldsymbol{\Omega}_0))^T$. Then, $\mathbf{h}$ is a function of $\boldsymbol{\phi}$. Web Proposition 1 indicates that the EPLSM estimator is $\sqrt{n}$-consistent and asymptotically normal under mild conditions. Note that although the EPLSM estimators are derived using the normal likelihood as an objective function, normality is not required in the following proposition.

Web Proposition 1 *Suppose that the EPLSM model* (3) *holds,* $(\boldsymbol{\epsilon}^T, \mathbf{e}^T)^T$ *has finite fourth moments and is independently and identically distributed in the sample. Let* $\widehat{\mathbf{h}}$ *denote the EPLSM estimator of* $\mathbf{h}$*, then we have* $\sqrt{n}(\widehat{\mathbf{h}} - \mathbf{h}) \xrightarrow{d} N(\mathbf{0}, \mathbf{U}), \mathbf{U} = \boldsymbol{\Delta}(\boldsymbol{\Delta}^T \mathbf{V} \boldsymbol{\Delta})^{\dagger} \boldsymbol{\Delta}^T$*, where* $\boldsymbol{\Delta} = \partial \mathbf{h} / \partial \boldsymbol{\phi}^T$ *is the gradient matrix, and* $\mathbf{V}$ *is the Fisher information matrix from the standard estimation (performed by OLS).*

Web Proposition 1 is established under a fixed p setting, following the classical low-dimensional asymptotic framework. From Web Proposition 1, we notice the

relationship between $\mathbf{V}^{-1}$, the asymptotic covariance matrix of the standard estimator of $\mathbf{h}$, and $\mathbf{U}$, the asymptotic covariance matrix of the EPLSM estimator of $\mathbf{h}$, and provide the remark in the following.

Web Corollary 2 *The EPLSM estimator is more efficient than or as efficient as the standard estimator asymptotically, i.e.,* $\mathbf{V}^{-1} - \mathbf{U} \succeq 0$.

The optimization problem in (7) is solved on the Grassmann manifold, which is a set of all d-dimensional subspaces in a p-dimensional space. The optimization on the Grassmann manifold can be slow in high-dimensional settings. To address this, we reparametrize $\mathbf{\Gamma}$ and convert the problem into a nonmanifold optimization as follows. We choose d rows of $\mathbf{\Gamma}$ and then constraint the matrix $\mathbf{\Gamma}_1$ to be nonsingular. This gives us a partition of $\mathbf{\Gamma}$ as

$$\mathbf{\Gamma} = \begin{pmatrix} \mathbf{\Gamma}_1 \\ \mathbf{\Gamma}_2 \end{pmatrix} = \begin{pmatrix} \mathbf{I}_d \\ \mathbf{A} \end{pmatrix} \mathbf{\Gamma}_1 \equiv \mathbf{G}_{\mathbf{A}} \mathbf{\Gamma}_1, \tag{S1}$$

where $\mathbf{A} = \mathbf{\Gamma}_2 \mathbf{\Gamma}_1^{-1}$. Thus, the objective function (7) can be written as $\widehat{\mathbf{A}} = \underset{\mathbf{A} \in \mathbb{R}^{(p-d) \times d}}{\operatorname{argmin}} f_{obj}(\mathbf{A})$,

$$f_{obj}(\mathbf{A}) = -2 \log |\mathbf{G}_{\mathbf{A}}^T \mathbf{G}_{\mathbf{A}}| + \log |\mathbf{G}_{\mathbf{A}}^T \mathbf{S}_{\mathbf{M}|\mathbf{X},\mathbf{Z}}^{-1} \mathbf{G}_{\mathbf{A}}| + \log |\mathbf{G}_{\mathbf{A}}^T \mathbf{S}_{\mathbf{M}|\mathbf{Y},\mathbf{X},\mathbf{Z}} \mathbf{G}_{\mathbf{A}}| + \lambda \sum_{i=1}^{p-d} w_i \|\mathbf{a}_i\|_2, \tag{S2}$$

where $\mathbf{a}_i$ denotes the ith row of $\mathbf{A}$. Therefore, Theorem 6 says that there exists a local minimizer $\hat{\mathbf{A}}$ of (S2) such that $\hat{\mathbf{A}}$ is a $\sqrt{n}$-consistent estimator of $\mathbf{A}$. Thus,

Theorem 7 says that under the conditions in Theorem 7, $\Pr(\hat{\mathbf{a}}_i = \mathbf{0}) \to 1$, for $i = p_{\mathcal{A}} - d + 1, \ldots, p - d$.

We next study the asymptotic variance of the ESPLSM estimator. In preparation, we first define the oracle estimator and study its properties. Suppose we have the oracle information, i.e., we know in advance which mediators are sparsity-selected and which mediators are sparsity-excluded. We would then construct the oracle ESPLSM model by

$$\mathbf{Y} = \boldsymbol{\mu}_{\mathbf{Y}} + \boldsymbol{\eta}^T \boldsymbol{\Gamma}^T \begin{pmatrix} \mathbf{M}_{\mathcal{A}} - \boldsymbol{\mu}_{\mathbf{M}_{\mathcal{A}}} \\ \mathbf{M}_{\mathcal{I}} - \boldsymbol{\mu}_{\mathbf{M}_{\mathcal{I}}} \end{pmatrix} + \boldsymbol{\beta}_{\mathbf{X}}^T (\mathbf{X} - \boldsymbol{\mu}_{\mathbf{X}}) + \boldsymbol{\beta}_{\mathbf{Z}}^T (\mathbf{Z} - \boldsymbol{\mu}_{\mathbf{Z}}) + \boldsymbol{\epsilon},$$

$$\boldsymbol{\Gamma} = \begin{pmatrix} \boldsymbol{\Gamma}_{\mathcal{A}} \\ \mathbf{0} \end{pmatrix}$$

$$\mathbf{M} = \boldsymbol{\mu}_{\mathbf{M}} + \boldsymbol{\gamma}_{\mathbf{X}}^T (\mathbf{X} - \boldsymbol{\mu}_{\mathbf{X}}) + \boldsymbol{\gamma}_{\mathbf{Z}}^T (\mathbf{Z} - \boldsymbol{\mu}_{\mathbf{Z}}) + \mathbf{e} \quad \text{and} \quad \boldsymbol{\Sigma}_{\mathbf{M}|\mathbf{X},\mathbf{Z}} = \boldsymbol{\Gamma}\boldsymbol{\Omega}\boldsymbol{\Gamma}^T + \boldsymbol{\Gamma}_0 \boldsymbol{\Omega}_0 \boldsymbol{\Gamma}_0^T. \tag{S3}$$

Notice that we still include $\mathbf{M}_{\mathcal{I}}$ in the oracle model (S3) even though we know that its coefficients are zero. This is because inclusion of $\mathbf{M}_{\mathcal{I}}$ improves the estimation of $\boldsymbol{\beta}_{\mathbf{M}_{\mathcal{A}}}$. To determine this, we need to look more closely at the immaterial information. When $\boldsymbol{\Gamma}$ has the sparse structure (4), $\boldsymbol{\Gamma}_0$ may have the block diagonal structure

$$\begin{pmatrix} \boldsymbol{\Gamma}_{\mathcal{A},0} & \mathbf{0} \\ \mathbf{0} & \mathbf{I}_{p_{\mathcal{I}}} \end{pmatrix}$$

where $\boldsymbol{\Gamma}_{\mathcal{A},0} \in \mathbb{R}^{p_{\mathcal{A}}\times(p_{\mathcal{A}}-d)}$ is a completion of $\boldsymbol{\Gamma}_{\mathcal{A}}$. If $\boldsymbol{\Gamma}_0$ has this structure, we denote it by $\tilde{\boldsymbol{\Gamma}}_0$. A general structure for $\boldsymbol{\Gamma}_0$ is $\tilde{\boldsymbol{\Gamma}}_0\mathbf{O}$, where $\mathbf{O} \in \mathbb{R}^{(p-d)\times(p-d)}$ is an orthogonal matrix. Then the immaterial part is $\mathbf{Q}_{\mathcal{E}}\mathrm{M} = \mathbf{P}_{\tilde{\boldsymbol{\Gamma}}_0}\mathrm{M} = (\mathrm{M}_{\mathcal{A}}^T\mathbf{Q}_{\boldsymbol{\Gamma}_{\mathcal{A}}}, \mathrm{M}_{\mathcal{I}}^T)^T$. We see that the immaterial part has two sources, one from the immaterial part in the sparsity-selected mediator $\mathbf{Q}_{\boldsymbol{\Gamma}_{\mathcal{A}}}\mathrm{M}_{\mathcal{A}}$ and the other from the sparsity-excluded mediators $\mathrm{M}_{\mathcal{I}}$. Under the basis $\tilde{\boldsymbol{\Gamma}}_0$, we denote the coordinates $\boldsymbol{\Omega}_0$ as $\tilde{\boldsymbol{\Omega}}_0$, where

$$\tilde{\boldsymbol{\Omega}}_0 = \begin{pmatrix} \tilde{\boldsymbol{\Omega}}_{0,\mathcal{A}} & \tilde{\boldsymbol{\Omega}}_{0,\mathcal{A}\mathcal{I}} \\ \tilde{\boldsymbol{\Omega}}_{0,\mathcal{I}\mathcal{A}} & \tilde{\boldsymbol{\Omega}}_{0,\mathcal{I}} \end{pmatrix}$$

and $\tilde{\boldsymbol{\Omega}}_{0,\mathcal{A}} \in \mathbb{R}^{(p_{\mathcal{A}}-d)\times(p_{\mathcal{A}}-d)}$. We can see that the two sources $\mathbf{Q}_{\boldsymbol{\Gamma}_{\mathcal{A}}}\mathrm{M}_{\mathcal{A}}$ and $\mathrm{M}_{\mathcal{I}}$ are correlated with each other, i.e., $\mathrm{Cov}(\mathbf{Q}_{\boldsymbol{\Gamma}_{\mathcal{A}}}\mathrm{M}_{\mathcal{A}}, \mathrm{M}_{\mathcal{I}}) = \boldsymbol{\Gamma}_{\mathcal{A},0}\tilde{\boldsymbol{\Omega}}_{0,\mathcal{A}\mathcal{I}}$ if $\tilde{\boldsymbol{\Omega}}_{0,\mathcal{A}\mathcal{I}} \neq \mathbf{0}$. Therefore, the existence of $\mathrm{M}_{\mathcal{I}}$ helps identify the immaterial part and lowers the cost of estimating $\mathcal{E}$. We can show that the presence of $\mathrm{M}_{\mathcal{I}}$ increases efficiency by comparing the asymptotic variance of the estimators obtained by including or excluding $\mathrm{M}_{\mathcal{I}}$. When we include $\mathrm{M}_{\mathcal{I}}$ in the estimation, Web Proposition 3 gives the expression of the oracle ESPLSM estimator $\widehat{\boldsymbol{\beta}}_{\mathrm{M}_{\mathcal{A}},O}$ as well as its asymptotic variance when $\boldsymbol{\epsilon}$ and $\mathbf{e}$ are normally distributed. We assume normality in this proposition only to obtain an explicit form for the asymptotic variance. It can be proved that without the normality assumption that $\widehat{\boldsymbol{\beta}}_{\mathrm{M}_{\mathcal{A}},O}$ is a $\sqrt{n}$-consistent

estimator as long as $(\boldsymbol{\epsilon}^T, \mathbf{e}^T)^T$ has finite fourth moments. A subscript "O" is attached to an estimator if it is based on the oracle ESPLSM model. Let $\tilde{\boldsymbol{\Omega}}_{0,\mathcal{A}|\mathcal{I}} = \tilde{\boldsymbol{\Omega}}_{0,\mathcal{A}} - \tilde{\boldsymbol{\Omega}}_{0,\mathcal{A}\mathcal{I}}\tilde{\boldsymbol{\Omega}}_{0,\mathcal{I}}^{-1}\tilde{\boldsymbol{\Omega}}_{0,\mathcal{I}\mathcal{A}}$ and $\mathbf{G}_{\mathbf{A}_\mathcal{A}} = (\mathbf{I}_d, \mathbf{A}_\mathcal{A}^T)^T$, where $\mathbf{A}_\mathcal{A}$ denotes the first $p_\mathcal{A} - d$ rows of $\mathbf{A}$. Let $\mathbf{S}_{\mathbf{M}_\mathcal{A}|\mathbf{Y},\mathbf{X},\mathbf{Z}}$, $\mathbf{S}_{\mathbf{M}_\mathcal{A}|\mathbf{X},\mathbf{Z}}$, and $(\mathbf{S}^{-1}_{\mathbf{M}|\mathbf{X},\mathbf{Z}})_\mathcal{A}$ be the upper left $p_\mathcal{A} \times p_\mathcal{A}$ block in $\mathbf{S}_{\mathbf{M}|\mathbf{Y},\mathbf{X},\mathbf{Z}}$, $\mathbf{S}_{\mathbf{M}|\mathbf{X},\mathbf{Z}}$, and $\mathbf{S}^{-1}_{\mathbf{M}|\mathbf{X},\mathbf{Z}}$, respectively.

Web Proposition 3 *Assume that the oracle ESPLSM model* (S3) *holds, and the errors* $\boldsymbol{\epsilon}$ *and* $\mathbf{e}$ *are normally distributed. Then the maximum likelihood estimator (MLE) of* $\boldsymbol{\beta}_{\mathbf{M}_\mathcal{A}}$ *is* $\widehat{\boldsymbol{\beta}}_{\mathbf{M}_\mathcal{A},O} = \mathbf{P}_{\hat{\mathbf{G}}_{\mathbf{A}_\mathcal{A},O}}(\mathbf{S}_{\mathbf{M}_\mathcal{A}|\mathbf{X},\mathbf{Z}})\widehat{\boldsymbol{\beta}}_{\mathbf{M}_\mathcal{A}|\mathbf{X},\mathbf{Z},ols}$*, where*

$$\hat{\mathbf{A}}_{\mathcal{A},O} = \underset{\mathbf{A}_\mathcal{A} \in \mathbb{R}^{(p_\mathcal{A}-d)\times d}}{\operatorname{argmin}} -2\log|\mathbf{G}^T_{\mathbf{A}_\mathcal{A}}\mathbf{G}_{\mathbf{A}_\mathcal{A}}| + \log|\mathbf{G}^T_{\mathbf{A}_\mathcal{A}}\mathbf{S}_{\mathbf{M}_\mathcal{A}|\mathbf{Y},\mathbf{X},\mathbf{Z}}\mathbf{G}_{\mathbf{A}_\mathcal{A}}| + \log|\mathbf{G}^T_{\mathbf{A}_\mathcal{A}}(\mathbf{S}^{-1}_{\mathbf{M}|\mathbf{X},\mathbf{Z}})_\mathcal{A}\mathbf{G}_{\mathbf{A}_\mathcal{A}}|,$$

and $\sqrt{n}\{\operatorname{vec}(\widehat{\boldsymbol{\beta}}_{\mathbf{M}_\mathcal{A},O}) - \operatorname{vec}(\boldsymbol{\beta}_{\mathbf{M}_\mathcal{A}})\} \xrightarrow{d} N(\mathbf{0}, \mathbf{V}_O)$*, where*

$$\begin{aligned}\mathbf{V}_O &= \boldsymbol{\Sigma}_{\mathbf{Y}|\mathbf{M}_\mathcal{A},\mathbf{X},\mathbf{Z}} \otimes \boldsymbol{\Gamma}_\mathcal{A}\boldsymbol{\Omega}^{-1}\boldsymbol{\Gamma}_\mathcal{A}^T + (\boldsymbol{\eta}^T \otimes \boldsymbol{\Gamma}_{\mathcal{A},0})(\boldsymbol{\eta}\boldsymbol{\Sigma}^{-1}_{\mathbf{Y}|\mathbf{M}_\mathcal{A},\mathbf{X},\mathbf{Z}}\boldsymbol{\eta}^T \otimes \tilde{\boldsymbol{\Omega}}_{0,\mathcal{A}} + \boldsymbol{\Omega} \otimes \tilde{\boldsymbol{\Omega}}^{-1}_{0,\mathcal{A}|\mathcal{I}} \\ &\quad + \boldsymbol{\Omega}^{-1} \otimes \tilde{\boldsymbol{\Omega}}_{0,\mathcal{A}} - 2\mathbf{I}_d \otimes \mathbf{I}_{p_\mathcal{A}-d})^{-1}(\boldsymbol{\eta} \otimes \boldsymbol{\Gamma}_{\mathcal{A},0}).\end{aligned}$$

Let

$$\begin{aligned}\mathbf{V}_{\mathbf{M}_\mathcal{A}} &= \boldsymbol{\Sigma}_{\mathbf{Y}|\mathbf{M},\mathbf{X},\mathbf{Z}} \otimes \boldsymbol{\Gamma}_\mathcal{A}\boldsymbol{\Omega}^{-1}\boldsymbol{\Gamma}_\mathcal{A}^T + (\boldsymbol{\eta}^T \otimes \boldsymbol{\Gamma}_{\mathcal{A},0})(\boldsymbol{\eta}\boldsymbol{\Sigma}^{-1}_{\mathbf{Y}|\mathbf{M},\mathbf{X},\mathbf{Z}}\boldsymbol{\eta}^T \otimes \tilde{\boldsymbol{\Omega}}_{0,\mathcal{A}} + \boldsymbol{\Omega} \otimes \tilde{\boldsymbol{\Omega}}^{-1}_{0,\mathcal{A}|\mathcal{I}} \\ &\quad + \boldsymbol{\Omega}^{-1} \otimes \tilde{\boldsymbol{\Omega}}_{0,\mathcal{A}} - 2\mathbf{I}_u \otimes \mathbf{I}_{p_\mathcal{A}-u})^{-1}(\boldsymbol{\eta} \otimes \boldsymbol{\Gamma}^T_{\mathcal{A},0})\end{aligned}$$

and

$$\mathbf{K} = \begin{pmatrix} \mathbf{I}_{p_{\mathcal{A}}} \\ \mathbf{0} \end{pmatrix}$$

Then, we have $\sqrt{n}\{\,\mathrm{vec}(\widehat{\boldsymbol{\beta}}_{\mathbf{X},O}) -\ \mathrm{vec}(\boldsymbol{\beta}_{\mathbf{X}})\} \xrightarrow{d} N(\mathbf{0}, \mathbf{V}_{direct,O})$, *where*

$$\begin{aligned} \mathbf{V}_{direct,O} &= \boldsymbol{\Sigma}_{\mathbf{Y}|\mathbf{M},\mathbf{X},\mathbf{Z}} \otimes (\boldsymbol{\gamma}_{\mathbf{X}}\mathbf{K}\boldsymbol{\Gamma}_{\mathcal{A}}\boldsymbol{\Omega}^{-1}\boldsymbol{\Gamma}_{\mathcal{A}}^T\mathbf{K}^T\boldsymbol{\gamma}_{\mathbf{X}}^T + \boldsymbol{\Sigma}_{\mathbf{X}|\mathbf{Z}}^{-1}) + (\boldsymbol{\eta}^T \otimes \boldsymbol{\gamma}_{\mathbf{X}}\mathbf{K}\boldsymbol{\Gamma}_{\mathcal{A},0})(\boldsymbol{\eta}\boldsymbol{\Sigma}_{\mathbf{Y}|\mathbf{M},\mathbf{X},\mathbf{Z}}^{-1}\boldsymbol{\eta}^T \\ &\quad \otimes\tilde{\boldsymbol{\Omega}}_{0,\mathcal{A}} + \boldsymbol{\Omega} \otimes \tilde{\boldsymbol{\Omega}}_{0,\mathcal{A}|\mathcal{I}}^{-1} + \boldsymbol{\Omega}^{-1} \otimes \tilde{\boldsymbol{\Omega}}_{0,\mathcal{A}} - 2\mathbf{I}_u \otimes \mathbf{I}_{p_{\mathcal{A}}-u})^{-1}(\boldsymbol{\eta} \otimes \boldsymbol{\Gamma}_{\mathcal{A},0}^T\mathbf{K}^T\boldsymbol{\gamma}_{\mathbf{X}}^T) \end{aligned}$$

and $\sqrt{n}\{\,\mathrm{vec}(\hat{\boldsymbol{\gamma}}_{\mathbf{X},O}\widehat{\boldsymbol{\beta}}_{\mathbf{M}_{\mathcal{A}},O}) -\ \mathrm{vec}(\boldsymbol{\gamma}_X\boldsymbol{\beta}_{\mathbf{M}_{\mathcal{A}}})\} \xrightarrow{d} N(\mathbf{0}, \mathbf{V}_{indirect,O})$*, where*

$$\mathbf{V}_{indirect,O} = (\mathbf{I}_{p_{\mathcal{A}}r}\otimes\boldsymbol{\gamma}_{\mathbf{X}})\mathbf{V}_{\mathbf{M}_{\mathcal{A}}}(\mathbf{I}_{p_{\mathcal{A}}r}\otimes\boldsymbol{\gamma}_{\mathbf{X}})^T+(\boldsymbol{\beta}_{\mathbf{M}_{\mathcal{A}}}^T\otimes\mathbf{I}_{kp})(\boldsymbol{\Sigma}_{\mathbf{M}|\mathbf{X},\mathbf{Z}}\otimes\boldsymbol{\Sigma}_{\mathbf{X}|\mathbf{Z}}^{-1})(\boldsymbol{\beta}_{\mathbf{M}_{\mathcal{A}}}\otimes\mathbf{I}_{kp}).$$

If we exclude $\mathbf{M}_{\mathcal{I}}$ in (S3), and only use $\mathbf{M}_{\mathcal{A}}$ to construct the oracle ESPLSM model, the asymptotic variance of the estimator of $\mathrm{vec}(\boldsymbol{\beta}_{\mathbf{M}_{\mathcal{A}}})$ is

$$\begin{aligned} \mathbf{V}_1 &= \boldsymbol{\Sigma}_{\mathbf{Y}|\mathbf{M}_{\mathcal{A}},\mathbf{X},\mathbf{Z}} \otimes \boldsymbol{\Gamma}_{\mathcal{A}}\boldsymbol{\Omega}^{-1}\boldsymbol{\Gamma}_{\mathcal{A}}^T + (\boldsymbol{\eta}^T \otimes \boldsymbol{\Gamma}_{\mathcal{A},0})(\boldsymbol{\eta}\boldsymbol{\Sigma}_{\mathbf{Y}|\mathbf{M}_{\mathcal{A}},\mathbf{X},\mathbf{Z}}^{-1}\boldsymbol{\eta}^T \otimes \tilde{\boldsymbol{\Omega}}_{0,\mathcal{A}} + \boldsymbol{\Omega} \otimes \tilde{\boldsymbol{\Omega}}_{0,\mathcal{A}}^{-1} \\ &\quad +\boldsymbol{\Omega}^{-1} \otimes \tilde{\boldsymbol{\Omega}}_{0,\mathcal{A}} - 2\mathbf{I}_d \otimes \mathbf{I}_{p_{\mathcal{A}}-d})^{-1}(\boldsymbol{\eta} \otimes \boldsymbol{\Gamma}_{\mathcal{A},0}). \end{aligned}$$

Comparing $\mathbf{V}_O$ and $\mathbf{V}_1$, we see that the first summand is exactly the same, but the second summand differs. Specifically, since $\tilde{\boldsymbol{\Omega}}_{0,\mathcal{A}|\mathcal{I}} \leq \tilde{\boldsymbol{\Omega}}_{0,\mathcal{A}}$, $\mathbf{V}_O \leq \mathbf{V}_1$. Moreo-

ever, the higher the correlation between $\mathbf{M}_{\mathcal{A}}$ and $\mathbf{M}_{\mathcal{I}}$, the greater are the efficiency gains.

Web Theorem 4 *Assume that the conditions in Theorem 7 holds. Then* $\sqrt{n}\{\operatorname{vec}(\widehat{\boldsymbol{\beta}}_{\mathbf{M}_{\mathcal{A}}}) - \operatorname{vec}(\boldsymbol{\beta}_{\mathbf{M}_{\mathcal{A}}})\}$ *is asymptotically normally distributed with mean* $\mathbf{0}$ *and variance the same as that of* $\widehat{\boldsymbol{\beta}}_{\mathbf{M}_{\mathcal{A}},O}$. *In addition,* $\sqrt{n}\{\operatorname{vec}(\widehat{\boldsymbol{\beta}}_{\mathbf{X}}) - \operatorname{vec}(\boldsymbol{\beta}_{\mathbf{X}})\}$ *and* $\sqrt{n}\{\operatorname{vec}(\widehat{\boldsymbol{\beta}}_{\mathbf{X},O}) - \operatorname{vec}(\boldsymbol{\beta}_{\mathbf{X}})\}$ *converges to the same asymptotic distribution; and* $\sqrt{n}\{\operatorname{vec}(\hat{\boldsymbol{\gamma}}_{\mathbf{X}}\widehat{\boldsymbol{\beta}}_{\mathbf{M}}) - \operatorname{vec}(\boldsymbol{\gamma}_{\mathbf{X}}\boldsymbol{\beta}_{\mathbf{M}})\}$ *and* $\sqrt{n}\{\operatorname{vec}(\hat{\boldsymbol{\gamma}}_{\mathbf{X},O}\widehat{\boldsymbol{\beta}}_{\mathbf{M},O}) - \operatorname{vec}(\boldsymbol{\gamma}_{\mathbf{X}}\boldsymbol{\beta}_{\mathbf{M}})\}$ *converges to the same asymptotic distribution.*

Web Theorem 4 indicates that the ESPLSM estimator has the optimal estimation rate. Together with Theorem 7, it shows that the ESPLSM estimator enjoys the oracle property, it correctly selects the sparsity-excluded predictors with probability tending to 1, and estimates the coefficients of the sparsity-selected predictors with the same efficiency as if the true model were known.

While Web Proposition 1 concerns the fixed p case, the following theorem extends the theoretical properties of the ESPLSM estimator to the high-dimensional regime, where p diverges with n. Since p varies with n, we denote p as p_n. Let s_1 and s_2 denote the number of nonzero off-diagonal elements in the lower triangle of $\boldsymbol{\Sigma}^{-1}_{\mathbf{M}|\mathbf{X},\mathbf{Z}}$ and $\boldsymbol{\Sigma}^{-1}_{\mathbf{M}|\mathbf{Y},\mathbf{X},\mathbf{Z}}$, and $s = \max\{s_1, s_2\}$. We also need the sub-Gaussian assumption for deriving the convergence rate.

Web Definition 5 *A random variable* V *with mean* μ_V *follows a sub-Gaussian distribution with parameter* σ^2 *if* $E[\exp\{t(V-\mu_V)\}] \leq \exp(t^2\sigma^2/2)$ *for all* $t \in \mathbb{R}$.

Web Theorem 6 *Assume that the ESPLSM holds, the largest eigenvalue of* $\mathbf{\Sigma}_{\mathbf{M}}$ *is upper bounded by a constant* $\bar{k}$*, and the smallest eigenvalue of* $\mathbf{\Sigma}_{\mathbf{M}|\mathbf{Y},\mathbf{X},\mathbf{Z}}$ *is lower bounded by a constant* $\underline{k}$*. Let* $\mathbf{V} = (\mathbf{X}^T - \boldsymbol{\mu}_{\mathbf{X}}^T, \mathbf{Z}^T - \boldsymbol{\mu}_{\mathbf{Z}}^T, \mathbf{e}^T, \boldsymbol{\epsilon}^T)^T \in \mathbb{R}^{k+q+p_n+r}$ *and* $\mathbf{\Sigma}_{\mathbf{V}}$ *denote the covariance matrix of* $\mathbf{V}$*. Assume that each* $\mathbf{V}_i/\sqrt{\mathbf{\Sigma}_{\mathbf{V},ii}}, i = 1, \ldots, k+q+p_n+r$ *follows a sub-Gaussian distribution with parameter* σ^2*, where* $\mathbf{V}_i$ *is the* i*th element in* $\mathbf{V}$*, and* $\mathbf{\Sigma}_{\mathbf{V},ii}$ *denotes the* (i,i)*th element in* $\mathbf{\Sigma}_{\mathbf{V}}$*. We further assume* $\|\boldsymbol{\gamma}_{\mathbf{X}}\|_{\max} \leq C_{\mathbf{X}}$ *and* $\|\boldsymbol{\gamma}_{\mathbf{Z}}\|_{\max} \leq C_{\mathbf{Z}}$*. If* $\lambda_{\mathcal{A}} = o(\sqrt{(p_n+s)\log(p_n)/n})$*, then there exists a local minimizer* $\hat{\mathbf{A}}$ *of*

$$\hat{\mathbf{A}} = \underset{\mathbf{A}_{\mathcal{A}} \in \mathbb{R}^{(p_n-d)\times d}}{\operatorname{argmin}} -2\log|\mathbf{G}_{\mathbf{A}}^T\mathbf{G}_{\mathbf{A}}| + \log|\mathbf{G}_{\mathbf{A}}^T\mathbf{S}_{\mathbf{M}|\mathbf{X},\mathbf{Z},sp}^{-1}\mathbf{G}_{\mathbf{A}}| + \log|\mathbf{G}_{\mathbf{A}}^T\mathbf{S}_{\mathbf{M}|\mathbf{Y},\mathbf{X},\mathbf{Z},sp}\mathbf{G}_{\mathbf{A}}| + \lambda\sum_{i=1}^{p_n-d} w_i\|\boldsymbol{a}_i\|_2 \tag{S4}$$

such that $\|\hat{\mathbf{A}} - \mathbf{A}\|_F = O_p((p_n+s)\log(p_n)/n)$*, and the ESPLSM estimators converge at the same rate* $\|\widehat{\boldsymbol{\beta}}_{\mathbf{X}} - \boldsymbol{\beta}_{\mathbf{X}}\|_F = \|\hat{\boldsymbol{\gamma}}_{\mathbf{X}} - \boldsymbol{\gamma}_{\mathbf{X}}\|_F = \|\widehat{\boldsymbol{\beta}}_{\mathbf{M}} - \boldsymbol{\beta}_{\mathbf{M}}\|_F = O_p(\sqrt{(p_n+s)\log(p_n)/n})$*, and* $\|\hat{\boldsymbol{\gamma}}_{\mathbf{X}}\widehat{\boldsymbol{\beta}}_{\mathbf{M}} - \boldsymbol{\gamma}_{\mathbf{X}}\boldsymbol{\beta}_{\mathbf{M}}\|_F = O_p(\sqrt{(p_n+s)\log(p_n)/n})$.

Web Theorem 6 gives the convergence rate of ESPLSM estimator. It is the same as that of the SPICE estimators of $\mathbf{\Sigma}_{\mathbf{M}|\mathbf{X},\mathbf{Z}}$ and $\mathbf{\Sigma}_{\mathbf{M}|\mathbf{Y},\mathbf{X},\mathbf{Z}}$, which are used in the

objective function (S4). Since SPICE does not assume sparsity on $\boldsymbol{\Sigma}^{-1}_{\mathbf{M}|\mathbf{X},\mathbf{Z}}$ or $\boldsymbol{\Sigma}^{-1}_{\mathbf{M}|\mathbf{Y},\mathbf{X},\mathbf{Z}}$, the total number of parameters under the ESPLSM model is $r + p_n + k + q + r(p_n + k + q) + p_n(k + q) + r(r + 1)/2 + p_n(p_n + 1)/2$, which is of the order of p_n^2. We can improve the convergence rate to a faster rate if we further impose sparsity assumption on $\boldsymbol{\Sigma}^{-1}_{\mathbf{M}|\mathbf{X},\mathbf{Z}}$, e.g., assume that the total number of non-zero off-diagonal elements is fixed number or grows slower than n.

Web Theorem 7 *Assume that the conditions in Web Theorem 6 holds,* $\sqrt{(p_n + s)\log(p_n)/n} \to 0$ *as* $n \to \infty$ *and* $\sqrt{(p_n + s)\log(p_n)/n} = o(\lambda_I)$. *Then* $\Pr(\hat{\boldsymbol{a}}_i \neq 0) \to 1$ *for* $i = 1, \ldots, p_{\mathcal{A}} - d$ *and* $\Pr(\hat{\boldsymbol{a}}_i = 0, i = p_{\mathcal{A}} - d + 1, \ldots, p_n - d) \to 1.$

Web Theorem 7 establishes the selection consistency of the ESPLSM estimator, i.e., when $p_n \to \infty$ as $n \to \infty$, the ESPLSM estimator correctly identifies sparsity-selected and excluded predictors with probability tending to 1. Regarding the tuning parameters, the condition $\lambda_{\mathcal{A}} = o(\sqrt{(p_n + s)\log(p_n)/n})$ is equivalent to $n^{1/2}(p_n + s)^{-1/2}\{\log(p_n)\}^{-1/2}\lambda \to 0$. With $\lambda_{\mathcal{I}}$, the range of λ depends on how fast $\min\{w_{p_{\mathcal{A}}-d+1}, \ldots, w_{p_n-d}\}$ diverges. If $\min\{w_{p_{\mathcal{A}}-d+1}, \ldots, w_{p_n-d}\} = O_p(n^{v_1}p_n^{v_2})$, where $v_1 > 0$ and $v_2 > 0$, then $\sqrt{(p_n + s)\log(p_n)/n} = o(\lambda_{\mathcal{I}})$ is equivalent to $n^{(1+2v_1)/2}p_n^{v_2}(p_n + s)^{-1/2}\{\log(p_n)\}^{-1/2}\lambda \to \infty$. If λ satisfies both conditions, then the assumptions of the tuning parameters in both Web Theorem 6

and 7 hold. But Web Theorem 6 only requires that λ satisfy the former assumption.

Web Appendix B: Proofs of theoretical results

Proof of Proposition 1 By A1, there is no interference between units and no hidden versions of the exposure, ensuring that all potential outcomes $\mathbf{Y}(\mathbf{x}, \mathbf{m})$ and $\mathbf{M}(\mathbf{x})$ are well-defined for each individual. For any $\mathbf{z}$ in the support of $\mathcal{Z} \subseteq \mathbb{R}^q$, define the conditional ACME contrast

$$\mathbf{C}_{\mathbf{x},\mathbf{x}'}(\mathbf{z}) = \mathrm{E}[\mathbf{Y}(\mathbf{x}, \mathbf{M}(\mathbf{x})) - \mathbf{Y}(\mathbf{x}, \mathbf{M}(\mathbf{x}'))|\mathbf{Z} = \mathbf{z}]$$

and the conditional ANDE contrast

$$\mathbf{D}_{\mathbf{x},\mathbf{x}'}(\mathbf{z}) = \mathrm{E}[\mathbf{Y}(\mathbf{x}, \mathbf{M}(\mathbf{x}')) - \mathbf{Y}(\mathbf{x}', \mathbf{M}(\mathbf{x}'))|\mathbf{Z} = \mathbf{z}].$$

By the law of iterated expectations, $\bar{\boldsymbol{\delta}}(\mathbf{x}, \mathbf{x}') = \int \mathbf{C}_{\mathbf{x},\mathbf{x}'}(\mathbf{z}) dF_{\mathbf{Z}}(\mathbf{z})$ and $\bar{\boldsymbol{\zeta}}(\mathbf{x}, \mathbf{x}') = \int \mathbf{D}_{\mathbf{x},\mathbf{x}'}(\mathbf{z}) dF_{\mathbf{Z}}(\mathbf{z})$, so it suffices to identify $\mathbf{C}_{\mathbf{x},\mathbf{x}'}(\mathbf{z})$ and $\mathbf{D}_{\mathbf{x},\mathbf{x}'}(\mathbf{z})$ for each fixed $\mathbf{z}$.

The first part of sequential ignorability (i.e., A3) says that (i) $\mathbf{M}(\mathbf{x}) \perp\!\!\!\perp \mathbf{X}|\mathbf{Z} = \mathbf{z}$

for any $\mathbf{x}$; (ii) $\mathbf{Y}(\mathbf{x}', \mathbf{m}) \perp \mathbf{X}|\mathbf{Z} = \mathbf{z}$ for any $\mathbf{x}' \in \mathbb{R}^k$ and any $\mathbf{m} \in \mathbf{M}(\mathbf{x}')$. By A2, (i) gives that for any measurable set $\mathbf{A} \subseteq \mathbb{R}^p$, $\Pr(\mathbf{M}(\mathbf{x}) \in \mathbf{A}|\mathbf{Z} = \mathbf{z}) = \Pr(\mathbf{M} \in \mathbf{A}|\mathbf{X} = \mathbf{x}, \mathbf{Z} = \mathbf{z})$. Equivalently,

$$F_{\mathbf{M}(\mathbf{x})|\mathbf{Z}=\mathbf{z}} = F_{\mathbf{M}|\mathbf{X}=\mathbf{x},\mathbf{Z}=\mathbf{z}}. \tag{S5}$$

By A4, this conditional distribution is well-defined for all values of $\mathbf{x}$ and $\mathbf{z}$ with positive probability. Also, (ii) gives

$$\mathrm{E}(\mathbf{Y}(\mathbf{x}', \mathbf{m})|\mathbf{Z} = \mathbf{z}) = \mathrm{E}(\mathbf{Y}(\mathbf{x}', \mathbf{m})|\mathbf{X} = \mathbf{x}', \mathbf{Z} = \mathbf{z}). \tag{S6}$$

The second part of sequential ignorability (i.e., A3) says $\mathbf{Y}(\mathbf{x}, \mathbf{m}) \perp \mathbf{M}(\mathbf{x})|\{\mathbf{X} = \mathbf{x}, \mathbf{Z} = \mathbf{z}\}$, and we have $\mathrm{E}(\mathbf{Y}(\mathbf{x}, \mathbf{m})|\mathbf{X} = \mathbf{x}, \mathbf{Z} = \mathbf{z}) = \mathrm{E}(\mathbf{Y}|\mathbf{M} = \mathbf{m}, \mathbf{X} = \mathbf{x}, \mathbf{Z} = \mathbf{z})$. This is a vector equality in $\mathbb{R}^r$, understood componentwise, and again uses consistency to identify $\mathbf{Y}(\mathbf{x}, \mathbf{m})$ with the observed $\mathbf{Y}$ when we intervene on $\mathbf{M}$ at value $\mathbf{m}$ within $\mathbf{X} = \mathbf{x}, \mathbf{Z} = \mathbf{z}$. Combining this with (S6), we obtain

$$\mathrm{E}(\mathbf{Y}(\mathbf{x}, \mathbf{m})|\mathbf{Z} = \mathbf{z}) = \mathrm{E}(\mathbf{Y}|\mathbf{M} = \mathbf{m}, \mathbf{X} = \mathbf{x}, \mathbf{Z} = \mathbf{z}), \tag{S7}$$

where the equality is vector-valued when $\mathbf{Y} \in \mathbb{R}^r$, and it holds componentwise.

Again, A4 guarantees that the above conditional expectation is identifiable from the observed data.

By the law of iterated expectations, i.e., $\mathrm{E}(\mathbf{Y}(\mathbf{x}, \mathbf{M}(\mathbf{x}))|\mathbf{Z} = \mathbf{z}) = \int \mathrm{E}(\mathbf{Y}(\mathbf{x}, \mathbf{m})|\mathbf{Z} = \mathbf{z}) dF_{\mathbf{M}(\mathbf{x})|\mathbf{Z}=\mathbf{z}}(\mathbf{m})$, (S5) and (S7) gives

$$\mathrm{E}(\mathbf{Y}(\mathbf{x}, \mathbf{M}(\mathbf{x}))|\mathbf{Z} = \mathbf{z}) = \int \mathrm{E}(\mathbf{Y}|\mathbf{M} = \mathbf{m}, \mathbf{X} = \mathbf{x}, \mathbf{Z} = \mathbf{z}) dF_{\mathbf{M}|\mathbf{X}=\mathbf{x},\mathbf{Z}=\mathbf{z}}(\mathbf{m}) \tag{S8}$$

and

$$\mathrm{E}(\mathbf{Y}(\mathbf{x}, \mathbf{M}(\mathbf{x}'))|\mathbf{Z} = \mathbf{z}) = \int \mathrm{E}(\mathbf{Y}|\mathbf{M} = \mathbf{m}, \mathbf{X} = \mathbf{x}, \mathbf{Z} = \mathbf{z}) dF_{\mathbf{M}|\mathbf{X}=\mathbf{x}',\mathbf{Z}=\mathbf{z}}(\mathbf{m}). \tag{S9}$$

By definition of the conditional ACME contrast, combining (S8) and (S9) gives

$$\begin{aligned}
\mathbf{C}_{\mathbf{x},\mathbf{x}'}(\mathbf{z}) &= \mathrm{E}[\mathbf{Y}(\mathbf{x}, \mathbf{M}(\mathbf{x}))|\mathbf{Z} = \mathbf{z}] - \mathrm{E}[\mathbf{Y}(\mathbf{x}, \mathbf{M}(\mathbf{x}'))|\mathbf{Z} = \mathbf{z}] \\
&= \int \mathrm{E}(\mathbf{Y}|\mathbf{M} = \mathbf{m}, \mathbf{X} = \mathbf{x}, \mathbf{Z} = \mathbf{z}) dF_{\mathbf{M}|\mathbf{X}=\mathbf{x},\mathbf{Z}=\mathbf{z}}(\mathbf{m}) \\
&\quad - \int \mathrm{E}(\mathbf{Y}|\mathbf{M} = \mathbf{m}, \mathbf{X} = \mathbf{x}, \mathbf{Z} = \mathbf{z}) dF_{\mathbf{M}|\mathbf{X}=\mathbf{x}',\mathbf{Z}=\mathbf{z}}(\mathbf{m}).
\end{aligned}$$

This is now written entirely in terms of observable quantities. Because all expectations and integrals are vector-valued and interpreted componentwise, $\mathbf{C}_{\mathbf{x},\mathbf{x}'}(\mathbf{z})$ is

identified. Marginalizing over $\mathbf{Z}$, the ACME is identified. By the same reasoning, we have

$$\begin{aligned}\mathbf{D}_{\mathbf{x},\mathbf{x}'}(\mathbf{z}) &= \int \mathrm{E}(\mathbf{Y}|\mathbf{M}=\mathbf{m},\mathbf{X}=\mathbf{x},\mathbf{Z}=\mathbf{z})dF_{\mathbf{M}|\mathbf{X}=\mathbf{x}',\mathbf{Z}=\mathbf{z}}(\mathbf{m}) \\ &\quad - \int \mathrm{E}(\mathbf{Y}|\mathbf{M}=\mathbf{m},\mathbf{X}=\mathbf{x}',\mathbf{Z}=\mathbf{z})dF_{\mathbf{M}|\mathbf{X}=\mathbf{x}',\mathbf{Z}=\mathbf{z}}(\mathbf{m})\end{aligned}$$

leading to $\bar{\boldsymbol{\zeta}}(\mathbf{x},\mathbf{x}') = \int \mathbf{D}_{\mathbf{x},\mathbf{x}'}(\mathbf{z})dF_{\mathbf{Z}}(\mathbf{z})$ again in terms of observed quantities only.

Proof of Proposition 2 By A1 and A2, the causal effects defined in terms of potential outcomes coincide with functions of observable variables. As shown in Proposition 1, under A1-A4, both multivariate ACME and ANDE are nonparametrically identified and can be written as contrasts of conditional expectations of the form $\mathrm{E}(\mathbf{Y}|\mathbf{M},\mathbf{X},\mathbf{Z})$. Therefore, it suffices to characterize these conditional expectations under the EPLSM model.

We first restate the equation (3) in potential outcome form. Interpreting $\mathbf{M}$ causally, the mediator that would be realized if we were to set the exposure to an arbitrary value $\mathbf{x} \in \mathbb{R}^k$ is $\mathbf{M}(\mathbf{x}) = \boldsymbol{\mu}_{\mathbf{M}} + \boldsymbol{\gamma}_{\mathbf{X}}^T(\mathbf{x} - \boldsymbol{\mu}_{\mathbf{X}}) + \boldsymbol{\gamma}_{\mathbf{Z}}^T(\mathbf{Z} - \boldsymbol{\mu}_{\mathbf{Z}}) + \mathbf{e}$. This is an $\mathbb{R}^p$-valued vector. Note in particular that $\mathbf{M}(\mathbf{x})$ depends linearly on $\mathbf{x}$, with slope matrix $\boldsymbol{\gamma}_{\mathbf{X}}^T \in \mathbb{R}^{p\times k}$. Interpreting $\mathbf{Y}$ causally, the outcome that would be realized

if we intervened to set the exposure $\mathbf{x} \in \mathbb{R}^k$ and set the mediator to $\mathbf{m} \in \mathbb{R}^p$ is $\mathbf{Y}(\mathbf{x}, \mathbf{m}) = \boldsymbol{\mu}_{\mathbf{Y}} + \boldsymbol{\eta}^T \boldsymbol{\Gamma}^T (\mathbf{m} - \boldsymbol{\mu}_{\mathbf{M}}) + \boldsymbol{\beta}_{\mathbf{X}}^T (\mathbf{x} - \boldsymbol{\mu}_{\mathbf{X}}) + \boldsymbol{\beta}_{\mathbf{Z}}^T (\mathbf{Z} - \boldsymbol{\mu}_{\mathbf{Z}}) + \boldsymbol{\epsilon}$. This is an $\mathbb{R}^r$-valued vector. Crucially, the same coefficient matrices $\boldsymbol{\beta}_{\mathbf{X}}^T$ and $\boldsymbol{\eta}^T \boldsymbol{\Gamma}^T$ appear in the counterfactual outcome.

Now consider the first term in the ACME contrast, $\mathbf{Y}(\mathbf{x}, \mathbf{M}(\mathbf{x})) = \boldsymbol{\mu}_{\mathbf{Y}} + \boldsymbol{\eta}^T \boldsymbol{\Gamma}^T (\mathbf{M}(\mathbf{x}) - \boldsymbol{\mu}_{\mathbf{M}}) + \boldsymbol{\beta}_{\mathbf{X}}^T (\mathbf{x} - \boldsymbol{\mu}_{\mathbf{X}}) + \boldsymbol{\beta}_{\mathbf{Z}}^T (\mathbf{Z} - \boldsymbol{\mu}_{\mathbf{Z}}) + \boldsymbol{\epsilon}$. and the second term, $\mathbf{Y}(\mathbf{x}, \mathbf{M}(\mathbf{x}')) = \boldsymbol{\mu}_{\mathbf{Y}} + \boldsymbol{\eta}^T \boldsymbol{\Gamma}^T (\mathbf{M}(\mathbf{x}') - \boldsymbol{\mu}_{\mathbf{M}}) + \boldsymbol{\beta}_{\mathbf{X}}^T (\mathbf{x} - \boldsymbol{\mu}_{\mathbf{X}}) + \boldsymbol{\beta}_{\mathbf{Z}}^T (\mathbf{Z} - \boldsymbol{\mu}_{\mathbf{Z}}) + \boldsymbol{\epsilon}$. Subtracting gives $\mathbf{Y}(\mathbf{x}, \mathbf{M}(\mathbf{x})) - \mathbf{Y}(\mathbf{x}, \mathbf{M}(\mathbf{x}')) = \boldsymbol{\eta}^T \boldsymbol{\Gamma}^T (\mathbf{M}(\mathbf{x}) - \mathbf{M}(\mathbf{x}'))$. By the mediator equation, we can write

$$\begin{aligned} \mathbf{M}(\mathbf{x}) - \mathbf{M}(\mathbf{x}') &= \{\boldsymbol{\mu}_{\mathbf{M}} + \boldsymbol{\gamma}_{\mathbf{X}}^T (\mathbf{x} - \boldsymbol{\mu}_{\mathbf{X}}) + \boldsymbol{\gamma}_{\mathbf{Z}}^T (\mathbf{Z} - \boldsymbol{\mu}_{\mathbf{Z}}) + \mathbf{e}\} \\ &\quad - \{\boldsymbol{\mu}_{\mathbf{M}} + \boldsymbol{\gamma}_{\mathbf{X}}^T (\mathbf{x}' - \boldsymbol{\mu}_{\mathbf{X}}) + \boldsymbol{\gamma}_{\mathbf{Z}}^T (\mathbf{Z} - \boldsymbol{\mu}_{\mathbf{Z}}) + \mathbf{e}\} = \boldsymbol{\gamma}_{\mathbf{X}}^T (\mathbf{x} - \mathbf{x}') \end{aligned}$$

This difference no longer depends on $\mathbf{Z}$ or the error terms. Subtracting back,

$$\mathbf{Y}(\mathbf{x}, \mathbf{M}(\mathbf{x})) - \mathbf{Y}(\mathbf{x}, \mathbf{M}(\mathbf{x}')) = \boldsymbol{\eta}^T \boldsymbol{\Gamma}^T \boldsymbol{\gamma}_{\mathbf{X}}^T (\mathbf{x} - \mathbf{x}').$$

This is an $\mathbb{R}^r$-valued vector that is constant across individual unit. Therefore, taking expectations over the population, $\bar{\boldsymbol{\delta}}(\mathbf{x}, \mathbf{x}') = \mathrm{E}_{\mathbf{Z}}[\mathrm{E}\{\mathbf{Y}(\mathbf{x}, \mathbf{M}(\mathbf{x})) - \mathbf{Y}(\mathbf{x}, \mathbf{M}(\mathbf{x}')) | \mathbf{Z}\}] = \boldsymbol{\eta}^T \boldsymbol{\Gamma}^T \boldsymbol{\gamma}_{\mathbf{X}}^T (\mathbf{x} - \mathbf{x}') = \boldsymbol{\beta}_{\mathbf{M}}^T \boldsymbol{\gamma}_{\mathbf{X}}^T (\mathbf{x} - \mathbf{x}')$, which proves statement.

Next, we consider the ANDE. The two terms in ANDE contrast are

$$\begin{aligned}\mathbf{Y}(\mathbf{x},\mathbf{M}(\mathbf{x}')) &= \boldsymbol{\mu}_{\mathbf{Y}} + \boldsymbol{\eta}^T\boldsymbol{\Gamma}^T(\mathbf{M}(\mathbf{x}') - \boldsymbol{\mu}_{\mathbf{M}}) + \boldsymbol{\beta}_{\mathbf{X}}^T(\mathbf{x} - \boldsymbol{\mu}_{\mathbf{X}}) + \boldsymbol{\beta}_{\mathbf{Z}}^T(\mathbf{Z} - \boldsymbol{\mu}_{\mathbf{Z}}) + \boldsymbol{\epsilon} \\ \mathbf{Y}(\mathbf{x}',\mathbf{M}(\mathbf{x}')) &= \boldsymbol{\mu}_{\mathbf{Y}} + \boldsymbol{\eta}^T\boldsymbol{\Gamma}^T(\mathbf{M}(\mathbf{x}') - \boldsymbol{\mu}_{\mathbf{M}}) + \boldsymbol{\beta}_{\mathbf{X}}^T(\mathbf{x}' - \boldsymbol{\mu}_{\mathbf{X}}) + \boldsymbol{\beta}_{\mathbf{Z}}^T(\mathbf{Z} - \boldsymbol{\mu}_{\mathbf{Z}}) + \boldsymbol{\epsilon}.\end{aligned}$$

Subtracting gives $\mathbf{Y}(\mathbf{x},\mathbf{M}(\mathbf{x}')) - \mathbf{Y}(\mathbf{x}',\mathbf{M}(\mathbf{x}')) = \boldsymbol{\beta}_{\mathbf{X}}^T(\mathbf{x} - \mathbf{x}')$. Taking expectations, we have $\bar{\boldsymbol{\zeta}}(\mathbf{x},\mathbf{x}') = \mathrm{E}_{\mathbf{Z}}[\mathrm{E}\{\mathbf{Y}(\mathbf{x},\mathbf{M}(\mathbf{x}')) - \mathbf{Y}(\mathbf{x}',\mathbf{M}(\mathbf{x}'))|\mathbf{Z}\}] = \boldsymbol{\beta}_{\mathbf{X}}^T(\mathbf{x}-\mathbf{x}')$, which proves statement.

Finally, we derive the total effect of moving the exposure from $\mathbf{x}'$ to $\mathbf{x}$. The observed outcome under exposure $\mathbf{x}$ and the corresponding mediator $\mathbf{M}(\mathbf{x})$ is

$$\mathbf{Y}(\mathbf{x},\mathbf{M}(\mathbf{x})) = \boldsymbol{\mu}_{\mathbf{Y}} + \boldsymbol{\eta}^T\boldsymbol{\Gamma}^T(\mathbf{M}(\mathbf{x}) - \boldsymbol{\mu}_{\mathbf{M}}) + \boldsymbol{\beta}_{\mathbf{X}}^T(\mathbf{x} - \boldsymbol{\mu}_{\mathbf{X}}) + \boldsymbol{\beta}_{\mathbf{Z}}^T(\mathbf{Z} - \boldsymbol{\mu}_{\mathbf{Z}}) + \boldsymbol{\epsilon}$$

Hence,

$$\mathbf{Y}(\mathbf{x},\mathbf{M}(\mathbf{x})) - \mathbf{Y}(\mathbf{x}',\mathbf{M}(\mathbf{x}')) = \{\boldsymbol{\eta}^T\boldsymbol{\Gamma}^T\mathbf{M}(\mathbf{x}) + \boldsymbol{\beta}_{\mathbf{X}}^T\mathbf{x}\} - \{\boldsymbol{\eta}^T\boldsymbol{\Gamma}^T\mathbf{M}(\mathbf{x}') + \boldsymbol{\beta}_{\mathbf{X}}^T\mathbf{x}'\}.$$

Taking expectations, $\mathrm{E}\{\mathbf{Y}(\mathbf{x},\mathbf{M}(\mathbf{x})) - \mathbf{Y}(\mathbf{x}',\mathbf{M}(\mathbf{x}'))\} = (\boldsymbol{\eta}^T\boldsymbol{\Gamma}^T\boldsymbol{\gamma}_{\mathbf{X}}^T + \boldsymbol{\beta}_{\mathbf{X}}^T)(\mathbf{x} - \mathbf{x}')$, and by A2, we have $\mathrm{E}\{\mathbf{Y}(\mathbf{x},\mathbf{M}(\mathbf{x})) - \mathbf{Y}(\mathbf{x}',\mathbf{M}(\mathbf{x}'))\} = \mathrm{E}(\mathbf{Y}|\mathbf{X} = \mathbf{x}) - \mathrm{E}(\mathbf{Y}|\mathbf{X} = \mathbf{x}')$. Then, $\mathrm{E}(\mathbf{Y}|\mathbf{X} = \mathbf{x}) - \mathrm{E}(\mathbf{Y}|\mathbf{X} = \mathbf{x}') = (\boldsymbol{\eta}^T\boldsymbol{\Gamma}^T\boldsymbol{\gamma}_{\mathbf{X}}^T + \boldsymbol{\beta}_{\mathbf{X}}^T)(\mathbf{x} - \mathbf{x}')$,

which proves statement. Note that $(\boldsymbol{\eta}^T\boldsymbol{\Gamma}^T\boldsymbol{\gamma}_{\mathbf{X}}^T + \boldsymbol{\beta}_{\mathbf{X}}^T)(\mathbf{x}-\mathbf{x}') = \boldsymbol{\beta}_{\mathbf{X}}^T(\mathbf{x}-\mathbf{x}') + \boldsymbol{\eta}^T\boldsymbol{\Gamma}^T\boldsymbol{\gamma}_{\mathbf{X}}^T(\mathbf{x}-\mathbf{x}') = \bar{\boldsymbol{\delta}}(\mathbf{x},\mathbf{x}') + \bar{\boldsymbol{\zeta}}(\mathbf{x},\mathbf{x}')$, so the total causal effect decomposes cleanly into a direct component and a mediated component. This completes the proof.

Proof of Proposition 3 Because $\mathbf{A}$ is invertible, the mapping $\mathbf{m} \to \mathbf{m}^\dagger = \mathbf{A}\mathbf{m}$ is one-to-one with inverse $\mathbf{m} = \mathbf{A}^{-1}\mathbf{m}^\dagger$. For each exposure level $\mathbf{x}$, define the potential mediator under exposure $\mathbf{x}$ as $\mathbf{M}(\mathbf{x})$, and define the reparameterized potential mediator by $\mathbf{M}^\dagger(\mathbf{x}) = \mathbf{A}\mathbf{M}(\mathbf{x})$. For any fixed $\mathbf{x}$ and any potential mediator value $\mathbf{m}^\dagger$, define the re-indexed potential outcome $\mathbf{Y}^\dagger(\mathbf{x},\mathbf{m}^\dagger) = \mathbf{Y}(\mathbf{x},\mathbf{A}^{-1}\mathbf{m}^\dagger)$. Then for each $\mathbf{x}$, $\mathbf{Y}^\dagger(\mathbf{x},\mathbf{M}^\dagger(\mathbf{x})) = \mathbf{Y}(\mathbf{x},\mathbf{A}^{-1}\mathbf{A}\mathbf{M}(\mathbf{x})) = \mathbf{Y}(\mathbf{x},\mathbf{M}(\mathbf{x}))$, and similarly, $\mathbf{Y}^\dagger(\mathbf{x},\mathbf{M}^\dagger(\mathbf{x}')) = \mathbf{Y}(\mathbf{x},\mathbf{M}(\mathbf{x}'))$ and $\mathbf{Y}^\dagger(\mathbf{x}',\mathbf{M}^\dagger(\mathbf{x}')) = \mathbf{Y}(\mathbf{x}',\mathbf{M}(\mathbf{x}'))$. Since identification under A3 is defined through conditional expectation given $\mathbf{Z}$, we express the causal effects using iterated expectations with respect to $\mathbf{Z}$. Accordingly, the ACME and ANDE under the reparameterized mediator satisfy

$$
\begin{aligned}
\bar{\boldsymbol{\delta}}^\dagger(\mathbf{x},\mathbf{x}') &= \mathrm{E}_{\mathbf{Z}}[\mathrm{E}\{\mathbf{Y}^\dagger(\mathbf{x},\mathbf{M}^\dagger(\mathbf{x})) - \mathbf{Y}^\dagger(\mathbf{x},\mathbf{M}^\dagger(\mathbf{x}'))|\mathbf{Z}\}] \\
&= \mathrm{E}_{\mathbf{Z}}[\mathrm{E}\{\mathbf{Y}(\mathbf{x},\mathbf{M}(\mathbf{x})) - \mathbf{Y}(\mathbf{x},\mathbf{M}(\mathbf{x}'))|\mathbf{Z}\}] = \bar{\boldsymbol{\delta}}(\mathbf{x},\mathbf{x}')
\end{aligned}
$$

and

$$\begin{aligned}\bar{\boldsymbol{\zeta}}^{\dagger}(\mathbf{x},\mathbf{x}') &= \mathrm{E}_{\mathbf{Z}}[\mathrm{E}\{\mathbf{Y}^{\dagger}(\mathbf{x},\mathbf{M}^{\dagger}(\mathbf{x}')) - \mathbf{Y}^{\dagger}(\mathbf{x}',\mathbf{M}^{\dagger}(\mathbf{x}'))|\mathbf{Z}\}] \\ &= \mathrm{E}_{\mathbf{Z}}[\mathrm{E}\{\mathbf{Y}(\mathbf{x},\mathbf{M}(\mathbf{x}')) - \mathbf{Y}(\mathbf{x}',\mathbf{M}(\mathbf{x}'))|\mathbf{Z}\}] = \bar{\boldsymbol{\zeta}}(\mathbf{x},\mathbf{x}').\end{aligned}$$

This proves the invariance of the causal functionals.

Next, we show that the identification cassumptions A1-A4 remain valid under the reparameterized mediator $\mathbf{M}^{\dagger} = \mathbf{AM}$. A1 is unaffected by deterministic reparameterization of $\mathbf{M}$. A2 holds since $\mathbf{M}^{\dagger} = \mathbf{AM}$ is a deterministic transformation. The observed mediator satisfies $\mathbf{M}^{\dagger} = \mathbf{M}^{\dagger}(\mathbf{X}) = \mathbf{AM}(\mathbf{X})$, and the observed outcome satisfies $\mathbf{Y} = \mathbf{Y}(\mathbf{X},\mathbf{M}(\mathbf{X})) = \mathbf{Y}^{\dagger}(\mathbf{X},\mathbf{M}^{\dagger}(\mathbf{X}))$, where $\mathbf{Y}^{\dagger}(\mathbf{x},\mathbf{m}^{\dagger}) = \mathbf{Y}(\mathbf{x},\mathbf{A}^{-1}\mathbf{m}^{\dagger})$. A3 is preserved because the transformation is deterministic and bijective. In particular, for any exposure level $\mathbf{x}$, $\mathbf{M}^{\dagger}(\mathbf{x}) = \mathbf{AM}(\mathbf{x})$ is a measurable one-to-one transformation of $\mathbf{M}(\mathbf{x})$, and $\mathbf{Y}^{\dagger}(\mathbf{x},\mathbf{m}^{\dagger}) = \mathbf{Y}(\mathbf{x},\mathbf{A}^{-1}\mathbf{m}^{\dagger})$ is a re-indexing of the same potential outcome. Therefore, $\mathbf{M}(\mathbf{x})$ and $\mathbf{M}^{\dagger}(\mathbf{x})$ generate the same σ-field, and conditioning on $\mathbf{M}(\mathbf{x})$ is equivalent to conditioning on $\mathbf{M}^{\dagger}(\mathbf{x})$. So, for any $\mathbf{x},\mathbf{x}'$, and $\mathbf{m}^{\dagger}$, the collections $\{\mathbf{Y}^{\dagger}(\mathbf{x},\mathbf{m}^{\dagger}),\mathbf{M}^{\dagger}(\mathbf{x}')\} = \{\mathbf{Y}(\mathbf{x},\mathbf{A}^{-1}\mathbf{m}^{\dagger}),\mathbf{AM}(\mathbf{x}')\}$ are measurable functions of $\{\mathbf{Y}(\mathbf{x},\mathbf{m}),\mathbf{M}(\mathbf{x}')\}$. Since the σ-field generated by $\mathbf{M}(\mathbf{x})$ and $\mathbf{Z}$ and $\mathbf{M}^{\dagger}(\mathbf{x})$ and $\mathbf{Z}$ coincide, the conditional

independence relations defining sequential ignorability remain valid with $\mathbf{M}^{\dagger}$ and $\mathbf{Y}^{\dagger}$ in place of $\mathbf{M}$ and $\mathbf{Y}$. Importantly, the conditioning on $\mathbf{Z}$ is unchanged. A4 requires that the relevant conditional densities or probabilities used for identification are positive on their support, typically statement like $f_{\mathbf{X}|\mathbf{Z}}(\mathbf{x}|\mathbf{z}) > 0$ and $f_{\mathbf{M}|\mathbf{X},\mathbf{Z}}(\mathbf{m}|\mathbf{x},\mathbf{z}) > 0$ for $\mathbf{x},\mathbf{z},\mathbf{m}$. Because the mapping between $\mathbf{M}$ and $\mathbf{M}^{\dagger}$ is bijective with Jacobian determinant $|\text{det}(\mathbf{A}^{-1})| \neq 0$, the conditional law of $\mathbf{M}^{\dagger}|(\mathbf{X},\mathbf{Z})$ is absolutely continuous with respect to that of $\mathbf{M}|(\mathbf{X},\mathbf{Z})$, and positivity is preserved:

$$f_{\mathbf{M}^{\dagger}|\mathbf{X},\mathbf{Z}}(\mathbf{m}^{\dagger}|\mathbf{x},\mathbf{z}) = f_{\mathbf{M}|\mathbf{X},\mathbf{Z}}(\mathbf{A}^{-1}\mathbf{m}^{\dagger}|\mathbf{x},\mathbf{z})|\text{det}(\mathbf{A}^{-1})|.$$

Hence, $f_{\mathbf{M}^{\dagger}|\mathbf{X},\mathbf{Z}}(\mathbf{m}^{\dagger}|\mathbf{x},\mathbf{z}) > 0$ whenever $f_{\mathbf{M}|\mathbf{X},\mathbf{Z}}(\mathbf{A}^{-1}\mathbf{m}^{\dagger}|\mathbf{x},\mathbf{z}) > 0$. Positivity of $\mathbf{X}|\mathbf{Z}$ is unaffected. Therefore, A4 transfers. Hence, A1-A4 hold with $\mathbf{M}^{\dagger}$ replacing $\mathbf{M}$, and the identified ACME and ANDE are unchanged.

Proof of Proposition 4 Under the EPLSM model in (3), $\mathbf{\Gamma} \in \mathbb{R}^{p \times d}$ has full column rank $d < p$, and $\mathbf{\Gamma}_0 \in \mathbb{R}^{p \times (p-d)}$ is an orthonormal complement such that

$$\mathbf{\Gamma}^T\mathbf{\Gamma} = \mathbf{I}_d, \mathbf{\Gamma}_0^T\mathbf{\Gamma}_0 = \mathbf{I}_{p-d}, \mathbf{\Gamma}^T\mathbf{\Gamma}_0 = \mathbf{0}, \mathbf{\Gamma}\mathbf{\Gamma}^T + \mathbf{\Gamma}_0\mathbf{\Gamma}_0^T = \mathbf{I}_p.$$

Thus, $\mathbf{U} = \mathbf{\Gamma}^T\mathbf{M}$ and $\mathbf{V} = \mathbf{\Gamma}_0^T\mathbf{M}$ denote the material and immaterial part of the mediator vector $\mathbf{M}$, and for any $\mathbf{m}$, $\boldsymbol{u} = \mathbf{\Gamma}^T\mathbf{m}$ and $\boldsymbol{v} = \mathbf{\Gamma}_0^T\mathbf{m}$. The assumption $\mathbf{Y}(\mathbf{x}, \mathbf{m}) \perp\!\!\!\perp \mathbf{V}|(\mathbf{U} = \mathbf{\Gamma}^T\mathbf{m}, \mathbf{X} = \mathbf{x}, \mathbf{Z} = \mathbf{z})$ for all $\mathbf{x}, \mathbf{z}, \boldsymbol{u}, \boldsymbol{v}$ implies that, for any fixed $\mathbf{x}$, $\mathbf{z}$, and any mediator $\mathbf{m}$ with $(\boldsymbol{u}, \boldsymbol{v}) = (\mathbf{\Gamma}^T\mathbf{m}, \mathbf{\Gamma}_0^T\mathbf{m})$,

$$\begin{aligned} \mathrm{E}\{\mathbf{Y}(\mathbf{x}, \mathbf{m})|\mathbf{X} = \mathbf{x}, \mathbf{Z} = \mathbf{z}, \mathbf{M} = \mathbf{m}\} &= \mathrm{E}\{\mathbf{Y}(\mathbf{x}, \mathbf{m})|\mathbf{X} = \mathbf{x}, \mathbf{Z} = \mathbf{z}, \mathbf{U} = \boldsymbol{u}, \mathbf{V} = \boldsymbol{v}\} \\ &= \mathrm{E}\{\mathbf{Y}(\mathbf{x}, \mathbf{m})|\mathbf{X} = \mathbf{x}, \mathbf{Z} = \mathbf{z}, \mathbf{U} = \boldsymbol{u}\}. \end{aligned}$$

Define the reduced conditional mean function

$$\mathbf{Y}_{\mathbf{U}}(\mathbf{x}, \boldsymbol{u}, \mathbf{z}) = \mathrm{E}\{\mathbf{Y}(\mathbf{x}, \mathbf{M})|\mathbf{X} = \mathbf{x}, \mathbf{Z} = \mathbf{z}, \mathbf{U} = \boldsymbol{u}\}.$$

Although the potential outcome is originally defined as $\mathbf{Y}(\mathbf{x}, \mathbf{m})$, the inclusion of $\mathbf{Z}$ in $\mathbf{Y}_{\mathbf{U}}(\mathbf{x}, \boldsymbol{u}, \mathbf{z})$ reflects the conditional mean representation required for identification under sequential ignorability. The covariates $\mathbf{Z}$ are not causal arguments of the outcome, but index heterogeneity in the conditional distribution of $\mathbf{Y}(\mathbf{x}, \mathbf{m})$. Thus, writing $\mathbf{Y}_{\mathbf{U}}(\mathbf{x}, \boldsymbol{u}, \mathbf{z})$ does not introduce additional causal assumptions, but provides a convenient representation for identification. Since $\mathbf{U} = \mathbf{\Gamma}^T\mathbf{M}$ is a deterministic function of $\mathbf{M}$, we have $\mathrm{E}\{\mathbf{Y}(\mathbf{x}, \mathbf{m})|\mathbf{X} = \mathbf{x}, \mathbf{Z} = \mathbf{z}, \mathbf{M} = \mathbf{m}\} = \mathbf{Y}_{\mathbf{U}}(\mathbf{x}, \mathbf{\Gamma}^T\mathbf{m}, \mathbf{z})$ for any $\mathbf{m}$ with $\boldsymbol{u} = \mathbf{\Gamma}^T\mathbf{m}$. Now define $\mathbf{U}(\mathbf{x}) = \mathbf{\Gamma}^T\mathbf{M}(\mathbf{x})$. Using

the identification formula under A1-A4 and the above conditional mean representation, we have

$$\begin{aligned}\bar{\boldsymbol{\delta}}(\mathbf{x},\mathbf{x}') &= \mathrm{E}_{\mathbf{Z}}[\mathrm{E}\{\mathbf{Y}(\mathbf{x},\mathbf{M}(\mathbf{x})) - \mathbf{Y}(\mathbf{x},\mathbf{M}(\mathbf{x}'))|\mathbf{Z}\}] \\ &= \mathrm{E}_{\mathbf{Z}}[\mathrm{E}\{\mathbf{Y}_{\mathbf{U}}(\mathbf{x},\mathbf{U}(\mathbf{x}),\mathbf{z}) - \mathbf{Y}_{\mathbf{U}}(\mathbf{x},\mathbf{U}(\mathbf{x}'),\mathbf{z})|\mathbf{Z}\}] \equiv \bar{\boldsymbol{\delta}}_{\mathbf{U}}(\mathbf{x},\mathbf{x}')\end{aligned}$$

An analogous argument applies to the multivariate ANDE, yielding $\bar{\boldsymbol{\zeta}}(\mathbf{x},\mathbf{x}') = \bar{\boldsymbol{\zeta}}_{\mathbf{U}}(\mathbf{x},\mathbf{x}')$. Therefore, the identified ACME and ANDE computed using the reduced mediator $\mathbf{U}$ are identical to those computed using the full mediator vector $\mathbf{M}$.

Proof of Web Proposition 1 Since the EPLSM model is over-parametrized, we will apply Proposition 4.1 of Shapiro (1986) to complete the proof. Shapiro's discrepancy function in our case is $l_{\max} - l$, where l is the objective function defined in (5) and $l_{\max}$ is the maximum value of l evaluated at the standard estimator of $\mathbf{h}$. Shapiro's $\boldsymbol{\theta}$ is our $\boldsymbol{\phi}$, Shapiro's $\boldsymbol{\xi}$ is our $\mathbf{h}$, Shapiro's $\hat{\mathbf{x}}$ is the standard estimator of $\mathbf{h}$, Shapiro's $\boldsymbol{\Delta}$ is our gradient matrix $\boldsymbol{\Delta} = \partial\mathbf{h}(\boldsymbol{\phi})/\partial\boldsymbol{\phi}^T$, and Shapiro's $\mathbf{V}$ is the Fisher information matrix of the standard estimator, which we also denote by $\mathbf{V}$. It is easy to check that our discrepancy function $l_{\max} - l$ satisfies the conditions 1-4 in Section 3 of Shapiro (1986) . Since $\mathbf{V}$ is full rank,

we have $\text{rank}(\mathbf{\Delta}^T \mathbf{V} \mathbf{\Delta}) = \text{rank}(\mathbf{\Delta})$. Since the standard estimator of $\mathbf{h}$ is $\sqrt{n}$-consistent and converges in distribution to a normal distribution with mean $\mathbf{0}$ and covariance $\mathbf{V}^{-1}$, all the conditions in Proposition 4.1 of Shapiro (1986) are satisfied. Hence, the EPLSM estimator $\hat{\mathbf{h}}$ is a $\sqrt{n}$-consistent estimator of $\mathbf{h}$ and $\sqrt{n}(\hat{\mathbf{h}} - \mathbf{h})$ has asymptotically normal distributed with mean $\mathbf{0}$ and covariance matrix $\mathbf{U} = \mathbf{\Delta}(\mathbf{\Delta}^T \mathbf{V} \mathbf{\Delta})^{\dagger} \mathbf{\Delta}^T$.

Proof of Web Corollary 2 We notice that

$$\mathbf{V}^{-1} - \mathbf{U} = \mathbf{V}^{-1} - \mathbf{V}^{-1/2}[\mathbf{V}^{1/2}\mathbf{\Delta}(\mathbf{\Delta}^T \mathbf{V} \mathbf{\Delta})^{\dagger} \mathbf{\Delta}^T \mathbf{V}^{1/2}]\mathbf{V}^{-1/2} = \mathbf{V}^{-1/2}(\mathbf{I} - \mathbf{P}_{\mathbf{V}^{1/2}\mathbf{\Delta}})\mathbf{V}^{-1/2}$$

and $\mathbf{I} - \mathbf{P}_{\mathbf{V}^{1/2}\mathbf{\Delta}} \succeq 0$. Thus, $\mathbf{V}^{-1} - \mathbf{U} \succeq 0$, and the EPLSM estimator is asymptotically more efficient than or as efficient as the standard estimator.

Proof of Proposition 5 Since $\sqrt{n}(\hat{\mathbf{h}} - \mathbf{h}) \xrightarrow{d} N(\mathbf{0}, \mathbf{U})$ by Web Proposition 1, we next derive $\mathbf{\Delta}$ and $\mathbf{V}$. The normality assumption makes the explicit form of $\mathbf{V}$ available. We start with $\mathbf{V}$. Note that $\mathbf{V}$ is the Fisher information matrix. With the observed data $(\mathbf{Y}_i, \mathbf{M}_i, \mathbf{X}_i, \mathbf{Z}_i), i = 1, \ldots, n$, the log-likelihood function of

$(\mathbf{Y}, \mathbf{M})$ given $(\mathbf{X}, \mathbf{Z})$ has the following form

$$
\begin{aligned}
l = & -\frac{n(r+p)}{2}\log(2\pi) - \frac{n}{2}\log|\boldsymbol{\Sigma}_{\mathbf{M}|\mathbf{X},\mathbf{Z}}| - \frac{1}{2}\operatorname{tr}[\{\mathbb{M} - \mathbf{1}_n\boldsymbol{\mu}_{\mathbf{M}}^T - (\mathbb{X} - \mathbf{1}_n\boldsymbol{\mu}_{\mathbf{X}}^T)\boldsymbol{\gamma}_{\mathbf{X}} - (\mathbb{Z} - \mathbf{1}_n\boldsymbol{\mu}_{\mathbf{Z}}^T)\boldsymbol{\gamma}_{\mathbf{Z}}\} \\
& \times \boldsymbol{\Sigma}_{\mathbf{M}|\mathbf{X},\mathbf{Z}}^{-1}\{\mathbb{M} - \mathbf{1}_n\boldsymbol{\mu}_{\mathbf{M}}^T - (\mathbb{X} - \mathbf{1}_n\boldsymbol{\mu}_{\mathbf{X}}^T)\boldsymbol{\gamma}_{\mathbf{X}} - (\mathbb{Z} - \mathbf{1}_n\boldsymbol{\mu}_{\mathbf{Z}}^T)\boldsymbol{\gamma}_{\mathbf{Z}}\}^T] - \frac{n}{2}\log|\boldsymbol{\Sigma}_{\mathbf{Y}|\mathbf{M},\mathbf{X},\mathbf{Z}}| \\
& - \frac{1}{2}\operatorname{tr}[\{\mathbb{Y} - \mathbf{1}_n\boldsymbol{\mu}_{\mathbf{Y}}^T - (\mathbb{M} - \mathbf{1}_n\boldsymbol{\mu}_{\mathbf{M}}^T)\boldsymbol{\beta}_{\mathbf{M}} - (\mathbb{X} - \mathbf{1}_n\boldsymbol{\mu}_{\mathbf{X}}^T)\boldsymbol{\beta}_{\mathbf{X}} - (\mathbb{Z} - \mathbf{1}_n\boldsymbol{\mu}_{\mathbf{Z}}^T)\boldsymbol{\beta}_{\mathbf{Z}}\}\boldsymbol{\Sigma}_{\mathbf{Y}|\mathbf{M},\mathbf{X},\mathbf{Z}}^{-1}\{\mathbb{Y} - \mathbf{1}_n\boldsymbol{\mu}_{\mathbf{Y}}^T \\
& - (\mathbb{M} - \mathbf{1}_n\boldsymbol{\mu}_{\mathbf{M}}^T)\boldsymbol{\beta}_{\mathbf{M}} - (\mathbb{X} - \mathbf{1}_n\boldsymbol{\mu}_{\mathbf{X}}^T)\boldsymbol{\beta}_{\mathbf{X}} - (\mathbb{Z} - \mathbf{1}_n\boldsymbol{\mu}_{\mathbf{Z}}^T)\boldsymbol{\beta}_{\mathbf{Z}}\}^T],
\end{aligned}
$$

where $\mathbb{Y} = (\mathbf{Y}_1, \ldots, \mathbf{Y}_n)^T \in \mathbb{R}^{n\times r}$, $\mathbb{M} = (\mathbf{M}_1, \ldots, \mathbf{M}_n)^T \in \mathbb{R}^{n\times p}$, $\mathbb{X} = (\mathbf{X}_1, \ldots, \mathbf{X}_n)^T \in \mathbb{R}^{n\times k}$, $\mathbb{Z} = (\mathbf{Z}_1, \ldots, \mathbf{Z}_n)^T \in \mathbb{R}^{n\times q}$ are data matrices. Note that $\mathbf{V} = (1/n)\mathrm{E}(-\frac{\partial^2 l}{\partial \mathbf{h}\partial \mathbf{h}^T})$. We first compute the derivatives of l. The first derivatives are displayed as follows.

$$
\begin{aligned}
\frac{\partial l}{\partial \boldsymbol{\mu}_{\mathbf{Y}}^T} &= n\{\bar{\mathbf{Y}}^T - \boldsymbol{\mu}_{\mathbf{Y}}^T - (\bar{\mathbf{M}} - \boldsymbol{\mu}_{\mathbf{M}})^T\boldsymbol{\beta}_{\mathbf{M}} - (\bar{\mathbf{X}} - \boldsymbol{\mu}_{\mathbf{X}})^T\boldsymbol{\beta}_{\mathbf{X}} - (\bar{\mathbf{Z}} - \boldsymbol{\mu}_{\mathbf{Z}})^T\boldsymbol{\beta}_{\mathbf{Z}}\}\boldsymbol{\Sigma}_{\mathbf{Y}|\mathbf{M},\mathbf{X},\mathbf{Z}}^{-1}, \\
\frac{\partial l}{\partial \boldsymbol{\mu}_{\mathbf{M}}^T} &= n\{\bar{\mathbf{M}}^T - \boldsymbol{\mu}_{\mathbf{M}}^T - (\bar{\mathbf{X}} - \boldsymbol{\mu}_{\mathbf{X}})^T\boldsymbol{\gamma}_{\mathbf{X}} - (\bar{\mathbf{Z}} - \boldsymbol{\mu}_{\mathbf{Z}})^T\boldsymbol{\gamma}_{\mathbf{Z}}\}\boldsymbol{\Sigma}_{\mathbf{M}|\mathbf{X},\mathbf{Z}}^{-1} \\
&\quad -n\{\bar{\mathbf{Y}}^T - \boldsymbol{\mu}_{\mathbf{Y}}^T - (\bar{\mathbf{M}} - \boldsymbol{\mu}_{\mathbf{M}})^T\boldsymbol{\beta}_{\mathbf{M}} - (\bar{\mathbf{X}} - \boldsymbol{\mu}_{\mathbf{X}})^T\boldsymbol{\beta}_{\mathbf{X}} - (\bar{\mathbf{Z}} - \boldsymbol{\mu}_{\mathbf{Z}})^T\boldsymbol{\beta}_{\mathbf{Z}}\}\boldsymbol{\Sigma}_{\mathbf{Y}|\mathbf{M},\mathbf{X},\mathbf{Z}}^{-1}\boldsymbol{\beta}_{\mathbf{M}}^T, \\
\frac{\partial l}{\partial \boldsymbol{\mu}_{\mathbf{X}}^T} &= -n\{\bar{\mathbf{Y}}^T - \boldsymbol{\mu}_{\mathbf{Y}}^T - (\bar{\mathbf{M}} - \boldsymbol{\mu}_{\mathbf{M}})^T\boldsymbol{\beta}_{\mathbf{M}} - (\bar{\mathbf{X}} - \boldsymbol{\mu}_{\mathbf{X}})^T\boldsymbol{\beta}_{\mathbf{X}} - (\bar{\mathbf{Z}} - \boldsymbol{\mu}_{\mathbf{Z}})^T\boldsymbol{\beta}_{\mathbf{Z}}\}\boldsymbol{\Sigma}_{\mathbf{Y}|\mathbf{M},\mathbf{X},\mathbf{Z}}^{-1}\boldsymbol{\beta}_{\mathbf{X}}^T \\
&\quad -n\{\bar{\mathbf{M}}^T - \boldsymbol{\mu}_{\mathbf{M}}^T - (\bar{\mathbf{X}} - \boldsymbol{\mu}_{\mathbf{X}})^T\boldsymbol{\gamma}_{\mathbf{X}} - (\bar{\mathbf{Z}} - \boldsymbol{\mu}_{\mathbf{Z}})^T\boldsymbol{\gamma}_{\mathbf{Z}}\}\boldsymbol{\Sigma}_{\mathbf{M}|\mathbf{X},\mathbf{Z}}^{-1}\boldsymbol{\gamma}_{\mathbf{X}}^T, \\
\frac{\partial l}{\partial \boldsymbol{\mu}_{\mathbf{Z}}^T} &= -n\{\bar{\mathbf{Y}}^T - \boldsymbol{\mu}_{\mathbf{Y}}^T - (\bar{\mathbf{M}} - \boldsymbol{\mu}_{\mathbf{M}})^T\boldsymbol{\beta}_{\mathbf{M}} - (\bar{\mathbf{X}} - \boldsymbol{\mu}_{\mathbf{X}})^T\boldsymbol{\beta}_{\mathbf{X}} - (\bar{\mathbf{Z}} - \boldsymbol{\mu}_{\mathbf{Z}})^T\boldsymbol{\beta}_{\mathbf{Z}}\}\boldsymbol{\Sigma}_{\mathbf{Y}|\mathbf{M},\mathbf{X},\mathbf{Z}}^{-1}\boldsymbol{\beta}_{\mathbf{Z}}^T \\
&\quad -n\{\bar{\mathbf{M}}^T - \boldsymbol{\mu}_{\mathbf{M}}^T - (\bar{\mathbf{X}} - \boldsymbol{\mu}_{\mathbf{X}})^T\boldsymbol{\gamma}_{\mathbf{X}} - (\bar{\mathbf{Z}} - \boldsymbol{\mu}_{\mathbf{Z}})^T\boldsymbol{\gamma}_{\mathbf{Z}}\}\boldsymbol{\Sigma}_{\mathbf{M}|\mathbf{X},\mathbf{Z}}^{-1}\boldsymbol{\gamma}_{\mathbf{Z}}^T.
\end{aligned}
$$

Let $\tilde{\mathbf{S}}_{\mathbf{AB}} = (1/n)\sum_{i=1}^{n}(\mathbf{A}_i - \boldsymbol{\mu}_{\mathbf{A}})(\mathbf{B}_i - \boldsymbol{\mu}_{\mathbf{B}})^T$, where $\mathbf{A}$ and $\mathbf{B}$ can be taken as $\mathbf{Y}, \mathbf{M}, \mathbf{X}$ or $\mathbf{Z}$, and $\boldsymbol{\mu}_{\mathbf{A}} = \mathrm{E}(\mathrm{A}_i)$ and $\boldsymbol{\mu}_{\mathbf{B}} = \mathrm{E}(\mathbf{B}_i)$. Then,

$$
\begin{aligned}
\frac{\partial l}{\partial \boldsymbol{\beta}_{\mathbf{X}}} &= n\big(\tilde{\boldsymbol{S}}_{\mathbf{XY}} - \tilde{\boldsymbol{S}}_{\mathbf{XM}}\boldsymbol{\beta}_{\mathbf{M}} - \tilde{\boldsymbol{S}}_{\mathbf{XX}}\boldsymbol{\beta}_{\mathbf{X}} - \tilde{\boldsymbol{S}}_{\mathbf{XZ}}\boldsymbol{\beta}_{\mathbf{Z}}\big)\boldsymbol{\Sigma}^{-1}_{\mathbf{Y}|\mathbf{M},\mathbf{X},\mathbf{Z}}, \\
\frac{\partial l}{\partial \boldsymbol{\beta}_{\mathbf{Z}}} &= n\big(\tilde{\boldsymbol{S}}_{\mathbf{ZY}} - \tilde{\boldsymbol{S}}_{\mathbf{ZM}}\boldsymbol{\beta}_{\mathbf{M}} - \tilde{\boldsymbol{S}}_{\mathbf{ZX}}\boldsymbol{\beta}_{\mathbf{X}} - \tilde{\boldsymbol{S}}_{\mathbf{ZZ}}\boldsymbol{\beta}_{\mathbf{Z}}\big)\boldsymbol{\Sigma}^{-1}_{\mathbf{Y}|\mathbf{M},\mathbf{X},\mathbf{Z}}, \\
\frac{\partial l}{\partial \boldsymbol{\beta}_{\mathbf{M}}} &= n\big(\tilde{\boldsymbol{S}}_{\mathbf{MY}} - \tilde{\boldsymbol{S}}_{\mathbf{MM}}\boldsymbol{\beta}_{\mathbf{M}} - \tilde{\boldsymbol{S}}_{\mathbf{MX}}\boldsymbol{\beta}_{\mathbf{X}} - \tilde{\boldsymbol{S}}_{\mathbf{MZ}}\boldsymbol{\beta}_{\mathbf{Z}}\big)\boldsymbol{\Sigma}^{-1}_{\mathbf{Y}|\mathbf{M},\mathbf{X},\mathbf{Z}}, \\
\frac{\partial l}{\partial \boldsymbol{\gamma}_{\mathbf{X}}} &= n\big(\tilde{\boldsymbol{S}}_{\mathbf{XM}} - \tilde{\boldsymbol{S}}_{\mathbf{XX}}\boldsymbol{\gamma}_{\mathbf{X}} - \tilde{\boldsymbol{S}}_{\mathbf{XZ}}\boldsymbol{\gamma}_{\mathbf{Z}}\big)\boldsymbol{\Sigma}^{-1}_{\mathbf{M}|\mathbf{X},\mathbf{Z}}, \\
\frac{\partial l}{\partial \boldsymbol{\gamma}_{\mathbf{Z}}} &= n\big(\tilde{\boldsymbol{S}}_{\mathbf{ZM}} - \tilde{\boldsymbol{S}}_{\mathbf{ZX}}\boldsymbol{\gamma}_{\mathbf{X}} - \tilde{\boldsymbol{S}}_{\mathbf{ZZ}}\boldsymbol{\gamma}_{\mathbf{Z}}\big)\boldsymbol{\Sigma}^{-1}_{\mathbf{M}|\mathbf{X},\mathbf{Z}}, \\
\frac{\partial l}{\partial \boldsymbol{\Sigma}_{\mathbf{M}|\mathbf{X},\mathbf{Z}}} &= -\big(n\boldsymbol{\Sigma}^{-1}_{\mathbf{M}|\mathbf{X},\mathbf{Z}} - \boldsymbol{\Sigma}^{-1}_{\mathbf{M}|\mathbf{X},\mathbf{Z}}\mathbf{R}^T_{\mathbf{M}|\mathbf{X},\mathbf{Z}}\mathbf{R}_{\mathbf{M}|\mathbf{X},\mathbf{Z}}\boldsymbol{\Sigma}^{-1}_{\mathbf{M}|\mathbf{X},\mathbf{Z}}\big), \\
\frac{\partial l}{\partial \boldsymbol{\Sigma}_{\mathbf{Y}|\mathbf{M},\mathbf{X},\mathbf{Z}}} &= -\big(n\boldsymbol{\Sigma}^{-1}_{\mathbf{Y}|\mathbf{M},\mathbf{X},\mathbf{Z}} - \boldsymbol{\Sigma}^{-1}_{\mathbf{Y}|\mathbf{M},\mathbf{X},\mathbf{Z}}\mathbf{R}^T_{\mathbf{Y}|\mathbf{M},\mathbf{X},\mathbf{Z}}\mathbf{R}_{\mathbf{Y}|\mathbf{M},\mathbf{X},\mathbf{Z}}\boldsymbol{\Sigma}^{-1}_{\mathbf{Y}|\mathbf{M},\mathbf{X},\mathbf{Z}}\big),
\end{aligned}
$$

where $\mathbf{R}_{\mathbf{M}|\mathbf{X},\mathbf{Z}} = \mathbb{M} - \mathbf{1}_n\boldsymbol{\mu}^T_{\mathbf{M}} - (\mathbb{X} - \mathbf{1}_n\boldsymbol{\mu}^T_{\mathbf{X}})\boldsymbol{\gamma}_{\mathbf{X}} - (\mathbb{Z} - \mathbf{1}_n\boldsymbol{\mu}^T_{\mathbf{Z}})\boldsymbol{\gamma}_{\mathbf{Z}}$ and $\mathbf{R}_{\mathbf{Y}|\mathbf{M},\mathbf{X},\mathbf{Z}} = \mathbb{Y} - \mathbf{1}_n\boldsymbol{\mu}^T_{\mathbf{Y}} - (\mathbb{M} - \mathbf{1}_n\boldsymbol{\mu}^T_{\mathbf{M}})\boldsymbol{\beta}_{\mathbf{M}} - (\mathbb{X} - \mathbf{1}_n\boldsymbol{\mu}^T_{\mathbf{X}})\boldsymbol{\beta}_{\mathbf{X}} - (\mathbb{Z} - \mathbf{1}_n\boldsymbol{\mu}^T_{\mathbf{Z}})\boldsymbol{\beta}_{\mathbf{Z}}$. Now we take the second derivative and have

$$
\begin{aligned}
\frac{\partial^2 l}{\partial \boldsymbol{\mu}_{\mathbf{Y}}\partial \boldsymbol{\mu}^T_{\mathbf{Y}}} &= -n\boldsymbol{\Sigma}^{-1}_{\mathbf{Y}|\mathbf{M},\mathbf{X},\mathbf{Z}}, \\
\frac{\partial^2 l}{\partial \boldsymbol{\mu}_{\mathbf{M}}\partial \boldsymbol{\mu}^T_{\mathbf{Y}}} &= n\boldsymbol{\beta}_{\mathbf{M}}\boldsymbol{\Sigma}^{-1}_{\mathbf{Y}|\mathbf{M},\mathbf{X},\mathbf{Z}}, \\
\frac{\partial^2 l}{\partial \boldsymbol{\mu}_{\mathbf{X}}\partial \boldsymbol{\mu}^T_{\mathbf{Y}}} &= n\boldsymbol{\beta}_{\mathbf{X}}\boldsymbol{\Sigma}^{-1}_{\mathbf{Y}|\mathbf{M},\mathbf{X},\mathbf{Z}},
\end{aligned}
$$

$$
\begin{aligned}
\frac{\partial^2 l}{\partial \boldsymbol{\mu}_{\mathbf{Z}} \partial \boldsymbol{\mu}_{\mathbf{Y}}^T} &= n\boldsymbol{\beta}_{\mathbf{Z}} \boldsymbol{\Sigma}_{\mathbf{Y}|\mathbf{M},\mathbf{X},\mathbf{Z}}^{-1}, \\
\frac{\partial^2 l}{\partial \mathrm{vec}(\boldsymbol{\beta}_{\mathbf{M}}) \partial \boldsymbol{\mu}_{\mathbf{Y}}^T} &= -n\boldsymbol{\Sigma}_{\mathbf{Y}|\mathbf{M},\mathbf{X},\mathbf{Z}}^{-1} \otimes (\bar{\mathbf{M}} - \boldsymbol{\mu}_{\mathbf{M}}), \\
\frac{\partial^2 l}{\partial \mathrm{vec}(\boldsymbol{\beta}_{\mathbf{X}}) \partial \boldsymbol{\mu}_{\mathbf{Y}}^T} &= -n\boldsymbol{\Sigma}_{\mathbf{Y}|\mathbf{M},\mathbf{X},\mathbf{Z}}^{-1} \otimes (\bar{\mathbf{X}} - \boldsymbol{\mu}_{\mathbf{X}}), \\
\frac{\partial^2 l}{\partial \mathrm{vec}(\boldsymbol{\beta}_{\mathbf{Z}}) \partial \boldsymbol{\mu}_{\mathbf{Y}}^T} &= -n\boldsymbol{\Sigma}_{\mathbf{Y}|\mathbf{M},\mathbf{X},\mathbf{Z}}^{-1} \otimes (\bar{\mathbf{Z}} - \boldsymbol{\mu}_{\mathbf{Z}}), \\
\frac{\partial^2 l}{\partial \mathrm{vec}(\boldsymbol{\gamma}_{\mathbf{X}}) \partial \boldsymbol{\mu}_{\mathbf{Y}}^T} &= \mathbf{0}, \quad \frac{\partial^2 l}{\partial \mathrm{vec}(\boldsymbol{\gamma}_{\mathbf{Z}}) \partial \boldsymbol{\mu}_{\mathbf{Y}}^T} = \mathbf{0}, \quad \frac{\partial^2 l}{\partial \mathrm{vech}(\boldsymbol{\Sigma}_{\mathbf{M}|\mathbf{X},\mathbf{Z}}^{-1}) \partial \boldsymbol{\mu}_{\mathbf{Y}}^T} = \mathbf{0}, \\
\frac{\partial^2 l}{\partial \mathrm{vech}(\boldsymbol{\Sigma}_{\mathbf{Y}|\mathbf{M},\mathbf{X},\mathbf{Z}}^{-1}) \partial \boldsymbol{\mu}_{\mathbf{Y}}^T} &= -n\mathbf{E}_r^T (\boldsymbol{\Sigma}_{\mathbf{Y}|\mathbf{M},\mathbf{X},\mathbf{Z}}^{-1} \otimes \boldsymbol{\Sigma}_{\mathbf{Y}|\mathbf{M},\mathbf{X},\mathbf{Z}}^{-1})(\mathbf{R}_{\mathbf{Y}|\mathbf{M},\mathbf{X},\mathbf{Z}} \otimes \mathbf{I}_r), \\
\frac{\partial^2 l}{\partial \boldsymbol{\mu}_{\mathbf{X}} \partial \boldsymbol{\mu}_{\mathbf{X}}^T} &= -n\boldsymbol{\beta}_{\mathbf{X}} \boldsymbol{\Sigma}_{\mathbf{Y}|\mathbf{M},\mathbf{X},\mathbf{Z}}^{-1} \boldsymbol{\beta}_{\mathbf{X}}^T - n\boldsymbol{\gamma}_{\mathbf{X}} \boldsymbol{\Sigma}_{\mathbf{M}|\mathbf{X},\mathbf{Z}}^{-1} \boldsymbol{\gamma}_{\mathbf{X}}^T, \\
\frac{\partial^2 l}{\partial \boldsymbol{\mu}_{\mathbf{Z}} \partial \boldsymbol{\mu}_{\mathbf{X}}^T} &= -n\boldsymbol{\beta}_{\mathbf{Z}} \boldsymbol{\Sigma}_{\mathbf{Y}|\mathbf{M},\mathbf{X},\mathbf{Z}}^{-1} \boldsymbol{\beta}_{\mathbf{X}}^T - n\boldsymbol{\gamma}_{\mathbf{Z}} \boldsymbol{\Sigma}_{\mathbf{M}|\mathbf{X},\mathbf{Z}}^{-1} \boldsymbol{\gamma}_{\mathbf{X}}^T, \\
\frac{\partial^2 l}{\partial \boldsymbol{\mu}_{\mathbf{M}} \partial \boldsymbol{\mu}_{\mathbf{X}}^T} &= -n\boldsymbol{\beta}_{\mathbf{M}} \boldsymbol{\Sigma}_{\mathbf{Y}|\mathbf{M},\mathbf{X},\mathbf{Z}}^{-1} \boldsymbol{\beta}_{\mathbf{X}}^T + \boldsymbol{\Sigma}_{\mathbf{M}|\mathbf{X},\mathbf{Z}}^{-1} \boldsymbol{\gamma}_{\mathbf{X}}^T, \\
\frac{\partial^2 l}{\partial \mathrm{vec}(\boldsymbol{\beta}_{\mathbf{M}}) \partial \boldsymbol{\mu}_{\mathbf{X}}^T} &= n\boldsymbol{\Sigma}_{\mathbf{Y}|\mathbf{M},\mathbf{X},\mathbf{Z}}^{-1} \boldsymbol{\beta}_{\mathbf{X}}^T \otimes (\bar{\mathbf{M}} - \boldsymbol{\mu}_{\mathbf{M}}), \\
\frac{\partial^2 l}{\partial \mathrm{vec}(\boldsymbol{\beta}_{\mathbf{X}}) \partial \boldsymbol{\mu}_{\mathbf{X}}^T} &= n\boldsymbol{\Sigma}_{\mathbf{Y}|\mathbf{M},\mathbf{X},\mathbf{Z}}^{-1} \boldsymbol{\beta}_{\mathbf{X}}^T \otimes (\bar{\mathbf{X}} - \boldsymbol{\mu}_{\mathbf{X}}), \\
\frac{\partial^2 l}{\partial \mathrm{vec}(\boldsymbol{\beta}_{\mathbf{Z}}) \partial \boldsymbol{\mu}_{\mathbf{X}}^T} &= n\boldsymbol{\Sigma}_{\mathbf{Y}|\mathbf{M},\mathbf{X},\mathbf{Z}}^{-1} \boldsymbol{\beta}_{\mathbf{X}}^T \otimes (\bar{\mathbf{Z}} - \boldsymbol{\mu}_{\mathbf{Z}}), \\
\frac{\partial^2 l}{\partial \mathrm{vec}(\boldsymbol{\gamma}_{\mathbf{X}}) \partial \boldsymbol{\mu}_{\mathbf{X}}^T} &= n\boldsymbol{\Sigma}_{\mathbf{Y}|\mathbf{M},\mathbf{X},\mathbf{Z}}^{-1} \boldsymbol{\gamma}_{\mathbf{X}}^T \otimes (\bar{\mathbf{X}} - \boldsymbol{\mu}_{\mathbf{X}}), \\
\frac{\partial^2 l}{\partial \mathrm{vec}(\boldsymbol{\gamma}_{\mathbf{X}}) \partial^T \mathrm{vec}(\boldsymbol{\beta}_{\mathbf{Z}})} &= \mathbf{0}, \quad \frac{\partial^2 l}{\partial \mathrm{vec}(\boldsymbol{\gamma}_{\mathbf{Z}}) \partial^T \mathrm{vec}(\boldsymbol{\beta}_{\mathbf{Z}})} = \mathbf{0}, \\
\frac{\partial^2 l}{\partial \mathrm{vech}(\boldsymbol{\Sigma}_{\mathbf{Y}|\mathbf{M},\mathbf{X},\mathbf{Z}}) \partial^T \mathrm{vec}(\boldsymbol{\beta}_{\mathbf{Z}})} &= -n\mathbf{E}_r^T (\boldsymbol{\Sigma}_{\mathbf{Y}|\mathbf{M},\mathbf{X},\mathbf{Z}}^{-1} \otimes \boldsymbol{\Sigma}_{\mathbf{Y}|\mathbf{M},\mathbf{X},\mathbf{Z}}^{-1}) \{\mathbf{I}_r \otimes (\tilde{\mathbf{S}}_{\mathbf{ZY}} - \tilde{\mathbf{S}}_{\mathbf{ZM}} \boldsymbol{\beta}_{\mathbf{M}} \\
&\quad - \tilde{\mathbf{S}}_{\mathbf{ZX}} \boldsymbol{\beta}_{\mathbf{X}} - \tilde{\mathbf{S}}_{\mathbf{ZZ}} \boldsymbol{\beta}_{\mathbf{Z}})^T\},
\end{aligned}
$$

$$\frac{\partial^2 l}{\partial \text{vech}(\mathbf{\Sigma}_{\mathbf{M}|\mathbf{X},\mathbf{Z}})\partial^T \operatorname{vec}(\boldsymbol{\beta}_{\mathbf{Z}})} = \mathbf{0}$$

$$\frac{\partial^2 l}{\partial \operatorname{vec}(\boldsymbol{\beta}_{\mathbf{M}})\partial^T \operatorname{vec}(\boldsymbol{\beta}_{\mathbf{M}})} = -n\mathbf{\Sigma}^{-1}_{\mathbf{Y}|\mathbf{M},\mathbf{X},\mathbf{Z}} \otimes \tilde{\mathbf{S}}_{\mathbf{MM}}$$

$$\frac{\partial^2 l}{\partial \operatorname{vec}(\boldsymbol{\gamma}_{\mathbf{X}})\partial^T \operatorname{vec}(\boldsymbol{\beta}_{\mathbf{M}})} = \mathbf{0}$$

$$\frac{\partial^2 l}{\partial \operatorname{vec}(\boldsymbol{\gamma}_{\mathbf{Z}})\partial^T \operatorname{vec}(\boldsymbol{\beta}_{\mathbf{M}})} = \mathbf{0}$$

$$\begin{aligned}\frac{\partial^2 l}{\partial \text{vech}(\mathbf{\Sigma}_{\mathbf{Y}|\mathbf{M},\mathbf{X},\mathbf{Z}})\partial^T \operatorname{vec}(\boldsymbol{\beta}_{\mathbf{M}})} &= -n\mathbf{E}_r^T(\mathbf{\Sigma}^{-1}_{\mathbf{Y}|\mathbf{M},\mathbf{X},\mathbf{Z}} \otimes \mathbf{\Sigma}^{-1}_{\mathbf{Y}|\mathbf{M},\mathbf{X},\mathbf{Z}})\{\mathbf{I}_r \otimes (\tilde{\mathbf{S}}_{\mathbf{MY}} - \tilde{\mathbf{S}}_{\mathbf{MM}}\boldsymbol{\beta}_{\mathbf{M}} \\ &\quad -\tilde{\mathbf{S}}_{\mathbf{MX}}\boldsymbol{\beta}_{\mathbf{X}} - \tilde{\mathbf{S}}_{\mathbf{MZ}}\boldsymbol{\beta}_{\mathbf{Z}})^T\}.\end{aligned}$$

$$\frac{\partial^2 l}{\partial \text{vech}(\mathbf{\Sigma}_{\mathbf{M}|\mathbf{X},\mathbf{Z}})\partial^T \operatorname{vec}(\boldsymbol{\beta}_{\mathbf{M}})} = \mathbf{0}$$

$$\frac{\partial^2 l}{\partial \operatorname{vec}(\boldsymbol{\gamma}_{\mathbf{X}})\partial^T \operatorname{vec}(\boldsymbol{\gamma}_{\mathbf{X}})} = -n\mathbf{\Sigma}^{-1}_{\mathbf{M}|\mathbf{X},\mathbf{Z}} \otimes \tilde{\mathbf{S}}_{\mathbf{XX}}$$

$$\frac{\partial^2 l}{\partial \operatorname{vec}(\boldsymbol{\gamma}_{\mathbf{Z}})\partial^T \operatorname{vec}(\boldsymbol{\gamma}_{\mathbf{X}})} = -n\mathbf{\Sigma}^{-1}_{\mathbf{M}|\mathbf{X},\mathbf{Z}} \otimes \tilde{\mathbf{S}}_{\mathbf{ZX}}$$

$$\frac{\partial^2 l}{\partial \text{vech}(\mathbf{\Sigma}_{\mathbf{Y}|\mathbf{M},\mathbf{X},\mathbf{Z}})\partial^T \operatorname{vec}(\boldsymbol{\gamma}_{\mathbf{X}})} = \mathbf{0}$$

$$\frac{\partial^2 l}{\partial \text{vech}(\mathbf{\Sigma}_{\mathbf{M}|\mathbf{X},\mathbf{Z}})\partial^T \operatorname{vec}(\boldsymbol{\gamma}_{\mathbf{X}})} = -n\mathbf{E}_p^T(\mathbf{\Sigma}^{-1}_{\mathbf{M}|\mathbf{X},\mathbf{Z}} \otimes \mathbf{\Sigma}^{-1}_{\mathbf{M}|\mathbf{X},\mathbf{Z}})\{\mathbf{I}_p \otimes (\tilde{\mathbf{S}}_{\mathbf{XM}} - \tilde{\mathbf{S}}_{\mathbf{XX}}\boldsymbol{\gamma}_{\mathbf{X}} - \tilde{\mathbf{S}}_{\mathbf{XZ}}\boldsymbol{\gamma}_{\mathbf{Z}})^T\}$$

$$\frac{\partial^2 l}{\partial \operatorname{vec}(\boldsymbol{\gamma}_{\mathbf{Z}})\partial^T \operatorname{vec}(\boldsymbol{\gamma}_{\mathbf{Z}})} = -n\mathbf{\Sigma}^{-1}_{\mathbf{M}|\mathbf{X},\mathbf{Z}} \otimes \tilde{\mathbf{S}}_{\mathbf{ZZ}}$$

$$\frac{\partial^2 l}{\partial \text{vec}(\boldsymbol{\beta}_{\mathbf{Z}})\partial \boldsymbol{\mu}^T_{\mathbf{M}}} = n\mathbf{\Sigma}^{-1}_{\mathbf{Y}|\mathbf{M},\mathbf{X},\mathbf{Z}}\boldsymbol{\beta}^T_{\mathbf{M}} \otimes (\bar{\mathbf{Z}} - \boldsymbol{\mu}_{\mathbf{Z}})$$

$$\frac{\partial^2 l}{\partial \text{vec}(\boldsymbol{\gamma}_{\mathbf{X}})\partial \boldsymbol{\mu}^T_{\mathbf{M}}} = -n\mathbf{\Sigma}^{-1}_{\mathbf{M}|\mathbf{X},\mathbf{Z}} \otimes (\bar{\mathbf{X}} - \boldsymbol{\mu}_{\mathbf{X}})$$

$$\frac{\partial^2 l}{\partial \text{vec}(\boldsymbol{\gamma}_{\mathbf{Z}})\partial \boldsymbol{\mu}^T_{\mathbf{M}}} = -n\mathbf{\Sigma}^{-1}_{\mathbf{M}|\mathbf{X},\mathbf{Z}} \otimes (\bar{\mathbf{Z}} - \boldsymbol{\mu}_{\mathbf{Z}})$$

$$\frac{\partial^2 l}{\partial \operatorname{vech}(\mathbf{\Sigma}_{\mathbf{Y}|\mathbf{M},\mathbf{X},\mathbf{Z}})\partial \boldsymbol{\mu}^T_{\mathbf{M}}} = n\mathbf{E}_r^T(\mathbf{\Sigma}^{-1}_{\mathbf{Y}|\mathbf{M},\mathbf{X},\mathbf{Z}} \otimes \mathbf{\Sigma}^{-1}_{\mathbf{Y}|\mathbf{M},\mathbf{X},\mathbf{Z}})(\mathbf{R}_{\mathbf{Y}|\mathbf{M},\mathbf{X},\mathbf{Z}} \otimes \boldsymbol{\beta}^T_{\mathbf{M}})$$

$$
\begin{aligned}
\frac{\partial^2 l}{\partial\,\mathrm{vech}(\boldsymbol{\Sigma}_{\mathbf{M}|\mathbf{X},\mathbf{Z}})\partial\boldsymbol{\mu}_{\mathbf{M}}^T} &= -n\mathbf{E}_p^T(\boldsymbol{\Sigma}_{\mathbf{M}|\mathbf{X},\mathbf{Z}}^{-1}\otimes\boldsymbol{\Sigma}_{\mathbf{M}|\mathbf{X},\mathbf{Z}}^{-1})(\mathbf{R}_{\mathbf{M}|\mathbf{X},\mathbf{Z}}\otimes\mathbf{I}_p)\\
\frac{\partial^2 l}{\partial\,\mathrm{vec}(\boldsymbol{\beta}_{\mathbf{X}})\partial^T\,\mathrm{vec}(\boldsymbol{\beta}_{\mathbf{X}})} &= -n\boldsymbol{\Sigma}_{\mathbf{Y}|\mathbf{M},\mathbf{X},\mathbf{Z}}^{-1}\otimes\tilde{\mathbf{S}}_{\mathbf{X}\mathbf{X}}\\
\frac{\partial^2 l}{\partial\,\mathrm{vec}(\boldsymbol{\beta}_{\mathbf{Z}})\partial^T\,\mathrm{vec}(\boldsymbol{\beta}_{\mathbf{X}})} &= -n\boldsymbol{\Sigma}_{\mathbf{Y}|\mathbf{M},\mathbf{X},\mathbf{Z}}^{-1}\otimes\tilde{\mathbf{S}}_{\mathbf{Z}\mathbf{X}}\\
\frac{\partial^2 l}{\partial\,\mathrm{vec}(\boldsymbol{\beta}_{\mathbf{M}})\partial^T\,\mathrm{vec}(\boldsymbol{\beta}_{\mathbf{X}})} &= -n\boldsymbol{\Sigma}_{\mathbf{Y}|\mathbf{M},\mathbf{X},\mathbf{Z}}^{-1}\otimes\tilde{\mathbf{S}}_{\mathbf{M}\mathbf{X}}\\
\frac{\partial^2 l}{\partial\,\mathrm{vec}(\boldsymbol{\gamma}_{\mathbf{X}})\partial^T\,\mathrm{vec}(\boldsymbol{\beta}_{\mathbf{X}})} &= \mathbf{0}\\
\frac{\partial^2 l}{\partial\,\mathrm{vec}(\boldsymbol{\gamma}_{\mathbf{Z}})\partial^T\,\mathrm{vec}(\boldsymbol{\beta}_{\mathbf{X}})} &= \mathbf{0}\\
\frac{\partial^2 l}{\partial\mathrm{vech}(\boldsymbol{\Sigma}_{\mathbf{Y}|\mathbf{M},\mathbf{X},\mathbf{Z}})\partial^T\,\mathrm{vec}(\boldsymbol{\beta}_{\mathbf{X}})} &= -n\mathbf{E}_r^T(\boldsymbol{\Sigma}_{\mathbf{Y}|\mathbf{M},\mathbf{X},\mathbf{Z}}^{-1}\otimes\boldsymbol{\Sigma}_{\mathbf{Y}|\mathbf{M},\mathbf{X},\mathbf{Z}}^{-1})\{\mathbf{I}_r\otimes(\tilde{\mathbf{S}}_{\mathbf{X}\mathbf{Y}}-\tilde{\mathbf{S}}_{\mathbf{X}\mathbf{M}}\boldsymbol{\beta}_{\mathbf{M}}-\tilde{\mathbf{S}}_{\mathbf{X}\mathbf{X}}\boldsymbol{\beta}_{\mathbf{X}}\\
&\quad -\tilde{\mathbf{S}}_{\mathbf{X}\mathbf{Z}}\boldsymbol{\beta}_{\mathbf{Z}})^T\}\\
\frac{\partial^2 l}{\partial\mathrm{vech}(\boldsymbol{\Sigma}_{\mathbf{M}|\mathbf{X},\mathbf{Z}})\partial^T\,\mathrm{vec}(\boldsymbol{\beta}_{\mathbf{X}})} &= \mathbf{0}\\
\frac{\partial^2 l}{\partial\,\mathrm{vec}(\boldsymbol{\beta}_{\mathbf{Z}})\partial^T\,\mathrm{vec}(\boldsymbol{\beta}_{\mathbf{Z}})} &= -n\boldsymbol{\Sigma}_{\mathbf{Y}|\mathbf{M},\mathbf{X},\mathbf{Z}}^{-1}\otimes\tilde{\mathbf{S}}_{\mathbf{Z}\mathbf{Z}}\\
\frac{\partial^2 l}{\partial\,\mathrm{vec}(\boldsymbol{\beta}_{\mathbf{M}})\partial^T\,\mathrm{vec}(\boldsymbol{\beta}_{\mathbf{Z}})} &= -n\boldsymbol{\Sigma}_{\mathbf{Y}|\mathbf{M},\mathbf{X},\mathbf{Z}}^{-1}\otimes\tilde{\mathbf{S}}_{\mathbf{M}\mathbf{Z}}\\
\frac{\partial^2 l}{\partial\,\mathrm{vec}(\boldsymbol{\gamma}_{\mathbf{Z}})\partial\boldsymbol{\mu}_{\mathbf{X}}^T} &= n\boldsymbol{\Sigma}_{\mathbf{M}|\mathbf{X},\mathbf{Z}}^{-1}\boldsymbol{\gamma}_{\mathbf{X}}^T\otimes(\bar{\mathbf{Z}}-\boldsymbol{\mu}_{\mathbf{Z}})\\
\frac{\partial^2 l}{\partial\mathrm{vech}(\boldsymbol{\Sigma}_{\mathbf{Y}|\mathbf{M},\mathbf{X},\mathbf{Z}})\partial\boldsymbol{\mu}_{\mathbf{X}}^T} &= n\mathbf{E}_r^T(\boldsymbol{\Sigma}_{\mathbf{Y}|\mathbf{M},\mathbf{X},\mathbf{Z}}^{-1}\otimes\boldsymbol{\Sigma}_{\mathbf{Y}|\mathbf{M},\mathbf{X},\mathbf{Z}}^{-1})(\mathbf{R}_{\mathbf{Y}|\mathbf{M},\mathbf{X},\mathbf{Z}}\otimes\boldsymbol{\beta}_{\mathbf{X}}^T)\\
\frac{\partial^2 l}{\partial\mathrm{vech}(\boldsymbol{\Sigma}_{\mathbf{M}|\mathbf{X},\mathbf{Z}})\partial\boldsymbol{\mu}_{\mathbf{X}}^T} &= n\mathbf{E}_p^T(\boldsymbol{\Sigma}_{\mathbf{M}|\mathbf{X},\mathbf{Z}}^{-1}\otimes\boldsymbol{\Sigma}_{\mathbf{M}|\mathbf{X},\mathbf{Z}}^{-1})(\mathbf{R}_{\mathbf{M}|\mathbf{X},\mathbf{Z}}\otimes\boldsymbol{\gamma}_{\mathbf{X}}^T)\\
\frac{\partial^2 l}{\partial\boldsymbol{\mu}_{\mathbf{Z}}\partial\boldsymbol{\mu}_{\mathbf{Z}}^T} &= -n\boldsymbol{\beta}_{\mathbf{Z}}\boldsymbol{\Sigma}_{\mathbf{Y}|\mathbf{M},\mathbf{X},\mathbf{Z}}^{-1}\boldsymbol{\beta}_{\mathbf{Z}}^T-n\gamma_{\mathbf{Z}}\boldsymbol{\Sigma}_{\mathbf{M}|\mathbf{X},\mathbf{Z}}^{-1}\gamma_{\mathbf{Z}}^T\\
\frac{\partial^2 l}{\partial\boldsymbol{\mu}_{\mathbf{M}}\partial\boldsymbol{\mu}_{\mathbf{Z}}^T} &= -n\boldsymbol{\beta}_{\mathbf{M}}\boldsymbol{\Sigma}_{\mathbf{Y}|\mathbf{M},\mathbf{X},\mathbf{Z}}^{-1}\boldsymbol{\beta}_{\mathbf{Z}}^T+n\boldsymbol{\Sigma}_{\mathbf{M}|\mathbf{X},\mathbf{Z}}^{-1}\gamma_{\mathbf{Z}}^T\\
\frac{\partial^2 l}{\partial\,\mathrm{vec}(\boldsymbol{\beta}_{\mathbf{M}})\partial\boldsymbol{\mu}_{\mathbf{Z}}^T} &= n\boldsymbol{\Sigma}_{\mathbf{Y}|\mathbf{M},\mathbf{X},\mathbf{Z}}^{-1}\boldsymbol{\beta}_{\mathbf{Z}}^T\otimes(\bar{\mathbf{M}}-\boldsymbol{\mu}_{\mathbf{M}})
\end{aligned}
$$

$$
\begin{aligned}
\frac{\partial^2 l}{\partial\,\mathrm{vec}(\boldsymbol{\beta}_{\mathbf{X}})\partial\boldsymbol{\mu}_{\mathbf{Z}}^T} &= n\boldsymbol{\Sigma}_{\mathbf{Y}|\mathbf{M},\mathbf{X},\mathbf{Z}}^{-1}\boldsymbol{\beta}_{\mathbf{Z}}^T \otimes (\bar{\mathbf{X}} - \boldsymbol{\mu}_{\mathbf{X}}) \\
\frac{\partial^2 l}{\partial\,\mathrm{vec}(\boldsymbol{\beta}_{\mathbf{Z}})\partial\boldsymbol{\mu}_{\mathbf{Z}}^T} &= n\boldsymbol{\Sigma}_{\mathbf{Y}|\mathbf{M},\mathbf{X},\mathbf{Z}}^{-1}\boldsymbol{\beta}_{\mathbf{Z}}^T \otimes (\bar{\mathbf{Z}} - \boldsymbol{\mu}_{\mathbf{Z}}) \\
\frac{\partial^2 l}{\partial\,\mathrm{vec}(\boldsymbol{\gamma}_{\mathbf{X}})\partial\boldsymbol{\mu}_{\mathbf{Z}}^T} &= n\boldsymbol{\Sigma}_{\mathbf{M}|\mathbf{X},\mathbf{Z}}^{-1}\boldsymbol{\gamma}_{\mathbf{Z}}^T \otimes (\bar{\mathbf{X}} - \boldsymbol{\mu}_{\mathbf{X}}) \\
\frac{\partial^2 l}{\partial\,\mathrm{vec}(\boldsymbol{\gamma}_{\mathbf{Z}})\partial\boldsymbol{\mu}_{\mathbf{Z}}^T} &= n\boldsymbol{\Sigma}_{\mathbf{M}|\mathbf{X},\mathbf{Z}}^{-1}\boldsymbol{\gamma}_{\mathbf{Z}}^T \otimes (\bar{\mathbf{Z}} - \boldsymbol{\mu}_{\mathbf{Z}}) \\
\frac{\partial^2 l}{\partial \mathrm{vech}(\boldsymbol{\Sigma}_{\mathbf{Y}|\mathbf{M},\mathbf{X},\mathbf{Z}})\partial\boldsymbol{\mu}_{\mathbf{Z}}^T} &= n\mathbf{E}_r^T(\boldsymbol{\Sigma}_{\mathbf{Y}|\mathbf{M},\mathbf{X},\mathbf{Z}}^{-1} \otimes \boldsymbol{\Sigma}_{\mathbf{Y}|\mathbf{M},\mathbf{X},\mathbf{Z}}^{-1})(\mathbf{R}_{\mathbf{Y}|\mathbf{M},\mathbf{X},\mathbf{Z}} \otimes \boldsymbol{\beta}_{\mathbf{Z}}^T) \\
\frac{\partial^2 l}{\partial \mathrm{vech}(\boldsymbol{\Sigma}_{\mathbf{M}|\mathbf{X},\mathbf{Z}})\partial\boldsymbol{\mu}_{\mathbf{Z}}^T} &= n\mathbf{E}_p^T(\boldsymbol{\Sigma}_{\mathbf{M}|\mathbf{X},\mathbf{Z}}^{-1} \otimes \boldsymbol{\Sigma}_{\mathbf{M}|\mathbf{X},\mathbf{Z}}^{-1})(\mathbf{R}_{\mathbf{M}|\mathbf{X},\mathbf{Z}} \otimes \boldsymbol{\gamma}_{\mathbf{Z}}^T) \\
\frac{\partial^2 l}{\partial\boldsymbol{\mu}_{\mathbf{M}}\partial\boldsymbol{\mu}_{\mathbf{M}}^T} &= -n\boldsymbol{\Sigma}_{\mathbf{M}|\mathbf{X},\mathbf{Z}}^{-1} - n\boldsymbol{\beta}_{\mathbf{M}}\boldsymbol{\Sigma}_{\mathbf{Y}|\mathbf{M},\mathbf{X},\mathbf{Z}}^{-1}\boldsymbol{\beta}_{\mathbf{M}}^T \\
\frac{\partial^2 l}{\partial\,\mathrm{vec}(\boldsymbol{\beta}_{\mathbf{M}})\partial\boldsymbol{\mu}_{\mathbf{M}}^T} &= n\boldsymbol{\Sigma}_{\mathbf{Y}|\mathbf{M},\mathbf{X},\mathbf{Z}}^{-1}\boldsymbol{\beta}_{\mathbf{M}}^T \otimes (\bar{\mathbf{M}} - \boldsymbol{\mu}_{\mathbf{M}}) \\
\frac{\partial^2 l}{\partial\,\mathrm{vec}(\boldsymbol{\beta}_{\mathbf{X}})\partial\boldsymbol{\mu}_{\mathbf{M}}^T} &= n\boldsymbol{\Sigma}_{\mathbf{Y}|\mathbf{M},\mathbf{X},\mathbf{Z}}^{-1}\boldsymbol{\beta}_{\mathbf{M}}^T \otimes (\bar{\mathbf{X}} - \boldsymbol{\mu}_{\mathbf{X}}) \\
\frac{\partial^2 l}{\partial\,\mathrm{vech}(\boldsymbol{\Sigma}_{\mathbf{Y}|\mathbf{M},\mathbf{X},\mathbf{Z}})\partial^T\,\mathrm{vec}(\boldsymbol{\gamma}_{\mathbf{Z}})} &= \mathbf{0} \\
\frac{\partial^2 l}{\partial\,\mathrm{vech}(\boldsymbol{\Sigma}_{\mathbf{M}|\mathbf{X},\mathbf{Z}})\partial^T\,\mathrm{vec}(\boldsymbol{\gamma}_{\mathbf{Z}})} &= -n\mathbf{E}_p^T(\boldsymbol{\Sigma}_{\mathbf{M}|\mathbf{X},\mathbf{Z}}^{-1} \otimes \boldsymbol{\Sigma}_{\mathbf{M}|\mathbf{X},\mathbf{Z}}^{-1})\{\mathbf{I}_p \otimes (\tilde{\mathbf{S}}_{\mathbf{ZM}} \\
&\quad -\tilde{\mathbf{S}}_{\mathbf{ZX}}\boldsymbol{\gamma}_{\mathbf{X}} - \tilde{\mathbf{S}}_{\mathbf{ZZ}}\boldsymbol{\gamma}_{\mathbf{Z}})^T\} \\
\frac{\partial^2 l}{\partial\,\mathrm{vech}(\boldsymbol{\Sigma}_{\mathbf{Y}|\mathbf{M},\mathbf{X},\mathbf{Z}})\partial^T\,\mathrm{vech}(\boldsymbol{\Sigma}_{\mathbf{Y}|\mathbf{M},\mathbf{X},\mathbf{Z}})} &= -\frac{1}{2}\mathbf{E}_r^T(\mathbf{I}_r \otimes \boldsymbol{\Sigma}_{\mathbf{Y}|\mathbf{M},\mathbf{X},\mathbf{Z}}^{-1}\mathbf{R}_{\mathbf{Y}|\mathbf{M},\mathbf{X},\mathbf{Z}}^T\mathbf{R}_{\mathbf{Y}|\mathbf{M},\mathbf{X},\mathbf{Z}} \\
&\quad +\boldsymbol{\Sigma}_{\mathbf{Y}|\mathbf{M},\mathbf{X},\mathbf{Z}}^{-1}\mathbf{R}_{\mathbf{Y}|\mathbf{M},\mathbf{X},\mathbf{Z}}^T\mathbf{R}_{\mathbf{Y}|\mathbf{M},\mathbf{X},\mathbf{Z}} \otimes \mathbf{I}_r \\
&\quad -n\mathbf{I}_r \otimes \mathbf{I}_r)(\boldsymbol{\Sigma}_{\mathbf{Y}|\mathbf{M},\mathbf{X},\mathbf{Z}}^{-1} \otimes \boldsymbol{\Sigma}_{\mathbf{Y}|\mathbf{M},\mathbf{X},\mathbf{Z}}^{-1})\mathbf{E}_r \\
\frac{\partial^2 l}{\partial\,\mathrm{vech}(\boldsymbol{\Sigma}_{\mathbf{M}|\mathbf{X},\mathbf{Z}})\partial^T\,\mathrm{vech}(\boldsymbol{\Sigma}_{\mathbf{Y}|\mathbf{M},\mathbf{X},\mathbf{Z}})} &= \mathbf{0}
\end{aligned}
$$

$$\frac{\partial^2 l}{\partial \operatorname{vech}(\mathbf{\Sigma}_{\mathbf{M}|\mathbf{X},\mathbf{Z}})\partial^T \operatorname{vech}(\mathbf{\Sigma}_{\mathbf{M}|\mathbf{X},\mathbf{Z}})} = -\frac{1}{2}\mathbf{E}_p^T(\mathbf{I}_p \otimes \mathbf{\Sigma}_{\mathbf{M}|\mathbf{X},\mathbf{Z}}^{-1}\mathbf{R}_{\mathbf{M}|\mathbf{X},\mathbf{Z}}^T\mathbf{R}_{\mathbf{M}|\mathbf{X},\mathbf{Z}} + \mathbf{\Sigma}_{\mathbf{M}|\mathbf{X},\mathbf{Z}}^{-1}\mathbf{R}_{\mathbf{M}|\mathbf{X},\mathbf{Z}}^T\mathbf{R}_{\mathbf{M}|\mathbf{X},\mathbf{Z}} \otimes \mathbf{I}_p - n\mathbf{I}_p \otimes n\mathbf{I}_p)(\mathbf{\Sigma}_{\mathbf{M}|\mathbf{X},\mathbf{Z}}^{-1} \otimes \mathbf{\Sigma}_{\mathbf{M}|\mathbf{X},\mathbf{Z}}^{-1})\mathbf{E}_p.$$

Then, taking expectation of the negative second derivatives, we obtain the Fisher information

$$\mathbf{V} = \begin{pmatrix} \mathbf{V}_{\boldsymbol{\mu}} & \mathbf{0} & \mathbf{0} & \mathbf{0} \\ \mathbf{0} & \mathbf{V}_{\boldsymbol{\beta}} & \mathbf{0} & \mathbf{0} \\ \mathbf{0} & \mathbf{0} & \mathbf{V}_{\boldsymbol{\gamma}} & \mathbf{0} \\ \mathbf{0} & \mathbf{0} & \mathbf{0} & \mathbf{V}_{\mathbf{\Sigma}} \end{pmatrix},$$

where

$$\mathbf{V}_{\boldsymbol{\mu}} = \begin{pmatrix} \mathbf{\Sigma}_{\mathbf{Y}|\mathbf{M},\mathbf{X},\mathbf{Z}}^{-1} & -\mathbf{\Sigma}_{\mathbf{Y}|\mathbf{M},\mathbf{X},\mathbf{Z}}^{-1}\boldsymbol{\beta}_{\mathbf{M}}^T & -\mathbf{\Sigma}_{\mathbf{Y}|\mathbf{M},\mathbf{X},\mathbf{Z}}^{-1}\boldsymbol{\beta}_{\mathbf{X}}^T & -\mathbf{\Sigma}_{\mathbf{Y}|\mathbf{M},\mathbf{X},\mathbf{Z}}^{-1}\boldsymbol{\beta}_{\mathbf{Z}}^T \\ -\boldsymbol{\beta}_{\mathbf{M}}\mathbf{\Sigma}_{\mathbf{Y}|\mathbf{M},\mathbf{X},\mathbf{Z}}^{-1} & \boldsymbol{\beta}_{\mathbf{M}}\mathbf{\Sigma}_{\mathbf{Y}|\mathbf{M},\mathbf{X},\mathbf{Z}}^{-1}\boldsymbol{\beta}_{\mathbf{M}}^T + \mathbf{\Sigma}_{\mathbf{M}|\mathbf{X},\mathbf{Z}}^{-1} & \boldsymbol{\beta}_{\mathbf{M}}\mathbf{\Sigma}_{\mathbf{Y}|\mathbf{M},\mathbf{X},\mathbf{Z}}^{-1}\boldsymbol{\beta}_{\mathbf{X}}^T - \mathbf{\Sigma}_{\mathbf{M}|\mathbf{X},\mathbf{Z}}^{-1}\boldsymbol{\gamma}_{\mathbf{X}}^T & \boldsymbol{\beta}_{\mathbf{M}}\mathbf{\Sigma}_{\mathbf{Y}|\mathbf{M},\mathbf{X},\mathbf{Z}}^{-1}\boldsymbol{\beta}_{\mathbf{Z}}^T - \mathbf{\Sigma}_{\mathbf{M}|\mathbf{X},\mathbf{Z}}^{-1}\boldsymbol{\gamma}_{\mathbf{Z}}^T \\ -\boldsymbol{\beta}_{\mathbf{X}}\mathbf{\Sigma}_{\mathbf{Y}|\mathbf{M},\mathbf{X},\mathbf{Z}}^{-1} & \boldsymbol{\beta}_{\mathbf{X}}\mathbf{\Sigma}_{\mathbf{Y}|\mathbf{M},\mathbf{X},\mathbf{Z}}^{-1}\boldsymbol{\beta}_{\mathbf{M}}^T - \boldsymbol{\gamma}_{\mathbf{X}}\mathbf{\Sigma}_{\mathbf{M}|\mathbf{X},\mathbf{Z}}^{-1} & \boldsymbol{\beta}_{\mathbf{X}}\mathbf{\Sigma}_{\mathbf{Y}|\mathbf{M},\mathbf{X},\mathbf{Z}}^{-1}\boldsymbol{\beta}_{\mathbf{X}}^T + \boldsymbol{\gamma}_{\mathbf{X}}\mathbf{\Sigma}_{\mathbf{M}|\mathbf{X},\mathbf{Z}}^{-1}\boldsymbol{\gamma}_{\mathbf{X}}^T & \boldsymbol{\beta}_{\mathbf{X}}\mathbf{\Sigma}_{\mathbf{Y}|\mathbf{M},\mathbf{X},\mathbf{Z}}^{-1}\boldsymbol{\beta}_{\mathbf{Z}}^T + \boldsymbol{\gamma}_{\mathbf{X}}\mathbf{\Sigma}_{\mathbf{M}|\mathbf{X},\mathbf{Z}}^{-1}\boldsymbol{\gamma}_{\mathbf{Z}}^T \\ -\boldsymbol{\beta}_{\mathbf{Z}}\mathbf{\Sigma}_{\mathbf{Y}|\mathbf{M},\mathbf{X},\mathbf{Z}}^{-1} & \boldsymbol{\beta}_{\mathbf{Z}}\mathbf{\Sigma}_{\mathbf{Y}|\mathbf{M},\mathbf{X},\mathbf{Z}}^{-1}\boldsymbol{\beta}_{\mathbf{M}}^T - \boldsymbol{\gamma}_{\mathbf{Z}}\mathbf{\Sigma}_{\mathbf{M}|\mathbf{X},\mathbf{Z}}^{-1} & \boldsymbol{\beta}_{\mathbf{Z}}\mathbf{\Sigma}_{\mathbf{Y}|\mathbf{M},\mathbf{X},\mathbf{Z}}^{-1}\boldsymbol{\beta}_{\mathbf{X}}^T + \boldsymbol{\gamma}_{\mathbf{Z}}\mathbf{\Sigma}_{\mathbf{M}|\mathbf{X},\mathbf{Z}}^{-1}\boldsymbol{\gamma}_{\mathbf{X}}^T & \boldsymbol{\beta}_{\mathbf{Z}}\mathbf{\Sigma}_{\mathbf{Y}|\mathbf{M},\mathbf{X},\mathbf{Z}}^{-1}\boldsymbol{\beta}_{\mathbf{Z}}^T + \boldsymbol{\gamma}_{\mathbf{Z}}\mathbf{\Sigma}_{\mathbf{M}|\mathbf{X},\mathbf{Z}}^{-1}\boldsymbol{\gamma}_{\mathbf{Z}}^T \end{pmatrix}$$

$$\mathbf{V}_{\boldsymbol{\beta}} = \begin{pmatrix} \mathbf{\Sigma}_{\mathbf{Y}|\mathbf{M},\mathbf{X},\mathbf{Z}}^{-1} \otimes \mathbf{\Sigma}_{\mathbf{XX}} & \mathbf{\Sigma}_{\mathbf{Y}|\mathbf{M},\mathbf{X},\mathbf{Z}}^{-1} \otimes \mathbf{\Sigma}_{\mathbf{XZ}} & \mathbf{\Sigma}_{\mathbf{Y}|\mathbf{M},\mathbf{X},\mathbf{Z}}^{-1} \otimes \mathbf{\Sigma}_{\mathbf{XM}} \\ \mathbf{\Sigma}_{\mathbf{Y}|\mathbf{M},\mathbf{X},\mathbf{Z}}^{-1} \otimes \mathbf{\Sigma}_{\mathbf{ZX}} & \mathbf{\Sigma}_{\mathbf{Y}|\mathbf{M},\mathbf{X},\mathbf{Z}}^{-1} \otimes \mathbf{\Sigma}_{\mathbf{ZZ}} & \mathbf{\Sigma}_{\mathbf{Y}|\mathbf{M},\mathbf{X},\mathbf{Z}}^{-1} \otimes \mathbf{\Sigma}_{\mathbf{ZM}} \\ \mathbf{\Sigma}_{\mathbf{Y}|\mathbf{M},\mathbf{X},\mathbf{Z}}^{-1} \otimes \mathbf{\Sigma}_{\mathbf{MX}} & \mathbf{\Sigma}_{\mathbf{Y}|\mathbf{M},\mathbf{X},\mathbf{Z}}^{-1} \otimes \mathbf{\Sigma}_{\mathbf{MZ}} & \mathbf{\Sigma}_{\mathbf{Y}|\mathbf{M},\mathbf{X},\mathbf{Z}}^{-1} \otimes \mathbf{\Sigma}_{\mathbf{MM}} \end{pmatrix}$$

$$\mathbf{V}_{\boldsymbol{\gamma}} = \begin{pmatrix} \boldsymbol{\Sigma}^{-1}_{\mathbf{M}|\mathbf{X},\mathbf{Z}} \otimes \boldsymbol{\Sigma}_{\mathbf{XX}} & \boldsymbol{\Sigma}^{-1}_{\mathbf{M}|\mathbf{X},\mathbf{Z}} \otimes \boldsymbol{\Sigma}_{\mathbf{XZ}} \\ \boldsymbol{\Sigma}^{-1}_{\mathbf{M}|\mathbf{X},\mathbf{Z}} \otimes \boldsymbol{\Sigma}_{\mathbf{ZX}} & \boldsymbol{\Sigma}^{-1}_{\mathbf{M}|\mathbf{X},\mathbf{Z}} \otimes \boldsymbol{\Sigma}_{\mathbf{ZZ}} \end{pmatrix}$$

$$\mathbf{V}_{\boldsymbol{\Sigma}} = \begin{pmatrix} (1/2)\mathbf{E}_r^T(\boldsymbol{\Sigma}^{-1}_{\mathbf{Y}|\mathbf{M},\mathbf{X},\mathbf{Z}} \otimes \boldsymbol{\Sigma}^{-1}_{\mathbf{Y}|\mathbf{M},\mathbf{X},\mathbf{Z}})\mathbf{E}_r & \mathbf{0} \\ \mathbf{0} & (1/2)\mathbf{E}_p^T(\boldsymbol{\Sigma}^{-1}_{\mathbf{M}|\mathbf{X},\mathbf{Z}} \otimes \boldsymbol{\Sigma}^{-1}_{\mathbf{M}|\mathbf{X},\mathbf{Z}})\mathbf{E}_p \end{pmatrix},$$

where $\boldsymbol{\Sigma}_{\mathbf{UV}} = \mathrm{Cov}(\mathbf{U}, \mathbf{V})$ for $\mathbf{U}, \mathbf{V}$ taking $\mathbf{X}, \mathbf{Z}$, or $\mathbf{M}$, and we also obtain the gradient matrix

$$\boldsymbol{\Delta} = \frac{\partial \mathbf{h}(\boldsymbol{\phi})}{\partial \boldsymbol{\phi}^T} = \begin{pmatrix} \boldsymbol{\Delta}_1 & \mathbf{0} & \mathbf{0} & \mathbf{0} \\ \mathbf{0} & \boldsymbol{\Delta}_2 & \mathbf{0} & \mathbf{0} \\ \mathbf{0} & \mathbf{0} & \boldsymbol{\Delta}_3 & \mathbf{0} \\ \mathbf{0} & \boldsymbol{\Delta}_5 & \mathbf{0} & \boldsymbol{\Delta}_4 \end{pmatrix},$$

where

$$\boldsymbol{\Delta}_1 = \begin{pmatrix} \mathbf{I}_r & \mathbf{0} & \mathbf{0} & \mathbf{0} \\ \mathbf{0} & \mathbf{I}_p & \mathbf{0} & \mathbf{0} \\ \mathbf{0} & \mathbf{0} & \mathbf{I}_k & \mathbf{0} \\ \mathbf{0} & \mathbf{0} & \mathbf{0} & \mathbf{I}_q \end{pmatrix} \quad \boldsymbol{\Delta}_2 = \begin{pmatrix} \mathbf{I}_{kr} & \mathbf{0} & \mathbf{0} & \mathbf{0} \\ \mathbf{0} & \mathbf{I}_{qr} & \mathbf{0} & \mathbf{0} \\ \mathbf{0} & \mathbf{0} & \boldsymbol{\eta}^T \otimes \mathbf{I}_p & \mathbf{I}_r \otimes \boldsymbol{\Gamma} \end{pmatrix} \quad \boldsymbol{\Delta}_3 = \begin{pmatrix} \mathbf{I}_{kp} & \mathbf{0} \\ \mathbf{0} & \mathbf{I}_{qp} \end{pmatrix}$$

$$\boldsymbol{\Delta}_5 = \begin{pmatrix} \mathbf{0} & \mathbf{0} & \mathbf{0} & \mathbf{0} \\ \mathbf{0} & \mathbf{0} & 2\mathbf{C}_p(\boldsymbol{\Gamma}\boldsymbol{\Omega} \otimes \mathbf{I}_p - \boldsymbol{\Gamma} \otimes \boldsymbol{\Gamma}_0\boldsymbol{\Omega}_0\boldsymbol{\Gamma}_0^T) & \mathbf{0} \end{pmatrix}$$

$$\boldsymbol{\Delta}_4 = \begin{pmatrix} \mathbf{I}_{r(r+1)/2} & \mathbf{0} & \mathbf{0} \\ \mathbf{0} & \mathbf{C}_p(\boldsymbol{\Gamma} \otimes \boldsymbol{\Gamma})\mathbf{E}_d & \mathbf{C}_p(\boldsymbol{\Gamma}_0 \otimes \boldsymbol{\Gamma}_0)\mathbf{E}_{p-d} \end{pmatrix}.$$

We notice that because of the block structure of $\boldsymbol{\Delta}$ and $\mathbf{V}$, the asymptotic distribution of $\hat{\boldsymbol{\gamma}}_{\mathbf{X}}$ is independent of those of $\widehat{\boldsymbol{\beta}}_{\mathbf{X}}$, $\widehat{\boldsymbol{\beta}}_{\mathbf{Z}}$, and $\widehat{\boldsymbol{\beta}}_{\mathbf{M}}$. Then, we have $\sqrt{n}(\hat{\boldsymbol{\gamma}}_{\mathbf{X}} - \boldsymbol{\gamma}_{\mathbf{X}}) \xrightarrow{d} N(\mathbf{0}, \mathbf{V}_{\boldsymbol{\gamma}_{\mathbf{X}}})$, where $\mathbf{V}_{\boldsymbol{\gamma}_{\mathbf{X}}} = \{\boldsymbol{\Sigma}_{\mathbf{M}|\mathbf{X},\mathbf{Z}}^{-1} \otimes (\boldsymbol{\Sigma}_{\mathbf{XX}} - \boldsymbol{\Sigma}_{\mathbf{XZ}}\boldsymbol{\Sigma}_{\mathbf{ZZ}}^{-1}\boldsymbol{\Sigma}_{\mathbf{ZX}})\}^{-1} = \boldsymbol{\Sigma}_{\mathbf{M}|\mathbf{X},\mathbf{Z}} \otimes \boldsymbol{\Sigma}_{\mathbf{X}|\mathbf{Z}}^{-1}$. Now we derive the asymptotic distribution of $\widehat{\boldsymbol{\beta}}_{\mathbf{X}}$ and $\widehat{\boldsymbol{\beta}}_{\mathbf{M}}$. We have

$$\sqrt{n}\begin{pmatrix} \mathrm{vec}(\widehat{\boldsymbol{\beta}}_{\mathbf{X}}) - \mathrm{vec}(\boldsymbol{\beta}_{\mathbf{X}}) \\ \mathrm{vec}(\widehat{\boldsymbol{\beta}}_{\mathbf{Z}}) - \mathrm{vec}(\boldsymbol{\beta}_{\mathbf{Z}}) \\ \mathrm{vec}(\widehat{\boldsymbol{\beta}}_{\mathbf{M}}) - \mathrm{vec}(\boldsymbol{\beta}_{\mathbf{M}}) \\ \mathrm{vech}(\widehat{\boldsymbol{\Sigma}}_{\mathbf{Y}|\mathbf{M},\mathbf{X},\mathbf{Z}}) - \mathrm{vech}(\boldsymbol{\Sigma}_{\mathbf{Y}|\mathbf{M},\mathbf{X},\mathbf{Z}}) \\ \mathrm{vech}(\widehat{\boldsymbol{\Sigma}}_{\mathbf{M}|\mathbf{X},\mathbf{Z}}) - \mathrm{vech}(\boldsymbol{\Sigma}_{\mathbf{M}|\mathbf{X},\mathbf{Z}}) \end{pmatrix} \xrightarrow{d} N(0, \mathbf{V}_{\boldsymbol{\beta}\boldsymbol{\Sigma}}),$$

where

$$\mathbf{V}_{\boldsymbol{\beta}\boldsymbol{\Sigma}} = \begin{pmatrix} \boldsymbol{\Delta}_2 & \mathbf{0} \\ \boldsymbol{\Delta}_5 & \boldsymbol{\Delta}_4 \end{pmatrix} \left(\begin{pmatrix} \boldsymbol{\Delta}_2^T & \boldsymbol{\Delta}_5^T \\ \mathbf{0} & \boldsymbol{\Delta}_4^T \end{pmatrix} \begin{pmatrix} \mathbf{V}_{\boldsymbol{\beta}} & \mathbf{0} \\ \mathbf{0} & \mathbf{V}_{\boldsymbol{\Sigma}} \end{pmatrix} \begin{pmatrix} \boldsymbol{\Delta}_2 & \mathbf{0} \\ \boldsymbol{\Delta}_5 & \boldsymbol{\Delta}_4 \end{pmatrix} \right)^{\dagger} \begin{pmatrix} \boldsymbol{\Delta}_2^T & \boldsymbol{\Delta}_5^T \\ \mathbf{0} & \boldsymbol{\Delta}_4^T \end{pmatrix}.$$

Then, the asymptotic variance of $\begin{pmatrix} \mathrm{vec}(\widehat{\boldsymbol{\beta}}_{\mathbf{X}}) \\ \mathrm{vec}(\widehat{\boldsymbol{\beta}}_{\mathbf{Z}}) \\ \mathrm{vec}(\widehat{\boldsymbol{\beta}}_{\mathbf{M}}) \end{pmatrix}$ is $\boldsymbol{\Delta}_2\{\boldsymbol{\Delta}_2^T \mathbf{V}_{\boldsymbol{\beta}} \boldsymbol{\Delta}_2 + \boldsymbol{\Delta}_5^T \mathbf{V}_{\boldsymbol{\Sigma}} \boldsymbol{\Delta}_5$ - $\boldsymbol{\Delta}_5^T \mathbf{V}_{\boldsymbol{\Sigma}} \boldsymbol{\Delta}_4 \, (\boldsymbol{\Delta}_4^T \mathbf{V}_{\boldsymbol{\Sigma}} \boldsymbol{\Delta}_4)^{-1} \boldsymbol{\Delta}_4^T \mathbf{V}_{\boldsymbol{\Sigma}} \Delta_5\}^{-1} \boldsymbol{\Delta}_2^T$. After some straightforward calcu-

lations, we have $\Delta_4^T \mathbf{V}_{\boldsymbol{\Sigma}} \boldsymbol{\Delta}_5 = \mathbf{0}$, and the asymptotic variance can be simplified to $\boldsymbol{\Delta}_2 \mathbf{T}^{-1} \boldsymbol{\Delta}_2^T$, where $\mathbf{T} = \boldsymbol{\Delta}_2^T \mathbf{V}_{\boldsymbol{\beta}} \boldsymbol{\Delta}_2 + \boldsymbol{\Delta}_5^T \mathbf{V}_{\boldsymbol{\Sigma}} \boldsymbol{\Delta}_5 \equiv (\mathbf{T}_{ij}),\, i, j = 1, \ldots, 4$, $\mathbf{T}_{11} = \boldsymbol{\Sigma}_{\mathbf{Y}|\mathbf{M},\mathbf{X},\mathbf{Z}}^{-1} \otimes \boldsymbol{\Sigma}_{\mathbf{XX}}$, $\mathbf{T}_{12} = \boldsymbol{\Sigma}_{\mathbf{Y}|\mathbf{M},\mathbf{X},\mathbf{Z}}^{-1} \otimes \boldsymbol{\Sigma}_{\mathbf{XZ}}$, $\mathbf{T}_{13} = \boldsymbol{\Sigma}_{\mathbf{Y}|\mathbf{M},\mathbf{X},\mathbf{Z}}^{-1} \boldsymbol{\eta}^T \otimes \boldsymbol{\Sigma}_{\mathbf{XM}} \boldsymbol{\Gamma}_0$, $\mathbf{T}_{14} = \boldsymbol{\Sigma}_{\mathbf{Y}|\mathbf{M},\mathbf{X},\mathbf{Z}}^{-1} \otimes \boldsymbol{\Sigma}_{\mathbf{XM}} \boldsymbol{\Gamma}$ $\mathbf{T}_{21} = \boldsymbol{\Sigma}_{\mathbf{Y}|\mathbf{M},\mathbf{X},\mathbf{Z}}^{-1} \otimes \boldsymbol{\Sigma}_{\mathbf{ZX}}$, $\mathbf{T}_{22} = \boldsymbol{\Sigma}_{\mathbf{Y}|\mathbf{M},\mathbf{X},\mathbf{Z}}^{-1} \otimes \boldsymbol{\Sigma}_{\mathbf{ZZ}}$, $\mathbf{T}_{23} = \boldsymbol{\Sigma}_{\mathbf{Y}|\mathbf{M},\mathbf{X},\mathbf{Z}}^{-1} \boldsymbol{\eta}^T \otimes \boldsymbol{\Sigma}_{\mathbf{ZM}} \boldsymbol{\Gamma}_0$, $\mathbf{T}_{24} = \boldsymbol{\Sigma}_{\mathbf{Y}|\mathbf{M},\mathbf{X},\mathbf{Z}}^{-1} \otimes \boldsymbol{\Sigma}_{\mathbf{ZM}} \boldsymbol{\Gamma}$, $\mathbf{T}_{31} = \boldsymbol{\eta} \boldsymbol{\Sigma}_{\mathbf{Y}|\mathbf{M},\mathbf{X},\mathbf{Z}}^{-1} \otimes \boldsymbol{\Gamma}_0^T \boldsymbol{\Sigma}_{\mathbf{MX}}$, $\mathbf{T}_{32} = \boldsymbol{\eta} \boldsymbol{\Sigma}_{\mathbf{Y}|\mathbf{M},\mathbf{X},\mathbf{Z}}^{-1} \otimes \boldsymbol{\Gamma}_0^T \boldsymbol{\Sigma}_{\mathbf{MZ}}$, $\mathbf{T}_{33} = \boldsymbol{\eta} \boldsymbol{\Sigma}_{\mathbf{Y}|\mathbf{M},\mathbf{X},\mathbf{Z}}^{-1} \boldsymbol{\eta}^T \otimes \boldsymbol{\Gamma}_0^T \boldsymbol{\Sigma}_{\mathbf{MM}} \boldsymbol{\Gamma}_0 + \boldsymbol{\Omega} \otimes \boldsymbol{\Omega}_0^{-1} + \boldsymbol{\Omega}^{-1} \otimes \boldsymbol{\Omega}_0 - 2\mathbf{I}_d \otimes \mathbf{I}_{p-d}$, $\mathbf{T}_{34} = \boldsymbol{\eta} \boldsymbol{\Sigma}_{\mathbf{Y}|\mathbf{M},\mathbf{X},\mathbf{Z}}^{-1} \otimes \boldsymbol{\Gamma}_0^T \boldsymbol{\Sigma}_{\mathbf{MM}} \boldsymbol{\Gamma}$, $\mathbf{T}_{41} = \boldsymbol{\Sigma}_{\mathbf{Y}|\mathbf{M},\mathbf{X},\mathbf{Z}}^{-1} \otimes \boldsymbol{\Gamma}^T \boldsymbol{\Sigma}_{\mathbf{MX}}$, $\mathbf{T}_{42} = \boldsymbol{\Sigma}_{\mathbf{Y}|\mathbf{M},\mathbf{X},\mathbf{Z}}^{-1} \otimes \boldsymbol{\Gamma}^T \boldsymbol{\Sigma}_{\mathbf{MZ}}$, $\mathbf{T}_{43} = \boldsymbol{\Sigma}_{\mathbf{Y}|\mathbf{M},\mathbf{X},\mathbf{Z}}^{-1} \boldsymbol{\eta}^T \otimes \boldsymbol{\Gamma}^T \boldsymbol{\Sigma}_{\mathbf{MM}} \boldsymbol{\Gamma}_0$, $\mathbf{T}_{44} = \boldsymbol{\Sigma}_{\mathbf{Y}|\mathbf{M},\mathbf{X},\mathbf{Z}}^{-1} \otimes \boldsymbol{\Gamma}^T \boldsymbol{\Sigma}_{\mathbf{MM}} \boldsymbol{\Gamma}$. Therefore, $\sqrt{n}(\operatorname{vec}(\widehat{\boldsymbol{\beta}}_{\mathbf{X}}) - \operatorname{vec}(\boldsymbol{\beta}_{\mathbf{X}})) \xrightarrow{d} N(\mathbf{0}, \mathbf{V}_{\boldsymbol{\beta}_{\mathbf{X}}})$, where

$$
\begin{aligned}
\mathbf{V}_{\boldsymbol{\beta}_{\mathbf{X}}} &= \left\{ \mathbf{T}_{11} - \begin{pmatrix} \mathbf{T}_{12} & \mathbf{T}_{13} & \mathbf{T}_{14} \end{pmatrix} \begin{pmatrix} \mathbf{T}_{22} & \mathbf{T}_{23} & \mathbf{T}_{24} \\ \mathbf{T}_{32} & \mathbf{T}_{33} & \mathbf{T}_{34} \\ \mathbf{T}_{42} & \mathbf{T}_{43} & \mathbf{T}_{44} \end{pmatrix}^{-1} \begin{pmatrix} \mathbf{T}_{21} \\ \mathbf{T}_{31} \\ \mathbf{T}_{41} \end{pmatrix} \right\}^{-1} \\
&= \boldsymbol{\Sigma}_{\mathbf{Y}|\mathbf{M},\mathbf{X},\mathbf{Z}} \otimes (\boldsymbol{\gamma}_{\mathbf{X}} \boldsymbol{\Gamma} \boldsymbol{\Omega}^{-1} \boldsymbol{\Gamma}^T \boldsymbol{\gamma}_{\mathbf{X}}^T + \boldsymbol{\Sigma}_{\mathbf{X}|\mathbf{Z}}^{-1}) + (\boldsymbol{\eta}^T \otimes \boldsymbol{\gamma}_{\mathbf{X}} \boldsymbol{\Gamma}_0)(\boldsymbol{\eta} \boldsymbol{\Sigma}_{\mathbf{Y}|\mathbf{M},\mathbf{X},\mathbf{Z}}^{-1} \boldsymbol{\eta}^T \otimes \boldsymbol{\Omega}_0 \\
&\quad + \boldsymbol{\Omega} \otimes \boldsymbol{\Omega}_0^{-1} + \boldsymbol{\Omega}^{-1} \otimes \boldsymbol{\Omega}_0 - 2\mathbf{I}_d \otimes \mathbf{I}_{p-d})^{-1} (\boldsymbol{\eta} \otimes \boldsymbol{\Gamma}_0^T \boldsymbol{\gamma}_{\mathbf{X}}^T) \equiv \mathbf{V}_{direct},
\end{aligned}
$$

and $\sqrt{n}(\operatorname{vec}(\widehat{\boldsymbol{\beta}}_{\mathbf{M}}) - \operatorname{vec}(\boldsymbol{\beta}_{\mathbf{M}})) \xrightarrow{d} N(\mathbf{0}, \mathbf{V}_{\boldsymbol{\beta}_{\mathbf{M}}})$, where

$$
\mathbf{V}_{\boldsymbol{\beta}_{\mathbf{M}}} = \begin{pmatrix} \boldsymbol{\eta}^T \otimes \boldsymbol{\Gamma}_0 & \mathbf{I}_r \otimes \boldsymbol{\Gamma} \end{pmatrix} \left(\begin{pmatrix} \mathbf{T}_{33} & \mathbf{T}_{34} \\ \mathbf{T}_{43} & \mathbf{T}_{44} \end{pmatrix} - \begin{pmatrix} \mathbf{T}_{31} & \mathbf{T}_{32} \\ \mathbf{T}_{41} & \mathbf{T}_{42} \end{pmatrix} \begin{pmatrix} \mathbf{T}_{11} & \mathbf{T}_{12} \\ \mathbf{T}_{21} & \mathbf{T}_{22} \end{pmatrix}^{-1} \begin{pmatrix} \mathbf{T}_{13} & \mathbf{T}_{14} \\ \mathbf{T}_{23} & \mathbf{T}_{24} \end{pmatrix} \right)^{-1} \begin{pmatrix} \boldsymbol{\eta} \otimes \boldsymbol{\Gamma}_0^T \\ \mathbf{I}_r \otimes \boldsymbol{\Gamma}^T \end{pmatrix}.
$$

Since $\operatorname{vec}(\widehat{\boldsymbol{\beta}}_{\mathbf{M}})$ and $\operatorname{vec}(\hat{\boldsymbol{\gamma}}_{\mathbf{X}})$ are asymptotically independent, we have $\sqrt{n}(\operatorname{vec}(\hat{\boldsymbol{\gamma}}_{\mathbf{X}}\widehat{\boldsymbol{\beta}}_{\mathbf{M}})-\operatorname{vec}(\boldsymbol{\gamma}_{\mathbf{X}}\boldsymbol{\beta}_{\mathbf{M}})) \xrightarrow{d} N(\mathbf{0}, \mathbf{V}_{indirect})$, where

$$\begin{aligned}
\mathbf{V}_{indirect} &= \begin{pmatrix} \mathbf{I}_r \otimes \boldsymbol{\gamma}_{\mathbf{X}} & \boldsymbol{\beta}_{\mathbf{M}}^T \otimes \mathbf{I}_k \end{pmatrix} \begin{pmatrix} \mathbf{V}_{\boldsymbol{\beta}_{\mathbf{M}}} & \mathbf{0} \\ \mathbf{0} & \mathbf{V}_{\boldsymbol{\gamma}_{\mathbf{X}}} \end{pmatrix} \begin{pmatrix} \mathbf{I}_r \otimes \boldsymbol{\gamma}_{\mathbf{X}}^T \\ \boldsymbol{\beta}_{\mathbf{M}} \otimes \mathbf{I}_k \end{pmatrix} \\
&= (\mathbf{I}_r \otimes \boldsymbol{\gamma}_{\mathbf{X}})\mathbf{V}_{\boldsymbol{\beta}_{\mathbf{M}}}(\mathbf{I}_r \otimes \boldsymbol{\gamma}_{\mathbf{X}}^T) + (\boldsymbol{\beta}_{\mathbf{M}}^T \otimes \mathbf{I}_k)\mathbf{V}_{\boldsymbol{\gamma}_{\mathbf{X}}}(\boldsymbol{\beta}_{\mathbf{M}} \otimes \mathbf{I}_k). \\
&= \boldsymbol{\Sigma}_{\mathbf{Y}|\mathbf{M},\mathbf{X},\mathbf{Z}} \otimes \boldsymbol{\gamma}_{\mathbf{X}}\boldsymbol{\Gamma}\boldsymbol{\Omega}^{-1}\boldsymbol{\Gamma}^T\boldsymbol{\gamma}_{\mathbf{X}}^T + (\boldsymbol{\eta}^T \otimes \boldsymbol{\gamma}_{\mathbf{X}}\boldsymbol{\Gamma}_0)(\boldsymbol{\eta}\boldsymbol{\Sigma}_{\mathbf{Y}|\mathbf{M},\mathbf{X},\mathbf{Z}}^{-1}\boldsymbol{\eta}^T \otimes \boldsymbol{\Omega}_0 \\
&\quad + \boldsymbol{\Omega} \otimes \boldsymbol{\Omega}_0^{-1} + \boldsymbol{\Omega}^{-1} \otimes \boldsymbol{\Omega}_0 - 2\mathbf{I}_d \otimes \mathbf{I}_{p-d})^{-1}(\boldsymbol{\eta} \otimes \boldsymbol{\Gamma}_0^T\boldsymbol{\gamma}_{\mathbf{X}}^T) + \boldsymbol{\eta}^T\boldsymbol{\Omega}\boldsymbol{\eta} \otimes \boldsymbol{\Sigma}_{\mathbf{X}|\mathbf{Z}}^{-1}.
\end{aligned}$$

Proof of Theorem 6 We will show that for any small $\epsilon > 0$, there exists a sufficiently large constant C such that

$$\lim_{n\to\infty} \Pr\Big(\inf_{\substack{\boldsymbol{\Delta}\in\mathbb{R}^{(p-d)\times d}, \\ \|\boldsymbol{\Delta}\|_F = C}} f_{obj}(\mathbf{A} + n^{-1/2}\boldsymbol{\Delta}) > f_{obj}(\mathbf{A})\Big) > 1 - \epsilon. \tag{S10}$$

If (S10) holds, there exists a local minimizer $\hat{\mathbf{A}}$ of f_{obj} such that $\|\hat{\mathbf{A}} - \mathbf{A}\|_F = O_p(n^{-1/2})$. This establishes that $\hat{\mathbf{A}}$ is a $\sqrt{n}$-consistent estimator of $\mathbf{A}$. Because $\widehat{\boldsymbol{\beta}}_{\mathbf{M}} = \mathbf{P}_{\widehat{\boldsymbol{\Gamma}}(\mathbf{S}_{\mathbf{M}|\mathbf{X},\mathbf{Z}})}\mathbf{S}_{\mathbf{M}|\mathbf{X},\mathbf{Z}}^{-1}\mathbf{S}_{(\mathbf{Y},\mathbf{M})|\mathbf{X},\mathbf{Z}}$, and $\mathbf{S}_{\mathbf{M}|\mathbf{X},\mathbf{Z}}$ and $\mathbf{S}_{(\mathbf{Y},\mathbf{M})|\mathbf{X},\mathbf{Z}}$ are $\sqrt{n}$-consistent estimators of $\boldsymbol{\Sigma}_{\mathbf{M}|\mathbf{X},\mathbf{Z}}$ and $\boldsymbol{\Sigma}_{(\mathbf{Y},\mathbf{M})|\mathbf{X},\mathbf{Z}}$, respectively, then $\widehat{\boldsymbol{\beta}}_{\mathbf{M}}$ is a $\sqrt{n}$-consistent estimator of $\boldsymbol{\beta}_{\mathbf{M}}$. Since $\hat{\boldsymbol{\gamma}}_{\mathbf{X}} = \mathbf{S}_{\mathbf{X}|\mathbf{Z}}^{-1}\mathbf{S}_{(\mathbf{X},\mathbf{M})|\mathbf{Z}}$, then $\hat{\boldsymbol{\gamma}}_{\mathbf{X}}$ is a $\sqrt{n}$-consistent estimator of $\boldsymbol{\gamma}_{\mathbf{X}}$. The same logic applied to $\widehat{\boldsymbol{\beta}}_{\mathbf{X}}$. Because the indirect effect es-

timatros is $\hat{\gamma}_{\mathbf{X}}\widehat{\boldsymbol{\beta}}_{\mathbf{M}}$, the product of two $\sqrt{n}$-consistnet estimators, it is also a $\sqrt{n}$-consistent estimator.

Now, we prove (S10). We calculate $f_{obj}(\mathbf{A}+n^{-1/2}\boldsymbol{\Delta}) - f_{obj}(\mathbf{A})$ using Taylor expansion. For simplicity, we write the function f_{obj} into four parts: $f_{obj}(\mathbf{A}) \equiv f_1(\mathbf{A}) + f_2(\mathbf{A}) + f_3(\mathbf{A}) + f_4(\mathbf{A})$ where $f_1(\mathbf{A}) = -2\log|\mathbf{G}_{\mathbf{A}}^T\mathbf{G}_{\mathbf{A}}|$, $f_2(\mathbf{A}) = \log|\mathbf{G}_{\mathbf{A}}^T\mathbf{S}_{\mathbf{M}|\mathbf{X},\mathbf{Z}}^{-1}\mathbf{G}_{\mathbf{A}}|$, $f_3(\mathbf{A}) = \log|\mathbf{G}_{\mathbf{A}}^T\mathbf{S}_{\mathbf{M}|\mathbf{Y},\mathbf{X},\mathbf{Z}}\mathbf{G}_{\mathbf{A}}|$, and $f_4(\mathbf{A}) = \lambda\sum_{i=1}^{p-d} w_i\|\mathbf{a}_i\|_2$. We first expand $f_1(\mathbf{A}+n^{-1/2}\boldsymbol{\Delta})$ into $f_1(\mathbf{A}+n^{-1/2}\boldsymbol{\Delta}) = f_1(\mathbf{A}) + n^{-1/2}\overset{\rightarrow\Delta}{df_1}(\mathbf{A}) + \frac{1}{2}n^{-1}\overset{\rightarrow\Delta}{df_1^2}(\mathbf{A}) + o_p(n^{-1})$, where $\overset{\rightarrow\Delta}{df_1}(\mathbf{A})$ and $\overset{\rightarrow\Delta}{df_1^2}(\mathbf{A})$ are the first and second directional derivatives (Dattorro, 2010). The first directional derivative is

$$\overset{\rightarrow\Delta}{df_1}(\mathbf{A}) = \operatorname{tr}\left\{\left(\frac{df_1(\mathbf{A})}{d\mathbf{A}}\right)^T\boldsymbol{\Delta}\right\} = -4\operatorname{tr}[(\mathbf{I}_d + \mathbf{A}^T\mathbf{A})^{-1}\mathbf{A}^T\boldsymbol{\Delta}]$$

and the second directional derivative is

$$\begin{aligned}
\overset{\rightarrow\Delta}{df_1^2}(\mathbf{A}) &= \operatorname{tr}\left\{\left(\frac{d\{\overset{\rightarrow\Delta}{df_1}(\mathbf{A})\}}{d\mathbf{A}}\right)^T\boldsymbol{\Delta}\right\} \\
&= -4\operatorname{tr}\{[-\mathbf{A}(\mathbf{I}_d + \mathbf{A}^T\mathbf{A})^{-1}(\mathbf{A}^T\boldsymbol{\Delta} + \boldsymbol{\Delta}^T\mathbf{A})(\mathbf{I}_d + \mathbf{A}^T\mathbf{A})^{-1} + \boldsymbol{\Delta}(\mathbf{I}_d + \mathbf{A}^T\mathbf{A})^{-1}]^T\boldsymbol{\Delta}\} \\
&= 4\operatorname{tr}\{(\mathbf{I}_d + \mathbf{A}^T\mathbf{A})^{-1}(\mathbf{A}^T\boldsymbol{\Delta} + \boldsymbol{\Delta}^T\mathbf{A})(\mathbf{I}_d + \mathbf{A}^T\mathbf{A})^{-1}\mathbf{A}^T\boldsymbol{\Delta} - (\mathbf{I}_d + \mathbf{A}^T\mathbf{A})^{-1}\boldsymbol{\Delta}^T\boldsymbol{\Delta}\} \\
&= 4\operatorname{tr}\{(\mathbf{I}_d + \mathbf{A}^T\mathbf{A})^{-1}\mathbf{A}^T\boldsymbol{\Delta}(\mathbf{I}_d + \mathbf{A}^T\mathbf{A})^{-1}\mathbf{A}^T\boldsymbol{\Delta} \\
&\quad -(\mathbf{I}_d + \mathbf{A}^T\mathbf{A})^{-1}\boldsymbol{\Delta}_*^T(\mathbf{I}_p - \mathbf{G}_{\mathbf{A}}(\mathbf{G}_{\mathbf{A}}^T\mathbf{G}_{\mathbf{A}})^{-1}\mathbf{G}_{\mathbf{A}})\boldsymbol{\Delta}_*\},
\end{aligned}$$

where $\boldsymbol{\Delta}_* = \begin{pmatrix} \mathbf{0}_{d\times d} \\ \boldsymbol{\Delta} \end{pmatrix} \in \mathbb{R}^{p\times d}$. Then, $\overset{\rightarrow\Delta}{df_1^2}(\mathbf{A}) = 4\,\mathrm{tr}\{(\mathbf{I}_d + \mathbf{A}^T\mathbf{A})^{-1}\mathbf{A}^T\boldsymbol{\Delta}(\mathbf{I}_d + \mathbf{A}^T\mathbf{A})^{-1}\mathbf{A}^T\boldsymbol{\Delta} - (\mathbf{I}_d + \mathbf{A}^T\mathbf{A})^{-1}\boldsymbol{\Delta}_*^T\boldsymbol{\Gamma}_0\boldsymbol{\Gamma}_0^T\boldsymbol{\Delta}_*\}$. Substituting $\overset{\rightarrow\Delta}{df_1}(\mathbf{A})$ and $\overset{\rightarrow\Delta}{df_1^2}(\mathbf{A})$ into the expression for f_1, we obtain

$$\begin{aligned} & f_1(\mathbf{A} + n^{-1/2}\boldsymbol{\Delta}) - f_1(\mathbf{A}) \\ = \; & -4n^{-1/2}\,\mathrm{tr}[(\mathbf{I}_d + \mathbf{A}^T\mathbf{A})^{-1}\mathbf{A}^T\boldsymbol{\Delta}] + 2n^{-1}\,\mathrm{tr}\{(\mathbf{I}_d + \mathbf{A}^T\mathbf{A})^{-1}\mathbf{A}^T\boldsymbol{\Delta}(\mathbf{I}_d + \mathbf{A}^T\mathbf{A})^{-1}\mathbf{A}^T\boldsymbol{\Delta} \\ & -(\mathbf{I}_d + \mathbf{A}^T\mathbf{A})^{-1}\boldsymbol{\Delta}_*^T\boldsymbol{\Gamma}_0\boldsymbol{\Gamma}_0^T\boldsymbol{\Delta}_*\} + o_p(n^{-1}). \end{aligned}$$

Now we expand $f_2(\mathbf{A})$. The first directional derivative is

$$\overset{\rightarrow\Delta}{df_2}(\mathbf{A}) = \mathrm{tr}\left\{\left(\frac{df_2(\mathbf{A})}{d\mathbf{A}}\right)^T\boldsymbol{\Delta}\right\} = 2\,\mathrm{tr}[(\mathbf{G}_{\mathbf{A}}^T\mathbf{S}_{\mathbf{M}|\mathbf{X},\mathbf{Z}}^{-1}\mathbf{G}_{\mathbf{A}})^{-1}\mathbf{G}_{\mathbf{A}}^T\mathbf{S}_{\mathbf{M}|\mathbf{X},\mathbf{Z}}^{-1}\boldsymbol{\Delta}_*].$$

Since $\mathbf{S}_{\mathbf{M}|\mathbf{X},\mathbf{Z}}$ is a $\sqrt{n}$-consistent estimator of $\boldsymbol{\Sigma}_{\mathbf{M}|\mathbf{X},\mathbf{Z}}$, $(\mathbf{G}_{\mathbf{A}}^T\mathbf{S}_{\mathbf{M}|\mathbf{X},\mathbf{Z}}^{-1}\mathbf{G}_{\mathbf{A}})^{-1}\mathbf{G}_{\mathbf{A}}^T\mathbf{S}_{\mathbf{M}|\mathbf{X},\mathbf{Z}}^{-1}$ is a consistent estimator of $(\mathbf{G}_{\mathbf{A}}^T\boldsymbol{\Sigma}_{\mathbf{M}|\mathbf{X},\mathbf{Z}}^{-1}\mathbf{G}_{\mathbf{A}})^{-1}\mathbf{G}_{\mathbf{A}}^T\boldsymbol{\Sigma}_{\mathbf{M}|\mathbf{X},\mathbf{Z}}^{-1}$. Then, we have

$$(\mathbf{G}_{\mathbf{A}}^T\mathbf{S}_{\mathbf{M}|\mathbf{X},\mathbf{Z}}^{-1}\mathbf{G}_{\mathbf{A}})^{-1}\mathbf{G}_{\mathbf{A}}^T\mathbf{S}_{\mathbf{M}|\mathbf{X},\mathbf{Z}}^{-1} = (\mathbf{G}_{\mathbf{A}}^T\boldsymbol{\Sigma}_{\mathbf{M}|\mathbf{X},\mathbf{Z}}^{-1}\mathbf{G}_{\mathbf{A}})^{-1}\mathbf{G}_{\mathbf{A}}^T\boldsymbol{\Sigma}_{\mathbf{M}|\mathbf{X},\mathbf{Z}}^{-1} + n^{-1/2}\mathbf{T}_n + O_p(n^{-1}),$$

where $\mathrm{vec}(\mathbf{T}_n)$ converges in distribution to a normal random vector with mean $\mathbf{0}$.

Substituting the expression to $\overset{\rightarrow\Delta}{df_2}(\mathbf{A})$, we have

$$\begin{aligned}\overset{\rightarrow\Delta}{df_2}(\mathbf{A}) &= 2\,\mathrm{tr}[(\mathbf{G}_{\mathbf{A}}^T\mathbf{S}_{\mathbf{M}|\mathbf{X},\mathbf{Z}}^{-1}\mathbf{G}_{\mathbf{A}})^{-1}\mathbf{G}_{\mathbf{A}}^T\mathbf{S}_{\mathbf{M}|\mathbf{X},\mathbf{Z}}^{-1}\boldsymbol{\Delta}_*] \\ &= 2\,\mathrm{tr}[(\mathbf{G}_{\mathbf{A}}^T\boldsymbol{\Sigma}_{\mathbf{M}|\mathbf{X},\mathbf{Z}}^{-1}\mathbf{G}_{\mathbf{A}})^{-1}\mathbf{G}_{\mathbf{A}}^T\boldsymbol{\Sigma}_{\mathbf{M}|\mathbf{X},\mathbf{Z}}^{-1}\boldsymbol{\Delta}_*] + 2n^{-1/2}\,\mathrm{tr}(\mathbf{T}_n\boldsymbol{\Delta}_*) + O_p(n^{-1}).\end{aligned}$$

Since $\boldsymbol{\Sigma}_{\mathbf{M}|\mathbf{X},\mathbf{Z}}^{-1} = \boldsymbol{\Gamma}\boldsymbol{\Omega}^{-1}\boldsymbol{\Gamma}^T + \boldsymbol{\Gamma}_0\boldsymbol{\Omega}_0^{-1}\boldsymbol{\Gamma}_0^T$ and $\boldsymbol{\Gamma} = \mathbf{G}_{\mathbf{A}}\boldsymbol{\Gamma}_1$, we have $(\mathbf{G}_{\mathbf{A}}^T\boldsymbol{\Sigma}_{\mathbf{M}|\mathbf{X},\mathbf{Z}}^{-1}\mathbf{G}_{\mathbf{A}})^{-1}\mathbf{G}_{\mathbf{A}}^T\boldsymbol{\Sigma}_{\mathbf{M}|\mathbf{X},\mathbf{Z}}^{-1} = \boldsymbol{\Gamma}_1(\boldsymbol{\Gamma}^T\boldsymbol{\Sigma}_{\mathbf{M}|\mathbf{X},\mathbf{Z}}^{-1}\boldsymbol{\Gamma})^{-1}\boldsymbol{\Gamma}_1^T\boldsymbol{\Gamma}_1^{-T}\boldsymbol{\Gamma}^T\boldsymbol{\Sigma}_{\mathbf{M}|\mathbf{X},\mathbf{Z}}^{-1} = \boldsymbol{\Gamma}_1\boldsymbol{\Gamma}_1^T\mathbf{G}_{\mathbf{A}}^T = (\mathbf{I}_d + \mathbf{A}^T\mathbf{A})^{-1}\mathbf{G}_{\mathbf{A}}^T$. The last equality is because $\mathbf{I}_d = \boldsymbol{\Gamma}^T\boldsymbol{\Gamma} = \boldsymbol{\Gamma}_1^T\mathbf{G}_{\mathbf{A}}^T\mathbf{G}_{\mathbf{A}}\boldsymbol{\Gamma}_1$. This gives $\mathbf{G}_{\mathbf{A}}^T\mathbf{G}_{\mathbf{A}} = \boldsymbol{\Gamma}_1^{-T}\boldsymbol{\Gamma}_1^{-1}$. Then,

$$\boldsymbol{\Gamma}_1\boldsymbol{\Gamma}_1^T = (\mathbf{G}_{\mathbf{A}}^T\mathbf{G}_{\mathbf{A}})^{-1} = (\mathbf{I}_d + \mathbf{A}^T\mathbf{A})^{-1}. \tag{S11}$$

So, the first directional derivative is $\overset{\rightarrow\Delta}{df_2}(\mathbf{A}) = 2\,\mathrm{tr}[(\mathbf{I}_d + \mathbf{A}^T\mathbf{A})^{-1}\mathbf{A}^T\boldsymbol{\Delta}] + 2n^{-1/2}\,\mathrm{tr}(\mathbf{T}_n\boldsymbol{\Delta}_*) + O_p(n^{-1})$. By Cauchy-Schwartz inequality (Harville, 1998), $|\,\mathrm{tr}(\mathbf{T}_n\boldsymbol{\Delta}_*)| \leq \|\boldsymbol{\Delta}\|_F\|\mathbf{T}_n\|_F$. The second directional derivative of f_2 is

$$\begin{aligned}\overset{\rightarrow\Delta}{df_2^2}(\mathbf{A}) &= \mathrm{tr}\left\{\left(\frac{d\{\overset{\rightarrow\Delta}{df_2}(\mathbf{A})\}}{d\mathbf{A}}\right)^T\boldsymbol{\Delta}\right\} \\ &= 2\,\mathrm{tr}\{(\mathbf{G}_{\mathbf{A}}^T\mathbf{S}_{\mathbf{M}|\mathbf{X},\mathbf{Z}}^{-1}\mathbf{G}_{\mathbf{A}})^{-1}\boldsymbol{\Delta}_*^T\mathbf{S}_{\mathbf{M}|\mathbf{X},\mathbf{Z}}^{-1}\boldsymbol{\Delta}_* - (\mathbf{G}_{\mathbf{A}}^T\mathbf{S}_{\mathbf{M}|\mathbf{X},\mathbf{Z}}^{-1}\mathbf{G}_{\mathbf{A}})^{-1}(\mathbf{G}_{\mathbf{A}}^T\mathbf{S}_{\mathbf{M}|\mathbf{X},\mathbf{Z}}^{-1}\boldsymbol{\Delta}_* \\ &\quad + \boldsymbol{\Delta}_*^T\mathbf{S}_{\mathbf{M}|\mathbf{X},\mathbf{Z}}^{-1}\mathbf{G}_{\mathbf{A}})(\mathbf{G}_{\mathbf{A}}^T\mathbf{S}_{\mathbf{M}|\mathbf{X},\mathbf{Z}}^{-1}\mathbf{G}_{\mathbf{A}})^{-1}\mathbf{G}_{\mathbf{A}}^T\mathbf{S}_{\mathbf{M}|\mathbf{X},\mathbf{Z}}^{-1}\boldsymbol{\Delta}_*\}.\end{aligned}$$

Since $\mathbf{S}_{\mathbf{M}|\mathbf{X},\mathbf{Z}}^{-1} = \boldsymbol{\Sigma}_{\mathbf{M}|\mathbf{X},\mathbf{Z}}^{-1} + o_p(1)$, after some straightforward algebra, we have

$\overset{\rightarrow\Delta}{df_2^2}(\mathbf{A}) = 2\,\mathrm{tr}\{\boldsymbol{\Omega}\boldsymbol{\Gamma}_1^T\boldsymbol{\Delta}_*^T\boldsymbol{\Gamma}_0\boldsymbol{\Omega}_0^{-1}\boldsymbol{\Gamma}_0^T\boldsymbol{\Delta}_*\boldsymbol{\Gamma}_1 - (\mathbf{I}_d + \mathbf{A}^T\mathbf{A})^{-1}\mathbf{A}^T\boldsymbol{\Delta}(\mathbf{I}_d + \mathbf{A}^T\mathbf{A})^{-1}\mathbf{A}^T\boldsymbol{\Delta}\} + o_p(1)$. Substituting $\overset{\rightarrow\Delta}{df_2}(\mathbf{A})$ and $\overset{\rightarrow\Delta}{df_2^2}(\mathbf{A})$ into the expression for f_2, we get

$$
\begin{aligned}
& f_2(\mathbf{A} + n^{-1/2}\boldsymbol{\Delta}) - f_2(\mathbf{A}) \\
= \;& 2n^{-1/2}\,\mathrm{tr}\{(\mathbf{I}_d + \mathbf{A}^T\mathbf{A})^{-1}\mathbf{A}^T\boldsymbol{\Delta}\} + 2n^{-1}\,\mathrm{tr}(\mathbf{T}_n\boldsymbol{\Delta}_*) + n^{-1}\,\mathrm{tr}\{\boldsymbol{\Omega}\boldsymbol{\Gamma}_1^T\boldsymbol{\Delta}_*^T\boldsymbol{\Gamma}_0\boldsymbol{\Omega}_0^{-1}\boldsymbol{\Gamma}_0^T\boldsymbol{\Delta}_*\boldsymbol{\Gamma}_1 \\
& -(\mathbf{I}_d + \mathbf{A}^T\mathbf{A})^{-1}\mathbf{A}^T\boldsymbol{\Delta}(\mathbf{I}_d + \mathbf{A}^T\mathbf{A})^{-1}\mathbf{A}^T\boldsymbol{\Delta}\} + o_p(n^{-1}) \\
\geq \;& 2n^{-1/2}\,\mathrm{tr}\{(\mathbf{I}_d + \mathbf{A}^T\mathbf{A})^{-1}\mathbf{A}^T\boldsymbol{\Delta}\} - 2n^{-1}\|\boldsymbol{\Delta}\|_F\|\mathbf{T}_n\|_F + n^{-1}\,\mathrm{tr}\{\boldsymbol{\Omega}\boldsymbol{\Gamma}_1^T\boldsymbol{\Delta}_*^T\boldsymbol{\Gamma}_0\boldsymbol{\Omega}_0^{-1}\boldsymbol{\Gamma}_0^T\boldsymbol{\Delta}_*\boldsymbol{\Gamma}_1 \\
& -(\mathbf{I}_d + \mathbf{A}^T\mathbf{A})^{-1}\mathbf{A}^T\boldsymbol{\Delta}(\mathbf{I}_d + \mathbf{A}^T\mathbf{A})^{-1}\mathbf{A}^T\boldsymbol{\Delta}\} + o_p(n^{-1}).
\end{aligned}
$$

Notice that f_3 ans f_2 have the same structure except that $\mathbf{S}_{\mathbf{M}|\mathbf{X},\mathbf{Z}}^{-1}$ is replaced by $\mathbf{S}_{\mathbf{M}|\mathbf{Y},\mathbf{X},\mathbf{Z}}$. Because $\mathbf{S}_{\mathbf{M}|\mathbf{Y},\mathbf{X},\mathbf{Z}}$ is a $\sqrt{n}$-consistent estimator of $\boldsymbol{\Sigma}_{\mathbf{M}|\mathbf{Y},\mathbf{X},\mathbf{Z}} = \boldsymbol{\Gamma}\boldsymbol{\Omega}\boldsymbol{\Gamma}^T + \boldsymbol{\Gamma}_0\boldsymbol{\Omega}_0\boldsymbol{\Gamma}_0^T - \boldsymbol{\Gamma}\boldsymbol{\Omega}\boldsymbol{\eta}\boldsymbol{\Sigma}_{\mathbf{Y}|\mathbf{X},\mathbf{Z}}^{-1}\boldsymbol{\eta}^T\boldsymbol{\Omega}\boldsymbol{\Gamma}^T$, $(\mathbf{G}_{\mathbf{A}}^T\mathbf{S}_{\mathbf{M}|\mathbf{Y},\mathbf{X},\mathbf{Z}}\mathbf{G}_{\mathbf{A}})^{-1}\mathbf{G}_{\mathbf{A}}^T\mathbf{S}_{\mathbf{M}|\mathbf{Y},\mathbf{X},\mathbf{Z}}$ is a $\sqrt{n}$-consistent estimator of $(\mathbf{G}_{\mathbf{A}}^T\boldsymbol{\Sigma}_{\mathbf{M}|\mathbf{Y},\mathbf{X},\mathbf{Z}}\mathbf{G}_{\mathbf{A}})^{-1}\mathbf{G}_{\mathbf{A}}^T\boldsymbol{\Sigma}_{\mathbf{M}|\mathbf{Y},\mathbf{X},\mathbf{Z}}$. Then we have $(\mathbf{G}_{\mathbf{A}}^T\mathbf{S}_{\mathbf{M}|\mathbf{Y},\mathbf{X},\mathbf{Z}}\mathbf{G}_{\mathbf{A}})^{-1}\mathbf{G}_{\mathbf{A}}^T\mathbf{S}_{\mathbf{M}|\mathbf{Y},\mathbf{X},\mathbf{Z}} = (\mathbf{G}_{\mathbf{A}}^T\boldsymbol{\Sigma}_{\mathbf{M}|\mathbf{Y},\mathbf{X},\mathbf{Z}}\mathbf{G}_{\mathbf{A}})^{-1}\mathbf{G}_{\mathbf{A}}^T\boldsymbol{\Sigma}_{\mathbf{M}|\mathbf{Y},\mathbf{X},\mathbf{Z}} + n^{-1/2}\mathbf{W}_n + O_p(n^{-1})$, where $\mathrm{vec}(\mathbf{W}_n)$ converges in distribution to a normal random vector

with mean $\mathbf{0}$. With the same expansion to f_3, we get

$$
\begin{aligned}
& f_3(\mathbf{A}+n^{-1/2}\boldsymbol{\Delta}) - f_3(\mathbf{A}) \\
= \; & 2n^{-1/2}\operatorname{tr}\{(\mathbf{I}_d+\mathbf{A}^T\mathbf{A})^{-1}\mathbf{A}^T\boldsymbol{\Delta}\} + 2n^{-1}\operatorname{tr}(\mathbf{W}_n\boldsymbol{\Delta}_*) + n^{-1}\operatorname{tr}\{\boldsymbol{\Omega}\boldsymbol{\Gamma}_1^T\boldsymbol{\Delta}_*^T\boldsymbol{\Gamma}_0\boldsymbol{\Omega}_0^{-1}\boldsymbol{\Gamma}_0^T\boldsymbol{\Delta}_*\boldsymbol{\Gamma}_1 \\
& -(\mathbf{I}_d+\mathbf{A}^T\mathbf{A})^{-1}\mathbf{A}^T\boldsymbol{\Delta}(\mathbf{I}_d+\mathbf{A}^T\mathbf{A})^{-1}\mathbf{A}^T\boldsymbol{\Delta}\} + o_p(n^{-1}) \\
\geq \; & 2n^{-1/2}\operatorname{tr}\{(\mathbf{I}_d+\mathbf{A}^T\mathbf{A})^{-1}\mathbf{A}^T\boldsymbol{\Delta}\} - 2n^{-1}\|\boldsymbol{\Delta}\|_F\|\mathbf{W}_n\|_F \\
& +n^{-1}\operatorname{tr}\{(\boldsymbol{\Omega}-\boldsymbol{\Omega}\boldsymbol{\eta}\boldsymbol{\Sigma}_{\mathbf{Y}|\mathbf{X},\mathbf{Z}}^{-1}\boldsymbol{\eta}^T\boldsymbol{\Omega})^{-1}\boldsymbol{\Gamma}_1^T\boldsymbol{\Delta}_*^T\boldsymbol{\Gamma}_0\boldsymbol{\Omega}_0\boldsymbol{\Gamma}_0^T\boldsymbol{\Delta}_*\boldsymbol{\Gamma}_1 \\
& -(\mathbf{I}_d+\mathbf{A}^T\mathbf{A})^{-1}\mathbf{A}^T\boldsymbol{\Delta}(\mathbf{I}_d+\mathbf{A}^T\mathbf{A})^{-1}\mathbf{A}^T\boldsymbol{\Delta}\} + o_p(n^{-1}).
\end{aligned}
$$

By Woodbury matrix identity, we have $(\boldsymbol{\Omega}-\boldsymbol{\Omega}\boldsymbol{\eta}\boldsymbol{\Sigma}_{\mathbf{Y}|\mathbf{X},\mathbf{Z}}^{-1}\boldsymbol{\eta}^T\boldsymbol{\Omega})^{-1} = \boldsymbol{\Omega}^{-1}+\boldsymbol{\eta}(\boldsymbol{\Sigma}_{\mathbf{Y}|\mathbf{X},\mathbf{Z}}- \boldsymbol{\eta}^T\boldsymbol{\Omega}\boldsymbol{\eta})^{-1}\boldsymbol{\eta}^T = \boldsymbol{\Omega}^{-1}+\boldsymbol{\eta}\boldsymbol{\Sigma}_{\mathbf{Y}|\mathbf{M},\mathbf{X},\mathbf{Z}}^{-1}\boldsymbol{\eta}^T$. Thus,

$$
\begin{aligned}
& f_3(\mathbf{A}+n^{-1/2}\boldsymbol{\Delta}) - f_3(\mathbf{A}) \\
\geq \; & 2n^{-1/2}\operatorname{tr}\{(\mathbf{I}_d+\mathbf{A}^T\mathbf{A})^{-1}\mathbf{A}^T\boldsymbol{\Delta}\} - 2n^{-1}\|\boldsymbol{\Delta}\|_F\|\mathbf{W}_n\|_F + n^{-1}\operatorname{tr}\{(\boldsymbol{\Omega}^{-1} \\
& +\boldsymbol{\eta}\boldsymbol{\Sigma}_{\mathbf{Y}|\mathbf{M},\mathbf{X},\mathbf{Z}}^{-1}\boldsymbol{\eta}^T)\boldsymbol{\Gamma}_1^T\boldsymbol{\Delta}_*^T\boldsymbol{\Gamma}_0\boldsymbol{\Omega}_0\boldsymbol{\Gamma}_0^T\boldsymbol{\Delta}_*\boldsymbol{\Gamma}_1 - (\mathbf{I}_d+\mathbf{A}^T\mathbf{A})^{-1}\mathbf{A}^T\boldsymbol{\Delta}(\mathbf{I}_d+\mathbf{A}^T\mathbf{A})^{-1}\mathbf{A}^T\boldsymbol{\Delta}\} + o_p(n^{-1}).
\end{aligned}
$$

Now we expand $f_4(\mathbf{A})$. Let $\boldsymbol{\delta}_i^T$ be the ith row of $\boldsymbol{\Delta}$. Then,

$$
\begin{aligned}
f_4(\mathbf{A}+n^{-1/2}\boldsymbol{\Delta}) - f_4(\mathbf{A}) &= \sum_{i=1}^{p-d}\{\lambda w_i\|\mathbf{a}_i+n^{-1/2}\boldsymbol{\delta}_i\|_2 - \lambda w_i\|\mathbf{a}_i\|_2\} \\
&\geq \sum_{i=1}^{p_{\mathcal{A}}-d}\{\lambda w_i\|\mathbf{a}_i+n^{-1/2}\boldsymbol{\delta}_i\|_2 - \lambda w_i\|\mathbf{a}_i\|_2\} \geq -(p_{\mathcal{A}}-d)n^{-1/2}\lambda_{\mathcal{A}}\max_{1\leq i\leq p_{\mathcal{A}-d}}\|\boldsymbol{\delta}_i\|_2.
\end{aligned}
$$

The third inequality is based on the triangular inequality that $-\| - n^{-1/2}\boldsymbol{\delta}_i\|_2 \leq \|\mathbf{a}_i + n^{-1/2}\boldsymbol{\delta}_i\|_2 - \|\mathbf{a}_i\|_2$. As $\sqrt{n}\lambda_{\mathcal{A}} \to 0$, $f_4(\mathbf{A} + n^{-1/2}\boldsymbol{\Delta}) - f_4(\mathbf{A}) = o_p(n^{-1})$. Combining the results for f_1, f_2, f_3, and f_4, we have

$$
\begin{aligned}
&f_{obj}(\mathbf{A} + n^{-1/2}\boldsymbol{\Delta}) - f_{obj}(\mathbf{A}) \\
&\geq \; -2n^{-1}\|\boldsymbol{\Delta}\|_F\|\mathbf{T}_n\|_F - 2n^{-1}\|\boldsymbol{\Delta}\|_F\|\mathbf{W}_n\|_F + n^{-1}\,\mathrm{tr}\{\boldsymbol{\Omega}\boldsymbol{\Gamma}_1^T\boldsymbol{\Delta}_*^T\boldsymbol{\Gamma}_0\boldsymbol{\Omega}_0^{-1}\boldsymbol{\Gamma}_0^T\boldsymbol{\Delta}_*\boldsymbol{\Gamma}_1 \\
&\quad +(\boldsymbol{\Omega}^{-1} + \boldsymbol{\eta}\boldsymbol{\Sigma}_{\mathbf{Y}|\mathbf{M},\mathbf{X},\mathbf{Z}}^{-1}\boldsymbol{\eta}^T)\boldsymbol{\Gamma}_1^T\boldsymbol{\Delta}_*^T\boldsymbol{\Gamma}_0\boldsymbol{\Omega}_0\boldsymbol{\Gamma}_0^T\boldsymbol{\Delta}_*\boldsymbol{\Gamma}_1 - 2(\mathbf{I}_d + \mathbf{A}^T\mathbf{A})^{-1}\boldsymbol{\Delta}_*^T\boldsymbol{\Gamma}_0\boldsymbol{\Gamma}_0^T\boldsymbol{\Delta}_*\} \\
&\quad -n^{-1}(p_{\mathcal{A}} - d)\sqrt{n}\lambda_{\mathcal{A}} \max_{1\leq i\leq p_{\mathcal{A}}-d}\|\boldsymbol{\delta}_i\|_2 + o_p(n^{-1}).
\end{aligned}
$$

Let $\mathrm{M} = (\boldsymbol{\Omega}^{-1} + \boldsymbol{\eta}\boldsymbol{\Sigma}_{\mathbf{Y}|\mathbf{M},\mathbf{X},\mathbf{Z}}^{-1}\boldsymbol{\eta}^T) \otimes \boldsymbol{\Omega}_0 + \boldsymbol{\Omega} \otimes \boldsymbol{\Omega}_0^{-1} - 2\mathbf{I}_d \otimes \mathbf{I}_{p-d}$ and let m_1 be the smallest eigenvalue of M. The matrix M appears in Proposition 5 in the asymptotic variance of indirect effect. By Shapiro (1986), M is a positive definite matrix and $m_1 > 0$. Then we have

$$
\begin{aligned}
&\mathrm{tr}\{(\boldsymbol{\Omega}^{-1} + \boldsymbol{\eta}\boldsymbol{\Sigma}_{\mathbf{Y}|\mathbf{M},\mathbf{X},\mathbf{Z}}^{-1}\boldsymbol{\eta}^T)\boldsymbol{\Gamma}_1^T\boldsymbol{\Delta}_*^T\boldsymbol{\Gamma}_0\boldsymbol{\Omega}_0\boldsymbol{\Gamma}_0^T\boldsymbol{\Delta}_*\boldsymbol{\Gamma}_1 + \boldsymbol{\Omega}\boldsymbol{\Gamma}_1^T\boldsymbol{\Delta}_*^T\boldsymbol{\Gamma}_0\boldsymbol{\Omega}_0^{-1}\boldsymbol{\Gamma}_0^T\boldsymbol{\Delta}_*\boldsymbol{\Gamma}_1 \\
&\quad -2(\mathbf{I}_d + \mathbf{A}^T\mathbf{A})^{-1}\boldsymbol{\Delta}_*^T\boldsymbol{\Gamma}_0\boldsymbol{\Gamma}_0^T\boldsymbol{\Delta}_*^T\} \\
&= \; \mathrm{tr}\{(\boldsymbol{\Omega}^{-1} + \boldsymbol{\eta}\boldsymbol{\Sigma}_{\mathbf{Y}|\mathbf{M},\mathbf{X},\mathbf{Z}}^{-1}\boldsymbol{\eta}^T)\boldsymbol{\Gamma}_1^T\boldsymbol{\Delta}_*^T\boldsymbol{\Gamma}_0\boldsymbol{\Omega}_0\boldsymbol{\Gamma}_0^T\boldsymbol{\Delta}_*\boldsymbol{\Gamma}_1 + \boldsymbol{\Omega}\boldsymbol{\Gamma}_1^T\boldsymbol{\Delta}_*^T\boldsymbol{\Gamma}_0\boldsymbol{\Omega}_0^{-1}\boldsymbol{\Gamma}_0^T\boldsymbol{\Delta}_*\boldsymbol{\Gamma}_1 \\
&\quad -2\boldsymbol{\Gamma}_1^T\boldsymbol{\Delta}_*^T\boldsymbol{\Gamma}_0\boldsymbol{\Gamma}_0^T\boldsymbol{\Delta}_*\boldsymbol{\Gamma}_1\}
\end{aligned}
$$

$$
\begin{aligned}
&= \operatorname{vec}(\boldsymbol{\Gamma}_0^T \boldsymbol{\Delta}_*^T \boldsymbol{\Gamma}_1)^T \mathbf{M} \operatorname{vec}(\boldsymbol{\Gamma}_0^T \boldsymbol{\Delta}_* \boldsymbol{\Gamma}_1) \geq m_1 \|\boldsymbol{\Gamma}_0^T \boldsymbol{\Delta}_* \boldsymbol{\Gamma}_1\|_F^2 = m_1 \operatorname{tr}(\boldsymbol{\Gamma}_0 \boldsymbol{\Gamma}_0^T \boldsymbol{\Delta}_* \boldsymbol{\Gamma}_1 \boldsymbol{\Gamma}_1^T \boldsymbol{\Delta}_*^T) \\
&= m_1 \operatorname{tr}\{[\mathbf{I}_p - \mathbf{G}_{\mathbf{A}}(\mathbf{I}_d + \mathbf{A}^T\mathbf{A})^{-1}\mathbf{G}_{\mathbf{A}}^T]\boldsymbol{\Delta}_*(\mathbf{I}_d + \mathbf{A}^T\mathbf{A})^{-1}\boldsymbol{\Delta}_*^T\} \\
&= m_1 \operatorname{tr}\{\boldsymbol{\Delta}^T[\mathbf{I}_{p-d} - \mathbf{A}(\mathbf{I}_d + \mathbf{A}^T\mathbf{A})^{-1}\mathbf{A}^T]\boldsymbol{\Delta}(\mathbf{I}_d + \mathbf{A}^T\mathbf{A})^{-1}\} \\
&= m_1 \operatorname{tr}\{\boldsymbol{\Delta}^T(\mathbf{I}_d + \mathbf{A}^T\mathbf{A})^{-1}\boldsymbol{\Delta}(\mathbf{I}_d + \mathbf{A}^T\mathbf{A})^{-1}\} \\
&= m_1 \operatorname{vec}(\boldsymbol{\Delta})^T[(\mathbf{I}_d + \mathbf{A}^T\mathbf{A})^{-1} \otimes (\mathbf{I}_d + \mathbf{A}^T\mathbf{A})^{-1}] \operatorname{vec}(\boldsymbol{\Delta}) \geq m_1 m_2^2 \|\boldsymbol{\Delta}\|_F^2,
\end{aligned}
$$

where m_2 is the smallest eigenvalue of $(\mathbf{I}_d + \mathbf{A}^T\mathbf{A})^{-1}$. When $\|\boldsymbol{\Delta}\|_F > C$ for sufficiently large C, the terms with order $\|\boldsymbol{\Delta}\|_F^2$ dominate the terms with order $\|\boldsymbol{\Delta}\|_F$. Then $f_{obj}(\mathbf{A} + n^{-1/2}\boldsymbol{\Delta}) - f_{obj}(\mathbf{A}) > 0$ with probability tending to 1, and conclusion (S10) follows.

Proof of Theorem 7 Recall that, in the proof of Theorem 6, we use the reparameterization of $\boldsymbol{\Gamma}$, and $\widehat{\boldsymbol{\Gamma}}_i = \mathbf{0}$ is equivalent to $\hat{\mathbf{a}}_i = \mathbf{0}$. Here, we prove $\Pr(\hat{\mathbf{a}}_i = \mathbf{0}) \to 1$, $i = p_{\mathcal{A}} - d + 1, \ldots, p - d$ by contradiction. Suppose that $\hat{\mathbf{a}}_i \neq \mathbf{0}$ for $i = p_{\mathcal{A}} - d + 1, \ldots, p - d$. Let $\mathbf{e}_i \in \mathbb{R}^d$ denote a vector of $\mathbf{0}$ with a 1

in the ith place. Then,

$$\left.\frac{df_{obj}(\mathbf{A})}{d\mathbf{a}_i^T}\right|_{\mathbf{a}_i=\hat{\mathbf{a}}_i} = \mathbf{0}, \tag{S12}$$

where

$$\begin{aligned}\left.\frac{df_{obj}(\mathbf{A})}{d\mathbf{a}_i^T}\right|_{\mathbf{a}_i=\hat{\mathbf{a}}_i} \quad = \quad & -4\mathbf{e}_i^T\mathbf{G}_\mathbf{A}(\mathbf{I}_d+\hat{\mathbf{A}}^T\hat{\mathbf{A}})^{-1} + 2\mathbf{e}_i^T\mathbf{S}_{\mathbf{M}|\mathbf{X},\mathbf{Z}}^{-1}\mathbf{G}_\mathbf{A}(\mathbf{G}_\mathbf{A}^T\mathbf{S}_{\mathbf{M}|\mathbf{X},\mathbf{Z}}^{-1}\mathbf{G}_\mathbf{A})^{-1} \\ & +2\mathbf{e}_i^T\mathbf{S}_{\mathbf{M}|\mathbf{Y},\mathbf{X},\mathbf{Z}}\mathbf{G}_\mathbf{A}(\mathbf{G}_\mathbf{A}^T\mathbf{S}_{\mathbf{M}|\mathbf{Y},\mathbf{X},\mathbf{Z}}\mathbf{G}_\mathbf{A})^{-1} + \lambda w_i\hat{\mathbf{a}}_i^T/\|\hat{\mathbf{a}}_i\|_2.\end{aligned}$$

Since $\hat{\mathbf{A}}$, $\mathbf{S}_{\mathbf{M}|\mathbf{X},\mathbf{Z}}$, and $\mathbf{S}_{\mathbf{M}|\mathbf{Y},\mathbf{X},\mathbf{Z}}$ are $\sqrt{n}$-consistent estimators of $\mathbf{A}$, $\boldsymbol{\Sigma}_{\mathbf{M}|\mathbf{X},\mathbf{Z}}$, and $\boldsymbol{\Sigma}_{\mathbf{M}|\mathbf{Y},\mathbf{X},\mathbf{Z}}$,

$$\begin{aligned}& -4\mathbf{e}_i^T\mathbf{G}_\mathbf{A}(\mathbf{I}_d+\hat{\mathbf{A}}^T\hat{\mathbf{A}})^{-1} + 2\mathbf{e}_i^T\mathbf{S}_{\mathbf{M}|\mathbf{X},\mathbf{Z}}^{-1}\mathbf{G}_\mathbf{A}(\mathbf{G}_\mathbf{A}^T\mathbf{S}_{\mathbf{M}|\mathbf{X},\mathbf{Z}}^{-1}\mathbf{G}_\mathbf{A})^{-1} + 2\mathbf{e}_i^T\mathbf{S}_{\mathbf{M}|\mathbf{Y},\mathbf{X},\mathbf{Z}}\mathbf{G}_\mathbf{A}(\mathbf{G}_\mathbf{A}\mathbf{S}_{\mathbf{M}|\mathbf{Y},\mathbf{X},\mathbf{Z}}\mathbf{G}_\mathbf{A}^T)^{-1} \\ = \quad & -4\mathbf{e}_i^T\mathbf{G}_\mathbf{A}(\mathbf{I}_d+\mathbf{A}^T\mathbf{A})^{-1} + 2\mathbf{e}_i^T\boldsymbol{\Sigma}_{\mathbf{M}|\mathbf{X},\mathbf{Z}}^{-1}\mathbf{G}_\mathbf{A}(\mathbf{G}_\mathbf{A}^T\boldsymbol{\Sigma}_{\mathbf{M}|\mathbf{X},\mathbf{Z}}^{-1}\mathbf{G}_\mathbf{A})^{-1} \\ & +2\mathbf{e}_i^T\boldsymbol{\Sigma}_{\mathbf{M}|\mathbf{Y},\mathbf{X},\mathbf{Z}}\mathbf{G}_\mathbf{A}(\mathbf{G}_\mathbf{A}\boldsymbol{\Sigma}_{\mathbf{M}|\mathbf{Y},\mathbf{X},\mathbf{Z}}\mathbf{G}_\mathbf{A}^T)^{-1} + O_p(n^{-1/2}) \\ = \quad & -4\mathbf{e}_i^T\mathbf{G}_\mathbf{A}(\mathbf{I}_d+\mathbf{A}^T\mathbf{A})^{-1} + 2\mathbf{e}_i^T\boldsymbol{\Sigma}_{\mathbf{M}|\mathbf{X},\mathbf{Z}}^{-1}\boldsymbol{\Gamma}\boldsymbol{\Gamma}_1^{-1}(\boldsymbol{\Gamma}_1^{-T}\boldsymbol{\Gamma}^T\boldsymbol{\Sigma}_{\mathbf{M}|\mathbf{X},\mathbf{Z}}^{-1}\boldsymbol{\Gamma}\boldsymbol{\Gamma}_1^{-1})^{-1} \\ & +2\mathbf{e}_i^T\boldsymbol{\Sigma}_{\mathbf{M}|\mathbf{Y},\mathbf{X},\mathbf{Z}}\boldsymbol{\Gamma}\boldsymbol{\Gamma}_1^{-1}(\boldsymbol{\Gamma}_1^{-T}\boldsymbol{\Gamma}^T\boldsymbol{\Sigma}_{\mathbf{M}|\mathbf{Y},\mathbf{X},\mathbf{Z}}\boldsymbol{\Gamma}\boldsymbol{\Gamma}_1)^{-1} + O_p(n^{-1/2}) \\ = \quad & -4\mathbf{e}_i^T\mathbf{G}_\mathbf{A}(\mathbf{I}_d+\mathbf{A}^T\mathbf{A})^{-1} + 2\mathbf{e}_i^T\mathbf{G}_\mathbf{A}\boldsymbol{\Gamma}_1\boldsymbol{\Gamma}_1^T + 2\mathbf{e}_i^T\mathbf{G}_\mathbf{A}\boldsymbol{\Gamma}_1\boldsymbol{\Gamma}_1^T + O_p(n^{-1/2}) = O_p(n^{-1/2}).\end{aligned}$$

The last equality is because of (S11). Then,

$$\sqrt{n}\| -4\mathbf{e}_i^T\mathbf{G}_{\mathbf{A}}(\mathbf{I}_d+\hat{\mathbf{A}}^T\hat{\mathbf{A}})^{-1}+2\mathbf{e}_i^T\mathbf{S}_{\mathbf{M}|\mathbf{X},\mathbf{Z}}^{-1}\mathbf{G}_{\mathbf{A}}(\mathbf{G}_{\mathbf{A}}^T\mathbf{S}_{\mathbf{M}|\mathbf{X},\mathbf{Z}}^{-1}\mathbf{G}_{\mathbf{A}})^{-1}$$
$$+2\mathbf{e}_i^T\mathbf{S}_{\mathbf{M}|\mathbf{Y},\mathbf{X},\mathbf{Z}}\mathbf{G}_{\mathbf{A}}(\mathbf{G}_{\mathbf{A}}\mathbf{S}_{\mathbf{M}|\mathbf{Y},\mathbf{X},\mathbf{Z}}\mathbf{G}_{\mathbf{A}}^T)^{-1}\|_2 = O_p(1). \quad \text{(S13)}$$

On the other hand, for $i = p_{\mathcal{A}} - d + 1, \ldots, p - d$,

$$\sqrt{n}\lambda w_i \left\| \frac{\hat{\mathbf{a}}_i^T}{\|\hat{\mathbf{a}}_i\|_2} \right\|_2 = \sqrt{n}\lambda w_i \geq \sqrt{n}\lambda_{\mathcal{I}} \to \infty. \quad \text{(S14)}$$

Note that (S12), (S13), and (S14) are contradictory to each other. So we have $\Pr(\hat{\mathbf{a}}_i = \mathbf{0}) \to 1$, $i = p_{\mathcal{A}} - d + 1, \ldots, p - d$.

Proof of Web Proposition 3 By incorporating the oracle information and substituting the sparse structure of $\mathbf{A}$ to (6), we obtain the objective function of $\mathbf{A}_{\mathcal{A}}$ as displayed in Web Proposition 3. Since the oracle ESPLSM model (S3) is over-parameterized, we use Theorem 4.1 in Shapiro (1986) to derive the asymptotic distribution of $\widehat{\boldsymbol{\beta}}_{\mathbf{M}_{\mathcal{A}},O}$. We use $\boldsymbol{\xi}$ to denote the parameters in (6), and $\boldsymbol{\xi} = (\boldsymbol{\mu}_{\mathbf{Y}}^T, \boldsymbol{\mu}_{\mathbf{M}}^T, \boldsymbol{\mu}_{\mathbf{X}}^T, \boldsymbol{\mu}_{\mathbf{Z}}^T, \mathrm{vec}^T(\boldsymbol{\beta}_{\mathbf{X}}), \mathrm{vec}^T(\boldsymbol{\beta}_{\mathbf{Z}}), \mathrm{vec}^T(\boldsymbol{\beta}_{\mathbf{M}_{\mathcal{A}}}), \mathrm{vec}^T(\boldsymbol{\gamma}_{\mathbf{X}}), \mathrm{vec}^T(\boldsymbol{\gamma}_{\mathbf{Z}}), \mathrm{vech}^T(\boldsymbol{\Sigma}_{\mathbf{Y}|\mathbf{M},\mathbf{X},\mathbf{Z}}), \mathrm{vech}^T(\boldsymbol{\Sigma}_{\mathbf{M}|\mathbf{X},\mathbf{Z}}))^T$. We use $\boldsymbol{\phi}$ to denote the constituent parameters, and $\boldsymbol{\phi} = (\boldsymbol{\mu}_{\mathbf{Y}}^T, \boldsymbol{\mu}_{\mathbf{M}}^T, \boldsymbol{\mu}_{\mathbf{X}}^T, \boldsymbol{\mu}_{\mathbf{Z}}^T, \mathrm{vec}^T(\boldsymbol{\beta}_{\mathbf{X}}), \mathrm{vec}^T(\boldsymbol{\beta}_{\mathbf{Z}}), \mathrm{vec}^T(\boldsymbol{\Gamma}), \mathrm{vec}^T(\boldsymbol{\eta}), \mathrm{vec}^T(\boldsymbol{\gamma}_{\mathbf{X}}), \mathrm{vec}^T(\boldsymbol{\gamma}_{\mathbf{Z}}),$

$\text{vech}^T(\boldsymbol{\Sigma}_{\mathbf{Y}|\mathbf{M},\mathbf{X},\mathbf{Z}}),\ \text{vech}^T(\boldsymbol{\Omega}),\ \text{vech}^T(\boldsymbol{\Omega}_0))^T$. They are the same as the parameters of the EPLSM model except that $\text{vec}^T(\boldsymbol{\beta}_{\mathbf{M}})$ in $\boldsymbol{\xi}$ and $\text{vec}^T(\boldsymbol{\Gamma})$ in $\boldsymbol{\phi}$ have sparse structure

$$\boldsymbol{\beta}_{\mathbf{M}} = \begin{pmatrix} \boldsymbol{\beta}_{\mathbf{M}_{\mathcal{A}}} \\ \mathbf{0} \end{pmatrix} \quad \text{and} \quad \boldsymbol{\Gamma} = \begin{pmatrix} \boldsymbol{\Gamma}_{\mathcal{A}} \\ \mathbf{0} \end{pmatrix},$$

and we have oracle information about the sparsity structure. The gradient matrix $\mathbf{H} = \partial\boldsymbol{\xi}/\partial\boldsymbol{\phi}^T$ and the Fisher information matrix $\mathbf{J}$ of $\boldsymbol{\xi}$ have similar forms as those in Web Proposition 1. We omit those for simplicity. Using Proposition 4.1 of Shapiro (1986), we have $\sqrt{n}\{\,\text{vec}(\hat{\boldsymbol{\xi}}(\hat{\boldsymbol{\phi}})) - \text{vec}(\boldsymbol{\xi}(\boldsymbol{\phi}))\} \xrightarrow{d} N(\mathbf{0}, \mathbf{H}(\mathbf{H}^T\mathbf{J}\mathbf{H})^{\dagger}\mathbf{H}^T)$. Following the same logic as in the derivation of Proposition 5, we have $\sqrt{n}(\hat{\boldsymbol{\gamma}}_{\mathbf{X},O} - \boldsymbol{\gamma}_{\mathbf{X}}) \xrightarrow{d} N(\mathbf{0}, \mathbf{V}_{\boldsymbol{\gamma}_{\mathbf{X},O}})$, where $\mathbf{V}_{\boldsymbol{\gamma}_{\mathbf{X},O}} = \boldsymbol{\Sigma}_{\mathbf{M}|\mathbf{X},\mathbf{Z}} \otimes \boldsymbol{\Sigma}^{-1}_{\mathbf{X}|\mathbf{Z}}$. We notice that this is unchanged from $\mathbf{V}_{\boldsymbol{\gamma}_{\mathbf{X}}}$ because its asymptotic distribution is independent of those that involved in $\boldsymbol{\mu}$'s, $\boldsymbol{\beta}$'s, and $\boldsymbol{\Sigma}$'s. After some calculations similar to those in Web Proposition 1, we have $\sqrt{n}\{\,\text{vec}(\widehat{\boldsymbol{\beta}}_{\mathbf{X},O}) - \text{vec}(\boldsymbol{\beta}_{\mathbf{X}})\} \xrightarrow{d} N(\mathbf{0}, \mathbf{V}_{direct,O})$ and $\sqrt{n}\{\,\text{vec}(\widehat{\boldsymbol{\beta}}_{\mathbf{M}_{\mathcal{A}},O}) - \text{vec}(\boldsymbol{\beta}_{\mathbf{M}_{\mathcal{A}}})\} \xrightarrow{d} N(\mathbf{0}, \mathbf{V}_O)$, where

$$\begin{aligned}
\mathbf{V}_{direct,O} \;=\;& \boldsymbol{\Sigma}_{\mathbf{Y}|\mathbf{M},\mathbf{X},\mathbf{Z}} \otimes (\boldsymbol{\gamma}_{\mathbf{X}}\mathbf{K}\boldsymbol{\Gamma}_{\mathcal{A}}\boldsymbol{\Omega}^{-1}\boldsymbol{\Gamma}^T_{\mathcal{A}}\mathbf{K}^T\boldsymbol{\gamma}^T_{\mathbf{X}} + \boldsymbol{\Sigma}^{-1}_{\mathbf{X}|\mathbf{Z}}) \\
& + (\boldsymbol{\eta}^T \otimes \boldsymbol{\gamma}_{\mathbf{X}}\mathbf{K}\boldsymbol{\Gamma}_{\mathcal{A},0})(\boldsymbol{\eta}\boldsymbol{\Sigma}^{-1}_{\mathbf{Y}|\mathbf{M},\mathbf{X},\mathbf{Z}}\boldsymbol{\eta}^T \otimes \tilde{\boldsymbol{\Omega}}_{0,\mathcal{A}} \\
& + \boldsymbol{\Omega} \otimes \tilde{\boldsymbol{\Omega}}^{-1}_{0,\mathcal{A}|\mathcal{I}} + \boldsymbol{\Omega}^{-1} \otimes \tilde{\boldsymbol{\Omega}}_{0,\mathcal{A}} - 2\mathbf{I}_u \otimes \mathbf{I}_{p_{\mathcal{A}}-u})^{-1}(\boldsymbol{\eta} \otimes \boldsymbol{\Gamma}^T_{\mathcal{A},0}\mathbf{K}^T\boldsymbol{\gamma}^T_{\mathbf{X}})
\end{aligned}$$

and

$$\mathbf{V}_O = \boldsymbol{\Sigma}_{\mathbf{Y}|\mathbf{M}_\mathcal{A},\mathbf{X},\mathbf{Z}} \otimes \boldsymbol{\Gamma}_\mathcal{A}\boldsymbol{\Omega}^{-1}\boldsymbol{\Gamma}_\mathcal{A}^T + (\boldsymbol{\eta}^T \otimes \boldsymbol{\Gamma}_{\mathcal{A},0})(\boldsymbol{\eta}\boldsymbol{\Sigma}^{-1}_{\mathbf{Y}|\mathbf{M}_\mathcal{A},\mathbf{X},\mathbf{Z}}\boldsymbol{\eta}^T \otimes \tilde{\boldsymbol{\Omega}}_{0,\mathcal{A}} + \boldsymbol{\Omega} \otimes \tilde{\boldsymbol{\Omega}}^{-1}_{0,\mathcal{A}|\mathcal{I}}$$
$$+\boldsymbol{\Omega}^{-1} \otimes \tilde{\boldsymbol{\Omega}}_{0,\mathcal{A}} - 2\mathbf{I}_d \otimes \mathbf{I}_{p_\mathcal{A}-d})^{-1}(\boldsymbol{\eta} \otimes \boldsymbol{\Gamma}_{\mathcal{A},0}).$$

By the Delta's method, the indirect effect $\mathrm{vec}(\hat{\boldsymbol{\gamma}}_\mathbf{X}\hat{\boldsymbol{\beta}}_\mathbf{M})$ has asymptotic variance

$$\mathbf{V}_{indirect,O} = (\mathbf{I}_{p_\mathcal{A}r}\otimes\boldsymbol{\gamma}_\mathbf{X})\mathbf{V}_{\mathbf{M}_\mathcal{A}}(\mathbf{I}_{p_\mathcal{A}r}\otimes\boldsymbol{\gamma}_\mathbf{X})^T+(\boldsymbol{\beta}^T_{\mathbf{M}_\mathcal{A}}\otimes\mathbf{I}_{kp})(\boldsymbol{\Sigma}_{\mathbf{M}|\mathbf{X},\mathbf{Z}}\otimes\boldsymbol{\Sigma}^{-1}_{\mathbf{X}|\mathbf{Z}})(\boldsymbol{\beta}_{\mathbf{M}_\mathcal{A}}\otimes\mathbf{I}_{kp}).$$

Proof of Web Theorem 4 Let $\hat{\mathbf{A}}_\mathcal{A}$ denote the ESPLSM estimator of $\mathbf{A}_\mathcal{A}$. Suppose we can prove $\hat{\mathbf{A}}_\mathcal{A} = \hat{\mathbf{A}}_{\mathcal{A},O} + o_p(a_n)$ for a sequence a_n such that $a_n = o(n^{-1/2})$, then $\hat{\mathbf{G}}_{\mathbf{A}_\mathcal{A}} = \hat{\mathbf{G}}_{\mathbf{A}_{\mathcal{A},O}}+o_p(a_n)$, $\hat{\boldsymbol{\beta}}_{\mathbf{M}_\mathcal{A}} = \hat{\boldsymbol{\beta}}_{\mathbf{M}_\mathcal{A},O}+o_p(a_n)$, and $\sqrt{n}\{\mathrm{vec}(\widehat{\boldsymbol{\beta}}_{\mathbf{M}_\mathcal{A}})- \mathrm{vec}(\boldsymbol{\beta}_{\mathbf{M}_\mathcal{A}})\}$ and $\sqrt{n}\{\mathrm{vec}(\widehat{\boldsymbol{\beta}}_{\mathbf{M}_\mathcal{A},O}) - \mathrm{vec}(\boldsymbol{\beta}_{\mathbf{M}_\mathcal{A}})\}$ converge to the same asymptotic distribution. Similarly, $\sqrt{n}\{\mathrm{vec}(\widehat{\boldsymbol{\beta}}_\mathbf{X}) - \mathrm{vec}(\boldsymbol{\beta}_\mathbf{X})\}$ and $\sqrt{n}\{\mathrm{vec}(\widehat{\boldsymbol{\beta}}_{\mathbf{X},O}) - \mathrm{vec}(\boldsymbol{\beta}_\mathbf{X})\}$ converge to the same asymptotic distribution, $\sqrt{n}\{\mathrm{vec}(\hat{\boldsymbol{\gamma}}_\mathbf{X})- \mathrm{vec}(\boldsymbol{\gamma}_\mathbf{X})\}$ and $\sqrt{n}\{\mathrm{vec}(\hat{\boldsymbol{\gamma}}_{\mathbf{X},O})- \mathrm{vec}(\boldsymbol{\gamma}_\mathbf{X})\}$ converge to the same asymptotic distribution, and thus $\sqrt{n}\{\mathrm{vec}(\hat{\boldsymbol{\gamma}}_\mathbf{X}\widehat{\boldsymbol{\beta}}_\mathbf{M})- \mathrm{vec}(\boldsymbol{\gamma}_\mathbf{X}\boldsymbol{\beta}_\mathbf{M})\}$ and $\sqrt{n}\{\mathrm{vec}(\hat{\boldsymbol{\gamma}}_{\mathbf{X},O}\widehat{\boldsymbol{\beta}}_{\mathbf{M},O})- \mathrm{vec}(\boldsymbol{\gamma}_\mathbf{X}\boldsymbol{\beta}_\mathbf{M})\}$ converge to the same asymptotic distribution. Regarding the choice of a_n, we can take $a_n = (n^{-1/2}\lambda_\mathcal{A})^{1/2}$.

Since the ESPLSM estimator enjoys the selection consistency, the objective function to estimate $\mathbf{A}_\mathcal{A}$ is

$$\begin{aligned} f_{obj,\mathcal{A}}(\mathbf{A}_\mathcal{A}) &= -2\log|\mathbf{G}_{\mathbf{A}_\mathcal{A}}^T\mathbf{G}_{\mathbf{A}_\mathcal{A}}| + \log|\mathbf{G}_{\mathbf{A}_\mathcal{A}}^T\mathbf{S}_{\mathbf{M}_\mathcal{A}|\mathbf{Y},\mathbf{X},\mathbf{Z}}\mathbf{G}_{\mathbf{A}_\mathcal{A}}| \\ &\quad + \log|\mathbf{G}_{\mathbf{A}_\mathcal{A}}^T(\mathbf{S}_{\mathbf{M}|\mathbf{X},\mathbf{Z}}^{-1})_\mathcal{A}\mathbf{G}_{\mathbf{A}_\mathcal{A}}| + \sum_{i=1}^{p_\mathcal{A}-d} w_i\|\mathbf{a}_i\|_2. \end{aligned}$$

In order to prove $\hat{\mathbf{A}}_\mathcal{A} = \hat{\mathbf{A}}_{\mathcal{A},O} + o_p(a_n)$, it is sufficient to show that for any $\epsilon > 0$, there exists a sufficiently large constant C such that

$$\lim_{n\to\infty} \Pr\left(\inf_{\substack{\mathbf{\Delta}\in\mathbb{R}^{(p_\mathcal{A}-d)\times d}, \\ \|\mathbf{\Delta}\|_F=C}} f_{obj,\mathcal{A}}(\hat{\mathbf{A}}_{\mathcal{A},O} + a_n\mathbf{\Delta}) > f_{obj,\mathcal{A}}(\hat{\mathbf{A}}_{\mathcal{A},O})\right) > 1-\epsilon. \tag{S15}$$

We name the four summands in $f_{obj,\mathcal{A}}(\mathbf{A}_\mathcal{A})$ as $f_1(\mathbf{A}_\mathcal{A})$, $f_2(\mathbf{A}_\mathcal{A})$, $f_3(\mathbf{A}_\mathcal{A})$, and $f_4(\mathbf{A}_\mathcal{A})$, i.e., $f_1(\mathbf{A}_\mathcal{A}) = -2\log|\mathbf{G}_{\mathbf{A}_\mathcal{A}}^T\mathbf{G}_{\mathbf{A}_\mathcal{A}}|$, $f_2(\mathbf{A}_\mathcal{A}) = \log|\mathbf{G}_{\mathbf{A}_\mathcal{A}}^T\mathbf{S}_{\mathbf{M}_\mathcal{A}|\mathbf{Y},\mathbf{X},\mathbf{Z}}\mathbf{G}_{\mathbf{A}_\mathcal{A}}|$, $f_3(\mathbf{A}_\mathcal{A}) = \log|\mathbf{G}_{\mathbf{A}_\mathcal{A}}^T(\mathbf{S}_{\mathbf{M}|\mathbf{X},\mathbf{Z}}^{-1})_\mathcal{A}\mathbf{G}_{\mathbf{A}_\mathcal{A}}|$, and $f_4(\mathbf{A}_\mathcal{A}) = \sum_{i=1}^{p_\mathcal{A}-d}\lambda w_i\|\mathbf{a}_i\|_2$. Notice that the objective function of $\mathbf{A}_\mathcal{A}$ under the oracle ESPLSM model (S3) is $f_1(\mathbf{A}_\mathcal{A}) + f_2(\mathbf{A}_\mathcal{A}) + f_3(\mathbf{A}_\mathcal{A})$. So,

$$\left.\frac{df_1(\mathbf{A}_\mathcal{A})}{d\mathbf{A}_\mathcal{A}}\right|_{\mathbf{A}_\mathcal{A}=\hat{\mathbf{A}}_{\mathcal{A},O}} + \left.\frac{df_2(\mathbf{A}_\mathcal{A})}{d\mathbf{A}_\mathcal{A}}\right|_{\mathbf{A}_\mathcal{A}=\hat{\mathbf{A}}_{\mathcal{A},O}} + \left.\frac{df_3(\mathbf{A}_\mathcal{A})}{d\mathbf{A}_\mathcal{A}}\right|_{\mathbf{A}_\mathcal{A}=\hat{\mathbf{A}}_{\mathcal{A},O}} = \mathbf{0}. \tag{S16}$$

Now, we compute $f_{obj,\mathcal{A}}(\hat{\mathbf{A}}_{\mathcal{A},O} + a_n\boldsymbol{\Delta}) - f_{obj,\mathcal{A}}(\hat{\mathbf{A}}_{\mathcal{A},O})$ using Taylor expansion

$$\begin{aligned}
& f_{obj,\mathcal{A}}(\hat{\mathbf{A}}_{\mathcal{A},O} + a_n\boldsymbol{\Delta}) - f_{obj,\mathcal{A}}(\hat{\mathbf{A}}_{\mathcal{A},O}) \\
= \;& a_n\{\overset{\rightarrow\Delta}{df_1}(\hat{\mathbf{A}}_{\mathcal{A},O}) + \overset{\rightarrow\Delta}{df_2}(\hat{\mathbf{A}}_{\mathcal{A},O}) + \overset{\rightarrow\Delta}{df_3}(\hat{\mathbf{A}}_{\mathcal{A},O})\} + (1/2)a_n^2\{\overset{\rightarrow\Delta}{df_1^2}(\hat{\mathbf{A}}_{\mathcal{A},O}) \\
& + \overset{\rightarrow\Delta}{df_2^2}(\hat{\mathbf{A}}_{\mathcal{A},O}) + \overset{\rightarrow\Delta}{df_3^2}(\hat{\mathbf{A}}_{\mathcal{A},O})\} + f_4(\hat{\mathbf{A}}_{\mathcal{A},O} + a_n\boldsymbol{\Delta}) - f_4(\hat{\mathbf{A}}_{\mathcal{A},O}) + o_p(a_n^2).
\end{aligned}$$

Because of (S16), we have

$$\overset{\rightarrow\Delta}{df_1}(\hat{\mathbf{A}}_{\mathcal{A},O}) + \overset{\rightarrow\Delta}{df_2}(\hat{\mathbf{A}}_{\mathcal{A},O}) + \overset{\rightarrow\Delta}{df_3}(\hat{\mathbf{A}}_{\mathcal{A},O}) = \mathbf{0}. \tag{S17}$$

Let $\boldsymbol{\Delta}_{*\mathcal{A}} = (\mathbf{0}, \boldsymbol{\Delta}^T)^T \in \mathbb{R}^{p_{\mathcal{A}} \times d}$. Following similar calculations as those in the proof of Theorem 6, we have

$$\begin{aligned}
& \overset{\rightarrow\Delta}{df_1^2}(\hat{\mathbf{A}}_{\mathcal{A},O}) + \overset{\rightarrow\Delta}{df_2^2}(\hat{\mathbf{A}}_{\mathcal{A},O}) + \overset{\rightarrow\Delta}{df_3^2}(\hat{\mathbf{A}}_{\mathcal{A},O}) \\
& = 2\,\mathrm{tr}\{\boldsymbol{\Omega}\boldsymbol{\Gamma}_1^T\boldsymbol{\Delta}_{*\mathcal{A}}^T\boldsymbol{\Gamma}_{\mathcal{A},0}\tilde{\boldsymbol{\Omega}}_{0,\mathcal{A}|\mathcal{I}}^{-1}\boldsymbol{\Gamma}_{\mathcal{A},0}^T\boldsymbol{\Delta}_{*\mathcal{A}}\boldsymbol{\Gamma}_1 + (\boldsymbol{\Omega}^{-1} + \boldsymbol{\eta}\boldsymbol{\Sigma}_{\mathbf{Y}|\mathbf{M},\mathbf{X},\mathbf{Z}}^{-1}\boldsymbol{\eta}^T)\boldsymbol{\Gamma}_1^T\boldsymbol{\Delta}_{*\mathcal{A}}^T\boldsymbol{\Gamma}_{\mathcal{A},0}\tilde{\boldsymbol{\Omega}}_{0,\mathcal{A}}\boldsymbol{\Gamma}_{\mathcal{A},0}\boldsymbol{\Delta}_{*\mathcal{A}}\boldsymbol{\Gamma}_1 \\
& - 2(\mathbf{I}_d + \mathbf{A}_{\mathcal{A}}^T\mathbf{A}_{\mathcal{A}})^{-1}\boldsymbol{\Delta}_{*\mathcal{A}}^T\boldsymbol{\Gamma}_{\mathcal{A},0}\boldsymbol{\Gamma}_{\mathcal{A},0}^T\boldsymbol{\Delta}_{*\mathcal{A}}\} + o_p(1)
\end{aligned} \tag{S18}$$

and

$$f_4(\hat{\mathbf{A}}_{\mathcal{A},O} + a_n\boldsymbol{\Delta}) - f_4(\hat{\mathbf{A}}_{\mathcal{A},O}) \geq \sum_{i=1}^{p_{\mathcal{A}}-d} -(p_{\mathcal{A}} - d)a_n\lambda_{\mathcal{A}} \max_{1\leq i\leq p_{\mathcal{A}}-d}\|\boldsymbol{\delta}_i\|_2. \tag{S19}$$

Since $a_n = (n^{-1/2}\lambda_{\mathcal{A}})^{1/2}$ and $\lambda_{\mathcal{A}} = o(n^{-1/2})$, then $a_n\lambda_{\mathcal{A}} = o(a_n^2)$. The right-hand side of (S19) is dominated by a_n^2. Combining (S17), (S18), and (S19),

$$
\begin{aligned}
& f_{obj,\mathcal{A}}(\hat{\mathbf{A}}_{\mathcal{A},O} + a_n\boldsymbol{\Delta}) - f_{obj,\mathcal{A}}(\hat{\mathbf{A}}_{\mathcal{A},O}) \\
= \; & a_n^2 \operatorname{tr}\{\boldsymbol{\Omega}\boldsymbol{\Gamma}_1^T\boldsymbol{\Delta}_{*\mathcal{A}}^T\boldsymbol{\Gamma}_{\mathcal{A},0}\tilde{\boldsymbol{\Omega}}_{0,\mathcal{A}|\mathcal{I}}^{-1}\boldsymbol{\Gamma}_{\mathcal{A},0}^T\boldsymbol{\Delta}_{*\mathcal{A}}\boldsymbol{\Gamma}_1 + (\boldsymbol{\Omega}^{-1} + \boldsymbol{\eta}\boldsymbol{\Sigma}_{\mathbf{Y}|\mathbf{M},\mathbf{X},\mathbf{Z}}^{-1}\boldsymbol{\eta}^T)\boldsymbol{\Gamma}_1^T\boldsymbol{\Delta}_{*\mathcal{A}}^T\boldsymbol{\Gamma}_{\mathcal{A},0}\tilde{\boldsymbol{\Omega}}_{0,\mathcal{A}}\boldsymbol{\Gamma}_{\mathcal{A},0}\boldsymbol{\Delta}_{*\mathcal{A}}\boldsymbol{\Gamma}_1 \\
& -2(\mathbf{I}_d + \mathbf{A}_{\mathcal{A}}^T\mathbf{A}_{\mathcal{A}})^{-1}\boldsymbol{\Delta}_{*\mathcal{A}}^T\boldsymbol{\Gamma}_{\mathcal{A},0}\boldsymbol{\Gamma}_{\mathcal{A},0}^T\boldsymbol{\Delta}_{*\mathcal{A}}\} + o_p(a_n^2) \\
= \; & a_n^2 \operatorname{vec}(\boldsymbol{\Gamma}_{\mathcal{A},0}^T\boldsymbol{\Delta}_{*\mathcal{A}}\boldsymbol{\Gamma}_1)^T\mathbf{T}\operatorname{vec}(\boldsymbol{\Gamma}_{\mathcal{A},0}^T\boldsymbol{\Delta}_{*\mathcal{A}}\boldsymbol{\Gamma}_1) + o_p(a_n^2),
\end{aligned}
$$

where $\mathbf{T} = (\boldsymbol{\Omega}^{-1} + \boldsymbol{\eta}\boldsymbol{\Sigma}_{\mathbf{Y}|\mathbf{M},\mathbf{X},\mathbf{Z}}^{-1}\boldsymbol{\eta}^T) \otimes \tilde{\boldsymbol{\Omega}}_{0,\mathcal{A}} + \boldsymbol{\Omega} \otimes \tilde{\boldsymbol{\Omega}}_{0,\mathcal{A}|I}^{-1} - 2\mathbf{I}_d \otimes \mathbf{I}_{p_{\mathcal{A}}-d}$ appeared in Web Proposition 3, and it is invertible. Because

$$
\begin{aligned}
\mathbf{T} \;\geq\; & \boldsymbol{\Omega}^{-1} \otimes \tilde{\boldsymbol{\Omega}}_{0,\mathcal{A}} + \boldsymbol{\Omega} \otimes \tilde{\boldsymbol{\Omega}}_{0,\mathcal{A}}^{-1} - 2\mathbf{I}_d \otimes \mathbf{I}_{p_{\mathcal{A}}-d} + \boldsymbol{\eta}\boldsymbol{\Sigma}_{\mathbf{Y}|\mathbf{M},\mathbf{X},\mathbf{Z}}^{-1}\boldsymbol{\eta}^T \otimes \tilde{\boldsymbol{\Omega}}_{0,\mathcal{A}} \\
= \; & (\boldsymbol{\Omega}^{-1/2} \otimes \tilde{\boldsymbol{\Omega}}_{0,\mathcal{A}}^{1/2} - \boldsymbol{\Omega}^{1/2} \otimes \tilde{\boldsymbol{\Omega}}_{0,\mathcal{A}}^{-1/2})^2 + \boldsymbol{\eta}\boldsymbol{\Sigma}_{\mathbf{Y}|\mathbf{M},\mathbf{X},\mathbf{Z}}^{-1}\boldsymbol{\eta}^T \otimes \tilde{\boldsymbol{\Omega}}_{0,\mathcal{A}},
\end{aligned}
$$

$\mathbf{T}$ is semi-positive definite. Since $\mathbf{T}$ is invertible, $\mathbf{T}$ is then positive definite. Let m_1 denote the smallest eigenvalue. Then,

$$
\operatorname{vec}(\boldsymbol{\Gamma}_{\mathcal{A},0}^T\boldsymbol{\Delta}_{*\mathcal{A}}\boldsymbol{\Gamma}_1)^T\mathbf{T}\operatorname{vec}(\boldsymbol{\Gamma}_{\mathcal{A},0}^T\boldsymbol{\Delta}_{*\mathcal{A}}\boldsymbol{\Gamma}_1) \geq m_1\|\boldsymbol{\Gamma}_{\mathcal{A},0}^T\boldsymbol{\Delta}_{*\mathcal{A}}\boldsymbol{\Gamma}_1\|_F^2.
$$

Following the discussion at the end of the proof of Theorem 6, we also have

$\|\boldsymbol{\Gamma}_{\mathcal{A},0}^T \boldsymbol{\Delta}_{*\mathcal{A}} \boldsymbol{\Gamma}_1\|_F \geq m_2^2 \|\boldsymbol{\Delta}\|_F^2$, where m_2 denotes the smallest eigenvalue of $(\mathbf{I}_d + \mathbf{A}_{\mathcal{A}}^T \mathbf{A}_{\mathcal{A}})^{-1}$. Substituting these results to $f_{obj,\mathcal{A}}(\hat{\mathbf{A}}_{\mathcal{A},O} + a_n\boldsymbol{\Delta}) - f_{obj,\mathcal{A}}(\hat{\mathbf{A}}_{\mathcal{A},O})$, we have $f_{obj,\mathcal{A}}(\hat{\mathbf{A}}_{\mathcal{A},O} + a_n\boldsymbol{\Delta}) - f_{obj,\mathcal{A}}(\hat{\mathbf{A}}_{\mathcal{A},O}) \geq a_n^2 m_1 m_2^2 \|\boldsymbol{\Delta}\|_F^2 + o_p(a_n^2)$. Since $f_{obj,\mathcal{A}}(\hat{\mathbf{A}}_{\mathcal{A},O} + a_n\boldsymbol{\Delta}) - f_{obj,\mathcal{A}}(\hat{\mathbf{A}}_{\mathcal{A},O}) > 0$ with probability tending to 1, we have established (S15).

Proof of Web Theorem 6 We first show that

$$\|\mathbf{S}_{\mathbf{M}|\mathbf{X},\mathbf{Z},sp}^{-1} - \boldsymbol{\Sigma}_{\mathbf{M}|\mathbf{X},\mathbf{Z}}^{-1}\|_F = O_p[\{(p_n + s_1)\log(p_n)/n\}^{1/2}] \tag{S20}$$

$$\|\mathbf{S}_{\mathbf{M}|\mathbf{Y},\mathbf{X},\mathbf{Z},sp}^{-1} - \boldsymbol{\Sigma}_{\mathbf{M}|\mathbf{Y},\mathbf{X},\mathbf{Z}}^{-1}\|_F = O_p[\{(p_n + s_2)\log(p_n)/n\}^{1/2}]. \tag{S21}$$

Let $\|\cdot\|_{\max}$ denote the max norm of a matrix, which is the maximum of the absolute values of all elements in the matrix. To establish (S20) and (S21), it is sufficient to show that there exist positive constants C_1 and C_2 such that

$$\|\mathbf{S}_{\mathbf{M}|\mathbf{X},\mathbf{Z}} - \boldsymbol{\Sigma}_{\mathbf{M}|\mathbf{X},\mathbf{Z}}\|_{\max} \leq C_1\{\log(p_n)/n\}^{1/2}$$

$$\|\mathbf{S}_{\mathbf{M}|\mathbf{Y},\mathbf{X},\mathbf{Z}} - \boldsymbol{\Sigma}_{\mathbf{M}|\mathbf{Y},\mathbf{X},\mathbf{Z}}\|_{\max} \leq C_2\{\log(p_n)/n\}^{1/2}.$$

Let $\bar{\mathbf{V}}$ denote the sample mean of $\mathbf{V}$ and $\mathbf{S}_{\mathbf{V}}$ denote the sample covariance matrix of $\mathbf{V}$. For a matrix $\mathbf{A}$, let $\mathbf{A}_{ij}$ denote the (i, j)th element in $\mathbf{A}$. Then for $\delta > 0$,

$$\begin{aligned}\Pr(|\mathbf{S}_{\mathbf{V},ij} - \boldsymbol{\Sigma}_{\mathbf{V},ij}| > \delta) \quad \leq \quad & \Pr(|\{(1/n)\sum_{k=1}^{n}(\mathbf{V}_k - \boldsymbol{\mu}_{\mathbf{V}})(\mathbf{V}_k - \boldsymbol{\mu}_{\mathbf{V}})^T\}_{ij} - \boldsymbol{\Sigma}_{\mathbf{V},ij}| > \delta/2) \\ & + \Pr(|\{(\bar{\mathbf{V}} - \boldsymbol{\mu}_{\mathbf{V}})(\bar{\mathbf{V}} - \boldsymbol{\mu}_{\mathbf{V}})^T\}_{ij}| > \delta/2)\end{aligned}$$

From Lemma 1 in Ravikumar et al. (2011), there exist positive constants C_1 and C_2 such that

$$\Pr(|\{(1/n)\sum_{k=1}^{n}(\mathbf{V}_k - \boldsymbol{\mu}_{\mathbf{V}})(\mathbf{V}_k - \boldsymbol{\mu}_{\mathbf{V}})^T\}_{ij} - \boldsymbol{\Sigma}_{\mathbf{V},ij}| > \delta/2) \leq C_1 \exp(-C_2 n\delta^2) \tag{S22}$$

for all $\delta \in (0, 8\bar{k}(1 + 4\sigma^2))$. Let $(\bar{\mathbf{V}} - \boldsymbol{\mu}_{\mathbf{V}})_i$ denote the ith element in the vector $\bar{\mathbf{V}} - \boldsymbol{\mu}_{\mathbf{V}}$. Then we have

$$E[\exp\{t(\bar{\mathbf{V}} - \boldsymbol{\mu}_{\mathbf{V}})_i\}] \leq \prod_{k=1}^{n} \exp\left(\frac{t^2\sigma^2}{2n^2}\right) = \exp\left(\frac{t^2\sigma^2}{2n}\right).$$

By the Chernoff's bound, we have $\Pr(|(\bar{\mathbf{V}} - \boldsymbol{\mu}_{\mathbf{V}})_i| > \delta) \leq 2\exp(-n\delta^2/(2\sigma^2))$. For any $\delta \in (0, 1/2)$, we have $\delta^2 < \delta/2$ and

$$
\begin{aligned}
&\Pr(|\{(\bar{\mathbf{V}}-\boldsymbol{\mu}_{\mathbf{V}})(\bar{\mathbf{V}}-\boldsymbol{\mu}_{\mathbf{V}})^T\}_{ij}| > \delta/2) \\
&\le \Pr(|\sqrt{\{(\bar{\mathbf{V}}-\boldsymbol{\mu}_{\mathbf{V}})(\bar{\mathbf{V}}-\boldsymbol{\mu}_{\mathbf{V}})^T\}_{ii}}\sqrt{\{(\bar{\mathbf{V}}-\boldsymbol{\mu}_{\mathbf{V}})(\bar{\mathbf{V}}-\boldsymbol{\mu}_{\mathbf{V}})^T\}_{jj}}| > \delta/2) \\
&\le \Pr(|\{(\bar{\mathbf{V}}-\boldsymbol{\mu}_{\mathbf{V}})(\bar{\mathbf{V}}-\boldsymbol{\mu}_{\mathbf{V}})^T\}_{ii}| > \delta/2) + \Pr(|\{(\bar{\mathbf{V}}-\boldsymbol{\mu}_{\mathbf{V}})(\bar{\mathbf{V}}-\boldsymbol{\mu}_{\mathbf{V}})^T\}_{jj}| > \delta/2) \\
&\le \Pr(|\{(\bar{\mathbf{V}}-\boldsymbol{\mu}_{\mathbf{V}})(\bar{\mathbf{V}}-\boldsymbol{\mu}_{\mathbf{V}})^T\}_{ii}| > \delta^2) + \Pr(|\{(\bar{\mathbf{V}}-\boldsymbol{\mu}_{\mathbf{V}})(\bar{\mathbf{V}}-\boldsymbol{\mu}_{\mathbf{V}})^T\}_{jj}| > \delta^2) \\
&\le \Pr(|(\bar{\mathbf{V}}-\boldsymbol{\mu}_{\mathbf{V}})_i| > \delta) + \Pr(|(\bar{\mathbf{V}}-\boldsymbol{\mu}_{\mathbf{V}})_j| > \delta) \le 4\exp\left(-\frac{n\delta^2}{2\sigma^2}\right). \qquad \text{(S23)}
\end{aligned}
$$

By (S22) and (S23), there exist positive constants C_3 and C_4 such that $\Pr(|\mathbf{S}_{\mathbf{V},ij} - \boldsymbol{\Sigma}_{\mathbf{V},ij}| > \delta) \le C_3 \exp(-4n\delta^2)$. Let $\delta = C\sqrt{\log(p_n)/n}$ for some $C > 0$. By the union sum inequality, there exists a positive constant $C_{\mathbf{V}}$ such that

$$
\|\mathbf{S}_{\mathbf{V}} - \boldsymbol{\Sigma}_{\mathbf{V}}\|_{\max} \le C_{\mathbf{V}}\{\log(p_n)/n\}^{1/2} \qquad \text{(S24)}
$$

with probability tending to 1 as $n \to \infty$. Then from (S24), we have $\|\mathbf{S}_{\mathbf{e}} - \boldsymbol{\Sigma}_{\mathbf{e}}\|_{\max} \le C_{\mathbf{V}}\{\log(p_n)/n\}^{1/2}$, $\|\mathbf{S}_{(\mathbf{X},\mathbf{Z})} - \boldsymbol{\Sigma}_{(\mathbf{X},\mathbf{Z})}\|_{\max} \le C_{\mathbf{V}}\{\log(p_n)/n\}^{1/2}$, $\|\mathbf{S}_{\mathbf{Xe}} - \boldsymbol{\Sigma}_{\mathbf{Xe}}\|_{\max} \le C_{\mathbf{V}}\{\log(p_n)/n\}^{1/2}$, $\|\mathbf{S}_{\mathbf{Ze}} - \boldsymbol{\Sigma}_{\mathbf{Ze}}\|_{\max} \le C_{\mathbf{V}}\{\log(p_n)/n\}^{1/2}$, where $\mathbf{S}_{\mathbf{e}} = \sum_{i=1}^n (\mathbf{e}_i - \bar{\mathbf{e}})(\mathbf{e}_i - \bar{\mathbf{e}})^T/n$, $\bar{\mathbf{e}} = (1/n)\sum_{i=1}^n \mathbf{e}_i$, $\mathbf{S}_{\mathbf{Xe}} = (1/n)\sum_{i=1}^n (\mathbf{X}_i -$

$\bar{\mathbf{X}})(\mathbf{e}_i-\bar{\mathbf{e}})^T, \mathbf{S}_{\mathbf{Ze}} = (1/n)\sum_{i=1}^n(\mathbf{Z}_i-\bar{\mathbf{Z}})(\mathbf{e}_i-\bar{\mathbf{e}})^T, \mathbf{S}_{(\mathbf{X},\mathbf{Z})} = (1/n)\sum_{i=1}^n \begin{pmatrix} \mathbf{X}_i - \bar{\mathbf{X}} \\ \mathbf{Z}_i - \bar{\mathbf{Z}} \end{pmatrix} \begin{pmatrix} \mathbf{X}_i^T - \bar{\mathbf{X}}^T & \mathbf{Z}_i^T - \bar{\mathbf{Z}}^T \end{pmatrix}$. Note that $\mathbf{\Sigma}_{\mathbf{Xe}} = \mathbf{0}$ and $\mathbf{\Sigma}_{\mathbf{Ze}} = \mathbf{0}$. By Proposition 1 in Li et al. (2012), if $\mathbf{M} \in \mathbb{R}^{d_1\times d_2}$ and $\mathbf{N} \in \mathbb{R}^{d_2\times d_3}$, then

$$\|\mathbf{MN}\|_{\max} \le d_2\|\mathbf{M}\|_{\max}\|\mathbf{N}\|_{\max}. \tag{S25}$$

Since $\mathbf{S}_{(\mathbf{X},\mathbf{Z}),\mathbf{M}} = \mathbf{S}_{(\mathbf{X},\mathbf{Z})} \begin{pmatrix} \gamma_{\mathbf{X}} \\ \gamma_{\mathbf{Z}} \end{pmatrix} + \mathbf{S}_{(\mathbf{X},\mathbf{Z}),\mathbf{e}}$, then

$$\begin{aligned}
&\|\mathbf{S}_{(\mathbf{X},\mathbf{Z}),\mathbf{M}} - \mathbf{\Sigma}_{(\mathbf{X},\mathbf{Z}),\mathbf{M}}\|_{\max} \\
&\le \|\mathbf{S}_{(\mathbf{X},\mathbf{Z})} \begin{pmatrix} \gamma_{\mathbf{X}} \\ \gamma_{\mathbf{Z}} \end{pmatrix} - \mathbf{\Sigma}_{(\mathbf{X},\mathbf{Z})} \begin{pmatrix} \gamma_{\mathbf{X}} \\ \gamma_{\mathbf{Z}} \end{pmatrix} \|_{\max} + \|\mathbf{S}_{(\mathbf{X},\mathbf{Z}),\mathbf{e}} - \mathbf{\Sigma}_{(\mathbf{X},\mathbf{Z}),\mathbf{e}}\|_{\max} \\
&\le (k+q)\|\mathbf{S}_{(\mathbf{X},\mathbf{Z})} - \mathbf{\Sigma}_{(\mathbf{X},\mathbf{Z})}\|_{\max}\| \begin{pmatrix} \gamma_{\mathbf{X}} \\ \gamma_{\mathbf{Z}} \end{pmatrix} \|_{\max} + \|\mathbf{S}_{(\mathbf{X},\mathbf{Z}),\mathbf{e}} - \mathbf{\Sigma}_{(\mathbf{X},\mathbf{Z}),\mathbf{e}}\|_{\max} \\
&\le (k+q)\max(C_{\mathbf{X}}, C_{\mathbf{Z}})\|\mathbf{S}_{(\mathbf{X},\mathbf{Z})} - \mathbf{\Sigma}_{(\mathbf{X},\mathbf{Z})}\|_{\max} + \|\mathbf{S}_{(\mathbf{X},\mathbf{Z}),\mathbf{e}} - \mathbf{\Sigma}_{(\mathbf{X},\mathbf{Z}),\mathbf{e}}\|_{\max}.
\end{aligned}$$

So there exists a positive constant C_5 such that $\|\mathbf{S}_{(\mathbf{X},\mathbf{Z}),\mathbf{M}} - \mathbf{\Sigma}_{(\mathbf{X},\mathbf{Z}),\mathbf{M}}\|_{\max} \le C_5\{\log(p_n)/n\}^{1/2}$. Since

$$\mathbf{S}_{\mathbf{M}} = \begin{pmatrix} \gamma_{\mathbf{X}}^T & \gamma_{\mathbf{Z}}^T \end{pmatrix} \mathbf{S}_{(\mathbf{X},\mathbf{Z})} \begin{pmatrix} \gamma_{\mathbf{X}} \\ \gamma_{\mathbf{Z}} \end{pmatrix} + \begin{pmatrix} \gamma_{\mathbf{X}}^T & \gamma_{\mathbf{Z}}^T \end{pmatrix} \mathbf{S}_{(\mathbf{X},\mathbf{Z}),\mathbf{e}} + \mathbf{S}_{\mathbf{e},(\mathbf{X},\mathbf{Z})} \begin{pmatrix} \gamma_{\mathbf{X}} \\ \gamma_{\mathbf{Z}} \end{pmatrix} + \mathbf{S}_{\mathbf{e}},$$

then

$$\begin{aligned}
&\|\mathbf{S}_{\mathbf{M}} - \mathbf{\Sigma}_{\mathbf{M}}\|_{\max} \\
&\leq \quad (k+q)^2 \| \begin{pmatrix} \gamma_{\mathbf{X}}^T & \gamma_{\mathbf{Z}}^T \end{pmatrix} \|_{\max} \|\mathbf{S}_{(\mathbf{X},\mathbf{Z})} - \mathbf{\Sigma}_{(\mathbf{X},\mathbf{Z})}\|_{\max} \| \begin{pmatrix} \gamma_{\mathbf{X}} \\ \gamma_{\mathbf{Z}} \end{pmatrix} \|_{\max} \\
&\quad\quad +2(k+q) \| \begin{pmatrix} \gamma_{\mathbf{X}}^T & \gamma_{\mathbf{Z}}^T \end{pmatrix} \|_{\max} \|\mathbf{S}_{(\mathbf{X},\mathbf{Z}),\mathbf{e}} - \mathbf{\Sigma}_{(\mathbf{X},\mathbf{Z}),\mathbf{e}}\|_{\max} + \|\mathbf{S}_{\mathbf{e}} - \mathbf{\Sigma}_{\mathbf{e}}\|_{\max} \\
&\leq \quad (k+q)^2 \{\max(C_{\mathbf{X}}, C_{\mathbf{Z}})\}^2 \|\mathbf{S}_{(\mathbf{X},\mathbf{Z})} - \mathbf{\Sigma}_{(\mathbf{X},\mathbf{Z})}\|_{\max} \\
&\quad\quad +2(k+q) \max(C_{\mathbf{X}}, C_{\mathbf{X}}) \|\mathbf{S}_{(\mathbf{X},\mathbf{Z}),\mathbf{e}} - \mathbf{\Sigma}_{(\mathbf{X},\mathbf{Z}),\mathbf{e}}\|_{\max} + \|\mathbf{S}_{\mathbf{e}} - \mathbf{\Sigma}_{\mathbf{e}}\|_{\max}.
\end{aligned}$$

So there exists a positive constant C_6 such that $\|\mathbf{S}_{\mathbf{M}} - \mathbf{\Sigma}_{\mathbf{M}}\|_{\max} \leq C_6 \{\log(p_n)/n\}^{1/2}$. Because $\mathbf{S}_{\mathbf{M}|\mathbf{X},\mathbf{Z}} = \mathbf{S}_{\mathbf{M}} - \mathbf{S}_{\mathbf{M},(\mathbf{X},\mathbf{Z})} \mathbf{S}_{(\mathbf{X},\mathbf{Z})}^{-1} \mathbf{S}_{(\mathbf{X},\mathbf{Z}),\mathbf{M}}$, we have $\mathbf{S}_{\mathbf{M}|\mathbf{X},\mathbf{Z}} - \mathbf{\Sigma}_{\mathbf{M}|\mathbf{X},\mathbf{Z}} = (\mathbf{S}_{\mathbf{M}} - \mathbf{\Sigma}_{\mathbf{M}}) - (\mathbf{S}_{\mathbf{M},(\mathbf{X},\mathbf{Z})} - \mathbf{\Sigma}_{\mathbf{M},(\mathbf{X},\mathbf{Z})}) \mathbf{\Sigma}_{(\mathbf{X},\mathbf{Z})}^{-1} \mathbf{\Sigma}_{(\mathbf{X},\mathbf{Z}),\mathbf{M}} - \mathbf{S}_{\mathbf{M},(\mathbf{X},\mathbf{Z})} (\mathbf{S}_{(\mathbf{X},\mathbf{Z})}^{-1} - \mathbf{\Sigma}_{(\mathbf{X},\mathbf{Z})}^{-1}) \mathbf{\Sigma}_{(\mathbf{X},\mathbf{Z}),\mathbf{M}} - \mathbf{\Sigma}_{\mathbf{M},(\mathbf{X},\mathbf{Z})} \mathbf{\Sigma}_{(\mathbf{X},\mathbf{Z})}^{-1} (\mathbf{S}_{(\mathbf{X},\mathbf{Z}),\mathbf{M}} - \mathbf{\Sigma}_{(\mathbf{X},\mathbf{Z}),\mathbf{M}}) - \mathbf{\Sigma}_{\mathbf{M},(\mathbf{X},\mathbf{Z})} (\mathbf{S}_{(\mathbf{X},\mathbf{Z})}^{-1} - \mathbf{\Sigma}_{(\mathbf{X},\mathbf{Z})}^{-1}) (\mathbf{S}_{(\mathbf{X},\mathbf{Z}),\mathbf{M}} - \mathbf{\Sigma}_{(\mathbf{X},\mathbf{Z}),\mathbf{M}}) - (\mathbf{S}_{\mathbf{M},(\mathbf{X},\mathbf{Z})} - \mathbf{\Sigma}_{\mathbf{M},(\mathbf{X},\mathbf{Z})}) (\mathbf{S}_{(\mathbf{X},\mathbf{Z})}^{-1} - \mathbf{\Sigma}_{(\mathbf{X},\mathbf{Z})}^{-1}) \mathbf{\Sigma}_{(\mathbf{X},\mathbf{Z}),\mathbf{M}} - (\mathbf{S}_{\mathbf{M},(\mathbf{X},\mathbf{Z})} - \mathbf{\Sigma}_{\mathbf{M},(\mathbf{X},\mathbf{Z})}) \mathbf{\Sigma}_{(\mathbf{X},\mathbf{Z})}^{-1} (\mathbf{S}_{(\mathbf{X},\mathbf{Z}),\mathbf{M}} - \mathbf{\Sigma}_{(\mathbf{X},\mathbf{Z}),\mathbf{M}}) - (\mathbf{S}_{\mathbf{M},(\mathbf{X},\mathbf{Z})} - \mathbf{\Sigma}_{\mathbf{M},(\mathbf{X},\mathbf{Z})}) (\mathbf{S}_{(\mathbf{X},\mathbf{Z})}^{-1} - \mathbf{S}_{(\mathbf{X},\mathbf{Z})}^{-1}) (\mathbf{S}_{(\mathbf{X},\mathbf{Z}),\mathbf{M}} -$

$\boldsymbol{\Sigma}_{(\mathbf{X},\mathbf{Z}),\mathbf{M}})$. By repeatedly using (S25) in the expansion of $\mathbf{S}_{\mathbf{M}|\mathbf{X},\mathbf{Z}} - \boldsymbol{\Sigma}_{\mathbf{M}|\mathbf{X},\mathbf{Z}}$, there exists a positive integer C_1 such that $\|\mathbf{S}_{\mathbf{M}|\mathbf{X},\mathbf{Z}} - \boldsymbol{\Sigma}_{\mathbf{M}|\mathbf{X},\mathbf{Z}}\|_{\max} \le C_1\{\log(p_n)/n\}^{1/2}$. Similarly, we can prove that $\|\mathbf{S}_{\mathbf{M}|\mathbf{Y},\mathbf{X},\mathbf{Z}} - \boldsymbol{\Sigma}_{\mathbf{M}|\mathbf{Y},\mathbf{X},\mathbf{Z}}\|_{\max} \le C_2\{\log(p_n)/n\}^{1/2}$. Then we have established (S20) and (S21). Let $a_n = \sqrt{(p_n+s)\log(p_n)/n}$. Now we show $\|\hat{\mathbf{A}} - \mathbf{A}\|_F = O_p(\sqrt{(p_n+s)\log(p_n)/n})$. We denote the objective function in (S4) as $f_{obj,2}$. It is sufficient to show that for any small $\epsilon > 0$, ther exists a sufficiently large constant C such that

$$\lim_{n\to\infty} \Pr\Big(\inf_{\substack{\boldsymbol{\Delta}\in\mathbb{R}^{(p_n-d)\times d},\\ \|\boldsymbol{\Delta}\|_F = C}} f_{obj,2}(\mathbf{A} + a_n\boldsymbol{\Delta}) > f_{obj,2}(\mathbf{A})\Big) > 1-\epsilon. \tag{S26}$$

Let $\boldsymbol{\Delta}_* = (\mathbf{0}_{d\times d}, \boldsymbol{\Delta}^T)^T \in \mathbb{R}^{p_n\times d}$. Following the calculations as in Theorem 6, we compute the Taylor expansion of $f_{obj,2}(\mathbf{A}+a_n\boldsymbol{\Delta})$ at $\mathbf{A}$, and get $f_{obj,2}(\mathbf{A}+a_n\boldsymbol{\Delta}) - f_{obj,2}(\mathbf{A}) \ge 2a_n\,\mathrm{tr}[(\mathbf{G}_{\mathbf{A}}^T\boldsymbol{\Sigma}_{\mathbf{M}|\mathbf{Y},\mathbf{X},\mathbf{Z}}\mathbf{G}_{\mathbf{A}})^{-1}\mathbf{G}_{\mathbf{A}}^T(\mathbf{S}_{\mathbf{M}|\mathbf{Y},\mathbf{X},\mathbf{Z},sp} - \boldsymbol{\Sigma}_{\mathbf{M}|\mathbf{Y},\mathbf{X},\mathbf{Z}})\boldsymbol{\Delta}_* + \{(\mathbf{G}_{\mathbf{A}}^T\mathbf{S}_{\mathbf{M}|\mathbf{Y},\mathbf{X},\mathbf{Z},sp}\mathbf{G}_{\mathbf{A}})^{-1} - (\mathbf{G}_{\mathbf{A}}^T\boldsymbol{\Sigma}_{\mathbf{M}|\mathbf{Y},\mathbf{X},\mathbf{Z}}\mathbf{G}_{\mathbf{A}})^{-1}\}\mathbf{G}_{\mathbf{A}}^T\boldsymbol{\Sigma}_{\mathbf{M}|\mathbf{Y},\mathbf{X},\mathbf{Z}}\boldsymbol{\Delta}_* + \{(\mathbf{G}_{\mathbf{A}}^T\mathbf{S}_{\mathbf{M}|\mathbf{Y},\mathbf{X},\mathbf{Z},sp}\mathbf{G}_{\mathbf{A}})^{-1} - (\mathbf{G}_{\mathbf{A}}^T\boldsymbol{\Sigma}_{\mathbf{M}|\mathbf{Y},\mathbf{X},\mathbf{Z}}\mathbf{G}_{\mathbf{A}})^{-1}\}\mathbf{G}_{\mathbf{A}}^T (\mathbf{S}_{\mathbf{M}|\mathbf{Y},\mathbf{X},\mathbf{Z},sp} - \boldsymbol{\Sigma}_{\mathbf{M}|\mathbf{Y},\mathbf{X},\mathbf{Z}})\boldsymbol{\Delta}_*] + 2a_n\,\mathrm{tr}[(\mathbf{G}_{\mathbf{A}}^T\boldsymbol{\Sigma}_{\mathbf{M}|\mathbf{Y},\mathbf{X}}^{-1}\mathbf{G}_{\mathbf{A}})^{-1}\mathbf{G}_{\mathbf{A}}^T\,(\mathbf{S}_{\mathbf{M}|\mathbf{X},\mathbf{Z},sp}^{-1} - \boldsymbol{\Sigma}_{\mathbf{M}|\mathbf{X},\mathbf{Z}}^{-1})\boldsymbol{\Delta}_* + \{(\mathbf{G}_{\mathbf{A}}^T\mathbf{S}_{\mathbf{M}|\mathbf{X},\mathbf{Z},sp}^{-1}\mathbf{G}_A)^{-1} - (\mathbf{G}_{\mathbf{A}}^T\boldsymbol{\Sigma}_{\mathbf{M}|\mathbf{X},\mathbf{Z}}^{-1}\mathbf{G}_A)^{-1}\}\mathbf{G}_{\mathbf{A}}^T\boldsymbol{\Sigma}_{\mathbf{M}|\mathbf{X},\mathbf{Z}}^{-1}\boldsymbol{\Delta}_* + \{(\mathbf{G}_{\mathbf{A}}^T\mathbf{S}_{\mathbf{M}|\mathbf{X},\mathbf{Z},sp}^{-1}\mathbf{G}_{\mathbf{A}})^{-1} - (\mathbf{G}_{\mathbf{A}}^T\boldsymbol{\Sigma}_{\mathbf{M}|\mathbf{X},\mathbf{Z}}^{-1}\mathbf{G}_{\mathbf{A}})^{-1}\}\mathbf{G}_{\mathbf{A}}^T(\mathbf{S}_{\mathbf{M}|\mathbf{X},\mathbf{Z},sp}^{-1} - \boldsymbol{\Sigma}_{\mathbf{M}|\mathbf{X},\mathbf{Z}}^{-1})\boldsymbol{\Delta}_*] + a_n^2\,\mathrm{tr}\{(\boldsymbol{\Omega}^{-1} + \boldsymbol{\eta}\boldsymbol{\Sigma}_{\mathbf{Y}|\mathbf{M},\mathbf{X},\mathbf{Z}}^{-1}\boldsymbol{\eta}^T)\boldsymbol{\Gamma}_1^T\boldsymbol{\Delta}_*^T\boldsymbol{\Gamma}_0\boldsymbol{\Omega}_0\boldsymbol{\Gamma}_0^T\boldsymbol{\Delta}_*\boldsymbol{\Gamma}_1 + \boldsymbol{\Omega}\boldsymbol{\Gamma}_1^T\boldsymbol{\Delta}_*^T\boldsymbol{\Gamma}_0\boldsymbol{\Omega}_0^{-1}\boldsymbol{\Gamma}_0^T\boldsymbol{\Delta}_*\boldsymbol{\Gamma}_1 - 2(\mathbf{I}_d + \mathbf{A}^T\mathbf{A})^{-1}\boldsymbol{\Delta}_*^T\boldsymbol{\Gamma}_0\boldsymbol{\Gamma}_0^T\boldsymbol{\Delta}_*\} - a_n^2(p_{\mathcal{A}} - d)a_n^{-1}\lambda_{\mathcal{A}}\max_{1\le i\le p_{\mathcal{A}}-d}\|\boldsymbol{\delta}_i\|_2 + o_p(a_n^2)$. Let $\|\cdot\|$ denote the spectral norm of a matrix. For any two matrices

$\mathbf{M} \in \mathbb{R}^{d_1 \times d_2}$ and $\mathbf{N} \in \mathbb{R}^{d_2 \times d_3}$,

$$\|\mathbf{M}\mathbf{N}\|_F \leq \|\mathbf{M}\|\|\mathbf{N}\|_F \tag{S27}$$

Then by (S27) and Cauchy-Schwartz inequality, the first term in the preceding display can be lower bound as follows $\mathrm{tr}\{(\mathbf{G}_{\mathbf{A}}^T \boldsymbol{\Sigma}_{\mathbf{M}|\mathbf{Y},\mathbf{X},\mathbf{Z}} \mathbf{G}_{\mathbf{A}})^{-1} \mathbf{G}_{\mathbf{A}}^T (\mathbf{S}_{\mathbf{M}|\mathbf{Y},\mathbf{X},\mathbf{Z},sp} - \boldsymbol{\Sigma}_{\mathbf{M}|\mathbf{Y},\mathbf{X},\mathbf{Z}}) \boldsymbol{\Delta}_*\} \geq -\|\mathbf{S}_{\mathbf{M}|\mathbf{Y},\mathbf{X},\mathbf{Z},sp} - \boldsymbol{\Sigma}_{\mathbf{M}|\mathbf{Y},\mathbf{X},\mathbf{Z}}\|_F \|\boldsymbol{\Delta}\|_F \|(\mathbf{G}_{\mathbf{A}}^T \boldsymbol{\Sigma}_{\mathbf{M}|\mathbf{Y},\mathbf{X},\mathbf{Z}} \mathbf{G}_{\mathbf{A}})^{-1}\| \|\mathbf{G}_{\mathbf{A}}\|$.
Now for the second term

$$\begin{aligned}
&\{(\mathbf{G}_{\mathbf{A}}^T \mathbf{S}_{\mathbf{M}|\mathbf{Y},\mathbf{X},\mathbf{Z},sp} \mathbf{G}_{\mathbf{A}})^{-1} - (\mathbf{G}_{\mathbf{A}}^T \boldsymbol{\Sigma}_{\mathbf{M}|\mathbf{Y},\mathbf{X},\mathbf{Z}} \mathbf{G}_{\mathbf{A}})^{-1}\} \mathbf{G}_{\mathbf{A}}^T \boldsymbol{\Sigma}_{\mathbf{M}|\mathbf{Y},\mathbf{X},\mathbf{Z}} \boldsymbol{\Delta}_* \\
&= -(\mathbf{G}_{\mathbf{A}}^T \boldsymbol{\Sigma}_{\mathbf{M}|\mathbf{Y},\mathbf{X},\mathbf{Z}} \mathbf{G}_{\mathbf{A}})^{-1} (\mathbf{G}_{\mathbf{A}}^T \mathbf{S}_{\mathbf{M}|\mathbf{Y},\mathbf{X},\mathbf{Z},sp} \mathbf{G}_{\mathbf{A}} \\
&\quad - \mathbf{G}_{\mathbf{A}}^T \boldsymbol{\Sigma}_{\mathbf{M}|\mathbf{Y},\mathbf{X},\mathbf{Z}} \mathbf{G}_{\mathbf{A}}) (\mathbf{G}_{\mathbf{A}}^T \boldsymbol{\Sigma}_{\mathbf{M}|\mathbf{Y},\mathbf{X},\mathbf{Z}} \mathbf{G}_{\mathbf{A}})^{-1} \mathbf{G}_{\mathbf{A}}^T \boldsymbol{\Sigma}_{\mathbf{M}|\mathbf{Y},\mathbf{X},\mathbf{Z}} \boldsymbol{\Delta}_* \\
&\quad + o_p(\mathbf{G}_{\mathbf{A}}^T \mathbf{S}_{\mathbf{M}|\mathbf{Y},\mathbf{X},\mathbf{Z},sp} \mathbf{G}_{\mathbf{A}} - \mathbf{G}_{\mathbf{A}}^T \boldsymbol{\Sigma}_{\mathbf{M}|\mathbf{Y},\mathbf{X},\mathbf{Z}} \mathbf{G}_{\mathbf{A}}) \\
&= (\mathbf{G}_{\mathbf{A}}^T \boldsymbol{\Sigma}_{\mathbf{M}|\mathbf{Y},\mathbf{X},\mathbf{Z}} \mathbf{G}_{\mathbf{A}})^{-1} \mathbf{G}_{\mathbf{A}}^T \boldsymbol{\Sigma}_{\mathbf{M}|\mathbf{Y},\mathbf{X},\mathbf{Z}} (\mathbf{S}_{\mathbf{M}|\mathbf{Y},\mathbf{X},\mathbf{Z},sp}^{-1} \\
&\quad - \boldsymbol{\Sigma}_{\mathbf{M}|\mathbf{Y},\mathbf{X},\mathbf{Z}}^{-1}) \boldsymbol{\Sigma}_{\mathbf{M}|\mathbf{Y},\mathbf{X},\mathbf{Z}} \mathbf{G}_{\mathbf{A}} (\mathbf{G}_{\mathbf{A}}^T \boldsymbol{\Sigma}_{\mathbf{M}|\mathbf{Y},\mathbf{X},\mathbf{Z}} \mathbf{G}_{\mathbf{A}})^{-1} \mathbf{G}_{\mathbf{A}}^T \boldsymbol{\Sigma}_{\mathbf{M}|\mathbf{Y},\mathbf{X},\mathbf{Z}} \boldsymbol{\Delta}_* + o_p(a_n),
\end{aligned}$$

the second term can be lower bounded as follows

$$\text{tr}[\{(\mathbf{G}_{\mathbf{A}}^T \mathbf{S}_{\mathbf{M}|\mathbf{Y},\mathbf{X},\mathbf{Z},sp}\mathbf{G}_{\mathbf{A}})^{-1} - (\mathbf{G}_{\mathbf{A}}^T \mathbf{\Sigma}_{\mathbf{M}|\mathbf{Y},\mathbf{X},\mathbf{Z}}\mathbf{G}_{\mathbf{A}})^{-1}\}\mathbf{G}_{\mathbf{A}}^T \mathbf{\Sigma}_{\mathbf{M}|\mathbf{Y},\mathbf{X},\mathbf{Z}}\mathbf{\Delta}_*]$$
$$\geq -\|\mathbf{S}_{\mathbf{M}|\mathbf{Y},\mathbf{X},\mathbf{Z},sp}^{-1} - \mathbf{\Sigma}_{\mathbf{M}|\mathbf{Y},\mathbf{X},\mathbf{Z}}^{-1}\|_F \|\mathbf{\Delta}\|_F \|\mathbf{P}_{\mathbf{G}_{\mathbf{A}}(\mathbf{\Sigma}_{\mathbf{M}|\mathbf{Y},\mathbf{X},\mathbf{Z}})}\}\|\mathbf{\Sigma}_{\mathbf{M}|\mathbf{Y},\mathbf{X},\mathbf{Z}}\|$$
$$\times \|(\mathbf{G}_{\mathbf{A}}^T \mathbf{\Sigma}_{\mathbf{M}|\mathbf{Y},\mathbf{X},\mathbf{Z}}\mathbf{G}_{\mathbf{A}})^{-1}\mathbf{G}_{\mathbf{A}}^T \mathbf{\Sigma}_{\mathbf{M}|\mathbf{Y},\mathbf{X},\mathbf{Z}}\|. \tag{S28}$$

Following the same derivatives and also using the fact that for any matrix $\mathbf{A}$, $\|\mathbf{A}\| \leq \|\mathbf{A}\|_F$, the third term can be lower bounded by

$$\{(\mathbf{G}_{\mathbf{A}}^T \mathbf{S}_{\mathbf{M}|\mathbf{Y},\mathbf{X},\mathbf{Z},sp}\mathbf{G}_{\mathbf{A}})^{-1} - (\mathbf{G}_{\mathbf{A}}^T \mathbf{\Sigma}_{\mathbf{M}|\mathbf{Y},\mathbf{X},\mathbf{Z}}\mathbf{G}_{\mathbf{A}})^{-1}\}\mathbf{G}_{\mathbf{A}}^T (\mathbf{S}_{\mathbf{M}|\mathbf{Y},\mathbf{X},\mathbf{Z},sp} - \mathbf{\Sigma}_{\mathbf{M}|\mathbf{Y},\mathbf{X},\mathbf{Z}})\mathbf{\Delta}_*$$
$$\geq -\|\mathbf{S}_{\mathbf{M}|\mathbf{Y},\mathbf{X},\mathbf{Z},sp}^{-1} - \mathbf{\Sigma}_{\mathbf{M}|\mathbf{Y},\mathbf{X},\mathbf{Z}}^{-1}\|_F \|\mathbf{S}_{\mathbf{M}|\mathbf{Y},\mathbf{X},\mathbf{Z},sp} - \mathbf{\Sigma}_{\mathbf{M}|\mathbf{Y},\mathbf{X},\mathbf{Z}}\|_F \|\mathbf{\Delta}\|_F \|\mathbf{P}_{\mathbf{G}_A(\mathbf{\Sigma}_{\mathbf{M}|\mathbf{Y},\mathbf{X},\mathbf{Z}})}\|$$
$$\times \|(\mathbf{G}_{\mathbf{A}}^T \mathbf{\Sigma}_{\mathbf{M}|\mathbf{Y},\mathbf{X},\mathbf{Z}}\mathbf{G}_{\mathbf{A}})^{-1}\mathbf{G}_{\mathbf{A}}^T \mathbf{\Sigma}_{\mathbf{M}|\mathbf{Y},\mathbf{X},\mathbf{Z}}\|. \tag{S29}$$

Based on the convergence rate of $\mathbf{S}_{\mathbf{M}|\mathbf{Y},\mathbf{X},\mathbf{Z},sp}$ and $\mathbf{S}_{\mathbf{M}|\mathbf{Y},\mathbf{X},\mathbf{Z},sp}^{-1}$, this lower bound is in the order of a_n^2. The bounds for the fourth till the sixth terms can be developed similarly. Let $m_1 = \|(\mathbf{G}_{\mathbf{A}}^T \mathbf{\Sigma}_{\mathbf{M}|\mathbf{Y},\mathbf{X},\mathbf{Z}}\mathbf{G}_{\mathbf{A}})^{-1}\|\|\mathbf{G}_{\mathbf{A}}\|$, $m_2 = \|\mathbf{P}_{\mathbf{G}_{\mathbf{A}}(\mathbf{\Sigma}_{\mathbf{M}|\mathbf{Y},\mathbf{X},\mathbf{Z}})}\|\|\mathbf{\Sigma}_{\mathbf{M}|\mathbf{Y},\mathbf{X},\mathbf{Z}}\|$ $\|(\mathbf{G}_{\mathbf{A}}^T \mathbf{\Sigma}_{\mathbf{M}|\mathbf{Y},\mathbf{X},\mathbf{Z}}\mathbf{G}_{\mathbf{A}})^{-1}\ \mathbf{G}_{\mathbf{A}}^T \mathbf{\Sigma}_{\mathbf{M}|\mathbf{Y},\mathbf{X},\mathbf{Z}}\|$, $m_3 = \|(\mathbf{G}_{\mathbf{A}}^T \mathbf{\Sigma}_{\mathbf{M}|\mathbf{X},\mathbf{Z}}^{-1}\mathbf{G}_{\mathbf{A}})^{-1}\|\|\mathbf{G}_{\mathbf{A}}\|$, and $m_4 = \|\mathbf{P}_{\mathbf{G}_{\mathbf{A}}(\mathbf{\Sigma}_{\mathbf{M}|\mathbf{X},\mathbf{Z}}^{-1})}\|\|\mathbf{\Sigma}_{\mathbf{M}|\mathbf{X},\mathbf{Z}}^{-1}\|\ \|(\mathbf{G}_{\mathbf{A}}^T \mathbf{\Sigma}_{\mathbf{M}|\mathbf{X},\mathbf{Z}}^{-1}\mathbf{G}_{\mathbf{A}})^{-1}\mathbf{G}_{\mathbf{A}}^T \mathbf{\Sigma}_{\mathbf{M}|\mathbf{X},\mathbf{Z}}^{-1}\|$. We also notice that since $\lambda_{\mathcal{A}} = o(a_n)$, then $a_n^{-1}\lambda_{\mathcal{A}} = o(1)$, and $a_n^2(p_{\mathcal{A}} - d) = a_n^{-1}\lambda_{\mathcal{A}} \max_{1\leq i\leq p_{\mathcal{A}}-d} \|\boldsymbol{\delta}_i\|_2 =$

$o_p(a_n^2)$. Collecting all these bounds and results together, we have

$$\begin{aligned} & f_{obj,2}(\mathbf{A}+a_n\boldsymbol{\Delta}) - f_{obj,2}(\mathbf{A}) \\ \geq\ & -2a_n(m_1\|\mathbf{S}_{\mathbf{M}|\mathbf{Y},\mathbf{X},\mathbf{Z},sp} - \boldsymbol{\Sigma}_{\mathbf{M}|\mathbf{Y},\mathbf{X},\mathbf{Z}}\|_F\|\boldsymbol{\Delta}\|_F + m_2\|\mathbf{S}^{-1}_{\mathbf{M}|\mathbf{Y},\mathbf{X},\mathbf{Z},sp} - \boldsymbol{\Sigma}^{-1}_{\mathbf{M}|\mathbf{Y},\mathbf{X},\mathbf{Z}}\|_F\|\boldsymbol{\Delta}\|_F \\ & +m_3\|\mathbf{S}^{-1}_{\mathbf{M}|\mathbf{X},\mathbf{Z},sp} - \boldsymbol{\Sigma}^{-1}_{\mathbf{M}|\mathbf{X},\mathbf{Z}}\|_F\|\boldsymbol{\Delta}\|_F + m_4\|\mathbf{S}_{\mathbf{M}|\mathbf{X},\mathbf{Z},sp} - \boldsymbol{\Sigma}_{\mathbf{M}|\mathbf{X},\mathbf{Z}}\|_F\|\boldsymbol{\Delta}\|_F) \\ & +a_n^2\,\mathrm{tr}\{(\boldsymbol{\Omega}^{-1} + \boldsymbol{\eta}\boldsymbol{\Sigma}^{-1}_{\mathbf{Y}|\mathbf{M},\mathbf{X},\mathbf{Z}}\boldsymbol{\eta}^T)\boldsymbol{\Gamma}_1^T\boldsymbol{\Delta}_*^T\boldsymbol{\Gamma}_0\boldsymbol{\Omega}_0\boldsymbol{\Gamma}_0^T\boldsymbol{\Delta}_*\boldsymbol{\Gamma}_1 + \boldsymbol{\Omega}\boldsymbol{\Gamma}_1^T\boldsymbol{\Delta}_*^T\boldsymbol{\Gamma}_0\boldsymbol{\Omega}_0^{-1}\boldsymbol{\Gamma}_0^T\boldsymbol{\Delta}_*\boldsymbol{\Gamma}_1 \\ & -2(\mathbf{I}_d + \mathbf{A}^T\mathbf{A})^{-1}\boldsymbol{\Delta}_*^T\boldsymbol{\Gamma}_0\boldsymbol{\Gamma}_0^T\boldsymbol{\Delta}_*^T\} + o_p(a_n^2). \end{aligned}$$

According to the proof at the end of Theorem 6, there exists a positive constant k such that $\mathrm{tr}\{(\boldsymbol{\Omega}^{-1}+\boldsymbol{\eta}\boldsymbol{\Sigma}^{-1}_{\mathbf{Y}|\mathbf{M},\mathbf{X},\mathbf{Z}}\boldsymbol{\eta}^T)\boldsymbol{\Gamma}_1^T\boldsymbol{\Delta}_*^T\boldsymbol{\Gamma}_0\boldsymbol{\Omega}_0\boldsymbol{\Gamma}_0^T\boldsymbol{\Delta}_*\boldsymbol{\Gamma}_1+\boldsymbol{\Omega}\boldsymbol{\Gamma}_1^T\boldsymbol{\Delta}_*^T\boldsymbol{\Gamma}_0\boldsymbol{\Omega}_0^{-1}\boldsymbol{\Gamma}_0\boldsymbol{\Delta}_*\boldsymbol{\Gamma}_1- 2(\mathbf{I}_d+\mathbf{A}^T\mathbf{A})^{-1}\boldsymbol{\Delta}_*^T\boldsymbol{\Gamma}_0\boldsymbol{\Gamma}_0^T\boldsymbol{\Delta}_*^T\} \geq k\|\boldsymbol{\Delta}\|_F^2$. Then with sufficiently large C, the second order term of $\|\boldsymbol{\Delta}\|_F$ dominates the first order term, and $f_{obj,2}(\mathbf{A}+a_n\boldsymbol{\Delta}) - f_{obj,2}(\mathbf{A}) > 0$ with probability tending to 1. Then we have established (S26). So $\|\hat{\mathbf{A}}-\mathbf{A}\|_F = O_p(a_n)$, where $a_n = \sqrt{(p_n+s)\log(p_n)/n}$. Since $\widehat{\boldsymbol{\beta}} = \widehat{\boldsymbol{\Gamma}}(\widehat{\boldsymbol{\Gamma}}^T\mathbf{S}_{\mathbf{M}|\mathbf{X},\mathbf{Z},sp}\widehat{\boldsymbol{\Gamma}})^{-1} \widehat{\boldsymbol{\Gamma}}^T\mathbf{S}_{(\mathbf{M},\mathbf{Y})|\mathbf{X},\mathbf{Z}} = \mathbf{P}_{\hat{\mathbf{G}}_{\mathbf{A}}(\mathbf{S}_{\mathbf{M}|\mathbf{X},\mathbf{Z},sp})}\mathbf{S}^{-1}_{\mathbf{M}|\mathbf{X},\mathbf{Z},sp}\mathbf{S}_{(\mathbf{M},\mathbf{Y})|\mathbf{X},\mathbf{Z}}$, $\|\widehat{\boldsymbol{\beta}}_{\mathbf{M}}-\boldsymbol{\beta}_{\mathbf{M}}\|_F = \|\mathbf{P}_{\hat{\mathbf{G}}_{\mathbf{A}}(\mathbf{S}_{\mathbf{M}|\mathbf{X},\mathbf{Z},sp})} (\mathbf{S}^{-1}_{\mathbf{M}|\mathbf{X},\mathbf{Z},sp}\mathbf{S}_{(\mathbf{M},\mathbf{Y})|\mathbf{X},\mathbf{Z}}-\boldsymbol{\Sigma}^{-1}_{\mathbf{M}|\mathbf{X},\mathbf{Z}}\boldsymbol{\Sigma}_{(\mathbf{M},\mathbf{Y})|\mathbf{X},\mathbf{Z}})\|_F+\|(\mathbf{P}_{\hat{\mathbf{G}}_{\mathbf{A}}(\mathbf{S}_{\mathbf{M}|\mathbf{X},\mathbf{Z},sp})}-\mathbf{P}_{\mathbf{G}_{\mathbf{A}}(\boldsymbol{\Sigma}_{\mathbf{M}|\mathbf{X},\mathbf{Z}})})\boldsymbol{\Sigma}^{-1}_{\mathbf{M}|\mathbf{X},\mathbf{Z}} \boldsymbol{\Sigma}_{(\mathbf{M},\mathbf{Y})|\mathbf{X},\mathbf{Z}}\|_F$. Since $\|\mathbf{P}_{\hat{\mathbf{G}}_{\mathbf{A}}(\mathbf{S}_{\mathbf{M}|\mathbf{X},\mathbf{Z},sp})}\| = 1$ and $\mathbf{S}_{(\mathbf{M},\mathbf{Y})|\mathbf{X},\mathbf{Z}}$ is a $\sqrt{n}$-consistent estimator of $\boldsymbol{\Sigma}_{(\mathbf{M},\mathbf{Y})|\mathbf{X},\mathbf{Z}}$, then

$$
\begin{aligned}
&\mathbf{P}_{\hat{\mathbf{G}}_{\mathbf{A}}(\mathbf{S}_{\mathbf{M}|\mathbf{X},\mathbf{Z},sp})}(\mathbf{S}^{-1}_{\mathbf{M}|\mathbf{X},\mathbf{Z},sp}\mathbf{S}_{(\mathbf{M},\mathbf{Y})|\mathbf{X},\mathbf{Z}} - \boldsymbol{\Sigma}^{-1}_{\mathbf{M}|\mathbf{X},\mathbf{Z}}\boldsymbol{\Sigma}_{(\mathbf{M},\mathbf{Y})|\mathbf{X},\mathbf{Z}})\|_F \\
&\quad \leq \ \|\mathbf{S}^{-1}_{\mathbf{M}|\mathbf{X},\mathbf{Z},sp}\mathbf{S}_{(\mathbf{M},\mathbf{Y})|\mathbf{X},\mathbf{Z}} - \boldsymbol{\Sigma}^{-1}_{\mathbf{M}|\mathbf{X},\mathbf{Z}}\boldsymbol{\Sigma}_{(\mathbf{M},\mathbf{Y})|\mathbf{X},\mathbf{Z}}\|_F \\
&\quad \leq \ \|(\mathbf{S}^{-1}_{\mathbf{M}|\mathbf{X},\mathbf{Z},sp} - \boldsymbol{\Sigma}^{-1}_{\mathbf{M}|\mathbf{X},\mathbf{Z}})\boldsymbol{\Sigma}_{(\mathbf{M},\mathbf{Y})|\mathbf{X},\mathbf{Z}}\|_F + \|\boldsymbol{\Sigma}^{-1}_{\mathbf{M}|\mathbf{X},\mathbf{Z}}(\mathbf{S}_{(\mathbf{M},\mathbf{Y})|\mathbf{X},\mathbf{Z}} - \boldsymbol{\Sigma}_{(\mathbf{M},\mathbf{Y})|\mathbf{X},\mathbf{Z}})\|_F \\
&\qquad + \|(\mathbf{S}^{-1}_{\mathbf{M}|\mathbf{X},\mathbf{Z},sp} - \boldsymbol{\Sigma}^{-1}_{\mathbf{M}|\mathbf{X},\mathbf{Z}})(\mathbf{S}_{(\mathbf{M},\mathbf{Y})|\mathbf{X},\mathbf{Z}} - \boldsymbol{\Sigma}_{(\mathbf{M},\mathbf{Y})|\mathbf{X},\mathbf{Z}})\|_F = O_p(a_n),
\end{aligned}
$$

and $\|(\mathbf{P}_{\hat{\mathbf{G}}_{\mathbf{A}}(\mathbf{S}_{\mathbf{M}|\mathbf{X},\mathbf{Z},sp})} - \mathbf{P}_{\mathbf{G}_{\mathbf{A}}(\boldsymbol{\Sigma}_{\mathbf{M}|\mathbf{X},\mathbf{Z}})})\boldsymbol{\Sigma}^{-1}_{\mathbf{M}|\mathbf{X},\mathbf{Z}}\boldsymbol{\Sigma}_{(\mathbf{M},\mathbf{Y})|\mathbf{X},\mathbf{Z}}\|_F \leq \|\mathbf{P}_{\hat{\mathbf{G}}_{\mathbf{A}}(\mathbf{S}_{\mathbf{M}|\mathbf{X},\mathbf{Z},sp})} - \mathbf{P}_{\mathbf{G}_{\mathbf{A}}(\boldsymbol{\Sigma}_{\mathbf{M}|\mathbf{X},\mathbf{Z}})}\|\|\boldsymbol{\beta}\|_F$. Because $\|\mathbf{S}_{\mathbf{M}|\mathbf{X},\mathbf{Z},sp} - \boldsymbol{\Sigma}_{\mathbf{M}|\mathbf{X},\mathbf{Z}}\|_F = O_p(a_n)$ and $\|\hat{\mathbf{A}} - \mathbf{A}\|_F = O_p(a_n)$, then $\mathbf{P}_{\hat{\mathbf{G}}_{\mathbf{A}}(\mathbf{S}_{\mathbf{M}|\mathbf{X},\mathbf{Z},sp})} - \mathbf{P}_{\mathbf{G}_{\mathbf{A}}(\boldsymbol{\Sigma}_{\mathbf{M}|\mathbf{X},\mathbf{Z}})}\| = O_p(a_n)$. So we have $\|\widehat{\boldsymbol{\beta}}_{\mathbf{M}} - \boldsymbol{\beta}_{\mathbf{M}}\|_F = O_p(a_n)$.

Proof of Web Theorem 7 Because of the consistency results in Web Theorem 6 that $\|\hat{\mathbf{A}} - \mathbf{A}\|_F = O_p(\sqrt{(p_n + s)\log(p_n)/n})$, then $\hat{\mathbf{a}}_i, i = 1, \ldots, p_{\mathcal{A}} - d$, converges to $\mathbf{a}_i$ with rate $\sqrt{(p_n + s)\log(p_n)/n}$. Therefore, $\Pr(\hat{\mathbf{a}}_i \neq \mathbf{0}) \to 1$ for $i = 1, \ldots, p_{\mathcal{A}} - d$.

Now, we prove the selection consistency $\Pr(\hat{\mathbf{a}}_i = \mathbf{0}, i = p_{\mathcal{A}} - d + 1, \ldots, p_n -$

$d) \to 1$. Let $f_{obj,2}$ be the objective function for $\mathbf{A}$ defined in (S4). Let

$$\mathbf{A}_{\mathcal{A}} = \begin{pmatrix} \mathbf{a}_1 \\ \vdots \\ \mathbf{a}_{p_{\mathcal{A}}-d} \end{pmatrix} \in \mathbb{R}^{(p_{\mathcal{A}-d})\times d} \qquad \text{and} \qquad \mathbf{A}_{\mathcal{I}} = \begin{pmatrix} \mathbf{a}_{p_{\mathcal{A}}-d+1} \\ \vdots \\ \mathbf{a}_{p_n-d} \end{pmatrix} \in \mathbb{R}^{(p_{\mathcal{I}})\times d}.$$

Then $\mathbf{A} = (\mathbf{A}_{\mathcal{A}}^T, \mathbf{A}_{\mathcal{I}}^T)^T$. Let $a_n = \sqrt{(p_n+s)\log(p_n)/n}$, $\hat{\mathbf{A}}_0 = \begin{pmatrix} \hat{\mathbf{A}}_{\mathcal{A}} \\ \mathbf{0} \end{pmatrix} \in \mathbb{R}^{(p_n-d)\times d}$, and $\boldsymbol{\Delta}_0 = \begin{pmatrix} \mathbf{0} \\ \hat{\mathbf{A}}_{\mathcal{I}} \end{pmatrix} \in \mathbb{R}^{(p_n-d)\times d}$. We need to show that for any constant $C > 0$, if $\|\hat{\mathbf{A}}_{\mathcal{A}} - \mathbf{A}_{\mathcal{A}}\|_F = O_p(a_n)$, then

$$\lim_{n\to\infty} \Pr\Big(\inf_{\substack{\|\hat{\mathbf{A}}_{\mathcal{I}}\|_F \le a_n C, \\ \hat{\mathbf{A}}_{\mathcal{I}} \neq \mathbf{0}}} f_{obj,2}(\hat{\mathbf{A}}_0 + \boldsymbol{\Delta}_0) > f_{obj,2}(\hat{\mathbf{A}}_0)\Big) > 1 - \epsilon, \tag{S30}$$

for any small $\epsilon > 0$. Let $\boldsymbol{\Delta} = \boldsymbol{\Delta}_0/a_n$, then $\|\boldsymbol{\Delta}\|_F \le C$. Let $\boldsymbol{\Delta}_* = (\mathbf{0}_{d\times d}, \boldsymbol{\Delta}^T)^T \in \mathbb{R}^{p_n \times d}$. Using the Taylor expansion of $f_{obj,2}(\hat{\mathbf{A}}_0 + a_n\boldsymbol{\Delta})$ at $\hat{\mathbf{A}}_0$, we have

$$
\begin{aligned}
& f_{obj,2}(\hat{\mathbf{A}}_0 + a_n\boldsymbol{\Delta}) - f_{obj,2}(\hat{\mathbf{A}}_0) \\
&= 2a_n \operatorname{tr}[(\mathbf{G}_{\mathbf{A}}^T\mathbf{S}_{\mathbf{M}|\mathbf{Y},\mathbf{X},\mathbf{Z},sp}\mathbf{G}_{\mathbf{A}})^{-1}\mathbf{G}_{\mathbf{A}}^T\mathbf{S}_{\mathbf{M}|\mathbf{Y},\mathbf{X},\mathbf{Z},sp}\boldsymbol{\Delta}_* + (\mathbf{G}_{\mathbf{A}}^T\mathbf{S}_{\mathbf{M}|\mathbf{X},\mathbf{Z},sp}^{-1}\mathbf{G}_{\mathbf{A}})^{-1}\mathbf{G}_{\mathbf{A}}^T\mathbf{S}_{\mathbf{M}|\mathbf{X},\mathbf{Z},sp}^{-1}\boldsymbol{\Delta}_* \\
&\quad -2(\mathbf{I}_d + \mathbf{A}^T\mathbf{A})^{-1}\mathbf{A}^T\boldsymbol{\Delta}] + a_n^2 \operatorname{tr}[(\boldsymbol{\Omega}^{-1} + \boldsymbol{\eta}\boldsymbol{\Sigma}_{\mathbf{Y}|\mathbf{M},\mathbf{X},\mathbf{Z}}^{-1}\boldsymbol{\eta}^T)\boldsymbol{\Gamma}_1^T\boldsymbol{\Delta}_*^T\boldsymbol{\Gamma}_0\boldsymbol{\Omega}_0\boldsymbol{\Gamma}_0^T\boldsymbol{\Delta}_*\boldsymbol{\Gamma}_1 \\
&\quad +\boldsymbol{\Omega}\boldsymbol{\Gamma}_1^T\boldsymbol{\Delta}_*^T\boldsymbol{\Gamma}_0\boldsymbol{\Omega}_0^{-1}\boldsymbol{\Gamma}_0^T\boldsymbol{\Delta}_*\boldsymbol{\Gamma}_1 - 2(\mathbf{I}_d + \mathbf{A}^T\mathbf{A})^{-1}\boldsymbol{\Delta}_*^T\boldsymbol{\Gamma}_0\boldsymbol{\Gamma}_0^T\boldsymbol{\Delta}_*] + \lambda \sum_{i=p_{\mathcal{A}}-d+1}^{p_n-d} w_i\|\hat{\mathbf{a}}_i\|_2 + o_p(a_n^2).
\end{aligned}
$$

First, $\lambda \sum_{i=p_{\mathcal{A}}-d+1}^{p_n-d} w_i\|\hat{\mathbf{a}}_i\|_2 \geq \lambda_{\mathcal{I}} \sum_{i=p_{\mathcal{A}}-d+1}^{p_n-d} \|\hat{\mathbf{a}}_i\|_2$. Using equations (S20) and (S21), $\mathbf{S}_{\mathbf{M}|\mathbf{X},\mathbf{Z},sp}^{-1} = \boldsymbol{\Sigma}_{\mathbf{M}|\mathbf{X},\mathbf{Z}}^{-1} + O_p(a_n)$ and $\mathbf{S}_{\mathbf{M}|\mathbf{Y},\mathbf{X},\mathbf{Z},sp}^{-1} = \boldsymbol{\Sigma}_{\mathbf{M}|\mathbf{Y},\mathbf{X},\mathbf{Z}}^{-1} + O_p(a_n)$, $\operatorname{tr}[(\mathbf{G}_{\mathbf{A}}^T\mathbf{S}_{\mathbf{M}|\mathbf{Y},\mathbf{X},\mathbf{Z},sp}\mathbf{G}_{\mathbf{A}})^{-1}\mathbf{G}_{\mathbf{A}}^T\mathbf{S}_{\mathbf{M}|\mathbf{Y},\mathbf{X},\mathbf{Z},sp}\boldsymbol{\Delta}_* + (\mathbf{G}_{\mathbf{A}}^T\mathbf{S}_{\mathbf{M}|\mathbf{X},\mathbf{Z},sp}^{-1}\mathbf{G}_{\mathbf{A}})^{-1}\, \mathbf{G}_{\mathbf{A}}^T\mathbf{S}_{\mathbf{M}|\mathbf{X},\mathbf{Z},sp}^{-1}\boldsymbol{\Delta}_* - 2(\mathbf{I}_d + \mathbf{A}^T\mathbf{A})^{-1}\mathbf{A}^T\boldsymbol{\Delta}] = \operatorname{tr}[(\mathbf{G}_{\mathbf{A}}^T\boldsymbol{\Sigma}_{\mathbf{M}|\mathbf{Y},\mathbf{X},\mathbf{Z}}\mathbf{G}_{\mathbf{A}})^{-1}\mathbf{G}_{\mathbf{A}}^T\boldsymbol{\Sigma}_{\mathbf{M}|\mathbf{Y},\mathbf{X},\mathbf{Z}}\boldsymbol{\Delta}_* + (\mathbf{G}_{\mathbf{A}}^T\boldsymbol{\Sigma}_{\mathbf{M}|\mathbf{X},\mathbf{Z}}^{-1}\mathbf{G}_{\mathbf{A}})^{-1} \mathbf{G}_{\mathbf{A}}^T\boldsymbol{\Sigma}_{\mathbf{M}|\mathbf{X},\mathbf{Z}}^{-1}\boldsymbol{\Delta}_* - 2(\mathbf{I}_d + \mathbf{A}^T\mathbf{A})^{-1}\mathbf{A}^T\boldsymbol{\Delta}] + O_p(a_n) = O_p(a_n)$. Based on the calculation in Web Theorem 6, we also have $\operatorname{tr}\{(\boldsymbol{\Omega}^{-1} + \boldsymbol{\eta}\boldsymbol{\Sigma}_{\mathbf{Y}|\mathbf{M},\mathbf{X},\mathbf{Z}}^{-1}\boldsymbol{\eta}^T)\boldsymbol{\Gamma}_1^T\boldsymbol{\Delta}_*^T\boldsymbol{\Gamma}_0 \boldsymbol{\Omega}_0\boldsymbol{\Gamma}_0^T\boldsymbol{\Delta}_*\boldsymbol{\Gamma}_1 + \boldsymbol{\Omega}\boldsymbol{\Gamma}_1^T\boldsymbol{\Delta}_*^T\boldsymbol{\Gamma}_0\boldsymbol{\Omega}_0^{-1}\boldsymbol{\Gamma}_0^T\boldsymbol{\Delta}_*\boldsymbol{\Gamma}_1 - 2(\mathbf{I}_d + \mathbf{A}^T\mathbf{A})^{-1}\boldsymbol{\Delta}_*^T\boldsymbol{\Gamma}_0\boldsymbol{\Gamma}_0^T\boldsymbol{\Delta}_*^T\} \geq k\|\boldsymbol{\Delta}\|_F^2$, where k is a positive constant. Combining all these results, $f_{obj,2}(\hat{\mathbf{A}}_0 + a_n\boldsymbol{\Delta}) - f_{obj,2}(\hat{\mathbf{A}}_0) \geq O_p(a_n^2) + a_n^2 k\|\boldsymbol{\Delta}\|_F^2 + a_n^2(\lambda_{\mathcal{I}}/a_n)\sum_{i=p_{\mathcal{A}}-d+1}^{p_n-d}\|\hat{\mathbf{a}}_i/a_n\|_2$. Since $\hat{\mathbf{A}}_{\mathcal{I}} \neq \mathbf{0}$ and $\|\boldsymbol{\Delta}\|_F \leq 0$, we have $0 < \sum_{i=p_{\mathcal{A}}-d+1}^{p_n-d}\|\hat{\mathbf{a}}_i/a_n\|_2 \leq C$. Because $a_n = o(\lambda_{\mathcal{I}})$, then $\lambda_{\mathcal{I}}/a_n \to \infty$. Therefore $f_{obj,2}(\hat{\mathbf{A}}_0 + a_n\boldsymbol{\Delta}) > f_{obj,2}(\hat{\mathbf{A}}_0)$ with probability tending to 1. In other words, we have established (S30).

Web Table 1: Results of average bias (standard deviation / (number of replications)$^{1/2}$) of the estimate based on 1000 replications, with a sample size of $n = 100$, across three values of the dimension $r = 1, 10, 50$.

Methods	$r = 1$	$r = 10$	$r = 50$
		$\Vert\widehat{\boldsymbol{\beta}}_X - \boldsymbol{\beta}_X\Vert_F$	
ESPLSM	0.745 (0.017)	2.171 (0.017)	4.490 (0.015)
OLS	2.591 (0.065)	9.716 (0.071)	22.406 (0.087)
		$\Vert\widehat{\boldsymbol{\beta}}_{\mathbf{M}} - \boldsymbol{\beta}_{\mathbf{M}}\Vert_F$	
ESPLSM	0.469 (0.005)	1.266 (0.007)	3.060 (0.016)
OLS	13.99 (0.252)	48.055 (0.286)	109.899 (0.359)
		$\Vert\hat{\gamma}_X\widehat{\boldsymbol{\beta}}_{\mathbf{M}} - \gamma_X\boldsymbol{\beta}_{\mathbf{M}}\Vert_F$	
ESPLSM	0.789 (0.017)	2.087 (0.043)	4.083 (0.086)
OLS	2.589 (0.065)	9.804 (0.073)	22.501 (0.090)

Web Appendix C: Further results of simulation study

Simulation study with multiple responses/exposures

We investigate the performance of ESPLSM when there are multiple responses, e.g., $r = 10, 50$, with sample size $n = 100$. The results are summarized in Web Table 1. For comparison, we also include the results for $r = 1$ from Table 1. Since HIMA and HIMA2 are applicable only to the univariate response setting, we do not report their results in this setting. ESPLSM consistently produces lower estimation bias across all values of r. As r increases, the estimation bias increases for both methods, but the relative advantage of ESPLSM remains significant. These results highlights the efficiency of ESPLSM, particularly for estimating mediation

effects in complex models.

We also investigate the performance of ESPLSM in the presence of multiple exposures, where $k = 2$ and the sample sizes are $n = 100$ and 1000. To generate correlated exposures, we set $\mathbf{X} = (X_1, X_2)^T \sim \text{MVN}(\mathbf{0}, \boldsymbol{\Sigma})$, where the covariance matrix $\boldsymbol{\Sigma}$ has diagonal elements equal to 1 and off-diagonal elements equal to ρ. The correlation parameter ρ is varied over $0, 0.3, 0.5$, and 0.75. The results are summarized in Web Table 2. Across all scenarios, ESPLSM demonstrates the best overall performance, yielding the smallest estimation bias for the exposure effects, mediator effects, and indirect effects. Although the bias increases moderately as the exposure correlation strengthens, ESPLSM remains stable and accurate under both moderate and strong correlation, reflecting its ability to efficiently exploit the envelope structure in the presence of correlated predictors. HIMA2 exhibits a distinct sample-size–dependent behavior. When the sample size is relatively small ($n = 100$), HIMA2 shows noticeably larger bias than HIMA. However, as the sample size increases to $n = 1000$, HIMA2 becomes substantially more stable, with improved bias levels across all target quantities. This suggests that HIMA2 benefits from larger sample sizes, under which its multiple-testing and false-discovery–control framework becomes more reliable. In contrast, HIMA shows persistent bias across all scenarios, especially for mediator and indirect ef-

Web Table 2: Results of average bias (standard deviation / (number of replications)$^{1/2}$) of the estimate based on 1000 replications, with a sample size of $n = 100, 1000$, across three values of the correlation between $k = 2$ exposures.

$n = 100$				
Methods	$\rho = 0$	$\rho = 0.3$	$\rho = 0.5$	$\rho = 0.75$
		$\|\widehat{\boldsymbol{\beta}}_{\mathbf{X}} - \boldsymbol{\beta}_{\mathbf{X}}\|_F$		
ESPLSM	0.691 (0.010)	0.706 (0.010)	0.734 (0.011)	0.840 (0.012)
HIMA	1.360 (0.008)	1.370 (0.008)	1.378 (0.008)	1.384 (0.008)
OLS	2.934 (0.069)	2.965 (0.069)	2.964 (0.069)	3.035 (0.068)
		$\|\widehat{\boldsymbol{\beta}}_{\mathbf{M}} - \boldsymbol{\beta}_{\mathbf{M}}\|_F$		
ESPLSM	0.396 (0.005)	0.398 (0.005)	0.396 (0.005)	0.398 (0.005)
HIMA2	4.960 (0.057)	5.042 (0.058)	4.897 (0.056)	4.732 (0.054)
HIMA	3.973 (0.043)	3.938 (0.041)	3.984 (0.043)	4.059 (0.043)
OLS	13.97 (0.250)	13.97 (0.250)	13.97 (0.250)	13.97 (0.250)
		$\|\hat{\boldsymbol{\gamma}}_{\mathbf{X}}\widehat{\boldsymbol{\beta}}_{\mathbf{M}} - \boldsymbol{\gamma}_{\mathbf{X}}\boldsymbol{\beta}_{\mathbf{M}}\|_F$		
ESPLSM	0.663 (0.009)	0.680 (0.009)	0.702 (0.010)	0.792 (0.011)
HIMA2	1.452 (0.038)	1.490 (0.036)	1.445 (0.035)	1.425 (0.037)
HIMA	1.219 (0.008)	1.227 (0.008)	1.236 (0.008)	1.243 (0.008)
OLS	2.927 (0.069)	2.944 (0.069)	2.943 (0.068)	3.005 (0.068)
$n = 1000$				
Methods	$\rho = 0$	$\rho = 0.3$	$\rho = 0.5$	$\rho = 0.75$
		$\|\widehat{\boldsymbol{\beta}}_{\mathbf{X}} - \boldsymbol{\beta}_{\mathbf{X}}\|_F$		
ESPLSM	0.581 (0.003)	0.587 (0.003)	0.592 (0.003)	0.607 (0.003)
HIMA	1.344 (0.002)	1.352 (0.003)	1.357 (0.002)	1.362 (0.002)
OLS	0.830 (0.020)	0.841 (0.020)	0.838 (0.020)	0.860 (0.020)
		$\|\widehat{\boldsymbol{\beta}}_{\mathbf{M}} - \boldsymbol{\beta}_{\mathbf{M}}\|_F$		
ESPLSM	0.303 (0.001)	0.303 (0.001)	0.303 (0.001)	0.303 (0.001)
HIMA2	1.620 (0.018)	1.551 (0.017)	1.500 (0.016)	1.442 (0.016)
HIMA	2.134 (0.011)	2.146 (0.012)	2.149 (0.013)	2.165 (0.013)
OLS	4.022 (0.071)	4.022 (0.071)	4.022 (0.071)	4.022 (0.071)
		$\|\hat{\boldsymbol{\gamma}}_{\mathbf{X}}\widehat{\boldsymbol{\beta}}_{\mathbf{M}} - \boldsymbol{\gamma}_{\mathbf{X}}\boldsymbol{\beta}_{\mathbf{M}}\|_F$		
ESPLSM	0.578 (0.003)	0.584 (0.003)	0.587 (0.003)	0.600 (0.003)
HIMA2	0.955 (0.014)	0.954 (0.013)	0.929 (0.014)	0.906 (0.014)
HIMA	1.248 (0.002)	1.256 (0.003)	1.261 (0.002)	1.266 (0.002)
OLS	0.833 (0.020)	0.842 (0.020)	0.837 (0.020)	0.860 (0.019)

fects, and does not exhibit meaningful improvement even with a large sample size. Finally, OLS performs poorly throughout.

Model misspecification

To evaluate the robustness of the proposed method when the underlying assumptions are violated, we conduct additional simulation studies under various forms of model misspecification.

First, to assess robustness to non-normal errors, we consider error distributions including a t distribution with 3 degrees of freedom, a chi-squared distribution with 4 degrees of freedom, and a uniform distribution defined on the unit interval. The results are summarized in Web Table 3. Across all misspecified settings and sample sizes, ESPLSM demonstrates robust and competitive performance, yielding smaller estimation bias than HIMA and HIMA2 in most scenarios and comparable or superior performance to OLS overall.

Second, we examine the performance of ESPLSM, HIMA, HIMA2, and OLS under unmeasured confounding, where the confounder affects both the mediator and the outcome but is not included in the fitted model. We consider increasing confounding strengths, with $\gamma_{\mathbf{U}} = 2\mathbf{1}_p^T$ and $\beta_{\mathbf{U}} = 2\mathbf{1}_r^T, 6\mathbf{1}_r^T, 10\mathbf{1}_r^T$ under the following data-generating model: $\mathbf{Y} = \boldsymbol{\mu}_{\mathbf{Y}} + \boldsymbol{\eta}^T\boldsymbol{\Gamma}^T(\mathbf{M} - \boldsymbol{\mu}_{\mathbf{M}}) + \boldsymbol{\beta}_{\mathbf{X}}(\mathbf{X} - \mu_{\mathbf{X}}) +$

Web Table 3: Results of average bias (standard deviation / (number of replications)$^{1/2}$) of the estimate based on 1000 replications with model misspecification.

t_3				
Methods	$n = 100$	$n = 300$	$n = 500$	$n = 1000$
		$\|\widehat{\boldsymbol{\beta}}_X - \boldsymbol{\beta}_X\|_F$		
ESPLSM	0.525 (0.011)	0.492 (0.007)	0.480 (0.005)	0.458 (0.004)
HIMA	1.362 (0.003)	1.347 (0.002)	1.341 (0.001)	1.330 (0.001)
OLS	1.419 (0.036)	0.757 (0.019)	0.592 (0.015)	0.414 (0.010)
		$\|\widehat{\boldsymbol{\beta}}_{\mathbf{M}} - \boldsymbol{\beta}_{\mathbf{M}}\|_F$		
ESPLSM	0.359 (0.004)	0.322 (0.001)	0.297 (0.001)	0.280 (0.001)
HIMA2	3.076 (0.046)	1.849 (0.026)	1.439 (0.017)	1.081 (0.013)
HIMA	3.059 (0.025)	2.414 (0.011)	2.151 (0.008)	1.932 (0.005)
OLS	7.717 (0.142)	4.080 (0.079)	3.183 (0.062)	2.259 (0.041)
		$\|\hat{\gamma}_X\widehat{\boldsymbol{\beta}}_{\mathbf{M}} - \gamma_X\boldsymbol{\beta}_{\mathbf{M}}\|_F$		
ESPLSM	0.726 (0.017)	0.516 (0.010)	0.488 (0.009)	0.454 (0.006)
HIMA2	1.750 (0.049)	1.096 (0.028)	0.913 (0.024)	0.734 (0.017)
HIMA	1.438 (0.004)	1.356 (0.002)	1.267 (0.002)	1.195 (0.001)
OLS	1.526 (0.038)	0.798 (0.020)	0.621 (0.016)	0.431 (0.011)
χ^2_4				
Methods	$n = 100$	$n = 300$	$n = 500$	$n = 1000$
		$\|\widehat{\boldsymbol{\beta}}_X - \boldsymbol{\beta}_X\|_F$		
ESPLSM	0.750 (0.016)	0.611 (0.010)	0.544 (0.008)	0.506 (0.006)
HIMA	1.373 (0.004)	1.376 (0.003)	1.367 (0.002)	1.357 (0.001)
OLS	2.459 (0.060)	1.318 (0.030)	0.992 (0.024)	0.695 (0.017)
		$\|\widehat{\boldsymbol{\beta}}_{\mathbf{M}} - \boldsymbol{\beta}_{\mathbf{M}}\|_F$		
ESPLSM	0.452 (0.005)	0.370 (0.002)	0.333 (0.002)	0.307 (0.001)
HIMA2	5.204 (0.063)	3.049 (0.036)	2.294 (0.027)	1.672 (0.020)
HIMA	3.753 (0.032)	2.852 (0.017)	2.480 (0.014)	2.169 (0.008)
OLS	12.95 (0.236)	7.15 (0.119)	5.359 (0.094)	3.752 (0.065)
		$\|\hat{\gamma}_X\widehat{\boldsymbol{\beta}}_{\mathbf{M}} - \gamma_X\boldsymbol{\beta}_{\mathbf{M}}\|_F$		
ESPLSM	0.798 (0.018)	0.615 (0.011)	0.579 (0.009)	0.530 (0.006)
HIMA2	2.817 (0.078)	1.665 (0.041)	1.309 (0.032)	0.976 (0.023)
HIMA	1.451 (0.005)	1.387 (0.003)	1.296 (0.002)	1.222 (0.001)
OLS	2.450 (0.061)	1.344 (0.031)	0.984 (0.024)	0.683 (0.017)

Web Table 3 Continued.

$U(0,1)$				
Methods	$n = 100$	$n = 300$	$n = 500$	$n = 1000$
		$\|\widehat{\boldsymbol{\beta}}_X - \boldsymbol{\beta}_X\|_F$		
ESPLSM	0.138 (0.002)	0.141 (0.001)	0.136 (0.001)	0.133 (0.001)
HIMA	1.336 (0.001)	1.324 (0.0004)	1.312 (0.0003)	1.300 (0.0002)
OLS	0.240 (0.006)	0.131 (0.003)	0.098 (0.002)	0.073 (0.002)
		$\|\widehat{\boldsymbol{\beta}}_{\mathbf{M}} - \boldsymbol{\beta}_{\mathbf{M}}\|_F$		
ESPLSM	0.373 (0.003)	0.376 (0.001)	0.359 (0.001)	0.350 (0.001)
HIMA2	0.609 (0.006)	0.370 (0.004)	0.297 (0.003)	0.224 (0.002)
HIMA	2.217 (0.003)	2.053 (0.001)	1.857 (0.001)	1.710 (0.001)
OLS	1.304 (0.022)	0.718 (0.012)	0.530 (0.009)	0.393 (0.007)
		$\|\hat{\gamma}_X\widehat{\boldsymbol{\beta}}_{\mathbf{M}} - \gamma_X\boldsymbol{\beta}_{\mathbf{M}}\|_F$		
ESPLSM	0.597 (0.014)	0.337 (0.008)	0.261 (0.006)	0.198 (0.004)
HIMA2	0.666 (0.016)	0.367 (0.009)	0.274 (0.007)	0.190 (0.005)
HIMA	1.409 (0.003)	1.333 (0.002)	1.239 (0.001)	1.170 (0.001)
OLS	0.642 (0.015)	0.354 (0.008)	0.262 (0.006)	0.169 (0.004)

$\boldsymbol{\beta}_{\mathbf{Z}}^T(\mathbf{Z}-\boldsymbol{\mu}_{\mathbf{Z}})+\boldsymbol{\beta}_{\mathbf{U}}^T\mathbf{U}+\boldsymbol{\epsilon}$ and $\mathbf{M} = \boldsymbol{\mu}_{\mathbf{M}}+\boldsymbol{\gamma}_{\mathbf{X}}^T(\mathbf{X}-\boldsymbol{\mu}_{\mathbf{X}})+\boldsymbol{\gamma}_{\mathbf{Z}}^T(\mathbf{Z}-\boldsymbol{\mu}_{\mathbf{Z}})+\boldsymbol{\gamma}_{\mathbf{U}}^T\mathbf{U}+\mathbf{e}$, while varying the sample size from $n = 100$ to $n = 1000$. The results are summarized in Web Table 4. When the confounding strength is relatively weak ($\beta_{\mathbf{U}} = 2\mathbf{1}_r^T$), ESPLSM shows the best performance with the smaller bias for the exposure effects, mediator effects, and indirect effects. Even under stronger confounding ($\beta_{\mathbf{U}} = 6\mathbf{1}_r^T$ and $10\mathbf{1}_r^T$), ESPLSM maintains stable overall performance, particularly for estimating mediator effects $\boldsymbol{\beta}_{\mathbf{M}}$. As the confounding strength increases, the bias of HIMA2 grows substantially and remains noticeably larger than that of ESPLSM. In contrast, HIMA achieves smaller bias than ESPLSM for es-

Web Table 4: Results of average bias (standard deviation / (number of replications)$^{1/2}$) of the estimate based on 1000 replications with unmeasured mediator-outcome confounding.

$\boldsymbol{\gamma}_{\mathbf{U}} = 2\mathbf{1}_p^T$ and $\boldsymbol{\beta}_{\mathbf{U}} = 2\mathbf{1}_r^T$				
Methods	$n = 100$	$n = 300$	$n = 500$	$n = 1000$
		$\Vert\widehat{\boldsymbol{\beta}}_X - \boldsymbol{\beta}_X\Vert_F$		
ESPLSM	0.776 (0.018)	0.646 (0.013)	0.557 (0.010)	0.502 (0.008)
HIMA	1.446 (0.002)	1.448 (0.001)	1.446 (0.001)	1.445 (0.001)
OLS	1.054 (0.019)	0.994 (0.011)	0.996 (0.009)	0.989 (0.006)
		$\Vert\widehat{\boldsymbol{\beta}}_{\mathbf{M}} - \boldsymbol{\beta}_{\mathbf{M}}\Vert_F$		
ESPLSM	2.088 (0.012)	2.272 (0.017)	2.263 (0.007)	2.301 (0.004)
HIMA2	2.950 (0.035)	2.983 (0.021)	2.819 (0.018)	2.625 (0.013)
HIMA	4.587 (0.049)	3.315 (0.026)	3.046 (0.022)	2.880 (0.017)
OLS	8.174 (0.088)	5.708 (0.051)	5.195 (0.039)	4.804 (0.029)
		$\Vert\hat{\gamma}_X\widehat{\boldsymbol{\beta}}_{\mathbf{M}} - \boldsymbol{\gamma}_X\boldsymbol{\beta}_{\mathbf{M}}\Vert_F$		
ESPLSM	0.585 (0.009)	0.709 (0.006)	0.518 (0.004)	0.490 (0.003)
HIMA2	1.626 (0.033)	2.311 (0.031)	2.415 (0.024)	2.413 (0.019)
HIMA	1.242 (0.002)	1.457 (0.001)	1.336 (0.001)	0.348 (0.001)
OLS	1.031 (0.018)	1.092 (0.013)	0.976 (0.009)	0.982 (0.007)
$\boldsymbol{\gamma}_{\mathbf{U}} = 2\mathbf{1}_p^T$ and $\boldsymbol{\beta}_{\mathbf{U}} = 6\mathbf{1}_r^T$				
Methods	$n = 100$	$n = 300$	$n = 500$	$n = 1000$
		$\Vert\widehat{\boldsymbol{\beta}}_X - \boldsymbol{\beta}_X\Vert_F$		
ESPLSM	2.562 (0.026)	2.685 (0.016)	2.564 (0.012)	2.541 (0.008)
HIMA	1.638 (0.002)	1.626 (0.001)	1.629 (0.001)	1.627 (0.001)
OLS	3.015 (0.020)	2.987 (0.011)	2.990 (0.009)	2.983 (0.006)
		$\Vert\widehat{\boldsymbol{\beta}}_{\mathbf{M}} - \boldsymbol{\beta}_{\mathbf{M}}\Vert_F$		
ESPLSM	3.155 (0.065)	3.632 (0.046)	4.037 (0.057)	4.002 (0.056)
HIMA2	6.419 (0.062)	8.297 (0.047)	8.841 (0.041)	9.306 (0.030)
HIMA	6.925 (0.058)	6.468 (0.034)	6.407 (0.028)	6.371 (0.020)
OLS	14.75 (0.102)	13.58 (0.054)	13.40 (0.044)	13.27 (0.030)
		$\Vert\hat{\gamma}_X\widehat{\boldsymbol{\beta}}_{\mathbf{M}} - \boldsymbol{\gamma}_X\boldsymbol{\beta}_{\mathbf{M}}\Vert_F$		
ESPLSM	2.358 (0.016)	2.410 (0.011)	2.558 (0.008)	2.626 (0.006)
HIMA2	3.371 (0.038)	4.120 (0.039)	3.739 (0.029)	3.568 (0.020)
HIMA	1.414 (0.002)	1.601 (0.001)	1.519 (0.001)	1.537 (0.001)
OLS	2.810 (0.020)	2.712 (0.014)	2.984 (0.009)	3.069 (0.007)

Web Table 4 Continued.

$\gamma_{\mathbf{U}} = 2\mathbf{1}_p^T$ and $\boldsymbol{\beta}_{\mathbf{U}} = 10\mathbf{1}_r^T$				
Methods	$n = 100$	$n = 300$	$n = 500$	$n = 1000$
		$\Vert\widehat{\boldsymbol{\beta}}_X - \boldsymbol{\beta}_X\Vert_F$		
ESPLSM	3.799 (0.034)	4.000 (0.022)	3.598 (0.018)	3.487 (0.013)
HIMA	1.715 (0.001)	1.699 (0.001)	1.702 (0.001)	1.700 (0.0004)
OLS	5.009 (0.021)	4.981 (0.012)	4.983 (0.009)	4.977 (0.006)
		$\Vert\widehat{\boldsymbol{\beta}}_{\mathbf{M}} - \boldsymbol{\beta}_{\mathbf{M}}\Vert_F$		
ESPLSM	4.552 (0.153)	4.501 (0.088)	3.715 (0.068)	3.517 (0.056)
HIMA2	13.28 (0.107)	16.34 (0.060)	17.10 (0.048)	17.74 (0.034)
HIMA	7.278 (0.044)	7.040 (0.026)	7.020 (0.020)	7.012 (0.014)
OLS	22.84 (0.102)	22.03 (0.059)	21.99 (0.043)	21.89 (0.030)
		$\Vert\hat{\gamma}_X\widehat{\boldsymbol{\beta}}_{\mathbf{M}} - \gamma_X\boldsymbol{\beta}_{\mathbf{M}}\Vert_F$		
ESPLSM	3.42 (0.026)	3.353 (0.016)	3.606 (0.013)	3.666 (0.010)
HIMA2	4.946 (0.035)	5.681 (0.033)	5.152 (0.024)	5.124 (0.017)
HIMA	1.480 (0.001)	1.661 (0.001)	1.589 (0.001)	1.609 (0.0004)
OLS	4.630 (0.020)	4.334 (0.014)	4.992 (0.009)	5.156 (0.007)

timating the exposure effect and the indirect effects under stronger unmeasured confounding, although this advantage does not extend to mediator effect estimation. OLS performs the worst overall, with large bias that increases sharply as the confounding strength grows.

Performance of variable selection

We vary the sample size ($n = 100, 300, 500, 1000$) and noise variance ($\boldsymbol{\Sigma}_{Y|\mathbf{M},X,\mathbf{Z}} = 3\mathbf{I}_r, \mathbf{I}_r, 0.5\mathbf{I}_r, 0.1\mathbf{I}_r$). The average of true positive rate (TPR), true negative rate (TNR), and Precision, computed over 1000 replications, are summarized in Web

Web Table 5: Results Results of TPR, TNR, and Precision for selection consistency based on 1000 replications when the sample size and noise variance vary.

		ESPLSM			SPLS		
$\boldsymbol{\Sigma}_{Y\|\mathbf{M},X,\mathbf{Z}}$	n	TPR	TNR	Precision	TPR	TNR	Precision
$3\mathbf{I}_r$	100	0.636	1	1	0.689	0.573	0.665
	300	0.677	1	1	0.709	0.570	0.667
	500	0.705	1	1	0.728	0.563	0.658
	1000	0.731	1	1	0.757	0.562	0.653
$\mathbf{I}_r$	100	0.949	0.994	0.991	0.743	0.794	0.765
	300	0.988	1	1	0.794	0.800	0.778
	500	0.996	1	1	0.815	0.798	0.772
	1000	1	1	1	0.847	0.823	0.797
$0.5\mathbf{I}_r$	100	0.954	0.995	0.992	0.811	0.777	0.756
	300	0.990	1	1	0.881	0.823	0.807
	500	0.998	1	1	0.912	0.846	0.829
	1000	1	1	1	0.956	0.912	0.899
$0.1\mathbf{I}_r$	100	0.991	0.994	0.992	0.988	0.939	0.935
	300	1	1	1	0.995	0.974	0.970
	500	1	1	1	0.996	0.985	0.981
	1000	1	1	1	0.998	0.993	0.990
		HIMA			HIMA2		
$\boldsymbol{\Sigma}_{Y\|\mathbf{M},X,\mathbf{Z}}$	n	TPR	TNR	Precision	TPR	TNR	Precision
$3\mathbf{I}_r$	100	0.604	0	0.254	0.023	0.997	0.858
	300	0.622	0	0.260	0.064	0.992	0.878
	500	0.662	0	0.273	0.101	0.988	0.896
	1000	0.718	0	0.290	0.235	0.982	0.942
$\mathbf{I}_r$	100	0.744	0	0.297	0.241	0.989	0.965
	300	0.763	0	0.303	0.575	0.940	0.889
	500	0.761	0	0.303	0.653	0.901	0.829
	1000	0.778	0	0.307	0.730	0.832	0.737
$0.5\mathbf{I}_r$	100	0.773	0	0.306	0.470	0.967	0.938
	300	0.801	0	0.313	0.757	0.880	0.811
	500	0.807	0	0.314	0.783	0.837	0.757
	1000	0.879	0	0.333	0.814	0.791	0.706
$0.1\mathbf{I}_r$	100	0.997	0	0.363	0.655	0.884	0.834
	300	1	0	0.364	0.989	0.658	0.643
	500	1	0	0.364	1.00	0.606	0.608
	1000	1	0	0.364	1	0.533	0.566

Table 5. Across all considered settings, ESPLSM achieves near-perfect TNR and precision, while maintaining competitive TPR as sample size increases and noise decreases. SPLS shows improving TPR and TNR as the noise level decreases and the sample size increases; however, its TNRs and precision remain lower than those of ESPLSM. HIMA reports TNRs close to zero, indicating that nearly all mediators are selected regardless of the underlying signal strength. As a result, its variable selection performance lacks sparsity and interpretability in these settings. HIMA2 improves upon HIMA by achieving substantially higher TNRs, reflecting better false discovery control. Nevertheless, its TPRs are markedly lower than those of ESPLSM, SPLS, and HIMA, suggesting a more conservative selection behavior that may miss true sparsity-selected mediators.

We further investigate variable selection performance by considering settings in which the proportion of active mediators varies across 45.5%, 54.6%, 63.6%, 72.7%, and 81.8%, corresponding to 5, 6, 7, 8, and 9 active mediators out of 11 in total. The results provided in Web Table 6 show that ESPLSM demonstrates robust and stable performance across all sparsity levels, maintaining high precision and TNR even as the model becomes increasingly dense. Although its TPR decreases slightly at higher proportions of active mediators, the overall balance between sensitivity and specificity remains favorable. In contrast, SPLS shows a

Web Table 6: Results Results of TPR, TNR, and Precision for selection consistency based on 1000 replications when the proportion of active mediators varies.

% of active	ESPLSM			SPLS		
mediators	TPR	TNR	Precision	TPR	TNR	Precision
45.5	0.822	0.991	0.991	0.992	0.812	0.865
54.6	0.968	0.988	0.991	0.993	0.728	0.852
63.6	0.953	0.984	0.991	0.990	0.355	0.743
72.7	0.828	0.997	0.999	0.983	0.313	0.799
81.8	0.752	0.999	1.000	0.933	0.237	0.852
% of active	HIMA			HIMA2		
mediators	TPR	TNR	Precision	TPR	TNR	Precision
45.5	0.999	0	0.454	0.655	0.882	0.872
54.6	0.986	0	0.542	0.660	0.829	0.872
63.6	0.899	0	0.611	0.691	0.986	0.991
72.7	0.897	0	0.705	0.658	0.972	0.988
81.8	0.899	0	0.802	0.615	0.986	0.996

pronounced degradation in TNR as the proportion of active mediators increases. HIMA continues to select nearly all mediators across all sparsity levels, resulting in TNR equal to zero. While its TPRs remain high, the lack of discrimination between active and inactive mediators limits its utility for sparse mediator identification. HIMA2 achieves high TNRs across most sparsity levels. However, its TPRs are consistently lower than those of ESPLSM, SPLS, and HIMA, leading to reduced overall selection efficiency.

We also report TPR, TNR, and precision under scenarios with weak indirect effects. Specifically, we choose $c_2 = 0.05, 0.07, 0.1, 0.2$ corresponding to the cases displayed in the right panel of Figure 2. The results are summarized in Web

Web Table 7: Results Results of TPR, TNR, and Precision for selection consistency based on 1000 replications when $c_1 = 0.5$ and $c_2 = 0.05, 0.07, 0.1, 0.2$ corresponding to the cases displayed in the right panel of Figure 2.

	ESPLSM			SPLS		
c_2	TPR	TNR	Precision	TPR	TNR	Precision
0.05	0.441	0.792	0.533	0.188	0.909	0.733
0.07	0.556	0.864	0.704	0.271	0.903	0.802
0.1	0.68	0.954	0.900	0.391	0.892	0.836
0.2	0.75	1	1	0.529	0.895	0.867
	HIMA			HIMA2		
c_2	TPR	TNR	Precision	TPR	TNR	Precision
0.05	0.293	0	0.141	0.009	0.990	0.203
0.07	0.327	0	0.155	0.009	0.990	0.227
0.1	0.405	0	0.184	0.009	0.989	0.205
0.2	0.539	0	0.234	0.012	0.988	0.297

Table 7. As expected, all methods show reduced TPRs when indirect effects are weak, highlighting the inherent difficulty of mediator identification in low-signal regimes. Under these challenging conditions, ESPLSM maintains comparatively higher precision and TNRs, particularly for smaller values of c_2, indicating robustness to weak signals while avoiding excessive false positives. As the signal strength increases, its TPRs improve rapidly, leading to a more balanced selection performance. SPLS appears more sensitive to changes in signal strength (i.e., TPR increases with c_2). HIMA exhibits uniformly low precision across all values of c_2, and its TNRs remain close to zero. HIMA2 achieves higher TNRs relative to HIMA; however, its TPRs remain extremely low for small values of c_2.

Simulation study with high-dimensional settings

We investigate the prediction performance of ESPLSM in a high-dimensional setting. To examine scenarios where $n = 100 < p$, we vary the number of mediators ($p = 150, 200, 300, 500$). The data are generated from the ESPLSM model with $k = 1, q = 2, d = 1, r = 1$, $\mathbf{\Sigma}_{Y|\mathbf{M},X,\mathbf{Z}} = 3\mathbf{I}_r$, and $n = 100$. We consider 10 active mediators among p mediators. The matrices $(\mathbf{\Gamma}, \mathbf{\Gamma}_0)$ are obtained by normalizing a $p \times p$ matrix of independent normal $(0, 1)$ variates. We set the last $p-10$ rows of $\mathbf{\Gamma}$ to be zero and normalize the first ten rows such that $\mathbf{\Gamma}^T\mathbf{\Gamma} = \mathbf{I}_d$. Let $\mathbf{A} \in \mathbb{R}^{d\times d}$ be a matrix of independent normal $(3, 1^2)$ variates and $\mathbf{B} \in \mathbb{R}^{(p-d)\times(p-d)}$ be a matrix of independent normal $(0, 0.01^2)$ variates. Define $\mathbf{\Omega} = \mathbf{A}\mathbf{A}^T$ and $\mathbf{\Omega}_0 = \mathbf{B}\mathbf{B}^T$, yielding $\|\mathbf{\Omega}\| = 24.41$ and $\|\mathbf{\Omega}_0\| = 0.20$, where $\|\cdot\|$ denotes the spectral norm. The remaining parameters $\boldsymbol{\eta}, \boldsymbol{\beta}_X, \boldsymbol{\beta}_\mathbf{Z}, \boldsymbol{\gamma}_X, \boldsymbol{\gamma}_\mathbf{Z}$ and the variables $\mathbf{M}, X, \mathbf{Z}$ are generated using the same parameter values and settings as those used to produce the results in Table 1, with $n = 100$.

In this setting, where $n < p$, OLS estimators cannot be computed; therefore, we include SIMPLS as a reference method. The prediction error is calculated using five-fold cross-validation, and the mean prediction error over 100 replications is summarized in Web Table 8. The results show that the ESPLSM estimator consistently achieves the lowest mean prediction error across all values of p.

Web Table 8: Results of average (standard deviation / $(\text{number of replications})^{1/2}$) of the prediction error based on 100 replications in a high-dimensional setting.

Methods	$p = 150$	$p = 200$	$p = 300$	$p = 500$
ESPLSM	1.337 (0.095)	1.329 (0.100)	1.333 (0.081)	1.325 (0.116)
HIMA	3.779 (0.291)	4.522 (0.313)	4.034 (0.321)	3.652 (0.246)
SPLS	8.126 (0.906)	10.225 (0.896)	9.770 (0.633)	9.281 (0.612)

Web Table 9: Results of TPR, TNR, and Precision for variable selection performance based on 100 replications in a high-dimensional setting.

	ESPLSM			SPLS		
	TPR	TNR	Precision	TPR	TNR	Precision
$p = 150$	0.856	0.976	0.764	0.818	0.564	0.377
$p = 200$	0.991	0.932	0.559	0.801	0.530	0.264
$p = 300$	1	0.977	0.674	1	0.160	0.047
$p = 500$	1	0.947	0.291	0.996	0.081	0.032
	HIMA			HIMA2		
	TPR	TNR	Precision	TPR	TNR	Precision
$p = 150$	0.189	0.901	0.121	0.054	1.000	0.969
$p = 200$	0.198	0.919	0.117	0.164	0.999	0.951
$p = 300$	0.299	0.912	0.107	0.089	1.000	0.941
$p = 500$	0.268	0.917	0.063	0.175	1.000	0.941

We investigate the variable selection performance in this setting. The average TPR, TNR, and precision over 100 replications are summarized in Web Table 9. ESPLSM achieves a well-balanced selection performance, maintaining high TPR and TNR while also attaining higher precision. Although the precision of ESPLSM decreases as p increases—reflecting the increasing difficulty of the selection problem—it remains markedly higher than that of SPLS and HIMA. In contrast, SPLS exhibits high TPR but extremely low TNR and precision, particularly

as p grows. HIMA shows relatively high TNR but very low TPR and precision, indicating overly conservative behavior that can fail to recover true active mediators. HIMA2 achieves perfect or near-perfect TNR across all dimensions but at the cost of very low TPR. While its precision is high, this is primarily driven by selecting very few mediators.

Web Appendix D: Further results of data application

The list of the selected RNA expressions among 296 is provided as follows: BTC EGFR FYN PIK3CB MET STRN CREB1 PIK3CA RAC1 VAV1 PIK3AP1 RICTOR MLST8 PTEN GRB2 CHUK ARRB2 IQGAP1 YWHAB PEBP1 CNKSR1 BRCA1 RFC1 RAD51 POLD1 TIPIN PALB2 UIMC1 CLSPN POLE2 NBN PIAS4 RPA3 RFC5 TIMELESS RAD50 RPA2 XRCC3 PCNA RFC3 BRIP1 MDC1 DNA2 BARD1 BRCA2 CCNA2 POLE3 CHEK1 RAD17 PPP4R2 TOPBP1 RFC4 EXO1 ATR POLD4 RMI2 POLE GEN1 RMI1 RAD51D BRCC3 SLX4 MAPK9 TAB1 RIPK2 VRK3 PPP2CA ATF1 UBA52 PTK6 PLCG1 PTPN12 DIAPH1 USP8

References

Dattorro, J. (2010). *Convex optimization & Euclidean distance geometry*. Lulu. com.

Harville, D. A. (1998). Matrix algebra from a statistician's perspective.

Li, B., Chun, H. and Zhao, H. (2012). Sparse estimation of conditional graphical models with application to gene networks. *Journal of the American Statistical Association* **107** 152–167.

Ravikumar, P., Wainwright, M. J., Raskutti, G. and Yu, B. (2011). High-dimensional covariance estimation by minimizing l 1-penalized log-determinant divergence .

Shapiro, A. (1986). Asymptotic theory of overparameterized structural models. *Journal of the American Statistical Association* **81** 142–149.
